# Integrated Transcriptome and Proteome Analyses Reveal Protein Metabolism in *Lactobacillus helveticus* CICC22171

**DOI:** 10.3389/fmicb.2021.635685

**Published:** 2021-06-02

**Authors:** Mengfan Xu, Shanhu Hu, Yiwen Wang, Tao Wang, Piotr Dziugan, Bolin Zhang, Hongfei Zhao

**Affiliations:** ^1^College of Biological Science and Biotechnology, Beijing Key Laboratory of Forest Food Processing and Safety, Beijing Forestry University, Beijing, China; ^2^Institute of Fermentation Technology and Microbiology, Lodz University of Technology, Łódź, Poland

**Keywords:** *Lactobacillus helveticus*, protein-utilization, transcriptome, proteome, mechanism

## Abstract

*Lactobacillus helveticus* is a homofermentative lactic acid bacterium. It is widely used in the fabrication of Swiss cheese and other dairy products. The aim of this study was to elucidate the mechanism by which *L. helveticus* utilizes protein. *Lactobacillus helveticus* CICC22171 were cultured in two different media with various nitrogen sources. The control contained 20 basic amino acids, while the experimental medium contained casein. *De novo* transcriptome and isobaric tags for relative and absolute quantification (iTRAQ) proteome analyses were applied to determine how *L. helveticus* utilizes protein. The casein underwent extracellular hydrolysis *via* ATP-binding cassette (ABC) transporter upregulation and Mn^2+^-associated cell envelope proteinase (CEP) downregulation. Sigma factors and EF-Tu were upregulated and Mg^2+^ was reduced in bacteria to accommodate DNA transcription and protein translation in preparation for proteolysis. Hydrolase activity was upregulated to digest intracellular polypeptides and control endopeptidase genes. In these bacteria, casein utilization affected glycolysis, trehalose phosphotransferase system (PTS), and key factors associated with aerobic respiration and reduced glucose consumption.

## Introduction

*Lactobacillus helveticus* is a homofermentative thermophilic lactic acid bacterium. It is used in the manufacture of Swiss-type and long-ripened Italian cheeses (Kenny et al., 2003). *Lactobacillus helveticus* strains have high nutritional requirements. They require specific exogenous carbon and nitrogen sources, nucleotides, vitamins, and minerals for growth (Smeianov et al., 2007). Previous studies elucidated numerous physiological functions of *L. helveticus*. The living bacteria regulate human intestinal cells and balance the gut microflora. However, bacterial metabolism also produces bioactive components. Polypeptides such as angiotensin I-converting enzyme (ACE) inhibitory peptide regulate human body function, improve sleep quality, and lower blood pressure (Yamamoto et al., 1993; Lopez-Fandinnoa et al., 2006; Pihlanto et al., 2010). *Lactobacillus helveticus* has the highest proteolytic activity in this genus and hydrolyses casein during cheese ripening (Lacou et al., 2015).

Different nutrients in the growth medium have various impacts on the bacterial enzyme system and enable the bacteria to alter their metabolism in response to environmental changes. Nitrogen is a vital element of nucleic acids and proteins. It is the third most abundant element in dry cells after carbon and oxygen and plays important roles in microbial growth (Fang et al., 2020). The growth of *L. helveticus* CRL 1062 did not decrease with ammonium ion content. Hence, the amino acids in the simplified, chemically predefined medium met bacterial nitrogen requirements for biomass synthesis (Hebert et al., 2000). DeMan-Rogosa-Sharpe (MRS) medium is abundant in free amino acids and polypeptides. Its main nitrogen sources are peptone and yeast and beef extracts. In contrast, the concentrations of free amino acids and polypeptides in reconstructed skim milk medium (RSM) are relatively low compared to those in MRS medium. The main carbon and nitrogen source in RSM was casein. Eleven physiological functions were significantly affected by growth medium in mid-log-phase cells. All these increased by approximately 2–6 fold in the stationary phase of bacteria cultured in milk (Kenny et al., 2003). *Lactobacillus casei* ssp. RP0 and *L. helveticus* M10 had the highest hydrolase activity and the latter could use casein as a nitrogen source (Simova and Beshkova, 2007).

Several studies have been conducted on the hydrolytic system of *Lactobacillus lactis*. Casein utilization comprised extracellular protein hydrolysis, polypeptide transport, and intracellular peptide hydrolysis (Guedon et al., 2001). However, lactic acid bacteria strains differ metabolically and few studies have been performed to elucidate the mechanisms of casein utilization and metabolism in *L. helveticus* strains.

Functional genomics and proteomics analyses can clarify the molecular mechanisms of probiotic LAB. Transcriptomics provides information about the global changes that occur in gene expression under specific conditions. However, changes at the post-transcriptional level can only be elucidated by proteomics. Therefore, it is recommended to use both transcriptomics and proteomics to link genomic sequences to their putative biological functions (Laakso et al., 2011).

In the present study, we applied both *de novo* transcriptomics and isobaric tags for relative and absolute quantification (iTRAQ) proteomics to investigate gene and protein expression and disclose the mechanisms involved in casein utilization by *L. helveticus*.

## Materials and Methods

### Bacteria and Chemicals

*Lactobacillus helveticus* CICC22171 were isolated from traditional Chinese cheeses in the microbiology laboratory of Beijing Forestry University. The strain was deposited at the China Industrial Culture Collection (CICC). Glucose, 20 basic amino acids, vitamins (nicotinic acid, calcium pantothenate, pyridoxal, riboflavin, biotin, folic acid, and thiamine), sodium acetate, MgSO_4_, K_2_HPO_4_, MnSO_4_, Tween 80, and casein acid hydrolysate were purchased from Sigma-Aldrich Corp. (St. Louis, MO, United States).

### Culture Media and Conditions

To explore protein metabolism in *L. helveticus* CICC22171, two chemically defined media were designed and named CDMA (control; Hebert et al., 2000) and CDMB (experimental). The compositions of the media are shown in Table 1. They were designed to meet bacterial growth requirements and elucidate the mechanisms by which *L. helveticus* utilizes proteins. All pre-formulated solutions were separately sterilized at 121°C for 15 min and aseptically mixed at equivalent concentrations.

**Table 1:** The two chemically defined mediums of CDMA and CDMB.

CDMA		CDMB	
20 basic amino acids	0.5 g/L each	Casein acid hydrolysate vitamin free	10 g/L
Glucose	20 g/L	Glucose	20 g/L
Nicotinic acid	0.001 g/L	Nicotinic acid	0.001 g/L
Calcium pantothenate	0.001 g/L	Calcium pantothenate	0.001 g/L
Pyridoxal	0.002 g/L	Pyridoxal	0.002 g/L
Riboflavin	0.001 g/L	Riboflavin	0.001 g/L
Biotin	0.001 g/L	Biotin	0.001 g/L
Thiamine	0.001 g/L	Thiamine	0.001 g/L
Sodium acetate	6 g/L	Sodium acetate	6 g/L
K_2_HPO_4_	2 g/L	K_2_HPO_4_	2 g/L
MgSO_4_	0.3 g/L	MgSO_4_	0.3 g/L
MnSO_4_	0.15 g/L	MnSO_4_	0.15 g/L
Tween-80	1.0 ml/L	Tween-80	1.0 ml/L

### Bacterial Cell Preparation

Freeze-dried bacteria were activated with MRS medium and propagated for three generations under anaerobic conditions. Bacteria were cultured in an incubator at 37°C until OD_600_ = 0.6–0.8. The cells were collected by centrifugation at 8,000 × *g* and 4°C for 10 min and washed twice with sterile distilled water. The suspensions were separately inoculated into CDMA and CDMB at 5% w/v.

### *De novo* Sequencing

#### RNA Extraction and Quality Detection

A and B were marked after collecting cell samples from CDMA and CDMB, respectively. RNA was extracted with TRIzol reagent (Invitrogen, Carlsbad, CA, United States) and ribosomal RNA was removed with the mRNA-only kit (Epicenter Biotechnologies, Madison, WI, United States) according to the manufacturer’s instructions. RNA quality and quantity were determined with an Agilent RNA 6000 nano reagents port 1 kit (Agilent Technologies, Santa Clara, CA, United States) and an Agilent 2100 Bioanalyzer, respectively. The mRNA was separated from the total RNA using Sera-Mag Magnetic oligo(dT) particles (Sigma-Aldrich Corp., St. Louis, MO, United States) and chemically fragmented in mortar. The RNA was quantitated and reverse-transcribed into cDNA and the library was constructed and sequenced.

#### Data Quality Control and Assembly

The sequence library was constructed according to the instructions of the Illumina-compatible ScriptSeq™ mRNA-Seq library preparation kit (Illumina, San Diego, CA, United States). The image data output from the Illumina Hiseq2000 sequencing machine (Illumina, San Diego, CA, United States) was transformed by base calling into sequence data (raw data or reads), stored in fast format, and included the sequences, names, and read quality. After fertilization, reads with adaptors, unknown nucleotides >5%, and quality value ≤ 10 in >20% of the instances were discarded. The clean reads were used in the next analysis. *De novo* transcriptome assembly was conducted with the short reads assembling program Trinity to obtain the final unigene.

#### Assembly Data Analysis

All libraries were aligned to the microbial genome with HISAT (Kim et al., 2015). The number of fragments overlapping each unigene was summarized *via* the National Center for Biotechnology Information (NCBI) RefSeq and SwissProt databases. Unigene expression levels were calculated by the fragments per kilobase per million mapped reads (FPKM) method and analyzed by SOAP v. 2.21. FDR ≤ 0.001 and |log2Ratio| ≥ 2 were the differentially expressed gene (DEG) thresholds in the subsequent Gene ontology (GO) and Kyoto Encyclopedia of Genes and Genomes (KEGG) pathway analyses.

### iTRAQ Quantitative Proteomics Determination

#### Protein Extraction, Quantitation, and Quality Assessment

A and B were marked after collecting cells from the control and experimental media. Proteins were extracted and quantitated as described by Wang et al. (2007). Each sample was mixed with 300 μl breaking buffer and 12 μl of 25× protease inhibitor and spun at 0.06 × g and 25°C for 1 min. Each sample was placed on six spoonsful of glass beads, spun for 30 s, and cooled on ice for 30 s. This process was repeated 6×. The samples were then centrifuged at 15,000 × *g* and 4°C for 10 min. Holes were punched in the bottom of an Eppendorf (EP) tube with a syringe needle and the perforated tube was inserted into an intact EP. The tubes were centrifuged at 1,000 × *g* and 4°C for 30 s. The supernatant was collected after centrifugation at 15,000 × *g* and 4°C for 10 min and the protein samples were prepared and stored at 4°C until use. Proteins were quantified with a bicinchoninic acid (BCA) kit (Yanjing Biotechnology Co. Ltd., Shanghai, China). Optical densities were measured at 562 nm on iMark™ microplate reader (BIO-RAD, Hercules, California, United States) and the protein concentrations were calculated by interpolation from a standard curve (y = 0.044x + 0.1263, *R*^2^ = 0.9972). Protein quality was assessed by 12% SDS-PAGE to which a constant 14-mA current was applied for 90 min.

#### Trypsin Hydrolysis

Each 200-μg sample was dissolved in 5 μl of 1 M dithiothreitol (DTT) and incubated at 37°C for 1 h. Then 20 μl of 1 M iodoacetamide (IAM) was added and the mixtures were stored in the dark at room temperature for 1 h. Each sample was centrifuged (15,000 × *g* at 4°C for 10 min) twice with 100 μl ultrapure water and the supernatants were discarded. The samples were then centrifuged (same as above) thrice with 100 μl of 0.5 M NH_4_HCO_3_. Then 50:1 protein: trypsin mixtures were prepared and incubated at 37°C for >12 h.

#### iTRAQ Labeling and Equivalent Mixing

Each 100-μg sample was prepared and labeled according to the protocol of the iTRAQ reagent-8plex multiplex kit (AB SCIEX, Waltham, MA, United States). The samples were then mixed and subjected to LC-MS/MS to determine labeling efficiency.

#### LC-MS/MS Analysis

The samples were separated with an UltiMate™ 3000 LC instrument (Thermo Fisher Scientific, Waltham, MA, United States). Buffer A was 0.1% (v/v) aqueous formic acid and buffer B was 80% (v/v) aqueous acetonitrile. The chromatographic column was balanced with 95% buffer A. The samples were collected with an automatic sampler into a C18 trap column (183 μm; 0.10 × 20 mm) following by separation of the analytical C18 column (181.9 μm; 0.15 × 120 mm) at 600 nl/min. The samples were then analyzed by a Q-Exactive HF mass spectrometer (Thermo Finnigan, Silicon Valley, CA, United States).

### Statistical Analysis

All experiments were performed in triplicate. Data were presented as the mean ± SD, were analyzed by IBM SPSS Statistics v. 19 (IBM Corp., Armonk, NY, United States) and EXCEL 2010 (Microsoft Corp., Redmond, WA, United States). Charts were plotted using Origin v. 8.0 (OriginLab Inc., Northampton, MA, United States). The level of significance was evaluated by one-way ANOVA, and *p* < 0.05 was considered a significant difference.

## Results

### DEG Analysis in *de novo* Sequencing

There were 674 DEGs between experimental group B and control group A. Of these, 269 were upregulated and 405 were downregulated. There were 379 that were mapped to the GO database of which 122 were upregulated and 257 were downregulated.

#### Functional DEG Analysis

**Figure 1:**
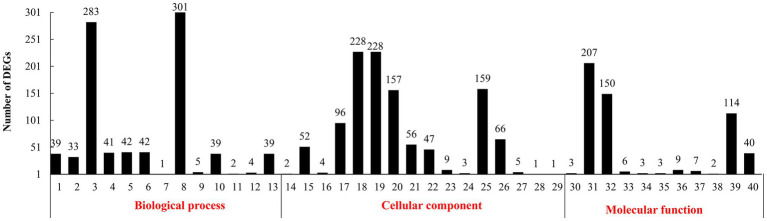
Gene ontology (GO) functional classification of differentially expressed genes (DEGs). (1): biological regulation. (2): cellular component organization or biogenesis. (3): cellular process. (4): developmental process. (5): establishment of localization. (6): localization. (7): locomotion. (8): metabolic process. (9): multi-organism process. (10): multicellular organismal process. (11): negative regulation of biological process. (12): positive regulation of biological process. (13): regulation of biological process. (14): reproduction. (15): response to stimulus. (16): signaling. (17): single-organism process. (18): cell. (19): cell part. (20): macromolecular complex. (21): membrane. (22): membrane part. (23): membrane-enclosed lumen. (24): nucleoid. (25): organelle. (26): organelle part. (27): synapse. (28): virion. (29): virion part. (30): antioxidant activity. (31): binding. (32): catalytic activity. (33): electron carrier activity. (34): enzyme regulator activity. (35): molecular transducer activity. (36): nucleic acid binding transcription factor activity. (37): protein binding transcription factor activity. (38): receptor activity. (39): structural molecule activity. (40): transporter activity.

In the GO annotation (Figure 1), 301 DEGs were found under the top-ranking “metabolic process.” There were 283 DEGs under “cellular process,” 228 under “cell and cell part,” 207 under “binding,” 159 under “organelles,” 157 under “macromolecular complex,” 150 under “catalytic activity,” and 144 under “structural molecule activity.”

**Figure 2:**
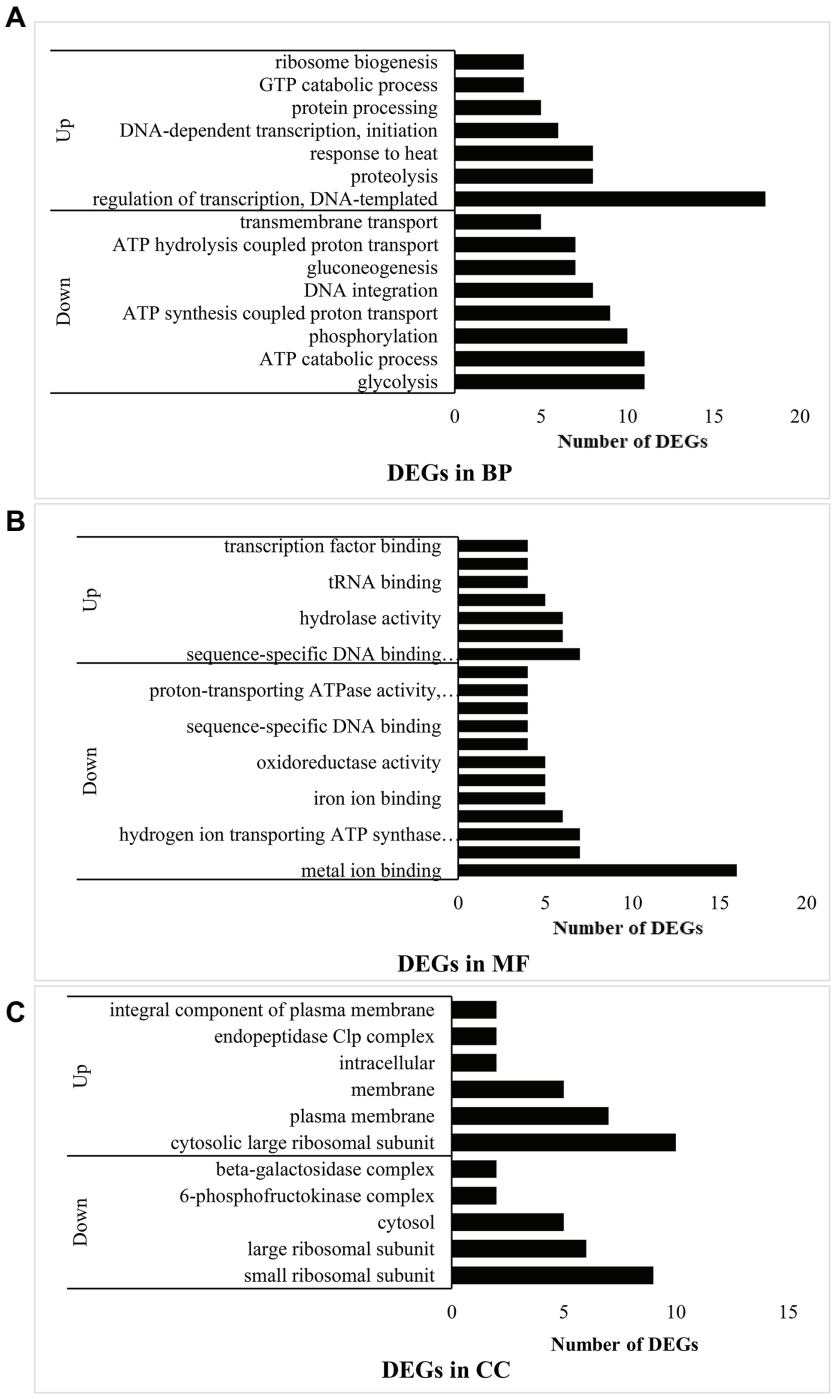
Function analysis of DEGs in B vs. A. **(A)** means DEGs in biological process (BP); **(B)** means DEGs in molecular function (MF); and **(C)** means DEGs in cellular component (CC).

Under the “biological process” (BP) classification (Figure 2A), “DNA-templated transcription regulation” had the most upregulated DEGs and while “glycolysis” had the most downregulated DEGs. The combined results of the GO annotation indicated that in experimental group B, most of the upregulated genes encoded DNA-dependent transcription at the initiation stage, regulation of templated transcription, and ribosome biogenesis at the translation stage. In contrast, processes such as DNA integration were downregulated. However, response to heat and GTP catabolism related to RNA synthesis were upregulated. Protein hydrolysates derived from proteolysis process-related genes were also upregulated. Hence, the bacteria in experimental group B hydrolyzed casein. The downregulated genes were involved mainly in energy metabolism related to glycolysis, ATP catabolism, phosphorylation, synthesis- and ATP hydrolysis coupled proton transport, gluconeogenesis, and transmembrane transport. Thus, when *L. helveticus* uses proteins, protein-related transport and DNA transcription were enhanced, while sugar utilization and ATP conversion pathways were attenuated.

Under the “molecular function” (MF) classification (Figure 2B), regulation of sequence-specific DNA binding transcription factor activity had the most upregulated DEGs, whereas metal ion binding had the most downregulated DEGs. Most of the upregulated genes in experimental group B participated in transcription including the sigma factor at the initiation stage, DNA-directed RNA polymerase, transcription factor binding, sequence-specific DNA binding transcription factor related to the entire transcription process, sequence-specific DNA binding, nucleic acid binding, and nucleotide binding. Peptidase activity and tRNA binding related to peptide hydrolysis and amino acid transport were also upregulated, while protein heterodimerization and metal ion binding were downregulated. The binding of magnesium, iron, heme, manganese, and other ions plays important roles in protein synthesis, the oxidative respiratory chain, and enzymatic hydrolysis. Hydrogen ion-transporting ATP synthase and oxidoreductase affect ATP synthesis. In experimental group B, transcription was induced and enzymes involved in peptide hydrolysis and amino acid transport were synthesized.

Under the “cellular component” (CC) classification (Figure 2C), regulation of the cytosolic large ribosomal subunit had the most upregulated DEGs, while small ribosomal subunit had the most downregulated DEGs. Most of the upregulated genes governed the cytosolic large ribosomal subunit, which collaborates with protein synthesis in bacteria. Other upregulated genes were concentrated in the plasma membrane and other membranes. The endopeptidase Clp complex was associated with protein hydrolysis. *Lactobacillus helveticus* CICC22171 is a facultative anaerobic prokaryote. Its aerobic respiratory chain is localized to the cell membrane, while its anaerobic respiration proceeds in the cytoplasm. Therefore, aerobic respiration and hydrolytic activity may strengthen in the bacteria when they utilized protein. The genes encoding the 6-phosphofructokinase complex in the glycolysis process (EMP) and the β-galactosidase complex were downregulated. As glucose hydrolyzed by β-galactosidase can function in EMP, the outcome is the same as that for biological processes. For this reason, casein in the medium suppresses bacterial glucose utilization.

#### GO and KEGG Pathway Enrichment Analyses of DEGs

The DEGs were mapped to each term in the GO database. Each GO term was calculated using gene numbers. When a corrected *p* ≤ 0.05 was set as a threshold, the GO terms meeting this criterion were defined as significantly enriched in DEGs. In this way, the main biological functions of the DEGs could be identified. Comparison of corrected *P* in groups A and B disclosed that BP was the most significant followed by “cellular component.” Table 2 shows that the most significant processes in BP were “translation” followed by “gene expression,” “cellular protein metabolic process,” and “protein metabolic process.” For CC, the most significant components were the organelle followed by the ribonucleoprotein complex, the cytoplasm, the macromolecular complex, and the ribosomal subunit. For MF, the most significantly enriched DEGs were present in the structural constituents of the ribosomes and structural molecule activity. Hence, when the bacteria used protein as a source of nitrogen, the main DEG functions were related to translation, expression, and protein metabolism as well as the various ribosomal components and functions including the subunit and the protein.

**Table 2:** GO and pathway enrichment analysis of B vs. A.

	Gene ontology term	Corrected *p*-value
BP	Translation	8.37 × 10^−15^
	Gene expression	4.76 × 10^−12^
	Cellular protein metabolic process	1.23 × 10^−11^
	Protein metabolic process	1.21 × 10^−07^
	Cellular macromolecule biosynthetic process	2.50 × 10^−07^
	Macromolecule biosynthetic process	9.61 × 10^−07^
	Organic substance biosynthetic process	5.50 × 10^−05^
	Cellular biosynthetic process	0.00028
CC	Organelle	1.40 × 10^−21^
	Ribonucleoprotein complex	7.99 × 10^−21^
	Cytoplasmic part	3.55 × 10^−20^
	Macromolecular complex	1.38 × 10^−17^
	Ribosomal subunit	2.64 × 10^−06^
MF	Structural constituent of ribosome	8.16 × 10^−26^
	Structural molecule activity	2.81 × 10^−24^
Pathway	Ribosome	0.0000000

Kyoto Encyclopedia of Genes and Genomes is a major public pathway-related database. Various genes collaborate in the execution of their biological functions. A pathway-based analysis was conducted to clarify the biological functions of the genes. A pathway enrichment analysis identified significantly enriched metabolic or signal transduction pathways in the DEGs compared with the entire genome. After multiple testing corrections, pathways with Q-value ≤ 0.05 were identified as significantly enriched in DEGs. Among all the DEGs in B vs. A (Table 2), the most significantly enriched pathway was the ribosome and involved differences among proteins in the large and small ribosome subunits. All DEGs were upregulated except for the S14 protein in the small ribosome subunit. The upregulated DEGs encoded ribosome proteins L3, L4, L2, S19, and S3 related to elongation factor thermo unstable (ET-Tu), ribosome proteins S12 and S7 associated with ET-Tu G, ribosome protein S2 connected to ET-Ts, ribosome proteins L31 and S6 related to RF1, ribosome protein S15 associated with IF2, and ribosome proteins L5, L6, L15, L28, L21, L27, S1, L13, L7/L12, L10, L1, and L11. L7 and L12 are the same protein which has an acylated *N* terminus. L7 and L12 react with the polypeptide chain extension factor ET-Tu, ET-Tu G, and the initiation and termination factors. L11 is involved in peptidyl transferase, while S1 binds mRNA. Thus, protein transcription and translation in *L. helveticus* CICC22171 are significantly stimulated in the presence of a nitrogen source such as casein.

### DEP Analysis in iTRAQ Quantitative Proteomics

Proteome Discoverer v. 1.4 (Thermo Fisher Scientific, Waltham, MA, United States) was used for the iTRAQ quantitative proteomics here. Proteins with fold change (FC) ≥ 1.2 were selected as significant DEPs. Four samples in group B were compared with four samples in group A. Between them, there were 55 upregulated and 52 downregulated DEPs.

#### GO and DEP Pathway Analyses

**Figure 3:**
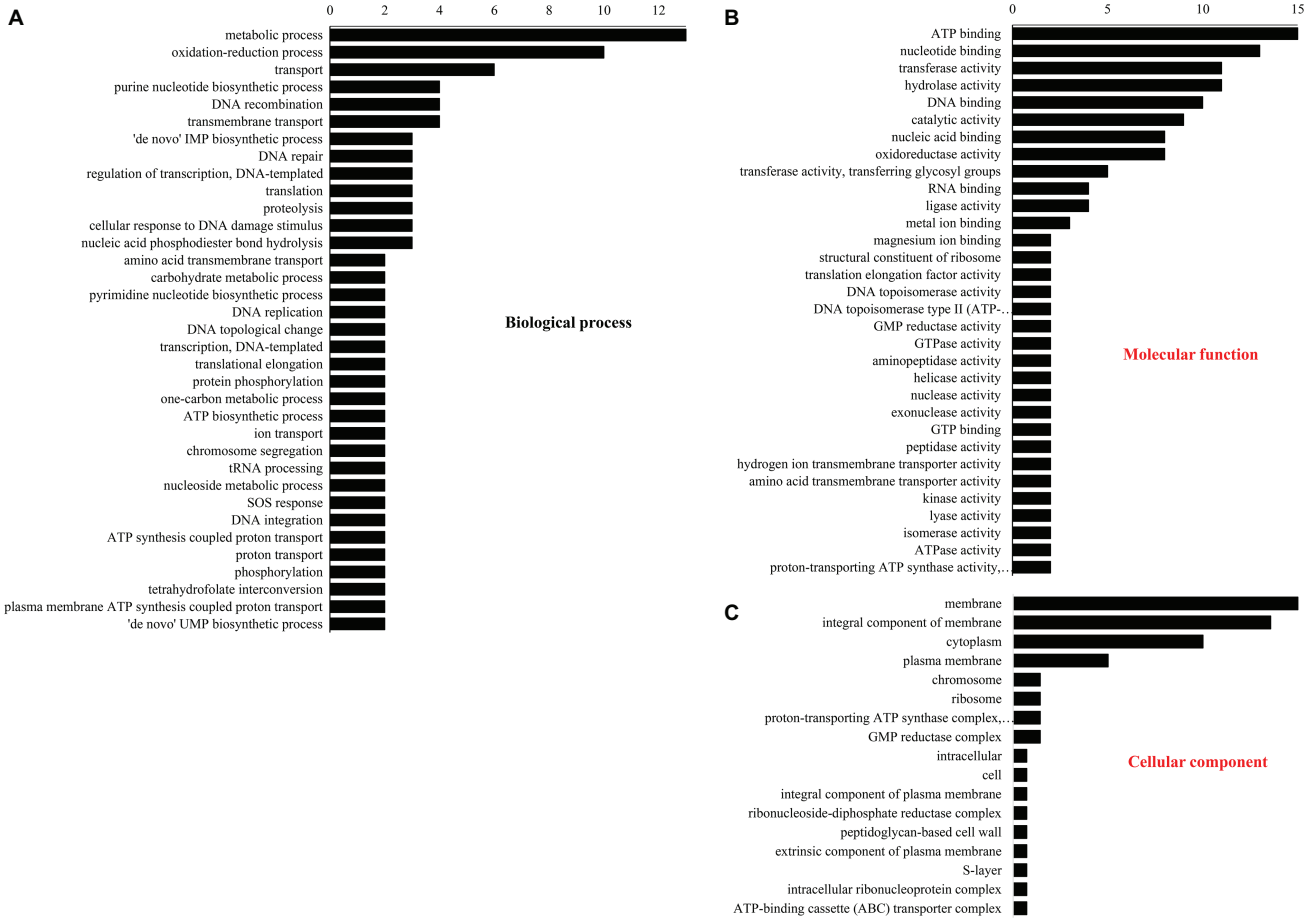
Gene ontology classification of different proteins. (A) Biological process, (B) molecular function, and (C) cellular component.

The number of DEPs in three GO database classifications are shown in Figure 3. In BP (Figure 3A), most DEPs participated in metabolism, redox, purine nucleotide biosynthesis, DNA recombination and transport, and transmembrane transport. Under “molecular function” (Figure 3B), most DEPs were involved in ATP, nucleotide, and DNA binding and transferase and hydrolase activity. Under “cellular component” (Figure 3C), the membrane had the most DEPs followed by the integral components of the cytoplasm, membranes, and plasma membrane.

Fold change > 1.2 in group B vs. group A indicated upregulation, whereas FC < 0.83 showed downregulation. Significantly (*p* < 0.05) upregulated DEPs are shown in Table 3. They included phage integrase-recombinase, uracil-DNA glycosylase, putative pre-16S rRNA nuclease, integrase-recombinase, ATP-binding cassette (ABC) transporter, and UPF0346 protein. Significantly (*p* < 0.05) downregulated DEPs included pyrimidine-nucleoside phosphorylase, putative elongation factor Tu (Fragment), phosphotransferase system (PTS) family mannose porter, and IID component. The significantly upregulated DEPs were related mainly to DNA binding, recombination, and reparation, ribosome function, and transmembrane transport. The significantly downregulated DEPs were associated mostly with the translational elongation stage and carbon source utilization.

**Table 3:** Regulation of DEPs in B vs. A.

	Accession	Description	FC (B vs. A)	*p*-value
Up	A8YVI1	Phage integrase-recombinase	1.26	0.009
	A8YUE7	Uracil-DNA glycosylase	1.28	0.011
	A8YTJ2	Putative pre-16S rRNA nuclease	1.25	0.020
	A8YV49	Integrase-recombinase	1.25	0.028
	F3MN18	ABC transporter	1.25	0.038
	A8YV42	UPF0346 protein	1.22	0.050
Down	F3MNC9	Pyrimidine-nucleoside phosphorylase	0.68	0.007
	Q8KMP9	Putative elongation factor Tu (Fragment)	0.80	0.036
	U6F4E4	PTS family mannose porter, IID component	0.81	0.038

#### GO and DEP Pathway Enrichment Analyses

Whereas GO annotation was analyzed *via* each protein, GO enrichment was analyzed through each GO function. Hence, the data revealed the general characteristics of functional enrichment in all the DEPs (Table 4). Significant pathway enrichment was also analyzed and identified for all proteins by KEGG. Nucleotide and purine nucleoside monophosphate comprised most of the “DNA transcription assembly” process. However, protein translation and modification were reflected mostly in the “tetrahydrofolate metabolism” and “cellular protein modification” processes. In this manner, the primary biochemical and signal transduction pathways in RNA transcription and protein translation were identified.

**Table 4:** GO enrichment analysis of DEPs in B vs. A.

ID	Description	*p*-value
GO:0006464	Cellular protein modification process	0.018
GO:0072522	Purine-containing compound biosynthetic process	0.019
GO:0009165	Nucleotide biosynthetic process	0.021
GO:1901293	Nucleoside phosphate biosynthetic process	0.023
GO:0009156	Ribonucleoside monophosphate biosynthetic process	0.033
GO:0009127	Purine nucleoside monophosphate biosynthetic process	0.039
GO:0009168	Purine ribonucleoside monophosphate biosynthetic process	0.039
GO:0006164	Purine nucleotide biosynthetic process	0.040
GO:0046653	Tetrahydrofolate metabolic process	0.041
GO:0009124	Nucleoside monophosphate biosynthetic process	0.043
GO:0090407	Organophosphate biosynthetic process	0.045
GO:0009163	Nucleoside biosynthetic process	0.045
GO:0042455	Ribonucleoside biosynthetic process	0.045
GO:1901659	Glycosyl compound biosynthetic process	0.045

## Discussion

### Transmembrane Protein Transport

#### Roles of Transmembrane Proteins and Their Associated Ions

**Figure 4:**
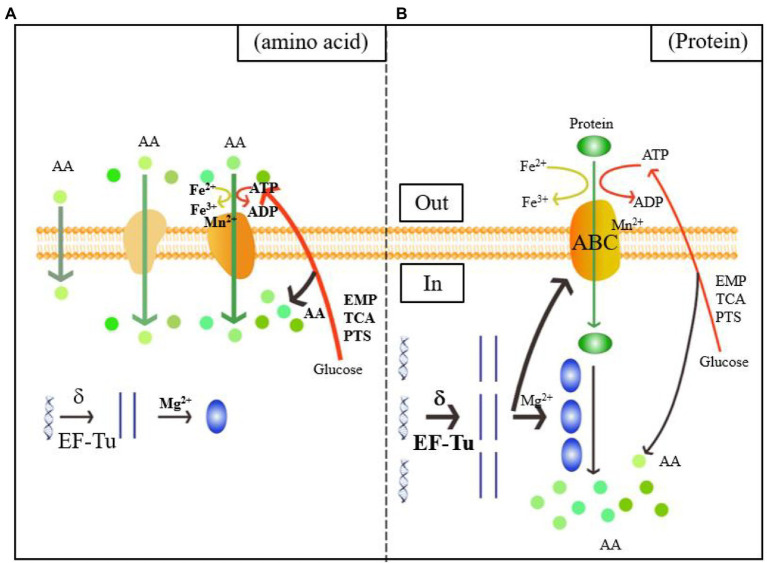
Diagram of the mechanism of the utilization of amino acids **(A)** and proteins **(B)** by *Lactobacillus helveticus* CICC22171. The orange color represents the membrane structure, which includes the phospholipid bilayer, the passive and active transport transporters, and the ATP-binding cassette (ABC) transporters. The boldness of the arrows and the addition of substrates, process enzymes, and products indicate the intensification of the reaction. Green represents the transport of nitrogen sources, spherical represents amino acids, and green ellipse represents proteins. Blue is the central rule. The blue helix is DNA, the straight strand is mRNA, and the blue ellipse is protein. Red indicates carbon source metabolism, ATP transport and amino acid production. The yellow color shows the Fe ion conversion of the electron respiratory chain.

As shown in the green and orange sections of Figure 4, when *L. helveticus* utilized proteins such as casein, it first hydrolyzed them into polypeptides. The DEPs of the ABC transporter were significantly upregulated. The ABC or ATP-binding cassette transporter is a type of transmembrane protein that uses energy from ATP hydrolysis to absorb essential nutrients. In contrast, exporters carry substrates out of the cell by active transport (Theodoulou and Kerr, 2015). The low Mn^2+^ concentration in experimental group B induced cell envelope proteinase (CEP) in *L. helveticus* (Guo et al., 2009). Research on the growth of *L. helveticus* CNRZ32 in skim milk media revealed upregulation of CEPs such as PrtH, PrtH2, and PrtM, the novel proteinases PrtH3 and PrtH5, the Dtp transport system, and peptidases such as pep O2 compared to CNRZ32 grown in MRS (Smeianov et al., 2007). A comparison of *L. helveticus* CRL1062 and CRL974 growth in media containing casitone, casamino acids, or β-casein disclosed that both strains showed the highest growth rates on casitone and the lowest PrtH activity levels in peptide-rich media (Hebert et al., 2000). Research on the caseinolytic properties of six *L. helveticus* strains in cheese revealed that after incubation in milk, all strains produced CEPs that could directly hydrolyze casein. In contrast, strains incubated in MRS did not have this ability (Jensen et al., 2009). Proteolytic genes were upregulated in vitamin-free media with casein acid hydrolysate compared to those with basic amino acids. This finding is consistent with those of previous reports.

Upregulation of DEGs associated with transmembrane transport is rarely seen. In fact, transmembrane transport is usually downregulated. Substitution of nitrogen-derived amino acids with proteins decreases relative transmembrane transport and the metabolic processes associated with it. Thus, casein is transported into cells *via* ABC transporter upregulation and Mn^2+^-associated CEP downregulation.

#### ATP and Energy Utilization Reduction During Transport

Figure 4 shows that the Mg^2+^ concentration was downregulated in B. Therefore, protein utilized by *L. helveticus* strengthens translation. Iron and heme play vital roles in the electron transfer chain (ETC). Low Fe^2+^ concentrations explain a decreased requirement for aerobic respiration. ATP catabolism and phosphorylation and synthesis- and hydrolysis-coupled proton transport were all downregulated. The DEPs regulated ATP synthesis and energy metabolism and included H^+^-ATPase, ATP synthase, and nucleotide-binding activity related to ATP synthesis. Low Mn^2+^ concentrations weaken glycosyltransferase and phosphoenolpyruvate (PEP) carboxykinase activity (Park et al., 1999).

Energy and carbon source metabolism requirements were reduced and these processes were attenuated. Glucose is often the main carbon and energy source for bacterial growth. As a heterotrophic microorganism, *L. helveticus* CICC22171 uses the glycolytic pathway (EMP) as its main energy source. Here, the EMP and PTS family mannose porter genes were downregulated in media containing casein. Thus, casein inhibits glucose utilization. The downregulated DEPs of the mannose PTS include a special transmembrane protein (Reizer and Saier, 1997) called the PTS family mannose porter. Its IID component identifies, transports, and phosphorylates carbohydrates. The PTS family mannose porter may operate in glucose and fructose transport systems (Saulnier et al., 2007). More sugar transport systems (including the PTS family and the mannose or fructose PTS family porter) were upregulated in *L. casei* Zhang grown on milk than in those cultured on soymilk. Hence, numerous carbohydrates could sustain bacterial growth (Wang et al., 2013). The alternate carbon source for *L. lactis* replaced glucose when the content of the latter had decreased because the enzymes involved in rapid carbon metabolism were downregulated (Redon et al., 2005). Hence, lactic acid bacteria could utilize alternate carbon sources by regulating the associated genes. Lactic acid bacteria require energy from glycometabolism to assist their respiration. Hence, bacterial respiration is strongly influenced by environmental conditions (Pedersen et al., 2012). Further studies should be conducted on the respiratory chains of bacteria in various media to optimize *L. helveticus* CICC22171 growth and metabolism.

The present study showed that bacterial energy metabolism declines with active transport and the respiratory ETC. Glycolysis generates amino acid carbon skeletons. The observed reduction in sugar utilization indicates that protein as a nitrogen source reduced carbon skeleton biosynthesis in *Lactobacillus* strains and compensated for the amino acid deficiency in the A medium.

### Intracellular Protein Hydrolysis

#### DNA Transcription and Protein Translation in Preparation for Hydrolysis

The blue section of Figure 4 shows that transcription was the first step in gene expression and the sigma factor was vital to this process. The sigma factor activates initial transcription by reversibly binding the active site of RNA polymerase (Helmann and Chamberlim, 1988). Phage integrase-recombinase is a site-specific recombinase and includes the tyrosine and serine families (Stoll et al., 2002). It mediates DNA recombination by covalent interactions. The tyrosine family acts on the DNA skeleton *via* the tyrosine hydroxyl and binds the DNA chain after breakage (Groth and Calos, 2004). Uracil-DNA glycosylase effects DNA repair by preventing and correcting mispairing. It recognizes and hydrolyzes the *N*-glycosidic linkages between deoxyribose and the wrong base (Sakumi and Sekiguchi, 1990). Transcription factors functionally couple sigma factor regulons under harsh environments and substantially modulate transcription (Binder et al., 2016). Elongation factors Tu (EF-Tu) were the most abundant proteins in translation. They play vital roles in protein synthesis elongation in prokaryotes, mitochondria, plastids, and plasmids (Fu et al., 2012). Here, the sigma factor was upregulated after the nitrogen source in the medium was replaced with acid-hydrolyzed casein. This finding aligned with the observed upregulation of various enzymes involved in transcription. Therefore, transcription may be induced when the bacteria utilize casein.

The putative pre-16S rRNA nuclease is in the ribosome biogenesis subprocess under the GO classification “biological process.” It might have hydrolase activity on the 5′ end of pre-16S rRNA and attack ester bonds. Pyrimidine-nucleoside phosphorylase is mainly involved in nucleic acid systems. It might catalyze pyrimidine nucleoside hydrolysis and add phosphate groups to the receptor. The putative elongation factor Tu is related to the translational elongation stage. Low Mg^2+^ concentrations promote the synthesis of large and small ribosome subunits and assist in protein translation (Chakraborty et al., 2019). Here, the Mg^2+^ level was decreased in B. This discovery confirmed that the protein utilized by *L. helveticus* could stimulate protein translation. A few studies examined how casein influences gene expression during *L. helveticus* growth (Chen et al., 2019; Zhao et al., 2019). However, most of them concluded with analyses of the expression levels of the enzymes involved in bacterial hydrolysis and did not address the changes occurring in transcription or translation. Hence, the results of the present work could lay a foundation for further investigations into the influences of casein media on protein synthesis in *L. helveticus* CICC22171 and the regulatory mechanisms of the enzymes and factors involved in transcription and translation.

Proteomics here disclosed that phage-integrase recombinase was upregulated in strain CICC22171 cultured on casein media. This enzyme participates in DNA recombination. A previous study reported that several phage-related genes, the small and large phage terminase subunits, and phage proteins were upregulated in *L. helveticus* CNRZ32 grown in milk media (Smeianov et al., 2007). However, no genes encoding phage cytase were confirmed for strain CICC22171. Thus, further study is required to validate prophage induction in DNA recombination and autolysis in *L. helveticus* CICC22171.

#### Protein Activity Was Enhanced to Promote Intracellular Hydrolysis

Proteolysis was induced to acquire the essential nutrients needed to sustain bacterial growth in media deficient in free amino acids. Proteins were hydrolyzed into peptides which were then transported to the cells. The endopeptidase Clp complex in the bacteria generated low-molecular-weight peptides and liberated amino acids from the casein matrix. The *L. helveticus* proteolytic system is very efficient and comprises numerous enzymes with various functions. Protein synthesis may vary with medium composition. The present study verified that the bacterial media primarily influenced protein metabolism.

The medium constituents also influenced the expression of the genes encoding protein transport and intracellular proteolysis. A study on *L. helveticus* CNRZ32 demonstrated that expression of the peptidase pepI was higher in MRS than skim milk media, whereas the opposite was true for the peptidases pep N, pep X, and pep R. However, the expression levels of the peptidase pepC were the same in both media (Smeianov et al., 2007). An investigation of *L. helveticus* CRL1062 and CRL974 revealed that peptidase pep N was not influenced by the nitrogen source in the medium as its expression levels were similar in both MRS and casitone (Hebert et al., 2000). Therefore, the impact of nitrogen source on peptidase activity varies with bacterial strain. In contrast, an analysis of *L. casei* Zhang cultured in bovine milk and soymilk revealed that its proteasome, oligopeptide transport, and peptidase systems were upregulated in soymilk. For this reason, the soymilk contained sufficient free amino acids to support bacterial growth. The *L. helveticus* membrane had a major influence, while that of its cytoplasm was minor. Thus, there was an evident aerobic effect. However, gene transcription, RNA translation, related genetics, and transmembrane metabolism were promoted, while energy metabolism was inhibited. ABC transporter upregulation may move the hydrolysates out of the cell so that protein metabolism may be completed.

## Conclusion

*Lactobacillus helveticus* CICC22171 delivered proteins by upregulating the ABC transporter and downregulating Mn^2+^-associated CEP. The sigma factor and EF-Tu were upregulated and Mg^2+^-associated enzyme expression was downregulated in the bacteria to effect DNA transcription, protein translation, and enzymatic proteolysis. CICC22171 completed intracellular polypeptide hydrolysis by upregulating hydrolases which, in turn, controlled endopeptidase genes. Glycolysis, the mannose PTS family, and proteins associated with iron and heme ions related to aerobic respiration were all downregulated in CICC22171 that could assimilate casein. Consequently, bacterial energy metabolism was attenuated. This study revealed the molecular processes of the transport and enzymatic hydrolysis of *L. helveticus* utilizing proteins, and proposed the mechanism of reducing energy consumption. This provides the basis for the economical and efficient utilization of protein as the nitrogen source of *Lactobacillus* strains.

## Data Availability Statement

The original contributions presented in the study are publicly available. This data can be found here: the transcriptome data presented in the study is deposited in the NCBI BioSample Submissions (SRA) repository, accession number SUB9545693. The proteome data presented in the study is deposited in the ProteomeXchange repository, accession number PXD025919.

## Author Contributions

MX: conceptualization, writing – original draft preparation, and data curation. HZ: methodology, software, and validation. YW: formal analysis and project administration. BZ: investigation, visualization, supervision, and funding acquisition. TW and HZ: resources. MX and YW: writing – review and editing. All authors contributed to the article and approved the submitted version.

### Conflict of Interest

The authors declare no conflict of interest. The funders had no role in the design of the study; in the collection, analyses, or interpretation of data; in the writing of the manuscript, or in the decision to publish the results.
