# Supplementary material for: Integrated Transcriptome and Proteome Analyses Reveal Protein Metabolism in Lactobacillus helveticus CICC22171
Source: Front Microbiol. 2021 Jun 2;12:635685. doi: 10.3389/fmicb.2021.635685 (PMC8206810; doi:10.3389/fmicb.2021.635685)
Supplement: Supplementary file 1 [file Data_Sheet_1.PDF]

Accession	Description	Score	Coverage	Prote	ue Pe	# Peptide	# PSMs	A1/all
U6F7I8	Pyruvate kinase OS=Lactobacillus helveticus CIRM-B	23013.97	76.23	1	4	51	1717	0.910
A8YUV4	Enolase OS=Lactobacillus helveticus (strain DPC 457	21730.00	89.25	1	1	30	1171	1.112
A8YTH8	60 kDa chaperonin OS=Lactobacillus helveticus (stra	19701.20	84.63	75	33	44	1116	0.958
A8YUS2	Elongation factor Tu OS=Lactobacillus helveticus (st	19311.93	80.30	8	3	30	1358	1.037
U6F3N6	Glyceraldehyde-3-phosphate dehydrogenase OS=La	19269.88	92.60	2	4	27	1469	0.906
F3MKM9	Enolase OS=Lactobacillus helveticus MTCC 5463 GN	19063.26	89.25	5	1	30	1116	1.130
A0A0R2MSR1	Pyruvate kinase OS=Lactobacillus helveticus GN=IV	16240.11	71.48	3	2	47	1481	1.077
C5J2C0	Tuf (Fragment) OS=Lactobacillus helveticus PE=4 S	14516.86	82.33	3	1	20	1005	1.061
U6F487	Inosine-5-monophosphate dehydrogenase OS=Lact	13780.79	87.89	1	5	29	788	1.052
W5XD07	Inosine-5-monophosphate dehydrogenase OS=Lact	13373.75	83.85	1	1	25	734	0.788
A0A0N9ZZH4	Glyceraldehyde-3-phosphate dehydrogenase OS=La	13317.63	87.87	1	1	25	1228	1.118
C5J2E8	Tuf (Fragment) OS=Lactobacillus helveticus PE=4 S	13170.98	83.19	2	1	17	910	1.056
A8YUE3	Phosphoglycerate kinase OS=Lactobacillus helveticu	13037.47	90.57	7	33	34	1026	0.981
A0A0P0A3H8	Fructose-bisphosphate aldolase OS=Lactobacillus he	12913.35	87.79	6	4	32	1004	1.032
J3XAK1	Elongation factor Tu OS=Lactobacillus helveticus R0	12755.84	68.43	35	1	25	987	1.047
Q8KMP9	Putative elongation factor Tu (Fragment) OS=Lactob	11832.57	75.20	2	1	18	761	0.921
J3X9R4	Fructose-bisphosphate aldolase OS=Lactobacillus he	10913.84	81.52	4	1	29	895	1.031
C7B9P9	Elongation factor Tu (Fragment) OS=Lactobacillus h	10458.65	71.21	31	1	17	755	1.076
A4ZH05	Inosine-5'-monophosphate dehydrogenase OS=Lact	10021.42	77.37	6	3	26	635	1.053
U6F3D9	2,3-bisphosphoglycerate-dependent phosphoglycera	9490.06	87.39	8	4	19	798	1.009
U6F7E1	Bifunctional purine biosynthesis protein PurH OS=La	9274.00	77.78	1	4	48	733	0.632
A4ZH18	Bifunctional purine biosynthesis protein PurH OS=La	9022.99	74.12	6	1	45	698	0.821
A8YXK3	Elongation factor G OS=Lactobacillus helveticus (str	8952.58	70.01	7	43	43	702	0.978
A0A0R2MMY3	Inosine-5-monophosphate dehydrogenase OS=Lact	7611.23	63.95	4	1	19	546	1.072
A8YVD9	Phosphoenolpyruvate carboxylase OS=Lactobacillus	7398.87	75.00	14	63	64	541	0.997
C5J2B1	Tuf (Fragment) OS=Lactobacillus helveticus PE=4 S	7316.72	69.11	1	1	14	610	0.826
E5FN96	Hsp60 (Fragment) OS=Lactobacillus helveticus GN=	7227.07	74.85	6	1	11	360	1.094
U4QDP6	2,3-bisphosphoglycerate-dependent phosphoglycera	7025.03	76.52	2	1	16	654	1.233
C4MCM0	Serine hydroxymethyltransferase OS=Lactobacillus h	6708.38	73.97	5	10	29	509	1.068
W5XHS0	Aminopeptidase N OS=Lactobacillus helveticus H9 G	5943.10	72.39	18	1	57	709	0.934
A8YW85	Phosphoribosylformylglycinamidine synthase subunit	5814.95	59.08	7	36	37	456	0.733
Q076V2	Aminopeptidase N OS=Lactobacillus helveticus GN=	5742.34	72.39	24	1	57	700	0.933
U6F1G0	Endopeptidase O3 OS=Lactobacillus helveticus CIRM	5413.64	74.34	19	47	50	438	0.996
U6F3G8	Xylulose-5-phosphate phosphoketolase OS=Lactoba	5394.24	62.58	11	10	48	781	0.940
A8YVX0	Bifunctional protein FolD OS=Lactobacillus helveticu	5383.31	86.88	12	12	20	359	1.018
O32765	L-lactate dehydrogenase OS=Lactobacillus helveticu	5227.07	69.35	2	1	21	505	0.832
A0A0P0A4G1	L-lactate dehydrogenase OS=Lactobacillus helveticu	5226.67	69.35	7	1	21	501	1.011
A8YW74	Adenosylcobalamin-dependent ribonucleoside-tripho	5142.03	68.01	8	46	46	515	0.731
U6F568	Surface layer protein OS=Lactobacillus helveticus CI	5097.73	76.22	24	11	33	583	1.302
U6F275	ATP-dependent Clp protease OS=Lactobacillus helve	5088.20	68.03	29	54	56	505	0.994
C4MCL9	Glutamine synthetase OS=Lactobacillus helveticus C	5049.49	82.47	4	2	34	708	0.808
A8YVQ3	Chaperone protein DnaK OS=Lactobacillus helveticu	5032.23	66.78	12	2	42	519	0.886
U6F571	Aminopeptidase C OS=Lactobacillus helveticus CIRM	5013.75	75.28	13	5	38	422	1.032
A8YXJ9	DNA-directed RNA polymerase subunit beta' OS=La	4934.24	69.10	5	5	82	439	0.958
A8YTF2	50S ribosomal protein L7/L12 OS=Lactobacillus helv	4919.32	88.33	5	1	13	313	0.867
A8YUE4	Triosephosphate isomerase OS=Lactobacillus helvet	4905.72	87.70	7	22	22	436	0.972
A8YUK1	ATP synthase subunit beta OS=Lactobacillus helveti	4769.93	65.14	6	24	24	310	1.022
A0A0D5MI40	Glutamine synthetase OS=Lactobacillus helveticus G	4743.08	82.47	1	1	33	684	1.170
A0A0F7H323	Phosphoglucomutase OS=Lactobacillus helveticus G	4607.90	78.05	1	3	44	423	0.904
A8YV22	ATP-dependent 6-phosphofructokinase OS=Lactoba	4532.25	87.50	6	25	26	420	0.901
A0A0R2MNW6	Phosphoketolase OS=Lactobacillus helveticus GN=I	4514.24	55.14	2	1	40	628	1.006
A0A0D5MJD8	Glucose-6-phosphate isomerase OS=Lactobacillus h	4468.24	56.95	4	27	27	476	0.989
U6F1M1	Ribonucleoside-diphosphate reductase OS=Lactoba	4457.09	60.53	10	4	46	445	0.801
U6F9S1	DNA-directed RNA polymerase subunit beta' OS=La	4404.37	66.14	3	1	78	399	1.049

J4BP89	Serine hydroxymethyltransferase OS=Lactobacillus h	4400.16	50.85	2	1	20	335	1.172
A0A0D5MI34	Aminopeptidase C OS=Lactobacillus helveticus GN=	4340.44	69.49	12	3	36	375	0.937
A0A0D5MH92	Ribose-phosphate pyrophosphokinase OS=Lactobac	4252.38	50.62	10	3	18	273	0.927
U6F050	Glutathione-disulfide reductase OS=Lactobacillus he	4249.17	83.18	4	30	30	321	1.079
U6F738	Glutamine--fructose-6-phosphate aminotransferase	4201.14	68.49	15	35	36	283	0.901
U6F4D4	Adenylosuccinate lyase OS=Lactobacillus helveticus	4171.51	80.79	7	35	35	464	0.657
A0A0N9ZCJ9	Phosphoglucomutase OS=Lactobacillus helveticus G	4159.38	80.66	3	4	45	414	0.910
A8YWE1	Threonine--tRNA ligase OS=Lactobacillus helveticus	4100.16	68.48	9	40	40	506	0.887
A0A0F7H2F1	Glutamate--tRNA ligase OS=Lactobacillus helveticus	4093.20	75.95	7	42	42	403	0.878
U6F8M2	Phosphoribosylaminoimidazole-succinocarboxamide	4088.50	89.08	14	23	23	494	0.785
A8YVZ3	Carbamoyl-phosphate synthase large chain OS=Lact	4038.06	66.10	14	63	63	409	0.737
A0A0R2MUZ0	Chaperone protein DnaK OS=Lactobacillus helveticu	4020.12	60.42	6	1	41	462	0.756
A8YVR9	30S ribosomal protein S2 OS=Lactobacillus helveticu	3941.31	73.54	5	18	18	357	0.921
A0A0F7H674	Ribonucleoside-diphosphate reductase OS=Lactobac	3880.74	57.48	2	2	44	382	0.703
U6F5Z1	Xaa-Pro dipeptidyl-peptidase OS=Lactobacillus helve	3830.56	64.19	5	2	44	342	1.079
A8YVZ9	Dihydroorotate dehydrogenase A (fumarate) OS=La	3797.43	67.75	7	2	13	210	0.958
A0A0D5MK11	Phosphoenolpyruvate-protein phosphotransferase O	3779.87	59.97	6	27	27	308	0.926
A0A0F7CZU0	Aminopeptidase OS=Lactobacillus helveticus GN=TU	3741.23	69.79	34	25	27	271	1.065
J7LHB7	D-lactate dehydrogenase OS=Lactobacillus helveticu	3739.59	74.48	1	1	25	457	0.938
U6F006	D-lactate dehydrogenase OS=Lactobacillus helveticu	3727.54	81.01	3	1	27	473	1.017
U6F9Y9	Branched-chain-amino-acid aminotransferase OS=La	3720.70	72.14	7	27	27	391	1.115
U6F2V3	Endopeptidase O OS=Lactobacillus helveticus CIRM-	3671.34	79.60	12	41	41	317	0.995
A0A0N9Z9E9	Lactate dehydrogenase OS=Lactobacillus helveticus	3627.86	76.56	4	1	26	459	0.988
A0A0N9ZI95	Pyridoxamine 5'-phosphate oxidase OS=Lactobacillu	3618.87	90.91	2	1	8	358	1.070
U6F2V8	Elongation factor Ts OS=Lactobacillus helveticus CIR	3605.09	69.79	6	23	23	289	0.947
A0A0D5MIC6	Xaa-Pro dipeptidyl-peptidase OS=Lactobacillus helve	3571.99	62.67	10	1	43	331	0.883
U6F0M8	DNA-directed RNA polymerase subunit beta OS=Lac	3496.28	60.51	11	71	73	397	0.947
P30901	D-lactate dehydrogenase OS=Lactobacillus helveticu	3485.99	75.96	2	1	26	445	1.126
A8YUJ9	ATP synthase subunit alpha OS=Lactobacillus helvet	3361.26	56.66	13	28	28	297	0.976
A0A0F7H6Q9	6-phosphogluconate dehydrogenase, decarboxylatin	3344.96	66.81	6	28	28	341	0.920
A8YXL7	50S ribosomal protein L5 OS=Lactobacillus helveticu	3336.27	78.33	4	17	17	268	0.842
A8YVM4	Glycine--tRNA ligase beta subunit OS=Lactobacillus	3308.71	55.75	9	42	43	271	0.901
U6F3H4	GMP synthase [glutamine-hydrolyzing] OS=Lactobac	3301.61	67.05	11	34	34	284	0.827
U6F495	Methionine--tRNA ligase OS=Lactobacillus helveticus	3290.65	71.58	9	48	48	345	1.006
A8YTK4	Adenylosuccinate synthetase OS=Lactobacillus helve	3273.48	68.07	8	30	30	306	0.586
W5XL06	Ribose-phosphate pyrophosphokinase OS=Lactobac	3259.69	46.30	2	1	16	216	1.006
A0A0D5MIG7	Ferritin OS=Lactobacillus helveticus GN=HUO_0599	3196.17	96.13	2	1	10	195	1.031
A8YV34	30S ribosomal protein S1 OS=Lactobacillus helveticu	3190.58	55.33	10	24	24	285	0.804
A8YXK5	50S ribosomal protein L3 OS=Lactobacillus helveticu	3039.53	72.17	3	18	18	263	0.789
A0A0F7H424	Dipeptidase OS=Lactobacillus helveticus GN=IV62_(	3017.75	54.89	6	24	24	242	0.955
Q8VW91	Surface layer protein OS=Lactobacillus helveticus GI	3001.44	46.10	13	1	23	408	1.242
A0A0D5MHK9	50S ribosomal protein L7/L12 OS=Lactobacillus helv	2975.79	88.33	2	1	13	265	1.028
A0A0R2M634	30S ribosomal protein S7 OS=Lactobacillus helveticu	2965.81	78.85	3	13	13	198	0.811
U6F5D6	L-lactate dehydrogenase OS=Lactobacillus helveticu	2925.69	64.61	7	17	17	162	0.962
U4QMJ6	Citrate lyase alpha-chain OS=Lactobacillus helveticu	2840.24	71.54	12	37	37	260	0.938
A8YXK6	50S ribosomal protein L4 OS=Lactobacillus helveticu	2796.39	59.51	4	12	12	211	0.807
J3ZEM7	Fumarate hydratase class II OS=Lactobacillus helve	2779.99	71.95	31	8	30	316	0.984
A0A0N9Z9J5	Ferritin OS=Lactobacillus helveticus GN=ALV80_050	2778.59	96.13	1	1	10	144	1.091
U6F5C8	Dipeptidase A OS=Lactobacillus helveticus CIRM-BIA	2693.87	67.09	10	30	30	304	1.077
P94870	Aminopeptidase E OS=Lactobacillus helveticus GN=p	2668.16	76.48	8	2	29	214	0.844
A8YTZ6	Glutamyl-tRNA(Gln) amidotransferase subunit A OS=	2636.87	71.61	4	1	31	240	0.951
A8YVQ7	Translation initiation factor IF-2 OS=Lactobacillus he	2626.05	59.43	19	53	54	295	0.972
U6F5U0	Uncharacterized protein OS=Lactobacillus helveticus	2624.60	47.43	9	4	41	256	1.088
U6F634	Isoleucine--tRNA ligase OS=Lactobacillus helveticus	2621.47	59.22	10	51	51	281	0.989
A8YTE3	50S ribosomal protein L1 OS=Lactobacillus helveticu	2616.44	70.43	1	16	16	211	0.802

W5XDT1	Glutamyl-tRNA(Gln) amidotransferase subunit A OS=	2615.52	65.76	1	1	30	237	1.256
A8YXA8	Endopeptidase F OS=Lactobacillus helveticus (strain	2607.51	41.47	21	22	23	285	1.023
U6F013	Uncharacterized protein OS=Lactobacillus helveticus	2603.71	75.79	8	21	21	137	1.010
A0A0D5MI86	DNA-binding protein OS=Lactobacillus helveticus GN	2556.61	72.53	3	8	8	140	1.054
A8YXS1	3-hydroxyacyl-[acyl-carrier-protein] dehydratase Fab	2520.78	54.05	3	9	9	183	1.264
A0A0F7H3F7	Aminopeptidase OS=Lactobacillus helveticus GN=TU	2504.43	76.48	1	2	29	218	1.153
U6F6M9	Formate--tetrahydrofolate ligase OS=Lactobacillus h	2496.46	54.07	6	5	30	234	0.828
Q2VWA7	Putative prolidase OS=Lactobacillus helveticus GN=p	2480.07	67.75	8	4	22	174	1.115
A0A0R2MJQ8	Dihydroorotate dehydrogenase OS=Lactobacillus he	2449.96	58.96	1	1	11	151	0.841
U6F670	Cell envelope-associated proteinase OS=Lactobacillu	2431.20	46.23	5	77	84	264	0.984
A0A0R2MMF3	Glutamyl-tRNA(Gln) amidotransferase subunit A OS=	2399.14	58.04	1	1	27	218	0.832
A0A0F7H5G0	Asparagine--tRNA ligase OS=Lactobacillus helveticus	2398.62	57.64	8	28	28	259	0.973
A8YVZ5	Dihydroorotase OS=Lactobacillus helveticus (strain I	2397.86	82.35	7	5	30	178	0.714
W5XKX4	Surface layer protein OS=Lactobacillus helveticus H	2390.97	36.31	1	1	17	276	1.338
A0A0D5MJ71	Cytochrome C OS=Lactobacillus helveticus GN=HUC	2382.65	62.23	8	23	23	177	0.965
F0NSW7	Pyridoxine 5'-phosphate oxidase V related favin-nuc	2361.18	77.69	2	1	7	192	1.190
A0A0D5MI18	Orotidine 5'-phosphate decarboxylase OS=Lactobac	2358.02	80.77	7	15	15	165	0.949
U6F005	Thioredoxin OS=Lactobacillus helveticus CIRM-BIA	2350.48	75.73	4	8	8	138	1.152
A0A0D5MIC5	Leucine--tRNA ligase OS=Lactobacillus helveticus GN	2325.69	68.66	7	2	53	266	0.966
U6F7W7	Bifunctional protein PyrR OS=Lactobacillus helveticu	2321.89	81.67	6	17	17	194	0.905
F3MN93	Ribonucleotide-diphosphate reductase subunit beta	2318.32	47.46	7	5	20	326	0.733
U6F614	Rod shape-determining protein MreB OS=Lactobacil	2290.71	64.67	5	2	19	150	0.988
A0A0F7H5A7	Pyridoxamine 5'-phosphate oxidase OS=Lactobacillu	2287.63	78.51	3	2	8	254	1.054
A8YX58	Bifunctional protein GlmU OS=Lactobacillus helvetic	2282.51	60.74	14	25	26	162	0.992
U6FA21	Leucine--tRNA ligase OS=Lactobacillus helveticus CI	2281.79	68.66	1	1	52	261	0.881
U6F4Q0	N5-carboxyaminoimidazole ribonucleotide synthase	2270.22	76.90	15	28	28	247	0.710
A0A0D5MHW7	X-Pro dipeptidase OS=Lactobacillus helveticus GN=I	2270.02	65.04	1	1	19	155	0.990
A0A0P0A2Y2	Lysine--tRNA ligase OS=Lactobacillus helveticus GN=	2262.40	59.69	17	36	36	285	0.910
A8YXM2	30S ribosomal protein S5 OS=Lactobacillus helveticu	2247.36	73.21	1	12	12	166	0.855
A0A0F7H4I5	Dihydroorotase OS=Lactobacillus helveticus GN=pyr	2225.42	76.94	1	1	26	165	0.616
A8YUS3	Trigger factor OS=Lactobacillus helveticus (strain DI	2213.88	43.58	16	24	24	186	0.983
F3MM03	Orotate phosphoribosyltransferase OS=Lactobacillus	2209.54	72.95	6	8	13	183	0.858
U6F790	Phosphomethylpyrimidine kinase OS=Lactobacillus h	2164.21	78.44	14	19	20	167	1.195
A8YTJ0	Alanine--tRNA ligase OS=Lactobacillus helveticus (st	2152.21	64.62	13	9	58	284	0.894
A0A0F7H4R0	Peptidase M13 OS=Lactobacillus helveticus GN=TU	2147.73	66.98	3	1	40	180	1.150
U6F929	Uncharacterized protein OS=Lactobacillus helveticus	2137.47	44.74	5	1	38	241	0.967
C4MCM7	5-methyltetrahydropteroyltriglutamate--homocystein	2134.00	60.18	12	4	44	237	1.019
A0A0F7H4J0	Aspartate--tRNA ligase OS=Lactobacillus helveticus	2114.69	68.07	2	1	41	225	0.847
A8YVH1	Probable manganese-dependent inorganic pyrophos	2106.92	76.53	6	1	23	241	0.951
F3MKV8	Aspartyl-tRNA synthetase OS=Lactobacillus helvetic	2104.06	68.07	8	1	41	224	1.045
A0A0F7H5Z5	Formate--tetrahydrofolate ligase OS=Lactobacillus h	2089.49	51.90	6	1	27	206	0.557
A8YW78	Phosphoserine aminotransferase OS=Lactobacillus h	2084.52	52.28	3	16	17	123	0.806
A0A0F7H5J7	Dihydroorotate dehydrogenase OS=Lactobacillus he	2053.43	59.28	1	1	11	138	0.776
A0A0D5MFW1	Uncharacterized protein OS=Lactobacillus helveticus	2052.75	84.33	4	7	8	122	1.234
A8YUP2	Cell division protein FtsZ OS=Lactobacillus helveticus	2036.78	68.34	10	23	23	132	1.072
U6FP45	Endopeptidase O OS=Lactobacillus helveticus CIRM-	2030.84	66.20	2	4	41	178	1.183
A0A0D5MH59	Tyrosine--tRNA ligase OS=Lactobacillus helveticus G	2022.76	59.76	9	27	27	184	1.107
U6FQ63	Fumarate hydratase OS=Lactobacillus helveticus CI	1985.01	64.64	24	1	23	182	0.798
A8YWQ7	UPF0342 protein lhv_1666 OS=Lactobacillus helvetic	1984.39	93.10	5	3	13	163	1.074
U6F6M0	Phosphoribosylamine--glycine ligase OS=Lactobacill	1978.31	53.35	10	23	23	239	0.659
J3WNH9	Rod shape-determining protein MreB OS=Lactobacil	1964.42	62.28	1	1	18	131	0.976
U6F767	Valine--tRNA ligase OS=Lactobacillus helveticus CIR	1936.56	70.42	11	56	58	289	0.960
A4ZH26	Universal stress protein OS=Lactobacillus helveticus	1934.07	87.01	6	14	14	142	1.032
A0A0D5MHF0	50S ribosomal protein L2 OS=Lactobacillus helveticu	1934.06	69.78	6	18	19	200	0.782
U6F652	Serine--tRNA ligase OS=Lactobacillus helveticus CIR	1919.66	75.63	22	30	32	195	0.964

A8YTF1	50S ribosomal protein L10 OS=Lactobacillus helvetic	1906.52	67.65	5	11	11	122	0.832
W5XGC6	Probable manganese-dependent inorganic pyrophos	1895.26	74.60	4	1	23	228	1.025
F3MM43	Putative hydrolase OS=Lactobacillus helveticus MTC	1892.37	74.88	16	21	23	215	1.003
U6F0D1	Arginine--tRNA ligase OS=Lactobacillus helveticus C	1892.07	77.54	12	43	43	197	0.851
A0A0D5MGF7	DNA-directed RNA polymerase subunit alpha OS=La	1876.87	58.33	27	21	21	236	0.948
A0A0R2MG13	Orotate phosphoribosyltransferase OS=Lactobacillus	1871.39	45.41	1	1	6	124	0.790
U4QMN2	Alanine--tRNA ligase OS=Lactobacillus helveticus CI	1868.17	56.43	11	1	50	246	0.980
A8YUL2	30S ribosomal protein S4 OS=Lactobacillus helveticu	1841.18	79.80	6	18	18	224	0.856
U6F3G7	Cell separation protein OS=Lactobacillus helveticus (	1839.75	54.31	21	15	25	218	1.489
A0A0N9ZWL9	Carboxylate--amine ligase OS=Lactobacillus helvetic	1834.09	59.48	6	33	33	220	0.942
A0A0F7H683	5-methyltetrahydropteroyltriglutamate--homocystein	1804.68	57.42	9	2	42	202	0.960
U6F6K8	D-alanine--poly(phosphoribitol) ligase subunit 1 OS=	1784.16	61.51	7	5	26	243	1.100
A8YUC9	Glycerol-3-phosphate dehydrogenase [NAD(P)+] OS	1776.31	70.50	14	22	23	158	0.972
U6F6X1	NADH peroxidase OS=Lactobacillus helveticus CIRM	1758.96	56.83	6	21	21	168	1.083
Q076V3	Endopeptidase O2 OS=Lactobacillus helveticus GN=	1755.37	62.50	2	1	38	163	1.203
A8YUW0	Thymidylate synthase OS=Lactobacillus helveticus (s	1754.38	68.24	5	22	22	184	0.955
U6F5P3	Phenylalanine--tRNA ligase beta subunit OS=Lactob	1752.38	52.11	9	39	39	172	0.896
A8YXL9	30S ribosomal protein S8 OS=Lactobacillus helveticu	1748.02	78.03	3	9	11	178	0.945
A0A0D5MHZ3	Uncharacterized protein OS=Lactobacillus helveticus	1743.17	49.19	6	25	25	145	0.983
Q2VWB2	Dipeptidase OS=Lactobacillus helveticus GN=pepD1	1740.45	57.89	6	26	26	125	1.075
A8YXM4	50S ribosomal protein L15 OS=Lactobacillus helvetic	1737.13	62.33	2	12	12	132	0.798
A0A0R2MV00	Oligopeptide ABC transporter substrate binding prot	1701.44	51.61	13	28	32	208	0.953
U6F8D8	Lipoprotein A-antigen OS=Lactobacillus helveticus C	1699.28	71.71	6	24	24	173	1.011
U6F8F6	Galactose-1-phosphate uridylyltransferase OS=Lacto	1673.50	57.70	3	2	30	172	0.904
U6F4L4	Dipeptidase OS=Lactobacillus helveticus CIRM-BIA 9	1658.48	49.89	17	21	21	165	1.010
U6F2A9	Carnosinase OS=Lactobacillus helveticus CIRM-BIA 9	1652.90	61.03	9	27	28	213	1.086
A8YVR3	Proline--tRNA ligase OS=Lactobacillus helveticus (str	1646.24	64.42	7	33	33	200	0.942
A8YTZ7	Aspartyl/glutamyl-tRNA(Asn/Gln) amidotransferase s	1645.23	65.13	5	32	32	265	0.937
A4ZH47	Aspartate/tyrosine/aromatic aminotransferase OS=L	1644.24	54.12	27	21	22	172	0.944
F3MR57	Glycerol-3-phosphate ABC transporter OS=Lactobac	1644.11	65.34	10	26	27	169	0.986
U6F4A0	Pur operon repressor PurR OS=Lactobacillus helveti	1625.31	70.65	5	18	19	133	0.949
F3MLX5	Bifunctional 5,10-methylene-tetrahydrofolate dehydr	1619.76	86.09	2	1	9	96	1.052
U6F734	DNA gyrase subunit A OS=Lactobacillus helveticus C	1612.13	46.92	11	39	41	139	1.107
A0A0D5MHG1	tRNA-binding protein OS=Lactobacillus helveticus GI	1611.94	71.16	5	16	16	146	1.055
U6F3R7	Cyclopropane-fatty-acyl-phospholipid synthase OS=l	1605.22	47.84	10	19	19	135	0.904
A0A0D5MKB4	UDP-N-acetylmuramyl-tripeptide synthetase OS=Lac	1589.17	69.60	7	30	30	158	0.944
U6F132	Uncharacterized protein OS=Lactobacillus helveticus	1565.69	57.98	2	6	23	297	1.467
F3MKX6	2',3'-cyclic-nucleotide 2'-phosphodiesterase OS=Lact	1564.68	63.08	9	3	35	198	0.962
A0A0D5MH97	CTP synthase OS=Lactobacillus helveticus GN=pyrG	1564.38	54.55	9	26	26	153	0.878
A8YV20	2',3'-cyclic-nucleotide 2'-phosphodiesterase OS=Lact	1557.58	67.88	1	2	34	192	0.963
A4ZH14	Phosphoribosylformylglycinamidine synthase subunit	1555.38	49.33	4	10	11	112	0.737
A0A0D5MI61	Amidophosphoribosyltransferase OS=Lactobacillus h	1553.45	48.22	6	22	22	131	0.717
U6F5U4	Pyridine nucleotide-disulfide oxidoreductase OS=Lac	1541.38	41.87	2	1	15	112	1.128
V5Z1S2	Xaa-Pro dipeptidyl-peptidase OS=Lactobacillus helve	1533.93	34.55	5	2	27	192	1.072
F3MML1	UPF0342 protein AAULH_09583 OS=Lactobacillus he	1529.37	86.21	2	1	11	125	0.934
A0A0F7H4D9	Uridylate kinase OS=Lactobacillus helveticus GN=py	1526.43	69.71	4	13	13	95	1.011
U6EZK9	N-acetylglucosamine kinase OS=Lactobacillus helvet	1511.36	64.50	8	18	18	159	1.018
U6F430	Catabolite control protein A OS=Lactobacillus helvet	1502.38	84.98	8	26	26	118	0.966
U6F4D5	Acetate kinase OS=Lactobacillus helveticus CIRM-BI	1497.65	66.50	19	19	20	135	1.085
F0NWV8	Galactose-1-phosphate uridylyltransferase OS=Lacto	1494.30	56.26	5	1	29	157	0.937
A0A0D5MJ45	GTP-binding protein TypA OS=Lactobacillus helvetic	1484.87	47.72	5	27	27	135	0.873
A0A0D5MGV1	UDP-N-acetylmuramoyl-tripeptide--D-alanyl-D-alanir	1483.23	56.48	4	24	24	144	1.003
A0A0P0A4S7	ATP-dependent protease ATPase subunit HslU OS=L	1468.34	56.96	5	29	29	153	0.972
A0A0N9Z9Z7	50S ribosomal protein L13 OS=Lactobacillus helvetic	1464.83	78.23	9	12	12	152	0.740
A0A0D5MKA1	Phosphocarrier protein HPr OS=Lactobacillus helveti	1464.45	59.09	3	4	5	126	1.086

A8YVR0	Transcription termination/antitermination protein Nu	1442.94	65.90	12	23	23	123	0.889
U6FD93	Uncharacterized protein OS=Lactobacillus helveticus	1438.48	64.22	9	13	13	102	1.084
A8YW83	Phosphoribosylformylglycinamidine cyclo-ligase OS=	1438.45	60.74	1	17	17	103	0.619
A0A0D5MJT1	NADPH-dependent FMN reductase OS=Lactobacillus	1436.75	78.02	5	11	11	126	0.997
A8YTZ3	DNA ligase OS=Lactobacillus helveticus (strain DPC	1431.11	63.32	8	36	37	141	1.053
A8YXQ1	S-ribosylhomocysteine lyase OS=Lactobacillus helve	1425.37	71.97	6	15	15	151	1.073
A8YWP6	D-alanine--D-alanine ligase OS=Lactobacillus helveti	1413.88	67.50	10	22	22	157	0.941
A8YUF8	Phosphoglucosamine mutase OS=Lactobacillus helve	1385.41	49.33	9	18	19	127	0.972
U6F5W1	Bifunctional protein PyrR OS=Lactobacillus helveticu	1376.95	82.39	9	15	15	131	0.922
A0A0D5MKX9	NH(3)-dependent NAD(+) synthetase OS=Lactobaci	1365.31	79.71	6	18	18	153	1.086
A8YXM0	50S ribosomal protein L6 OS=Lactobacillus helveticu	1361.01	75.57	3	13	13	133	0.889
A0A0R2MQS8	50S ribosomal protein L11 OS=Lactobacillus helvetic	1358.46	45.39	4	8	8	105	0.823
A0A0D5MFE1	Pyridine nucleotide-disulfide oxidoreductase OS=Lac	1344.18	42.54	5	1	15	101	0.966
A0A0F7H270	Asparagine synthase OS=Lactobacillus helveticus GN	1333.86	48.38	8	35	36	179	0.907
A0A0D5MHF6	Peptide ABC transporter ATPase OS=Lactobacillus h	1325.17	50.58	6	14	14	109	1.018
A4ZH31	ATP-dependent Clp protease proteolytic subunit OS=	1312.59	45.88	4	7	7	83	1.047
U6F648	Succinic semialdehyde dehydrogenase OS=Lactobac	1311.07	59.35	12	23	24	111	1.048
U6F2M4	Ribose-phosphate pyrophosphokinase OS=Lactobac	1290.82	60.37	9	16	17	143	0.945
A0A0D5MGZ6	Uncharacterized protein OS=Lactobacillus helveticus	1279.58	61.49	15	1	9	81	0.989
A0A0F7H1V3	DNA polymerase III subunit beta OS=Lactobacillus l	1261.72	62.50	7	19	19	106	1.000
A0A0D5MK75	Protein translocase subunit SecA OS=Lactobacillus h	1254.28	61.95	7	49	50	171	0.959
A4UAE6	Peptidase M42 family OS=Lactobacillus helveticus C	1253.01	68.33	9	19	19	122	0.974
U6F3V9	Uracil phosphoribosyltransferase OS=Lactobacillus h	1246.37	69.86	5	2	13	93	0.856
U6F3U1	Uncharacterized protein OS=Lactobacillus helveticus	1241.83	53.50	15	34	35	93	0.951
U6F5C9	Ribonuclease J 1 OS=Lactobacillus helveticus CIRM-	1239.66	45.44	11	20	20	74	0.901
A8YVP7	Adenine phosphoribosyltransferase OS=Lactobacillu	1215.16	76.00	5	14	14	134	0.992
U6F124	S-adenosylmethionine synthase OS=Lactobacillus he	1205.69	58.15	6	25	25	128	1.042
A0A0N9ZBV3	D-alanine--poly(phosphoribitol) ligase subunit 1 OS=	1204.48	46.83	1	1	22	199	1.210
U6F3B5	Dihydropteroate synthase OS=Lactobacillus helvetic	1178.34	57.02	4	17	17	94	1.009
U6F2K7	Hydrolase of alpha-beta family OS=Lactobacillus hel	1178.20	89.64	12	20	20	125	1.261
A0A0F7H6A8	Thioredoxin OS=Lactobacillus helveticus GN=ALV80	1177.02	80.19	5	10	10	121	1.203
F3MMQ9	Thioredoxin reductase OS=Lactobacillus helveticus M	1176.93	74.27	4	1	16	101	1.078
A8YV35	GTPase Der OS=Lactobacillus helveticus (strain DPC	1169.10	54.02	7	24	24	97	0.853
A4UAE0	Foldase protein PrsA OS=Lactobacillus helveticus CN	1166.65	59.33	9	21	22	137	1.088
A8YXH9	50S ribosomal protein L31 type B OS=Lactobacillus	1164.63	80.25	3	5	5	89	0.929
F3MMX4	Uncharacterized protein OS=Lactobacillus helveticus	1145.65	59.70	6	22	22	119	0.867
A8YTH7	10 kDa chaperonin OS=Lactobacillus helveticus (stra	1123.94	95.74	1	11	11	190	0.979
A4ZH41	Serine protease OS=Lactobacillus helveticus CNRZ32	1120.97	46.00	7	14	14	87	1.148
F3MMB2	Uncharacterized protein OS=Lactobacillus helveticus	1112.65	47.89	18	17	18	110	1.081
A8YV01	Aromatic amino acid aminotransferase OS=Lactobac	1108.82	52.79	9	5	22	145	0.964
A0A0D5MGP8	Universal stress protein UspA OS=Lactobacillus helv	1098.47	53.37	2	2	7	47	1.067
U6FMV7	Oligopeptide ABC superfamily ATP binding cassette	1078.34	62.46	5	21	22	127	1.011
U6F4W3	Uncharacterized protein OS=Lactobacillus helveticus	1077.40	68.57	8	21	21	99	0.925
A0A0P0A607	Acetyl-CoA carboxylase OS=Lactobacillus helveticus	1076.93	56.21	3	3	20	117	0.924
F0NWH0	50S ribosomal protein L20 OS=Lactobacillus helvetic	1071.93	70.30	2	10	10	147	0.776
U6F6G4	Citrate lyase OS=Lactobacillus helveticus CIRM-BIA	1063.13	71.05	7	21	21	179	0.948
A8YW47	30S ribosomal protein S6 OS=Lactobacillus helveticu	1044.84	82.65	2	9	9	125	0.986
U6F2Y4	L-2-hydroxyisocaproate dehydrogenase OS=Lactoba	1042.88	48.36	5	18	18	125	0.989
A8YUZ9	Histidine--tRNA ligase OS=Lactobacillus helveticus (	1042.60	57.48	10	22	22	103	0.930
U6F0M5	Enoyl-[acyl-carrier-protein] reductase [NADH] OS=L	1037.31	62.30	5	18	18	133	1.004
A8YVT1	50S ribosomal protein L19 OS=Lactobacillus helvetic	1035.92	73.04	3	12	12	103	0.799
A0A0D5MJC6	GTPase Obg OS=Lactobacillus helveticus GN=obgE	1033.36	55.76	5	22	22	85	0.923
J3WLQ1	Bifunctional purine biosynthesis protein PurH OS=La	1029.40	13.45	3	1	8	144	0.836
A0A0D5ML22	Sugar ABC transporter substrate-binding protein OS	1012.16	67.44	11	22	28	113	1.038
F3MNE2	Uncharacterized protein OS=Lactobacillus helveticus	1001.88	45.40	3	4	17	99	1.011

U6F3S4	Alpha-glucosidase OS=Lactobacillus helveticus CIRM	983.24	45.27	10	38	38	130	1.075
A0A0F7H284	Cell separation protein OS=Lactobacillus helveticus (	980.57	38.71	2	1	17	229	1.232
A8YXK7	50S ribosomal protein L23 OS=Lactobacillus helvetic	979.88	72.28	1	2	11	122	0.687
A8YUS4	ATP-dependent Clp protease ATP-binding subunit Cl	977.31	60.38	12	29	29	107	0.872
A0A0D5MH36	Phosphoribosylformylglycinamidine synthase subunit	966.38	72.29	4	7	7	59	0.799
A0A0D5MIS8	ATP synthase subunit delta OS=Lactobacillus helvet	964.77	55.49	7	9	9	60	0.957
U6F0S6	Uncharacterized protein OS=Lactobacillus helveticus	959.50	79.17	4	8	8	122	0.828
A8YWG1	UDP-N-acetylmuramate--L-alanine ligase OS=Lactob	958.03	55.61	12	3	23	141	0.918
A0A0R2MNX8	Peptide deformylase OS=Lactobacillus helveticus GN	956.76	81.22	5	12	12	79	1.088
A0A0P0A715	Tagatose-6-phosphate kinase OS=Lactobacillus helv	954.46	51.64	9	3	12	68	0.996
A8YXJ7	ATP-dependent Clp protease OS=Lactobacillus helve	951.16	50.00	9	41	41	105	0.817
A8YXL1	30S ribosomal protein S3 OS=Lactobacillus helveticu	947.19	58.48	5	18	18	146	0.788
A0A0D5MHT2	Tryptophan--tRNA ligase OS=Lactobacillus helveticu	946.57	56.47	6	17	17	83	0.974
A8YUJ7	ATP synthase subunit b OS=Lactobacillus helveticus	946.24	52.07	31	1	10	51	1.000
A0A0D5MI19	30S ribosomal protein S11 OS=Lactobacillus helvetic	945.62	33.33	3	4	4	81	0.774
A8YUK0	ATP synthase gamma chain OS=Lactobacillus helvet	941.56	67.81	3	1	19	107	1.237
A0A0D5MHM3	UDP-glucose 4-epimerase OS=Lactobacillus helvetic	940.81	58.18	3	16	16	101	0.918
U6F331	DEAD/DEAH box family ATP-dependent RNA helicas	940.35	49.30	8	22	22	68	0.722
A0A0N7HHW9	PhoB family transcriptional regulator OS=Lactobacill	935.21	49.12	4	11	11	59	1.028
J4BQG0	Glycine--tRNA ligase alpha subunit OS=Lactobacillus	934.73	58.28	5	17	17	94	0.902
A0A0F7D039	UDP-N-acetylmuramoylalanine--D-glutamate ligase (	929.37	57.30	12	26	27	89	1.004
U6F387	Phosphoglycerate mutase OS=Lactobacillus helvetic	924.17	67.71	4	15	15	98	0.980
A0A0R2MUT6	30S ribosomal protein S16 OS=Lactobacillus helvetic	922.51	64.29	2	6	6	88	0.768
A0A0P0A4P8	SAM-dependent methyltransferase OS=Lactobacillus	908.58	52.14	9	11	11	56	0.924
U6F6T1	Transcription elongation factor GreA OS=Lactobacill	906.91	64.56	4	8	8	100	1.085
A0A0R2MPT3	ATP synthase gamma chain OS=Lactobacillus helvet	903.69	67.81	2	1	19	104	1.156
A0A0F7H3B0	N-deoxyribosyltransferase OS=Lactobacillus helvetic	895.78	74.05	5	1	10	117	0.960
U6F035	FolKE OS=Lactobacillus helveticus CIRM-BIA 951 GN	893.04	67.98	3	2	22	82	1.012
Q8RLY4	N-deoxyribosyltransferase OS=Lactobacillus helvetic	883.94	74.05	5	1	10	113	0.905
W5XHD3	UDP-N-acetylmuramate--L-alanine ligase OS=Lactob	883.74	53.57	7	1	21	130	1.141
F3MMW5	Uncharacterized protein OS=Lactobacillus helveticus	876.26	61.49	10	1	9	59	1.337
U6F8A7	Aldehyde-alcohol dehydrogenase OS=Lactobacillus h	868.11	43.71	12	36	36	88	0.872
U6F0C8	3-carboxymuconate cyclase OS=Lactobacillus helvet	856.25	66.67	7	18	18	88	1.055
U6F5Y3	Carbamoyl-phosphate synthase small chain OS=Lac	854.07	39.61	7	14	14	105	0.766
A0A0D5MGE2	50S ribosomal protein L14 OS=Lactobacillus helvetic	851.34	81.15	3	15	15	105	0.761
A0A0N9ZI12	Peptidase T OS=Lactobacillus helveticus GN=ALV80	846.49	41.89	9	13	14	75	1.022
J7LFF8	Ribonucleoside-diphosphate reductase 2 alpha subu	845.54	38.81	4	1	16	173	0.956
A0A0F7H3S0	GTP cyclohydrolase OS=Lactobacillus helveticus GN=	844.48	65.27	5	1	21	78	1.416
U6F002	2,5-didehydrogluconate reductase OS=Lactobacillus	843.53	71.23	7	15	15	65	1.152
J3WL14	50S ribosomal protein L23 OS=Lactobacillus helvetic	842.37	72.28	2	1	10	104	0.855
A0A0R2MPS8	Uracil phosphoribosyltransferase OS=Lactobacillus h	841.35	57.42	2	1	12	74	1.088
A0A0D5MHL9	Uncharacterized protein OS=Lactobacillus helveticus	828.45	64.58	9	8	8	116	0.913
U6F3D8	ABC transporter ATP-binding protein OS=Lactobacill	821.40	52.96	18	3	29	91	0.919
A0A0N9ZGI3	Probable DNA-directed RNA polymerase subunit delt	819.30	32.61	6	6	6	57	1.034
F3MNN5	Cadmium efflux ATPase OS=Lactobacillus helveticus	818.37	45.04	10	4	22	102	0.888
A0A0F7H3C0	Probable tRNA sulfurtransferase OS=Lactobacillus h	817.02	63.46	16	2	22	105	0.845
U6F5X5	Formate--tetrahydrofolate ligase OS=Lactobacillus h	816.61	46.95	9	26	27	134	1.000
U6F2C3	Phosphoglycerate dehydrogenase OS=Lactobacillus	808.53	45.39	3	16	16	108	0.822
A0A0F7H5T6	Ribosomal RNA small subunit methyltransferase H C	807.47	62.22	7	1	13	34	0.872
A0A0N9ZAQ5	Maltose epimerase OS=Lactobacillus helveticus GN=	806.97	55.59	13	13	13	64	0.987
A8YTD3	Cysteine--tRNA ligase OS=Lactobacillus helveticus (s	806.94	36.13	11	16	16	63	0.972
U6F4C9	DNA topoisomerase 1 OS=Lactobacillus helveticus C	800.93	49.01	8	34	34	103	1.290
A8YUH0	Probable transcriptional regulatory protein lhv_0777	799.03	49.17	3	2	12	110	0.994
U4QNU4	Ribosomal RNA small subunit methyltransferase H C	792.11	62.22	2	1	13	34	0.869
U6F1J2	Heat shock protein HSP OS=Lactobacillus helveticus	787.03	84.46	6	9	12	104	1.045

U6F3U7	Rod shape-determining protein Mbl OS=Lactobacillu	785.18	59.27	4	16	16	78	0.910
U6EZF6	23S rRNA (Uracil-5-)-methyltransferase RumA OS=L	778.32	56.89	8	3	23	86	1.061
A8YXL0	50S ribosomal protein L22 OS=Lactobacillus helvetic	772.22	76.07	3	8	8	92	0.755
A8YVZ6	Aspartate carbamoyltransferase OS=Lactobacillus he	771.91	60.06	11	20	20	120	0.830
A0A0D5MGV9	Pyrrolidone-carboxylate peptidase OS=Lactobacillus	771.57	55.00	10	10	10	48	1.074
A0A0F7D0L2	Ribosome-binding ATPase YchF OS=Lactobacillus he	771.20	49.73	5	20	20	98	1.002
A8YUT6	Aspartate-semialdehyde dehydrogenase OS=Lactoba	767.90	65.34	7	20	20	107	1.032
A8YU90	Peptide chain release factor 3 OS=Lactobacillus helv	765.49	41.49	4	19	19	79	0.898
A8YXK9	30S ribosomal protein S19 OS=Lactobacillus helvetic	761.04	54.84	3	7	7	104	0.900
A8YXD6	Chromosome partitioning protein OS=Lactobacillus l	759.32	58.06	6	16	16	55	0.990
Q48570	Proline iminopeptidase OS=Lactobacillus helveticus l	757.09	58.55	3	17	17	76	0.997
A0A0D5MK99	DNA helicase OS=Lactobacillus helveticus GN=HUO_	754.63	58.16	8	42	42	111	0.977
A0A0F7H556	Thioredoxin reductase OS=Lactobacillus helveticus (	749.32	68.40	2	1	15	81	1.064
A0A0D5MKC7	NADPH-dependent FMN reductase OS=Lactobacillus	746.06	46.37	10	6	7	63	0.957
U6F572	LytA OS=Lactobacillus helveticus CIRM-BIA 951 GN=	745.75	42.26	10	5	15	47	1.208
Q2VWB3	Peptidase OS=Lactobacillus helveticus GN=pepCE P	742.72	51.26	1	18	21	81	0.973
F0NX54	Protein NrdI OS=Lactobacillus helveticus (strain H10	740.00	70.21	3	1	9	78	0.733
J4BUX5	Aspartate transaminase OS=Lactobacillus helveticus	732.35	42.43	2	1	18	93	1.014
A0A0N7HHT5	Methionine aminopeptidase OS=Lactobacillus helvet	731.58	63.27	5	12	12	70	0.970
A0A0N9ZAX9	Protein NrdI OS=Lactobacillus helveticus GN=nrdI P	729.44	69.29	4	1	9	71	0.616
U6F4Y4	DNA helicase OS=Lactobacillus helveticus CIRM-BIA	721.14	46.35	13	32	33	97	0.887
U6F603	Alkaline shock protein OS=Lactobacillus helveticus C	715.82	57.45	6	7	7	59	0.995
A8YUJ1	Peptide chain release factor 1 OS=Lactobacillus helv	704.09	50.55	2	19	19	107	0.975
A0A0D5MIC2	Signal recognition particle protein OS=Lactobacillus	701.30	48.53	8	24	24	75	0.866
U6F355	UDP-N-acetylglucosamine 2-epimerase OS=Lactobac	698.73	60.53	10	18	18	89	1.064
A0A0N9ZAH0	Threonylcarbamoyl-AMP synthase OS=Lactobacillus	696.07	65.17	10	17	17	59	0.991
U6F298	Phosphonate ABC superfamily ATP binding cassette	692.48	50.81	9	3	13	59	0.910
A0A0P0A221	NAD-dependent dehydratase OS=Lactobacillus helve	692.23	66.51	9	4	18	56	1.027
F3MNB9	Biotin carboxylase OS=Lactobacillus helveticus MTC(	690.05	56.65	1	5	21	85	0.918
A0A0D5MI21	rRNA methyltransferase OS=Lactobacillus helveticus	688.38	71.76	7	15	15	95	0.983
A8YVX7	50S ribosomal protein L21 OS=Lactobacillus helvetic	685.09	55.34	2	6	6	56	0.827
U6F4Q5	Uncharacterized protein OS=Lactobacillus helveticus	683.47	56.75	13	32	32	114	1.077
A0A0D5MKA7	Probable tRNA sulfurtransferase OS=Lactobacillus h	676.38	59.75	9	1	21	91	0.821
A8YU37	UPF0210 protein lhv_0606 OS=Lactobacillus helvetic	676.12	44.30	6	16	16	76	1.048
U6F4I7	Uncharacterized protein OS=Lactobacillus helveticus	673.50	59.81	5	2	12	55	1.013
U6F2X1	Cysteine synthase OS=Lactobacillus helveticus CIRM	672.02	50.16	11	11	11	57	1.183
U6F4I0	Phosphotransacetylase OS=Lactobacillus helveticus	671.50	73.56	7	22	22	105	1.045
A8YTU2	Mannose-specific PTS system component IIAB OS=l	669.97	56.85	9	19	19	78	0.945
A0A0D5MIS1	Aluminum resistance protein OS=Lactobacillus helve	663.76	43.44	6	18	18	80	0.934
A8YXK4	30S ribosomal protein S10 OS=Lactobacillus helvetic	661.81	82.35	1	10	10	103	0.847
A8YX12	Surface layer protein OS=Lactobacillus helveticus (s	654.96	11.16	4	1	8	112	0.775
A8YUC7	HPr kinase/phosphorylase OS=Lactobacillus helvetic	653.26	73.91	4	22	22	71	1.040
J4BUB9	Phosphate starvation-inducible protein PhoH OS=La	649.85	51.10	34	16	16	59	1.027
A0A0N9ZWW4	ATP-dependent zinc metalloprotease FtsH OS=Lacto	644.39	40.36	18	27	28	87	0.984
F3MR64	Ribokinase OS=Lactobacillus helveticus MTCC 5463	643.97	67.86	9	17	17	59	1.054
U6FAC3	P-ATPase superfamily P-type ATPase cadmium trans	642.75	34.69	2	1	19	79	1.192
J7LHP1	Universal stress protein UspA OS=Lactobacillus helv	641.39	44.79	1	1	6	37	1.094
J7LM78	NAD-dependent epimerase/dehydratase OS=Lactob	629.83	62.74	4	1	15	52	0.907
A0A0F7H2S9	RNA methyltransferase OS=Lactobacillus helveticus	628.03	52.44	1	1	21	72	1.089
A0A0D5MI17	PhoB family transcriptional regulator OS=Lactobacill	626.54	43.70	5	11	11	70	1.083
A0A0R2MJR7	Tagatose-6-phosphate kinase OS=Lactobacillus helv	624.93	53.62	2	1	10	50	0.909
A8YXL2	50S ribosomal protein L16 OS=Lactobacillus helvetic	623.62	21.23	1	3	3	53	0.806
A0A0D5MGU5	Acetyl-CoA carboxylase OS=Lactobacillus helveticus	622.09	88.46	5	13	13	75	0.968
A0A0D5MH73	30S ribosomal protein S18 OS=Lactobacillus helvetic	617.45	63.16	3	8	8	73	0.922
U6F435	Ribonuclease R OS=Lactobacillus helveticus CIRM-B	605.61	37.61	21	28	28	79	0.910

A8YXN1	50S ribosomal protein L17 OS=Lactobacillus helvetic	603.30	57.48	3	9	9	95	0.723
U6F1X2	Glucose-6-phosphate 1-dehydrogenase OS=Lactoba	602.61	44.31	9	20	20	57	1.013
U6F5T3	4-methyl-5(B-hydroxyethyl)-thiazole monophosphate	595.19	70.10	7	13	13	76	1.048
A0A0D5MG96	Acyl carrier protein OS=Lactobacillus helveticus GN=	591.01	36.25	2	6	6	61	0.940
U6F5L6	Hydrolase of the HAD family OS=Lactobacillus helve	589.98	42.80	8	10	10	36	1.007
A8YXM6	Adenylate kinase OS=Lactobacillus helveticus (strair	584.77	72.94	4	16	16	75	0.968
A0A0D5MI71	Trp operon repressor OS=Lactobacillus helveticus G	580.97	67.11	5	7	7	37	1.177
A0A0D5MGZ3	50S ribosomal protein L18 OS=Lactobacillus helvetic	580.82	48.74	4	8	8	117	0.969
A0A0D5MG56	tRNA uridine 5-carboxymethylaminomethyl modifica	580.06	45.08	10	28	28	78	0.888
A0A0D5MJ54	5'-methylthioadenosine/S-adenosylhomocysteine nu	573.52	34.20	8	7	7	32	1.085
U6F1X8	DNA mismatch repair protein MutS OS=Lactobacillus	572.58	41.73	11	30	30	58	0.931
A8YTJ4	Endonuclease MutS2 OS=Lactobacillus helveticus (st	572.25	38.73	34	29	30	68	0.895
U6F552	Ribonuclease H1 OS=Lactobacillus helveticus CIRM-	571.53	55.95	8	15	15	56	0.976
A0A0D5MJL5	Elongation factor P OS=Lactobacillus helveticus GN=	569.81	52.63	7	10	10	79	1.056
A0A0F7H643	Galactokinase OS=Lactobacillus helveticus GN=galK	568.73	49.48	6	2	19	64	0.936
A0A0N9ZQM7	Serine/threonine protein phosphatase OS=Lactobac	568.72	43.01	10	19	19	73	0.941
A0A0F7H4C5	UTP--glucose-1-phosphate uridylyltransferase OS=L	565.16	59.53	6	12	16	71	1.089
A0A0D5MLC2	Uncharacterized protein OS=Lactobacillus helveticus	563.60	51.87	8	1	11	49	1.010
A0A0D5MH72	30S ribosomal protein S9 OS=Lactobacillus helveticu	562.92	45.04	2	7	7	89	0.822
J3XAU1	ATPase component of ABC transporter with duplicat	562.03	50.37	10	1	27	80	0.945
A0A0F7H344	UDP-N-acetylglucosamine 1-carboxyvinyltransferase	557.18	49.65	10	19	19	60	1.009
A0A0F7D074	Peptide-binding protein OS=Lactobacillus helveticus	555.64	41.69	20	1	14	57	1.133
A0A0D5MJ13	Acyl carrier protein OS=Lactobacillus helveticus GN=	554.55	40.00	3	3	3	44	1.148
A0A0R2MQZ4	N-acetylmuramidase OS=Lactobacillus helveticus GN	544.39	32.97	2	1	11	33	1.299
A0A0N9ZU33	Metallo-beta-lactamase OS=Lactobacillus helveticus	535.93	48.26	8	19	19	67	1.047
F3MPJ2	Alanine racemase OS=Lactobacillus helveticus MTC(	534.84	42.55	8	15	16	83	0.962
Q2VWB1	Cytochrome C oxidase subunit II OS=Lactobacillus h	527.00	43.83	7	18	18	53	0.998
A0A0D5MI48	Transcription termination/antitermination protein Nu	525.01	72.97	6	11	11	50	0.945
A8YXK1	30S ribosomal protein S12 OS=Lactobacillus helvetic	521.45	65.19	3	9	9	92	0.865
U6F9C0	Uncharacterized protein OS=Lactobacillus helveticus	516.37	35.48	2	1	8	34	0.928
A0A0D5MJR3	Uncharacterized protein OS=Lactobacillus helveticus	513.90	92.47	4	7	7	39	1.317
A0A0D5MKM4	Ribosomal subunit interface protein OS=Lactobacillu	513.59	39.23	6	7	7	52	0.950
U6F4T7	3-oxoacyl-[acyl-carrier protein] reductase OS=Lacto	509.42	48.97	5	9	9	44	1.001
U4QFH7	N-acetylglucosamine-6-phosphate deacetylase OS=I	508.33	56.25	10	3	17	57	1.174
A0A0N7HHT9	Ribose-5-phosphate isomerase A OS=Lactobacillus h	503.74	46.09	6	10	10	36	0.941
A0A0D5MGU3	DegV family protein OS=Lactobacillus helveticus GN	501.16	35.48	7	1	8	33	0.897
U6F252	[Citrate [pro-3S]-lyase] ligase OS=Lactobacillus helv	500.36	66.29	10	18	18	52	0.927
Q8RLY5	Nucleoside 2-deoxyribosyltransferase OS=Lactobacil	499.47	61.08	6	8	8	58	1.073
A8YV64	Ornithine decarboxylase OS=Lactobacillus helveticus	498.74	37.97	13	24	26	67	0.853
A0A0F7H621	GTPase HflX OS=Lactobacillus helveticus GN=hflX P	498.60	42.99	20	1	18	49	0.984
A0A0D5MK57	Uncharacterized protein OS=Lactobacillus helveticus	489.07	64.60	2	7	7	39	0.976
A0A0D5MJT6	Peptide-binding protein OS=Lactobacillus helveticus	488.67	41.69	13	1	14	47	1.287
A8YUQ8	UPF0356 protein lhv_0877 OS=Lactobacillus helvetic	488.25	82.19	2	6	6	32	1.084
F3MR68	Nucleoside hydrolase OS=Lactobacillus helveticus M	485.46	55.07	6	12	12	68	1.014
A0A0N9Z5H6	DNA gyrase subunit B OS=Lactobacillus helveticus G	480.16	50.31	11	31	32	96	0.911
A8YTF9	Thymidylate kinase OS=Lactobacillus helveticus (stra	478.81	55.66	5	14	14	53	1.056
F0NU06	Metallo-beta-lactamase superfamily protein OS=Lac	478.13	43.42	13	26	26	84	0.873
A0A0F7H384	4-hydroxy-tetrahydrodipicolinate synthase OS=Lacto	477.67	58.20	8	15	15	36	1.122
U6F697	GMP reductase OS=Lactobacillus helveticus CIRM-BI	471.92	46.06	8	15	15	48	0.626
A0A0R2MMP7	Phenylalanine--tRNA ligase alpha subunit OS=Lactol	471.49	52.44	90	16	23	81	0.913
U6F3N5	DEAD/DEAH box family ATP-dependent RNA helicas	470.71	47.68	9	20	20	61	0.912
A8YXL3	50S ribosomal protein L29 OS=Lactobacillus helvetic	470.26	60.00	2	5	5	50	0.718
Q00052	Galactokinase OS=Lactobacillus helveticus GN=galK	461.68	49.48	5	1	18	59	0.913
A0A0F7H6I9	3-hydroxyacyl-ACP dehydratase OS=Lactobacillus he	460.45	65.94	6	9	9	46	1.181
U6F3S3	Malonyl CoA-acyl carrier protein transacylase OS=La	460.18	43.93	5	10	10	41	0.930

A4UAE4	GTPase HflX OS=Lactobacillus helveticus CNRZ32 G	458.39	39.90	17	1	18	48	0.873
W5XEB0	Probable transcriptional regulatory protein LBH_062	456.21	44.21	1	1	11	77	1.076
A0A0F7H2T3	Phosphate ABC transporter substrate-binding protei	455.44	44.34	3	1	11	46	0.896
U6F483	Fibronectin domain-containing protein OS=Lactobac	455.37	60.99	12	5	29	79	0.954
A8YXL4	30S ribosomal protein S17 OS=Lactobacillus helvetic	451.94	46.59	3	6	6	73	0.789
A0A0P0A5G4	Aldose epimerase OS=Lactobacillus helveticus GN=A	451.21	69.23	9	19	19	59	1.010
A0A0F7H5A9	Adenosine deaminase OS=Lactobacillus helveticus G	448.48	47.45	9	14	14	41	1.024
F3MLE8	Amino acid ABC transporter substrate-binding prote	447.69	59.44	5	17	17	44	1.141
F3MQK3	Non-canonical purine NTP pyrophosphatase OS=Lac	447.52	64.08	6	12	12	43	1.056
A0A0F7H2J8	Ribosomal RNA small subunit methyltransferase I O	446.22	52.11	8	14	15	41	0.947
U6F3I9	DNA polymerase OS=Lactobacillus helveticus CIRM-	442.48	41.26	14	36	36	70	0.985
A0A0D5MJN6	ADP-ribose pyrophosphatase OS=Lactobacillus helve	439.63	54.50	5	8	8	30	0.992
F3MMV9	Protein tyrosine phosphatase OS=Lactobacillus helv	437.39	65.54	8	16	16	64	0.929
A0A0D5MJT3	Hypoxanthine phosphoribosyltransferase OS=Lactob	431.23	65.08	8	11	11	42	1.081
A0A0D5MGV2	3'-5' exonuclease OS=Lactobacillus helveticus GN=H	430.68	32.31	5	10	10	29	1.032
A0A0N7HHU8	NUDIX hydrolase OS=Lactobacillus helveticus GN=A	430.39	70.80	2	15	15	131	1.093
A0A0N7HHX6	3-oxoacyl-[acyl-carrier-protein] synthase 3 OS=Lact	429.39	36.39	8	10	10	23	0.925
A8YTY3	Nicotinate phosphoribosyltransferase OS=Lactobacil	427.61	45.33	9	4	21	52	0.916
U6F8E5	5-nucleotidase OS=Lactobacillus helveticus CIRM-BI	426.67	46.31	14	25	25	58	0.877
A8YUF1	UDP-N-acetylenolpyruvoylglucosamine reductase OS	424.17	52.68	6	16	17	59	1.085
A0A0N7HHQ6	Isochorismatase OS=Lactobacillus helveticus GN=Al	423.89	55.43	4	10	10	47	1.122
J4BU41	Peptide methionine sulfoxide reductase MsrB OS=La	423.70	57.86	5	9	9	37	1.209
U6F702	Ornithine decarboxylase OS=Lactobacillus helveticus	420.83	38.66	13	29	30	79	0.952
A8YW53	50S ribosomal protein L9 OS=Lactobacillus helveticu	413.90	67.55	4	15	15	71	0.897
U6F4E9	Guanylate kinase OS=Lactobacillus helveticus CIRM-	408.52	53.43	6	11	11	46	1.006
A8YUU5	Nicotinate phosphoribosyltransferase OS=Lactobacil	407.19	51.15	6	4	23	60	1.028
A0A0N7HHK4	50S ribosomal protein L27 OS=Lactobacillus helvetic	406.68	47.92	4	4	4	36	0.746
A8YXM7	Translation initiation factor IF-1 OS=Lactobacillus he	403.09	68.49	2	4	4	30	1.014
U6F5B1	Cell division initiation protein OS=Lactobacillus helve	402.01	32.55	6	1	8	27	1.066
A0A0D5MKA8	Acetylglucosaminyldiphospho-UDP acetyl-beta-D-ma	401.93	47.52	9	11	11	29	0.872
F3MQY4	Uncharacterized protein OS=Lactobacillus helveticus	397.79	73.68	1	4	4	50	0.961
U6F2N0	Uncharacterized protein OS=Lactobacillus helveticus	396.62	57.62	6	5	5	39	1.007
A0A0D5MIF7	Elongation factor 4 OS=Lactobacillus helveticus GN=	394.94	44.44	12	24	24	67	0.958
A0A0D5MIG9	Diadenosine tetraphosphate hydrolase OS=Lactobac	394.05	55.86	7	8	8	56	1.149
A0A0D5MHD0	Exodeoxyribonuclease OS=Lactobacillus helveticus G	393.16	57.09	5	15	15	49	1.109
U6F5X9	N6-adenine-specific DNA methylase OS=Lactobacillu	392.60	50.80	9	5	15	53	1.002
U6F515	Possible site-specific DNA-methyltransferase (Adenir	391.71	38.66	3	18	18	42	1.016
U6F058	DNA polymerase III PolC-type OS=Lactobacillus helv	391.00	26.43	11	32	33	47	0.968
A0A0F7H5D9	DNA-directed RNA polymerase subunit omega OS=L	386.75	58.11	5	5	5	40	0.910
A0A0D5MI09	Translation initiation factor IF-3 OS=Lactobacillus he	385.59	35.85	8	7	7	44	0.839
A0A0D5MHN2	Cell surface protein OS=Lactobacillus helveticus GN=	381.95	38.15	4	13	13	60	1.244
A0A0D5MHT5	5-formyltetrahydrofolate cyclo-ligase OS=Lactobacill	381.52	42.47	4	8	8	40	1.055
U6EZQ4	23S rRNA (Uracil-5-)-methyltransferase RumA OS=L	380.92	58.26	9	24	24	67	0.940
A0A0D5MHF1	Alkaline shock protein OS=Lactobacillus helveticus G	379.87	36.67	1	5	5	35	0.998
A0A0F7H2D4	30S ribosomal protein S13 OS=Lactobacillus helvetic	376.83	47.41	3	8	8	72	0.931
U6F7Q4	Protein RecA OS=Lactobacillus helveticus CIRM-BIA	374.25	49.32	15	15	15	51	0.966
A0A0D5MIE1	Ferrous iron transporter A OS=Lactobacillus helvetic	374.08	34.22	9	18	18	46	0.995
U6FN58	50S ribosomal protein L32 OS=Lactobacillus helvetic	372.83	26.23	2	1	1	18	0.899
A0A0D5ML48	Fructose-2,6-bisphosphatase OS=Lactobacillus helve	371.94	77.78	5	15	15	71	1.048
A4ZH10	Conserved hypothetical penicillin-binding protein OS	370.43	45.48	9	14	14	39	1.106
A0A0D5MFU1	Glucosyltransferase OS=Lactobacillus helveticus GN=	370.10	39.62	11	12	12	41	0.986
U6F6R7	Oxidoreductase OS=Lactobacillus helveticus CIRM-B	368.21	49.62	7	13	13	34	0.956
U6F5E2	Uncharacterized protein OS=Lactobacillus helveticus	367.14	60.87	8	11	11	41	0.870
A0A0F7H2P3	N-acetylglucosamine-6-phosphate deacetylase OS=L	365.12	52.60	1	1	15	45	1.118
A8YXJ3	33 kDa chaperonin OS=Lactobacillus helveticus (stra	364.62	59.46	4	3	16	76	0.986

U6F6T2	Cytidylate kinase OS=Lactobacillus helveticus CIRM-	363.67	47.32	7	9	10	35	1.079
A0A0N9ZAI0	Cell surface protein OS=Lactobacillus helveticus GN=	360.60	53.28	11	17	17	40	1.004
A0A0D5MIX9	Tautomerase OS=Lactobacillus helveticus GN=ALV8	359.34	94.92	2	7	7	53	1.017
A0A0N9ZHA8	SlpX OS=Lactobacillus helveticus GN=ALV80_03275	357.40	35.46	3	16	16	38	1.197
A0A0F7D0P3	tRNA N6-adenosine threonylcarbamoyltransferase O	356.85	49.86	11	14	14	38	0.999
A0A0D5MFC8	3-oxoacyl-[acyl-carrier-protein] synthase 2 OS=Lact	356.17	28.85	4	1	10	33	1.072
A0A0P0A674	tRNA modification GTPase MnmE OS=Lactobacillus l	355.16	29.72	7	14	14	42	0.975
J3X9C4	Putative fibronectin domain protein OS=Lactobacillu	355.11	52.37	2	1	25	67	0.847
A0A0D5MIK8	HAD family hydrolase OS=Lactobacillus helveticus G	353.31	54.82	7	10	10	42	1.054
U6F342	UTP--glucose-1-phosphate uridylyltransferase OS=L	351.34	54.98	10	9	13	31	1.023
A0A0F7H4Y6	GTPase OS=Lactobacillus helveticus GN=TU99_085	351.29	40.11	12	14	15	45	0.878
A0A0D5MI41	Elongation factor P OS=Lactobacillus helveticus GN=	348.04	34.39	6	1	9	76	1.000
F3MRE2	Putative tRNA (cytidine(34)-2'-O)-methyltransferase	347.54	74.73	4	13	13	47	1.015
U6F2S5	Preprotein translocase OS=Lactobacillus helveticus (	347.24	26.95	8	4	4	18	0.993
U6F9Z3	Elongation factor P 1 OS=Lactobacillus helveticus CI	346.39	34.39	1	1	9	70	1.595
A4ZH32	ATP-dependent protease subunit HslV OS=Lactobac	345.66	46.55	4	8	9	47	1.022
U6F4Q3	Hydroxymethylglutaryl-CoA synthase OS=Lactobacil	342.96	39.28	11	13	13	36	0.969
U6F3K0	Uncharacterized protein OS=Lactobacillus helveticus	342.67	64.56	3	6	6	23	1.050
A8YVX2	N utilization substance protein B homolog OS=Lacto	341.58	75.38	8	9	9	35	0.965
A8YTK7	Aspartate--ammonia ligase OS=Lactobacillus helveti	341.43	67.06	11	20	20	49	0.946
A0A0D5MHC4	Endopeptidase OS=Lactobacillus helveticus GN=HU(	339.68	54.51	6	12	12	36	0.923
A0A0D5MGL5	Single-stranded DNA-binding protein OS=Lactobacill	337.21	46.51	4	7	7	29	1.115
A0A0N9ZT83	Aminotransferase V OS=Lactobacillus helveticus GN	335.01	59.33	32	4	22	60	0.903
A0A0R2M515	Nicotinate phosphoribosyltransferase OS=Lactobacil	332.71	40.67	4	1	18	46	0.644
A0A0D5MGT6	Alkaline phosphatase synthesis transcriptional regula	331.51	52.10	7	11	11	47	1.150
A8YTM0	Lysin OS=Lactobacillus helveticus (strain DPC 4571)	331.25	30.07	15	7	8	27	1.085
F3MRF6	Peptide chain release factor 2 OS=Lactobacillus helv	329.26	41.57	10	13	13	35	0.989
A0A0D5MGU4	Membrane protein OS=Lactobacillus helveticus GN=	329.14	65.05	4	11	11	31	1.112
A0A0D5MJB5	UPF0237 protein HUO_09120 OS=Lactobacillus helv	328.14	48.31	4	3	3	16	1.019
U6F288	Uncharacterized protein OS=Lactobacillus helveticus	327.94	35.37	8	8	8	38	0.908
U6F4T8	Glutamine ABC superfamily ATP binding cassette tra	327.87	44.31	7	13	13	63	0.937
A0A0P0A6A6	Phosphoglycerate mutase OS=Lactobacillus helvetic	326.29	69.86	6	13	13	44	1.034
A0A0D5MFC4	Acetyl-CoA carboxylase OS=Lactobacillus helveticus	325.20	25.78	8	4	4	17	0.951
A0A0N9Z9M2	Glutamine ABC transporter substrate-binding protein	324.79	52.57	3	1	12	33	1.026
F3MNC2	3-oxoacyl-[acyl-carrier-protein] synthase 2 OS=Lact	324.62	27.93	1	1	10	30	0.953
U6F248	HTH-type transcriptional regulator LacR OS=Lactoba	324.39	41.79	5	13	13	29	0.909
A8YVM7	GTPase Era OS=Lactobacillus helveticus (strain DPC	320.90	52.49	7	16	16	68	0.888
A0A0D5MJB9	Glutamine ABC transporter substrate-binding protein	320.79	54.78	3	2	13	36	0.907
A0A0N9Z6M0	Nucleoid-associated protein ALV80_01910 OS=Lacto	320.03	26.13	4	2	2	10	0.877
A8YXM3	50S ribosomal protein L30 OS=Lactobacillus helvetic	319.00	77.05	1	4	5	40	1.023
A0A0D5MKB1	Recombinase RarA OS=Lactobacillus helveticus GN=	318.23	45.31	8	16	16	36	0.989
F3MKA1	Thymidine kinase OS=Lactobacillus helveticus MTCC	318.18	89.95	10	18	18	63	0.945
U6F1H8	Cystathionine beta-lyase OS=Lactobacillus helveticus	317.93	45.94	5	15	15	44	1.102
U6F239	Carbamoyl-phosphate synthase (glutamine-hydrolyz	317.15	25.68	9	25	25	67	0.961
Q9L4G2	GTP cyclohydrolase 1 type 2 homolog OS=Lactobaci	315.32	46.42	7	10	10	31	0.960
A0A0N9ZYC9	RNA methyltransferase OS=Lactobacillus helveticus	315.06	34.22	1	1	11	38	1.073
U6F2H1	Tetrahydrofolate synthase OS=Lactobacillus helvetic	314.93	44.27	7	17	17	35	0.998
A0A0F7H516	tRNA (guanine-N(7)-)-methyltransferase OS=Lactob	314.87	47.93	3	10	10	42	0.915
U6F9F8	Phosphate acyltransferase OS=Lactobacillus helvetic	311.85	60.36	8	15	15	46	0.945
J3XAB6	Trehalose 6-P hydrolase OS=Lactobacillus helveticus	309.19	35.08	10	18	19	43	1.203
A0A0P0A4U0	Pseudouridine synthase OS=Lactobacillus helveticus	307.07	41.18	5	9	9	27	0.790
U6F1R7	Putative Acyl-ACP thioesterase OS=Lactobacillus hel	305.70	64.34	8	14	14	32	0.970
A8YUR9	30S ribosomal protein S15 OS=Lactobacillus helvetic	305.57	71.91	2	5	5	69	0.891
A0A0N9ZB07	Exodeoxyribonuclease 7 large subunit OS=Lactobac	304.74	33.33	7	13	13	26	1.010
A0A0D5MHV5	N5-carboxyaminoimidazole ribonucleotide mutase O	304.30	29.01	4	5	5	74	0.784

A0A0D5MKN4	Peptidase M16 OS=Lactobacillus helveticus GN=HU(	301.02	36.60	10	13	15	69	1.057
A0A0D5MIX4	ADP-ribose pyrophosphatase OS=Lactobacillus helve	300.65	50.72	3	11	11	24	1.076
A0A0D5MJU1	TetR family transcriptional regulator OS=Lactobacillu	299.51	24.00	9	5	5	23	0.994
A8YXI6	Peptidyl-tRNA hydrolase OS=Lactobacillus helveticus	299.03	81.08	8	15	15	46	0.979
U6F191	DNA polymerase III OS=Lactobacillus helveticus CIF	297.69	38.66	12	19	19	35	0.955
A0A0N9ZIR1	Nicotinate phosphoribosyltransferase OS=Lactobacil	294.69	40.84	1	1	20	53	0.918
U6F6K7	Uncharacterized protein OS=Lactobacillus helveticus	294.52	39.19	5	17	17	50	0.839
A0A0F7H5P9	Beta-galactosidase OS=Lactobacillus helveticus GN=	291.44	36.46	6	21	21	47	0.941
A0A0F7H2V2	3-ketoacyl-CoA thiolase OS=Lactobacillus helveticus	289.88	31.61	8	10	10	21	0.968
U6F7S1	Uncharacterized protein OS=Lactobacillus helveticus	288.57	34.90	2	5	5	18	1.043
A0A0D5MJS8	Methylenetetrahydrofolate--tRNA-(uracil-5-)-methylt	287.56	50.68	7	16	16	45	1.047
U6F859	GTP diphosphokinase OS=Lactobacillus helveticus C	285.85	32.04	13	27	27	47	0.957
J3XA25	Chromosome partition protein Smc OS=Lactobacillus	282.79	25.32	11	29	29	45	0.987
A0A0R2MUJ3	Phosphate-specific transport system accessory prote	279.88	61.78	4	13	13	34	0.989
A0A0D5MHB2	Lipase OS=Lactobacillus helveticus GN=HUO_04915	277.32	35.23	9	11	11	27	0.973
A0A0F7D071	DNA-directed DNA polymerase OS=Lactobacillus hel	277.04	32.95	11	29	29	49	1.009
A0A0D5MKX0	Aspartyl/glutamyl-tRNA(Asn/Gln) amidotransferase s	276.14	59.80	6	8	8	73	0.964
A0A0D5MHT7	Hydrolase TatD OS=Lactobacillus helveticus GN=HU	275.03	56.08	8	12	12	28	0.951
A8YV94	Uncharacterized protein OS=Lactobacillus helveticus	274.79	32.17	6	4	4	12	1.158
A0A0F7H354	Holo-[acyl-carrier-protein] synthase OS=Lactobacillu	271.44	54.24	4	7	7	21	1.007
F3MK90	Uncharacterized protein OS=Lactobacillus helveticus	270.79	46.32	6	12	13	55	1.294
U6F7F7	Morphine 6-dehydrogenase OS=Lactobacillus helvet	270.27	40.17	10	8	8	19	1.072
A0A0F7H5K8	Signal recognition particle receptor FtsY OS=Lactob	270.18	32.79	8	15	15	35	1.051
A8YUS5	Probable GTP-binding protein EngB OS=Lactobacillu	268.76	35.71	6	8	8	22	0.733
A0A0R2MSH5	Atp-dependent dna helicase ding OS=Lactobacillus l	267.10	24.06	12	21	21	43	0.985
A0A0F7H3R4	Ribosomal protein L11 methyltransferase OS=Lactol	265.65	45.08	15	12	13	43	0.984
F3MQC4	Initiation-control protein YabA OS=Lactobacillus helv	265.30	70.91	4	7	7	23	1.155
A0A0D5MHV1	Uncharacterized protein OS=Lactobacillus helveticus	261.51	69.89	3	9	9	42	1.164
U6F3M8	Transcriptional regulator, XRE family OS=Lactobacill	260.41	79.65	1	8	8	21	1.151
A0A0D5MIK6	Cysteine desulfurase OS=Lactobacillus helveticus GN	260.28	34.55	9	10	10	20	1.080
U6F053	Ribosome-recycling factor OS=Lactobacillus helvetic	258.91	57.30	4	13	13	59	1.030
U6F470	RND superfamily resistance-nodulation-cell division:|	255.92	28.25	13	24	24	45	1.019
U6F3E3	Nitro/flavin reductase OS=Lactobacillus helveticus C	255.22	46.43	7	11	11	38	1.034
U4QFE7	Uncharacterized protein OS=Lactobacillus helveticus	255.10	35.06	2	1	14	56	0.876
A0A0F7D0H2	50S ribosomal protein L35 OS=Lactobacillus helvetic	254.59	30.30	4	3	3	28	0.878
F3MKE7	Cell shape-determining protein MreC OS=Lactobacill	254.07	50.53	5	2	12	24	0.991
U6F463	Putative pyruvate, phosphate dikinase regulatory pr	252.08	35.25	7	9	9	24	0.757
A8YUW1	Dihydrofolate reductase OS=Lactobacillus helveticus	249.94	43.60	8	7	7	23	0.899
A8YVY6	Lysin OS=Lactobacillus helveticus (strain DPC 4571)	249.66	22.41	11	8	10	53	1.046
A0A0D5MHQ8	HAD family hydrolase OS=Lactobacillus helveticus G	248.97	40.54	7	10	10	24	0.931
A0A0R2MV26	tRNA (guanine-N(1)-)-methyltransferase OS=Lactob	246.57	54.09	8	10	10	35	1.040
A0A0N9ZAC6	Adenine phosphoribosyltransferase OS=Lactobacillus	245.87	67.03	4	11	11	34	1.165
A0A0N9Z9V5	33 kDa chaperonin OS=Lactobacillus helveticus GN=	244.14	46.28	2	1	14	60	1.037
U6F2L5	DNA topoisomerase 4 subunit A OS=Lactobacillus he	242.40	32.40	10	25	27	43	0.873
A0A0F7H4L7	Cell division protein DivIVA OS=Lactobacillus helveti	240.67	29.81	10	1	8	25	1.057
A0A0D5MH96	ATPase OS=Lactobacillus helveticus GN=HUO_0481	239.96	26.69	12	24	24	35	0.989
A8YVM2	RNA polymerase sigma factor SigA OS=Lactobacillus	239.45	41.01	9	15	15	47	0.871
U6FKM7	Phosphonate ABC superfamily ATP binding cassette	238.98	44.41	12	12	12	35	1.052
A0A0F7H575	Glutamine ABC transporter ATP-binding protein OS=	238.63	49.59	10	15	15	38	1.224
U6F9N9	Xaa-Pro dipeptidase OS=Lactobacillus helveticus CIF	238.57	68.18	8	15	15	30	1.100
U6EZM8	Isoprenyl transferase OS=Lactobacillus helveticus C	237.91	34.02	4	9	9	27	0.931
A8YVM8	Endoribonuclease YbeY OS=Lactobacillus helveticus	237.25	31.61	6	6	6	29	1.025
A8YUN9	UDP-N-acetylglucosamine--N-acetylmuramyl-(penta	236.44	31.52	7	13	13	32	0.918
A0A0D5ML53	Asparaginase OS=Lactobacillus helveticus GN=HUO_	236.26	33.23	10	7	7	13	0.973
F3MRC1	Uncharacterized protein OS=Lactobacillus helveticus	235.89	54.36	3	6	6	17	0.945

A0A0F7H467	Pseudouridine synthase OS=Lactobacillus helveticus	234.80	52.54	10	11	11	19	1.065
U6F6L0	Uncharacterized protein OS=Lactobacillus helveticus	234.07	62.96	1	8	8	38	1.113
A0A0N9Z8S6	Sulfurtransferase OS=Lactobacillus helveticus GN=A	233.76	39.85	5	5	5	13	1.089
A0A0D5MJH0	DNA topoisomerase 4 subunit B OS=Lactobacillus he	230.94	31.94	12	21	21	39	0.845
A0A0D5MJY4	Lipid kinase OS=Lactobacillus helveticus GN=HUO_(	229.85	36.93	5	10	10	34	0.979
A0A0D5MGW7	Histidine kinase OS=Lactobacillus helveticus GN=AL	229.25	38.41	2	5	5	18	1.213
J3ZEU3	Cell shape-determining protein MreC OS=Lactobacill	228.91	47.00	1	1	11	19	0.915
A0A0P0A514	Uncharacterized protein OS=Lactobacillus helveticus	227.41	27.57	8	6	6	17	1.029
A0A0D5MHM9	Redox-sensing transcriptional repressor Rex OS=Lac	227.40	38.14	5	8	8	17	1.095
A8YUY2	Citrate lyase acyl carrier protein OS=Lactobacillus he	226.81	68.04	4	11	11	43	0.961
U6F3H9	Type I restriction-modification system DNA-methyltr	226.24	27.03	3	12	12	18	1.019
A0A0D5MK07	Adenylate cyclase OS=Lactobacillus helveticus GN=I	225.65	37.25	7	6	6	12	1.041
A0A0D5MGZ7	Ribosomal silencing factor RsfS OS=Lactobacillus he	225.37	57.39	3	1	6	34	0.963
A0A0D5MJN1	tRNA-specific 2-thiouridylase MnmA OS=Lactobacillu	222.99	40.27	4	14	14	33	0.876
A8YVQ5	tRNA pseudouridine synthase B OS=Lactobacillus he	222.87	47.47	19	11	12	27	1.067
A0A0F7H3B1	rRNA methyltransferase OS=Lactobacillus helveticus	222.77	50.40	4	12	12	30	0.853
U6F2N1	Putative extracellular protein OS=Lactobacillus helve	221.62	42.68	8	10	10	21	1.072
U6F4F4	Serine/threonine protein phosphatase Stp1 OS=Lact	220.69	28.35	6	1	7	20	1.039
F3MLM0	Putative methyltransferase OS=Lactobacillus helveti	219.62	52.19	9	10	10	35	1.053
U6F065	Protein GrpE OS=Lactobacillus helveticus CIRM-BIA	218.84	64.32	7	13	14	69	0.992
A8YWE4	Transcriptional repressor NrdR OS=Lactobacillus hel	218.14	48.39	2	8	8	31	0.987
A0A0D5MHD4	Geranyl transferase OS=Lactobacillus helveticus GN	217.03	38.41	7	10	10	37	0.982
A0A0D5MJM3	Putative gluconeogenesis factor OS=Lactobacillus he	216.44	33.72	4	10	10	32	1.031
A8YW77	Phosphoglycerate mutase OS=Lactobacillus helvetic	216.40	60.29	5	15	15	37	0.952
A8YXA7	Transcription regulator OS=Lactobacillus helveticus	216.27	22.76	10	9	9	20	1.025
U6F4D9	Mannose-6-phosphate isomerase OS=Lactobacillus l	216.09	54.52	13	17	18	50	1.018
A0A0D5ML65	Exopolysaccharide biosynthesis protein OS=Lactoba	215.88	23.26	11	6	6	12	1.119
A8YVW3	Primosomal protein N' OS=Lactobacillus helveticus (	215.38	27.78	11	22	22	43	1.050
U6F0T9	Competence negative regulator MecA OS=Lactobaci	214.20	32.11	8	8	8	23	0.808
U6F227	Chaperone protein DnaJ OS=Lactobacillus helveticus	213.62	40.36	7	16	16	44	0.962
A8YWC6	UPF0348 protein lhv_1590 OS=Lactobacillus helvetic	213.47	36.55	8	14	14	29	0.937
A0A0D5MIR3	Aminotransferase V OS=Lactobacillus helveticus GN	213.24	48.45	1	1	18	44	0.819
U6EZH5	Aspartokinase OS=Lactobacillus helveticus CIRM-BI/	213.06	26.48	11	8	9	25	1.084
U6F3J9	Putative modification methylase OS=Lactobacillus he	213.02	39.34	9	12	12	41	0.987
J3ZF68	Flavodoxin OS=Lactobacillus helveticus R0052 GN=I	212.55	27.70	2	4	4	44	1.061
F3MQK2	Glutamate racemase OS=Lactobacillus helveticus M1	212.54	53.76	7	11	11	20	1.087
A0A0R2MFV8	D-alanine--poly(phosphoribitol) ligase subunit 2 OS=	211.05	34.18	4	3	3	30	1.134
A0A0D5MJZ9	Uncharacterized protein OS=Lactobacillus helveticus	210.64	56.59	1	4	4	18	0.976
U6F3E4	Putative glucan modifying protein OS=Lactobacillus	210.03	20.08	7	5	19	38	1.025
A0A0D5MGL0	RNA-binding protein OS=Lactobacillus helveticus GN	208.57	59.46	7	3	3	10	1.432
U6F539	2,5-diketo-D-gluconate reductase OS=Lactobacillus	207.94	61.82	8	17	17	37	1.098
A0A0P0A4Q0	ABC transporter OS=Lactobacillus helveticus GN=AL	207.17	28.57	7	17	17	31	0.977
U6F3E9	HD domain protein OS=Lactobacillus helveticus CIRI	206.66	48.17	8	11	11	26	0.902
A0A0D5MI88	Holliday junction ATP-dependent DNA helicase RuvA	206.22	27.55	7	5	5	16	0.879
F3MN34	Phosphotransferase system OS=Lactobacillus helvet	206.17	22.41	9	10	14	26	0.985
A0A0N9ZJR7	Esterase OS=Lactobacillus helveticus GN=ALV80_08	205.41	34.34	7	8	8	22	1.109
A0A0D5MJF8	GntR family transcriptional regulator OS=Lactobacill	204.34	62.66	9	13	13	26	1.026
A0A0D5MKC3	Ribosomal RNA small subunit methyltransferase G C	204.22	51.67	5	13	13	35	0.992
F3MK88	Putative cobalamin adenosyltransferase OS=Lactoba	203.59	56.45	6	11	11	31	0.826
A8YTN8	Glucosamine-6-phosphate deaminase OS=Lactobaci	202.23	63.18	8	16	16	37	1.133
U6F4U1	Acetyl-coenzyme A carboxylase carboxyl transferase	201.12	45.05	6	13	13	35	0.804
Q93K33	Mevalonate kinase OS=Lactobacillus helveticus GN=	200.62	52.32	8	14	14	27	0.906
A8YVW8	Exodeoxyribonuclease 7 small subunit OS=Lactobac	200.20	71.25	6	6	6	29	1.055
U4QDJ1	YslB-like protein OS=Lactobacillus helveticus CIRM-I	199.38	65.41	10	7	7	23	1.105
A8YU15	Putative acetyltransferase OS=Lactobacillus helvetic	198.90	41.21	8	6	6	13	1.080

U6F1V5	Diaminopimelate decarboxylase OS=Lactobacillus he	198.24	33.79	9	15	15	30	1.079
F3MMT9	Uncharacterized protein OS=Lactobacillus helveticus	197.92	34.43	8	8	8	27	1.011
J3WKV7	Transcriptional regulator OS=Lactobacillus helveticu	197.20	25.68	11	8	8	17	1.071
U6F3P4	Spermidine/putrescine import ATP-binding protein P	196.18	38.36	7	12	12	25	1.017
Q5UU96	LacM OS=Lactobacillus helveticus GN=lacM PE=4 S\	195.37	26.73	6	9	9	26	0.943
U6F579	Uncharacterized protein OS=Lactobacillus helveticus	194.19	47.79	6	11	11	29	1.003
U6F1A9	Phosphoserine phosphatase OS=Lactobacillus helvet	193.10	33.45	10	9	9	19	1.068
U6F5K7	Exopolysaccharide biosynthesis protein OS=Lactoba	191.43	43.91	10	13	13	20	1.047
A0A0F7H6W7	Cytochrome C552 OS=Lactobacillus helveticus GN=7	191.23	43.93	8	16	16	43	1.039
A0A0D5MJM2	FMN-binding protein OS=Lactobacillus helveticus GN	191.05	25.78	2	4	4	19	1.069
A0A0D5MLA4	Sporulation initiation inhibitor Soj OS=Lactobacillus	190.36	28.96	7	6	6	13	1.000
U6F596	Glycerol-3-phosphate ABC transporter OS=Lactobac	189.38	44.34	12	14	20	50	0.960
A0A0F7H760	DeoR faimly transcriptional regulator OS=Lactobacil	188.29	21.51	13	5	5	9	0.999
U6F5T7	Capsid protein OS=Lactobacillus helveticus CIRM-BI	187.89	28.50	1	5	5	12	1.041
U6EZX0	Uncharacterized protein OS=Lactobacillus helveticus	187.81	38.15	12	11	11	21	1.103
A0A0P0A5P3	Nitroreductase OS=Lactobacillus helveticus GN=ALV	187.73	22.22	5	4	4	19	1.063
A0A0N9Z9C2	Lactocepin S-layer protein OS=Lactobacillus helvetic	186.59	43.03	7	8	8	25	1.109
A0A0N9ZII6	Ribosome biogenesis GTPase A OS=Lactobacillus he	186.45	41.70	6	10	10	26	0.876
U6F0R3	PTS system, glucose-specific IIA component OS=La	184.29	57.76	3	9	9	29	0.997
A0A0F7H6H5	Cysteine synthase OS=Lactobacillus helveticus GN=7	183.52	28.38	4	8	8	16	1.231
A0A0F7D0I8	D-alanyl-D-alanine carboxypeptidase OS=Lactobacill	183.45	26.22	10	12	12	19	0.995
A0A0D5ML45	DNA-binding protein OS=Lactobacillus helveticus GN	182.77	65.93	7	8	8	30	0.884
A0A0N9ZIZ0	Septation ring formation regulator EzrA OS=Lactoba	181.84	32.86	11	16	17	29	1.034
U6F264	Putative nucleolar protein OS=Lactobacillus helveticu	180.38	32.83	8	11	11	25	0.926
U6F4E4	PTS family mannose porter, IID component OS=Lac	180.31	30.07	5	10	10	31	0.957
U6F4K3	Halo peroxidase OS=Lactobacillus helveticus CIRM-E	180.25	49.43	8	11	11	41	0.920
A0A0N7HHM3	Peptidyl-prolyl cis-trans isomerase OS=Lactobacillus	177.94	44.33	5	8	8	29	1.028
A0A0P0A323	50S ribosomal protein L36 OS=Lactobacillus helvetic	176.82	60.53	2	3	3	17	0.655
F0NU97	Phosphonates import ATP-binding protein PhnC OS=	175.60	48.02	6	11	11	23	0.984
U6F4X6	Hypoxanthine phosphoribosyltransferase OS=Lactob	175.56	44.63	11	9	9	20	0.647
U6F2A0	UDP-galactopyranose mutase OS=Lactobacillus helv	175.44	34.70	8	13	13	27	1.018
U6F6A6	Hemolysin A OS=Lactobacillus helveticus CIRM-BIA	174.27	28.52	8	7	7	18	0.988
A0A0P0A4L9	DUTP diphosphatase OS=Lactobacillus helveticus GI	173.97	22.40	3	4	4	17	1.010
U6F3K4	Peptidase T OS=Lactobacillus helveticus CIRM-BIA 9	173.92	35.75	8	13	13	35	1.042
U6F611	Uncharacterized protein OS=Lactobacillus helveticus	172.93	21.99	3	3	3	9	1.124
A8YU24	Glutamine ABC transporter OS=Lactobacillus helveti	172.26	36.40	10	8	9	15	1.026
F3MR08	Putative sex pheromone biosynthesis protein OS=La	171.64	32.53	5	3	10	15	1.139
A0A0D5MH81	Magnesium ABC transporter ATPase OS=Lactobacillu	170.21	25.22	9	25	25	44	1.013
A0A0R2M6S6	Oligopeptide ABC transporter substrate binding prot	169.26	21.22	4	10	14	27	1.150
U6F6D4	Uncharacterized protein OS=Lactobacillus helveticus	169.25	45.30	1	8	8	32	0.951
U4QK39	CamS sex pheromone cAM373 OS=Lactobacillus hel	168.54	28.00	4	1	8	11	0.890
A0A0F7CZU3	Ribosomal RNA small subunit methyltransferase A O	168.22	47.62	15	14	15	30	0.899
A4UAD9	Foldase protein PrsA OS=Lactobacillus helveticus CN	168.14	34.88	5	11	11	22	1.032
A0A0D5MJZ3	Serine/threonine protein phosphatase OS=Lactobac	167.73	29.48	9	1	7	18	0.949
A0A0D5MJ67	LexA repressor OS=Lactobacillus helveticus GN=lex/	167.01	28.85	6	7	7	25	1.070
U6F322	Sodium/dicarboxylate symporter family protein OS=	166.35	17.47	8	8	8	21	1.111
F3MRL3	Response regulator OS=Lactobacillus helveticus MT(	165.77	36.13	7	8	8	17	0.976
U6F5S6	Zinc ABC transporter OS=Lactobacillus helveticus CI	165.55	61.40	6	11	11	38	1.044
A0A0D5MI53	DNA repair protein RecN OS=Lactobacillus helveticu	164.91	32.14	7	18	18	41	0.968
A0A0N9ZJ11	UDP-N-acetylmuramyl peptide synthase OS=Lactoba	164.90	30.00	9	2	14	36	0.956
F3MKA0	UDP-N-acetylmuramyl tripeptide synthase OS=Lactc	164.90	28.00	2	1	13	31	1.019
A8YTJ1	UPF0297 protein lhv_0439 OS=Lactobacillus helvetic	162.93	47.06	13	4	4	19	1.181
A8YUB8	Ribonuclease Y OS=Lactobacillus helveticus (strain I	162.86	24.13	7	16	16	26	1.016
A0A0N7HHK6	Ribosomal RNA small subunit methyltransferase B O	161.95	30.77	12	12	12	25	0.997
A0A0D5MKZ2	Uncharacterized protein OS=Lactobacillus helveticus	161.23	33.33	5	4	4	12	1.136

U6F3F9	Putative transcriptional regulator OS=Lactobacillus h	160.78	28.13	9	8	8	19	0.976
U6F2S3	Putative hydrolase OS=Lactobacillus helveticus CIRN	159.86	44.00	6	7	8	24	0.874
A0A0N9ZCG8	Glycerate kinase OS=Lactobacillus helveticus GN=Al	158.18	31.25	10	11	11	23	1.085
A0A0D5MGQ0	Uncharacterized protein OS=Lactobacillus helveticus	155.63	66.04	3	5	5	10	0.971
A0A0D5MI35	Ribonuclease 3 OS=Lactobacillus helveticus GN=rnc	153.95	28.51	3	7	7	20	1.019
F0NX78	Prophage Lp1 protein 65 OS=Lactobacillus helveticu	153.27	31.53	3	1	11	40	0.983
U6F3N3	N-acetyltransferase OS=Lactobacillus helveticus CIR	152.38	15.85	7	3	3	7	1.056
A0A0D5MJQ5	GTP pyrophosphokinase OS=Lactobacillus helveticus	152.33	42.38	3	9	9	14	0.979
A0A0D5MGF5	Regulatory protein OS=Lactobacillus helveticus GN=	151.92	23.64	5	7	7	11	0.861
U6F642	Glutamate/aspartate ABC superfamily ATP binding c	151.05	41.27	11	11	11	26	0.952
A0A0D5MIP2	Ribosome-binding factor A OS=Lactobacillus helveti	151.04	20.66	3	3	3	18	0.845
A0A0D5MIR1	7,8-dihydroneopterin aldolase OS=Lactobacillus helv	150.87	55.17	4	6	6	20	0.841
F3MQA5	Phosphate ABC transporter phosphate-binding prote	150.68	27.84	8	9	9	19	1.038
A0A0R2MKR1	Nucleotide-binding protein IV62_GL001024 OS=Lact	150.45	41.24	7	12	12	25	0.947
A0A0N9ZUH2	Phosphoglycerate mutase OS=Lactobacillus helvetic	149.86	51.76	8	11	11	23	1.027
A8YTQ9	Ribonuclease P protein component OS=Lactobacillus	148.09	40.98	2	5	5	11	0.971
A0A0D5MJ50	Uncharacterized protein OS=Lactobacillus helveticus	146.95	18.25	8	3	3	15	0.949
U6F1K0	Ribonuclease M5 OS=Lactobacillus helveticus CIRM-	146.85	52.13	7	9	9	20	0.997
U6F7Z4	Secreted protein OS=Lactobacillus helveticus CIRM-	146.80	40.90	9	19	19	31	1.062
F3MLY7	Uncharacterized protein (Fragment) OS=Lactobacillu	146.59	58.79	8	9	9	13	1.058
U6F2H8	Ribulose-phosphate 3-epimerase OS=Lactobacillus h	146.33	31.02	7	5	5	9	0.984
A0A0F7H448	Uncharacterized protein OS=Lactobacillus helveticus	145.53	60.00	7	5	5	7	0.993
A0A0D5MKN7	3'-5' exonuclease OS=Lactobacillus helveticus GN=H	145.26	23.03	9	6	6	13	0.957
A0A0R2MJL1	Glucan modifying protein OS=Lactobacillus helveticu	145.19	15.96	2	1	15	29	0.977
F3MM44	Uncharacterized protein OS=Lactobacillus helveticus	144.37	48.52	8	12	12	21	1.002
U6F372	HAD-superfamily hydrolase OS=Lactobacillus helveti	141.84	48.94	10	10	10	19	1.082
A0A0F7H5U2	Aminopeptidase OS=Lactobacillus helveticus GN=TU	141.69	27.98	7	15	15	23	1.066
A8YWD8	Transposase ORF_A OS=Lactobacillus helveticus (st	140.81	46.04	15	9	9	18	1.032
A0A0F7H5Q6	Hydrolase OS=Lactobacillus helveticus GN=TU99_1(	140.66	27.82	10	7	7	13	1.059
U6F4M1	Glycosyl transferase CpoA OS=Lactobacillus helvetic	140.61	45.53	9	15	15	32	1.003
A0A0D5MKD1	Uncharacterized protein OS=Lactobacillus helveticus	140.04	33.75	6	3	3	9	1.078
A0A0F7CZY4	Acetate kinase OS=Lactobacillus helveticus GN=ack/	139.91	26.14	9	8	9	19	0.961
A8YTT9	SsrA-binding protein OS=Lactobacillus helveticus (st	138.96	43.42	3	7	8	21	0.917
F3MMV8	PTS system IIA OS=Lactobacillus helveticus MTCC 5	137.91	42.42	4	6	6	10	1.103
A0A0D5MJU8	Uncharacterized protein OS=Lactobacillus helveticus	137.88	44.00	2	4	4	16	0.878
A8YW41	Chromosomal replication initiator protein DnaA OS=	137.50	29.45	9	12	12	18	0.949
U6F5P1	Putative ATP-dependent helicase yeeB OS=Lactobac	136.38	22.65	1	12	12	21	0.987
A0A0D5MJV8	ATP synthase subunit a OS=Lactobacillus helveticus	136.32	8.86	5	3	3	10	0.984
A0A0N9ZB59	Ribosome maturation factor RimP OS=Lactobacillus	136.11	41.14	7	5	5	17	0.805
W5XM93	Folylpolyglutamate synthase OS=Lactobacillus helve	135.79	29.61	13	11	11	20	0.917
U6F2S2	Penicillin-binding protein OS=Lactobacillus helveticu	134.91	37.26	8	11	11	18	1.058
A0A0D5MJD9	ATP-dependent helicase/nuclease subunit A OS=Lac	134.71	12.38	9	14	14	20	1.019
A0A0R2MUU7	Transcriptional regulator OS=Lactobacillus helveticu	134.61	31.18	11	14	14	27	1.034
A0A0F7H379	30S ribosomal protein S20 OS=Lactobacillus helvetic	134.41	40.00	3	4	4	35	0.764
A0A0R2MMN2	Ribosomal silencing factor RsfS OS=Lactobacillus he	131.98	57.39	1	1	6	17	1.141
F3MN18	ABC transporter OS=Lactobacillus helveticus MTCC !	131.49	25.93	9	11	12	20	0.977
U6F8B7	Exonuclease SbcCD, D subunit OS=Lactobacillus hel	130.38	31.28	9	10	10	16	1.029
A8YV45	Ribonuclease HII OS=Lactobacillus helveticus (strair	130.09	36.80	7	8	8	10	1.111
A0A0F7D083	Antitoxin OS=Lactobacillus helveticus GN=ALV80_0!	129.21	68.48	2	5	5	13	1.122
A0A0F7H4S8	Multidrug ABC transporter ATP-binding protein OS=	128.98	29.55	3	6	6	12	1.003
A8YVK5	Isopentenyl-diphosphate delta-isomerase OS=Lacto	128.42	43.20	7	14	14	26	0.989
U6F832	Inosine-uridine preferring nucleoside hydrolase OS=	128.34	28.80	10	8	9	27	0.922
A0A0D5MJB6	RNA-binding protein OS=Lactobacillus helveticus GN	127.49	21.89	7	9	9	20	0.903
U6F7V2	Trehalose operon transcriptional repressor OS=Lact	127.01	42.32	1	9	9	19	1.061
U6F3H2	HD superfamily phosphohydrolase OS=Lactobacillus	126.79	24.67	8	10	10	19	1.057

A0A0D5MH56	Aldose epimerase OS=Lactobacillus helveticus GN=/	126.00	33.33	6	6	6	14	1.084
A0A0R2MS81	ATP synthase epsilon chain OS=Lactobacillus helvet	125.15	45.21	6	5	5	19	1.051
A0A0R2MJQ6	Methionyl-tRNA formyltransferase OS=Lactobacillus	124.76	48.70	15	14	16	39	1.002
F0NSA9	Oligopeptide ABC transporter, permease protein OS	123.37	21.94	6	7	7	21	1.064
A0A0N9ZUF3	Aspartate racemase OS=Lactobacillus helveticus GN	123.23	31.33	6	6	6	9	0.948
A0A0N9ZDL1	Nicotinate-nucleotide pyrophosphorylase OS=Lactob	123.02	42.29	3	1	9	13	1.297
U6F8J3	Nicotinate-nucleotide pyrophosphorylase OS=Lactob	123.02	37.63	3	1	9	14	1.071
J7LMF8	Uncharacterized protein OS=Lactobacillus helveticus	122.35	41.95	5	8	8	14	1.074
U6FD26	Capsular polysaccharide biosynthesis protein OS=La	121.93	25.77	12	7	7	24	0.959
A0A0D5MG72	Competence protein ComE OS=Lactobacillus helveti	121.80	32.08	4	4	4	8	1.071
A8YW34	ABC transporter OS=Lactobacillus helveticus (strain	121.51	29.57	9	11	16	30	0.948
U6F3Q2	Phosphotyrosine protein phosphatase OS=Lactobaci	120.89	22.58	4	3	3	8	1.075
U6F1X4	YueI protein OS=Lactobacillus helveticus CIRM-BIA	120.08	47.10	5	5	6	14	1.034
U6F3J5	Primosomal protein DnaI OS=Lactobacillus helveticu	119.66	23.18	8	6	6	10	0.955
U6F6M3	Capsular biosynthesis protein OS=Lactobacillus helv	119.55	52.63	3	14	14	26	0.939
A0A0D5MHY9	Uncharacterized protein OS=Lactobacillus helveticus	118.87	48.89	4	10	10	27	1.043
U6F4T1	L-fucose operon regulator OS=Lactobacillus helvetic	118.60	42.34	5	8	8	15	1.121
A0A0R2MQ55	Penicillin binding protein 1A OS=Lactobacillus helvet	117.98	15.40	9	10	10	12	1.006
A0A0D5MHE7	S-layer protein OS=Lactobacillus helveticus GN=HU	117.82	27.08	19	10	10	15	1.201
A0A0D5MHK5	Phosphate import ATP-binding protein PstB OS=Lac	117.44	23.48	6	6	6	9	0.995
F3MM51	NAD-dependent deacetylase OS=Lactobacillus helve	117.03	41.03	11	9	9	16	1.095
A0A0R2MDT4	Uncharacterized protein OS=Lactobacillus helveticus	116.72	26.39	4	1	1	2	0.913
A0A0R2MU56	Heat shock protein hsp OS=Lactobacillus helveticus	116.46	34.56	2	1	4	42	0.901
U6EZW2	Putative Galactose-1-phosphate uridylyltransferase (	115.98	23.60	8	6	6	12	0.980
U6F5T2	GpG protein OS=Lactobacillus helveticus CIRM-BIA	115.85	38.46	1	12	12	17	1.030
A0A0F7H1Z7	Degv family protein OS=Lactobacillus helveticus GN	115.66	28.48	9	7	7	12	1.009
U6EZI0	4-hydroxy-tetrahydrodipicolinate reductase OS=Lact	114.79	27.80	12	6	6	14	1.134
A8YVT3	Ribosome maturation factor RimM OS=Lactobacillus	114.44	68.42	5	11	11	28	1.080
A0A0F7H651	Transcriptional regulator OS=Lactobacillus helveticu	113.15	36.00	8	2	2	6	1.060
A0A0N7HHT8	ABC transporter OS=Lactobacillus helveticus GN=AL	112.81	24.94	6	9	10	18	1.029
U6F6L3	Uncharacterized protein OS=Lactobacillus helveticus	112.43	66.67	8	5	5	15	0.444
A0A0F7H4C0	Helicase OS=Lactobacillus helveticus GN=TU99_032	112.12	38.54	9	15	15	30	1.044
A0A0D5MFZ1	Serine protease OS=Lactobacillus helveticus GN=HU	111.80	20.19	10	8	8	13	0.970
A8YUE7	Uracil-DNA glycosylase OS=Lactobacillus helveticus	111.47	21.98	6	5	5	10	0.943
A0A0D5MJ79	Acetyltransferase OS=Lactobacillus helveticus GN=A	111.08	30.88	8	4	4	11	1.141
A0A0R2MUW5	ATP-dependent RecD-like DNA helicase OS=Lactoba	110.43	13.67	12	10	10	12	0.998
A0A0P0A3K4	Heat-inducible transcription repressor HrcA OS=Lact	109.52	20.92	7	6	6	9	0.994
W5XNE4	Putative ribosome biogenesis GTPase RsgA OS=Lact	108.90	16.13	9	5	5	11	0.864
A0A0N9ZWC8	Esterase OS=Lactobacillus helveticus GN=ALV80_00	108.68	43.16	6	8	8	12	1.003
U6F6N1	Uridine kinase OS=Lactobacillus helveticus CIRM-BI/	108.29	24.76	5	4	4	6	1.055
A8YWB1	Penicillin-binding protein OS=Lactobacillus helveticu	107.16	23.05	10	13	13	19	1.057
A0A0P0A475	Transcriptional regulator OS=Lactobacillus helveticu	106.86	52.44	8	13	14	28	1.107
U6F911	Peptide methionine sulfoxide reductase MsrA OS=La	106.55	74.60	6	11	11	27	1.103
U6F3P8	YbbR OS=Lactobacillus helveticus CIRM-BIA 951 GN	106.52	37.30	12	12	12	16	1.036
A0A0F7H6J2	MarR family transcriptional regulator OS=Lactobacill	105.95	30.97	4	4	4	5	0.952
A0A0D5MHB9	DNA polymerase III subunit delta OS=Lactobacillus	104.72	27.37	7	8	9	15	0.980
A0A0D5MJ31	UvrABC system protein A OS=Lactobacillus helveticu	104.16	19.66	8	17	17	20	0.991
A0A0P0A7L8	DNA/pantothenate metabolism flavoprotein OS=Lac	103.81	31.49	12	10	11	20	1.075
A0A0F7H5B5	ABC transporter ATP-binding protein OS=Lactobacill	103.79	26.38	5	6	6	11	0.983
U6F4H5	Type I restriction modification DNA specificity doma	103.34	32.67	3	12	12	16	0.980
A0A0D5MH09	Fructose-2,6-bisphosphatase OS=Lactobacillus helve	102.01	42.04	6	9	9	19	1.128
A0A0N7HHG1	16S rRNA methyltransferase OS=Lactobacillus helve	101.67	43.54	8	7	7	20	0.944
Q93K32	Mevalonate diphosphate decarboxylase OS=Lactoba	101.46	27.19	6	2	8	17	0.935
W5XEX9	3-oxoacyl-(Acyl-carrier protein) reductase OS=Lacto	101.38	35.21	8	6	6	13	1.004
A0A0D5MJF3	Carbamoyl phosphate synthase small subunit OS=La	100.87	21.37	6	8	8	15	0.954

A0A0D5MKE1	Aggregation promoting factor surface protein OS=La	100.11	32.77	6	4	4	18	1.134
A0A0D5MHX8	MFS transporter OS=Lactobacillus helveticus GN=HU	99.95	9.54	5	4	4	14	0.968
A0A0N9ZGB6	TetR family transcriptional regulator OS=Lactobacillu	99.91	22.03	10	4	5	17	0.991
J3WMU4	Trehalose PTS II ABC OS=Lactobacillus helveticus R	99.76	12.58	2	7	7	18	1.124
A0A0F7H2H5	DNA repair protein radA OS=Lactobacillus helveticus	99.48	19.87	8	7	7	10	0.900
U6FI27	Putative acetyltransferase OS=Lactobacillus helvetic	99.23	36.59	5	7	7	11	0.926
J4BRW1	TetR family transcriptional regulator OS=Lactobacillu	99.19	13.16	15	2	3	5	0.894
A8YVX8	Putative v-type sodium ATP synthase subunit OS=La	99.09	17.14	13	7	7	11	0.899
U6F5Y5	Possible methylated-DNA--[protein]-cysteine S-meth	97.85	39.77	9	6	7	12	0.974
A0A0D5MIL9	Cell division protein FtsA OS=Lactobacillus helveticu	97.26	19.91	8	9	9	18	1.002
U6F045	Uncharacterized protein OS=Lactobacillus helveticus	97.12	41.41	8	3	3	14	0.949
A8YUT2	N-acetyldiaminopimelate deacetylase OS=Lactobacil	96.59	34.38	11	11	11	16	1.029
A0A0D5MKK3	Uncharacterized protein OS=Lactobacillus helveticus	96.54	40.00	2	3	3	10	0.963
A0A0D5MKI6	UPF0291 protein HUO_05275 OS=Lactobacillus helv	95.52	29.27	8	3	3	17	0.984
U4QAI7	Prophage Lp1 protein 6 OS=Lactobacillus helveticus	95.49	30.25	3	1	11	38	0.994
A0A0R2MTY7	Diphosphomevalonate decarboxylase OS=Lactobacil	94.92	28.22	1	1	7	14	1.225
U6F504	ABC superfamily ATP binding cassette transporter,bi	92.84	36.25	12	10	10	19	1.103
U6F6K2	Possible type I site-specific deoxyribonuclease OS=L	90.97	14.40	4	15	15	22	1.039
A0A0F7H417	CDP-glycerol:glycerophosphate glycerophosphotrans	90.56	9.64	10	4	4	6	1.168
A0A0D5MK74	GMP synthase OS=Lactobacillus helveticus GN=HUC	89.28	9.46	5	2	2	5	1.048
J3ZEQ7	Uncharacterized protein OS=Lactobacillus helveticus	88.72	26.71	7	7	7	14	0.958
A0A0F7H2M2	Ferredoxin--NADP reductase OS=Lactobacillus helve	88.64	26.39	9	6	6	7	1.189
J4BTN7	ABC transport protein ATP-binding component OS=l	87.78	37.69	11	11	12	15	1.032
A0A0F7H663	FAD:protein FMN transferase OS=Lactobacillus helve	87.08	25.24	7	6	6	7	1.008
U6F078	Uncharacterized protein OS=Lactobacillus helveticus	86.87	28.19	1	5	5	8	1.067
A0A0D5MH34	Phosphate ABC transporter permease OS=Lactobaci	86.86	8.76	7	3	3	3	0.918
A0A0D5MJ23	Tyrosine recombinase XerD OS=Lactobacillus helvet	86.56	33.55	2	10	10	15	0.912
A0A0D5MGV8	Uncharacterized protein OS=Lactobacillus helveticus	86.42	30.00	1	2	2	8	0.927
A0A0F7D0L1	Histidine kinase OS=Lactobacillus helveticus GN=TU	86.41	15.71	5	5	5	6	1.064
U6F6S9	Putative 5-nucleotidase OS=Lactobacillus helveticus	86.38	16.20	7	7	7	18	0.977
U6F0C2	Pseudouridine synthase OS=Lactobacillus helveticus	85.33	8.05	11	2	2	4	0.927
U6F5Z0	Glutamine ABC superfamily ATP binding cassette tra	85.06	43.82	7	8	8	11	0.997
A0A0R2MQ11	Energy-coupling factor transporter ATP-binding prot	84.88	19.43	9	5	5	8	1.005
A0A0D5MKF8	DNA nuclease OS=Lactobacillus helveticus GN=HUC	83.48	28.52	5	8	8	13	0.974
W5XLK8	Glycosyltransferase group 1 protein OS=Lactobacillu	83.44	35.25	4	1	12	18	1.197
W5XES1	Aggregation promoting factor OS=Lactobacillus helv	83.21	21.40	8	6	6	16	1.217
U4QKQ3	PTS family mannose porter, IIC component OS=Lac	82.99	12.08	10	2	2	10	1.070
A0A0R2MNG2	Chromosome partitioning protein OS=Lactobacillus l	82.30	45.24	7	12	12	17	0.948
U6EZR8	UvrABC system protein B OS=Lactobacillus helveticu	82.26	15.54	9	8	9	10	1.031
A0A0D5MKT5	Iron ABC transporter ATP-binding protein OS=Lacto	81.34	39.02	5	12	12	24	0.975
A0A0R2MS90	Penicillin-binding protein OS=Lactobacillus helveticu	80.75	30.95	11	8	9	11	1.110
U4QLI0	Putative protease OS=Lactobacillus helveticus CIRM	80.65	23.88	12	7	7	15	1.070
A8YU75	3-hydroxy-3-methylglutaryl coenzyme A reductase C	80.36	28.78	5	12	12	18	0.986
A0A0N9Z904	Heme ABC transporter ATP-binding protein OS=Lact	80.33	9.51	2	1	5	12	0.885
U6F122	Uncharacterized protein OS=Lactobacillus helveticus	80.17	28.71	11	6	6	11	1.018
W5XGV2	Phosphotransferase system OS=Lactobacillus helvet	79.98	8.97	1	1	5	9	0.983
A0A0D5MGF3	Protein translocase subunit SecY OS=Lactobacillus h	78.68	11.37	3	5	5	12	1.074
A0A0P0A4H6	Methylase OS=Lactobacillus helveticus GN=ALV80_(	78.48	23.63	6	5	5	15	0.904
F3MPG8	Surface layer protein OS=Lactobacillus helveticus M	78.45	25.57	8	7	8	10	1.092
A0A0R2MSD6	tRNA pseudouridine synthase A OS=Lactobacillus he	78.36	26.17	6	6	6	10	0.988
A0A0F7H4Z5	Lipoprotein OS=Lactobacillus helveticus GN=TU99_(	77.83	17.48	8	5	5	6	1.112
A0A0F7H460	Galactosyltransferase OS=Lactobacillus helveticus G	77.75	35.25	7	1	12	19	1.112
A0A0N9ZUU4	Deoxynucleoside kinase OS=Lactobacillus helveticus	77.71	29.30	7	5	5	14	0.862
W5XLC3	Recombination protein RecR OS=Lactobacillus helve	77.70	19.47	6	4	4	8	0.984
U6F4W1	Transcription-repair-coupling factor OS=Lactobacillu	77.14	18.37	11	19	19	23	0.966

U6F7M3	ATPase OS=Lactobacillus helveticus CIRM-BIA 951 (	76.39	13.80	4	6	6	7	1.032
A0A0R2MQ70	Uncharacterized protein OS=Lactobacillus helveticus	76.35	20.82	5	5	5	8	1.010
U6F3A9	Uncharacterized protein OS=Lactobacillus helveticus	76.31	33.33	7	7	7	20	0.791
U6F3P3	Putative cell surface protein OS=Lactobacillus helvet	76.00	27.68	3	5	5	6	1.224
A0A0D5MH83	Replicative DNA helicase OS=Lactobacillus helveticu	75.53	8.84	5	4	4	5	1.131
U6F049	Acyltransferase OS=Lactobacillus helveticus CIRM-B	75.14	29.41	6	6	6	14	1.068
A0A0P0A312	50S ribosomal protein L24 OS=Lactobacillus helvetic	74.45	12.99	2	1	1	4	0.624
A0A0D5MGK4	Protease HtpX homolog OS=Lactobacillus helveticus	74.05	10.74	8	2	2	2	1.246
A8YTJ2	Putative pre-16S rRNA nuclease OS=Lactobacillus h	73.79	39.44	5	5	5	9	1.039
U6F4S0	Probable nicotinate-nucleotide adenylyltransferase (	73.61	34.82	10	9	9	28	1.030
A0A0D5MGP6	Uncharacterized protein OS=Lactobacillus helveticus	73.59	23.58	5	5	6	31	1.059
A0A0F7H6S6	Penicillin-binding protein OS=Lactobacillus helveticu	73.44	17.78	9	6	7	10	1.026
A0A0D5MIN5	GCN5 family acetyltransferase OS=Lactobacillus hel	73.39	27.33	7	5	5	8	1.154
A0A0D5MIL4	Histidine kinase OS=Lactobacillus helveticus GN=HU	73.27	9.83	10	4	4	4	1.039
A4UAB5	Putative membrane protein stomatin/prohibitin-like (	73.02	28.08	6	8	8	9	1.081
A0A0F7H3H3	Uncharacterized protein OS=Lactobacillus helveticus	72.97	27.64	4	3	3	10	0.849
U6F2T3	Penicillin-binding protein 1 OS=Lactobacillus helvetic	72.85	15.21	7	9	9	12	1.157
U6FMC5	Transcription elongation factor GreA OS=Lactobacill	72.53	34.00	6	5	5	7	0.884
U6F410	Pseudouridine synthase OS=Lactobacillus helveticus	72.31	31.02	8	9	9	13	1.005
U6FGS2	Prophage Lp1 protein 66 OS=Lactobacillus helveticu	71.96	68.75	1	1	1	2	1.166
A8YVN1	30S ribosomal protein S21 OS=Lactobacillus helvetic	71.39	53.45	2	3	3	9	1.057
A0A0D5MH02	Ribonucleoside-triphosphate reductase activating pr	71.05	21.94	4	6	6	9	0.721
U6F2V0	Putative glycosidase OS=Lactobacillus helveticus CI	70.57	27.32	10	6	6	19	1.123
A0A0D5MH46	Uncharacterized protein OS=Lactobacillus helveticus	69.97	7.55	2	1	1	4	1.903
A0A0D5MHJ7	Insertase OS=Lactobacillus helveticus GN=HUO_04	69.77	12.50	14	5	5	9	1.037
A0A0D5MII6	Uncharacterized protein OS=Lactobacillus helveticus	69.77	17.17	2	9	9	9	0.953
U6F4A2	Endonuclease III OS=Lactobacillus helveticus CIRM-	69.61	42.86	6	8	8	13	1.163
U6F5X2	Glutamine ABC superfamily ATP binding cassette tra	69.58	29.34	6	7	7	9	1.122
A0A0P0A5N8	Mano OS=Lactobacillus helveticus GN=ALV80_0881	69.04	52.46	8	7	7	10	0.895
A0A0D5MKM0	Transcriptional regulator OS=Lactobacillus helveticu	68.03	45.56	4	5	5	13	1.112
U6FFP3	Conserved protein OS=Lactobacillus helveticus CIRM	67.81	19.74	2	4	4	5	1.134
A0A0F7D0N1	Uncharacterized protein OS=Lactobacillus helveticus	67.71	28.21	7	2	2	4	1.093
A0A0F7H3I2	Proline iminopeptidase OS=Lactobacillus helveticus (	67.53	11.22	8	4	4	6	1.063
U6F259	ATP-dependent helicase/deoxyribonuclease subunit	67.48	11.29	10	12	12	14	1.072
U6F182	Phosphate import ATP-binding protein PstB OS=Lac	67.47	23.51	5	1	7	14	0.937
U6F1F6	Anaerobic ribonucleoside-triphosphate reductase OS	67.08	20.43	8	15	16	28	0.764
A0A0F7H627	Guanylate kinase OS=Lactobacillus helveticus GN=T	66.99	50.28	11	9	9	13	1.016
A8YTD4	Mini-ribonuclease 3 OS=Lactobacillus helveticus (str	66.98	27.14	5	4	4	10	0.881
A0A0N9ZAS7	Hemolysin transporter protein OS=Lactobacillus helv	66.97	28.28	6	8	8	14	0.949
U6F8D3	Uncharacterized protein OS=Lactobacillus helveticus	66.96	8.48	1	8	8	9	1.100
A8YWE6	Formamidopyrimidine-DNA glycosylase OS=Lactoba	66.78	25.00	5	8	8	14	1.048
U6F562	Amino acid ABC transporter permease protein OS=L	66.72	11.55	10	5	5	7	1.203
A0A0D5MK51	Central glycolytic genes regulator OS=Lactobacillus	66.50	20.99	8	6	6	8	1.018
A0A0R2MNM3	D-aminoacyl-tRNA deacylase OS=Lactobacillus helve	66.27	23.45	7	4	4	14	0.848
U6F4G5	Uncharacterized protein OS=Lactobacillus helveticus	66.05	8.96	1	3	3	6	1.162
A0A0D5MHS9	Phosphomevalonate kinase OS=Lactobacillus helveti	65.30	16.11	6	6	7	10	0.882
A0A0R2MRK1	2,3,4,5-tetrahydropyridine-2,6-dicarboxylate N-acety	65.10	19.15	3	5	5	12	1.121
A0A0F7CZT7	Amidase OS=Lactobacillus helveticus GN=TU99_008	65.04	19.28	19	7	7	11	0.985
A8YUA3	NAD kinase OS=Lactobacillus helveticus (strain DPC	64.69	27.04	6	8	8	19	0.923
C5J305	PheS (Fragment) OS=Lactobacillus helveticus PE=4	64.60	45.83	15	1	7	19	1.063
J3WMM8	Uncharacterized protein OS=Lactobacillus helveticus	64.51	27.78	4	2	2	8	1.145
A0A0D5MIV3	Cell division protein OS=Lactobacillus helveticus GN	64.21	20.29	4	4	4	17	1.206
A0A0D5MHX0	Tropomyosin OS=Lactobacillus helveticus GN=ALV8	63.98	33.33	3	4	4	8	1.139
U4QMJ2	Cobalt ABC transporter, ATP-binding protein OS=La	63.83	20.38	10	8	8	9	1.023
A0A0F7CZV9	Energy-coupling factor transporter transmembrane	63.71	7.92	9	2	2	4	0.980

U6F4G7	Oligopeptide ABC superfamily ATP binding cassette	63.64	25.57	6	8	8	42	1.024
A0A0N9ZA70	GntR family transcriptional regulator OS=Lactobacill	63.44	38.75	7	8	8	11	1.084
A0A0R2LSN4	Cytidine deaminase OS=Lactobacillus helveticus GN=	63.40	26.62	9	4	4	6	1.173
U6F9A8	HAD phosphatase, family IIIA OS=Lactobacillus helv	62.94	32.76	6	5	5	8	0.903
A0A0N9ZCT2	Fe-S cluster assembly protein SufB OS=Lactobacillus	62.83	13.28	6	5	5	6	0.937
U6F2T1	Phenolic acid metabolism transcriptional regulator O	62.61	35.06	8	6	6	7	1.075
A0A0D5MIW0	Ribonuclease Z OS=Lactobacillus helveticus GN=rnz	62.42	31.09	5	8	8	17	1.020
A0A0D5MGX4	ABC transporter permease OS=Lactobacillus helvetic	62.34	15.38	11	8	8	10	1.075
A0A0D5MK80	UDP-phosphate N-acetyl-glucosaminyl transferase O	61.80	11.17	7	3	3	7	0.886
A0A0D5MIX5	Arsenate reductase OS=Lactobacillus helveticus GN=	61.72	15.25	5	2	2	7	1.167
U6F4R5	Uncharacterized protein OS=Lactobacillus helveticus	61.66	28.86	7	4	5	9	0.955
A8YTX2	Putative phosphoglycerate mutase OS=Lactobacillus	61.42	20.00	20	8	9	11	0.992
U6F7F9	Uncharacterized protein OS=Lactobacillus helveticus	61.32	32.39	11	7	7	15	0.963
A0A0D5MHG0	30S ribosomal protein S14 type Z OS=Lactobacillus	61.20	29.51	4	2	3	18	0.968
A0A0D5MK40	Hemolysin OS=Lactobacillus helveticus GN=HUO_08	61.16	18.10	10	7	7	11	1.096
A0A0R2MQT3	Phosphate import ATP-binding protein PstB OS=Lac	60.47	21.51	5	1	7	13	0.945
A8YWA5	tRNA dimethylallyltransferase OS=Lactobacillus helv	60.10	43.14	6	13	13	17	1.058
U6F863	Uncharacterized protein OS=Lactobacillus helveticus	59.58	36.56	12	7	7	13	1.007
A0A0D5MGD8	Glycosyl transferase OS=Lactobacillus helveticus GN	58.95	19.06	5	5	5	9	0.986
A0A0D5MHT8	ADP-dependent (S)-NAD(P)H-hydrate dehydratase (	58.93	16.67	7	4	4	7	1.063
U6F2W9	Riboflavin biosynthesis protein OS=Lactobacillus hel	58.65	20.95	12	6	6	6	1.025
A0A0R2MME0	Mannosyltransferase och1 related enzyme OS=Lacto	57.66	13.96	6	3	3	5	0.997
A0A0D5MKY1	Peptide ABC transporter ATP-binding protein OS=La	57.56	38.39	6	6	6	10	0.997
U6F2U5	Uncharacterized protein OS=Lactobacillus helveticus	57.32	32.00	3	2	2	2	1.000
A0A0N9ZG54	Methyltransferase OS=Lactobacillus helveticus GN=/	57.27	13.25	11	3	3	5	1.227
U6F1L7	Raffinose operon transcriptional regulatory protein F	57.26	29.93	9	7	8	11	0.978
W5XCN3	Uncharacterized protein OS=Lactobacillus helveticus	57.05	26.02	9	8	8	13	1.182
A0A0D5MGH2	2', 3'-cyclic nucleotide 2'-phosphodiesterase OS=Lac	56.88	41.80	4	13	13	29	0.989
A0A0N9ZA79	Beta-carotene 15,15'-monooxygenase OS=Lactobac	56.73	7.09	7	2	2	3	0.981
U6F2U9	Exopolysaccharide biosynthesis protein OS=Lactoba	56.20	21.09	12	5	5	7	1.047
A0A0F7H545	Membrane protein OS=Lactobacillus helveticus GN=	55.94	3.68	3	1	1	2	0.917
A0A0D5MG95	Guanine permease OS=Lactobacillus helveticus GN=	55.46	5.42	10	2	2	5	1.031
A0A0D5MG36	Antitoxin OS=Lactobacillus helveticus GN=ALV80_1(	55.40	46.08	2	4	4	5	0.978
A0A0D5MHD6	tRNA-dihydrouridine synthase OS=Lactobacillus helv	55.34	23.46	10	9	9	12	0.899
F3MLH1	Glycerol-3-phosphate acyltransferase OS=Lactobacil	55.22	9.05	8	2	2	2	1.011
A0A0D5MJX2	Cys-tRNA(Pro)/Cys-tRNA(Cys) deacylase OS=Lactob	54.61	18.34	7	4	4	6	0.908
U6FCG3	Amino acid ABC transporter, permease protein OS=	54.33	17.49	10	4	4	14	1.202
A0A0D5MG49	Ammonium transporter OS=Lactobacillus helveticus	54.23	5.71	9	2	2	5	1.024
U6F2F0	Protein-tyrosine phosphatase OS=Lactobacillus helv	53.76	47.15	8	11	11	18	1.006
A0A0D5MK61	Cold-shock protein OS=Lactobacillus helveticus GN=	53.76	13.43	1	1	1	2	0.953
A0A0D5MI67	CDP-diglyceride synthetase OS=Lactobacillus helvet	53.61	9.23	3	3	3	7	1.065
A8YUD8	Putative sporulation transcription regulator WhiA OS	53.51	27.33	2	8	8	11	0.997
A0A0D5MFQ9	Spermidine/putrescine ABC transporter ATP-binding	53.03	15.57	15	3	3	3	1.060
A0A0R2MPE4	Uncharacterized protein OS=Lactobacillus helveticus	53.01	11.73	9	5	5	8	1.102
A0A0D5MFX1	Putative 3-methyladenine DNA glycosylase OS=Lact	52.79	20.48	12	4	4	6	1.078
F3ML57	Uncharacterized protein OS=Lactobacillus helveticus	52.70	18.60	8	2	2	2	1.122
A0A0D5MJ51	Excinuclease ABC subunit A OS=Lactobacillus helvet	52.47	9.71	13	8	8	9	1.045
A0A0D5MG37	Cobalamin biosynthesis protein CobQ OS=Lactobaci	52.21	20.61	5	5	5	9	1.030
A0A0D5MJJ2	Phosphohydrolase OS=Lactobacillus helveticus GN=	52.06	30.34	3	4	4	7	0.995
A0A0D5MG24	Uncharacterized protein OS=Lactobacillus helveticus	51.89	15.26	6	5	5	9	1.042
A0A0D5MJ10	CCA-adding enzyme OS=Lactobacillus helveticus GN	51.76	30.83	8	12	12	22	0.881
U6F2Z0	Type I restriction enzyme, S subunit OS=Lactobacill	51.73	13.37	1	7	7	10	1.016
A0A0D5MIC7	Uracil-DNA glycosylase OS=Lactobacillus helveticus	50.95	41.55	11	8	8	12	1.029
A0A0R2MDT9	Uncharacterized protein OS=Lactobacillus helveticus	50.59	13.39	4	3	3	5	1.049
A0A0R2MMR0	XRE family transcriptional regulator OS=Lactobacillu	50.47	70.94	7	8	8	23	1.172

A0A0N7HHH8	DNA repair protein OS=Lactobacillus helveticus GN=	49.93	13.57	10	11	11	12	0.970
A0A0F7H600	Pantothenate kinase OS=Lactobacillus helveticus GN	49.53	24.34	9	7	7	15	0.955
A8YTI0	DNA mismatch repair protein MutL OS=Lactobacillus	49.53	11.64	9	7	7	8	0.881
A0A0D5MJN0	Amino acid permease OS=Lactobacillus helveticus G	49.39	7.04	8	5	5	9	1.045
A0A0D5MGM8	Holliday junction ATP-dependent DNA helicase RuvE	49.25	13.61	8	4	4	5	0.891
A0A0D5MFU8	Uncharacterized protein OS=Lactobacillus helveticus	48.19	23.27	9	6	6	8	1.069
U6F7Y7	Uncharacterized protein OS=Lactobacillus helveticus	48.13	14.49	10	3	3	4	0.904
U6F6Y7	ORF 221 OS=Lactobacillus helveticus CIRM-BIA 951	48.00	34.45	1	7	7	13	1.039
U6EZZ8	Diaminopimelate epimerase OS=Lactobacillus helvet	47.42	6.59	3	2	2	4	1.020
A0A0N7HHM2	Adenylate kinase OS=Lactobacillus helveticus GN=A	47.23	12.27	8	2	2	4	0.984
A0A0D5MHH8	Transposase OS=Lactobacillus helveticus GN=HUO_	46.65	7.02	32	3	3	4	0.920
A0A0R2MDR6	Abc-type spermidine putrescine transport system pe	46.62	6.30	6	1	1	1	1.132
U6F514	Glycerol uptake facilitator protein OS=Lactobacillus	46.61	7.20	1	1	1	2	1.059
A0A0D5MKH2	Arylesterase OS=Lactobacillus helveticus GN=HUO_	46.25	27.42	7	4	4	6	1.062
A0A0F7H377	Helicase OS=Lactobacillus helveticus GN=TU99_003	46.19	3.82	10	4	4	4	1.016
A0A0D5MJ42	Peptide-binding protein OS=Lactobacillus helveticus	45.23	7.85	8	2	2	2	1.076
A0A0F7H3Z9	Amidohydrolase OS=Lactobacillus helveticus GN=TU	44.71	9.62	9	3	3	4	1.245
A0A0D5MGG9	Uroporphyrin-III C-methyltransferase OS=Lactobaci	44.67	57.50	5	6	6	14	1.294
A0A0D5MJ22	Uncharacterized protein OS=Lactobacillus helveticus	44.31	9.45	9	7	7	13	1.026
A0A0N7HHL4	Metal ABC transporter ATPase OS=Lactobacillus helv	44.00	3.17	11	2	2	3	1.088
A0A0F7H387	ADP-ribose pyrophosphatase OS=Lactobacillus helve	43.85	16.67	6	4	4	4	1.115
A0A0D5MJ11	ATPase OS=Lactobacillus helveticus GN=ALV80_075	43.78	44.58	4	10	10	22	0.983
A0A0F7D060	Methionine import ATP-binding protein MetN OS=La	43.73	17.85	14	7	7	8	1.055
F0NVM8	Glycosyl transferase, group 1 OS=Lactobacillus helv	43.09	33.82	6	9	10	14	1.080
A0A0P0A7I4	Ribosomal RNA large subunit methyltransferase H C	43.07	31.45	8	6	6	9	0.919
A0A0R2M1E6	Uncharacterized protein OS=Lactobacillus helveticus	42.67	11.46	5	2	2	5	0.912
A0A0D5MFX5	Uncharacterized protein OS=Lactobacillus helveticus	42.67	7.55	4	1	1	1	1.227
A8YVR4	Zinc metalloprotease OS=Lactobacillus helveticus (st	42.55	7.89	9	3	3	4	1.029
A0A0R2MRV5	Aspartokinase OS=Lactobacillus helveticus GN=IV62	42.38	7.59	1	1	2	4	1.108
J7LNK8	Major facilitator superfamily permease OS=Lactobac	42.36	9.47	7	3	3	8	1.139
U6F1Z4	Uncharacterized protein OS=Lactobacillus helveticus	41.86	23.97	7	3	3	4	0.988
A0A0D5MJL6	DNA polymerase III subunit delta OS=Lactobacillus	41.59	14.33	3	4	5	5	1.002
A0A0D5MI25	Uncharacterized protein OS=Lactobacillus helveticus	41.49	35.17	4	6	6	14	1.045
A0A0F7H4F2	CDP-diacylglycerol--glycerol-3-phosphate 3-phospha	41.26	20.43	4	5	5	13	0.939
A0A0D5MKJ3	DNA polymerase III alpha chain OS=Lactobacillus h	41.05	21.47	4	3	3	4	1.155
A0A0N9ZHZ4	GNAT family acetyltransferase OS=Lactobacillus helv	40.92	7.22	10	4	4	5	1.125
A0A0D5MGX6	Phosphatase OS=Lactobacillus helveticus GN=HUO_	40.87	15.95	9	5	5	6	1.048
A0A0R2MUE3	CorA family cationic transporter OS=Lactobacillus he	40.54	16.25	8	4	5	6	1.121
A0A0R2MNN1	Reductase OS=Lactobacillus helveticus GN=IV62_GI	40.32	60.75	3	7	7	12	0.949
A0A0F7H254	Probable potassium transport system protein kup O!	40.21	7.43	18	3	4	5	1.211
Q48567	Orf protein OS=Lactobacillus helveticus GN=orf PE=	40.05	20.39	2	3	3	5	1.020
F3MLP9	Uncharacterized protein OS=Lactobacillus helveticus	39.99	50.00	1	4	4	11	0.506
U6EZV7	Energy-coupling factor transporter ATP-binding prot	39.94	23.51	8	6	6	7	0.998
A0A0D5MGR2	Glycerol phosphate lipoteichoic acid synthase OS=La	39.64	5.26	7	3	4	6	1.019
A0A0D5MKJ6	Uncharacterized protein OS=Lactobacillus helveticus	39.21	11.56	2	2	2	4	1.111
U6FSZ6	Virulence protein OS=Lactobacillus helveticus CIRM-	39.16	19.78	6	1	1	1	1.004
A0A0R2MSH1	Acyltransferase family protein OS=Lactobacillus helv	39.01	5.42	11	3	3	4	0.947
U6F6I1	Putative acyl-coa thioesterase OS=Lactobacillus helv	39.00	17.24	11	3	3	3	1.063
F0NTC0	Segregation and condensation protein B OS=Lactob	38.84	26.47	6	5	5	12	1.040
U6F732	Putative oxidoreductase OS=Lactobacillus helveticus	38.84	24.77	5	6	6	8	0.916
F3MPU6	2,3-bisphosphoglycerate-dependent phosphoglycera	38.68	44.15	6	7	7	12	1.037
A0A0P0A2L3	Uncharacterized protein OS=Lactobacillus helveticus	38.04	16.53	8	3	3	4	1.128
A0A0N9ZAF4	Carboxypeptidase OS=Lactobacillus helveticus GN=/	38.03	9.34	10	6	6	6	1.148
A0A0D5MHG9	XRE family transcriptional regulator OS=Lactobacillu	38.00	37.50	4	5	5	9	1.271
F0NUC4	Oxidoreductase family, NAD-binding Rossmann fold	37.63	10.58	9	3	3	4	0.935

A0A0F7D0A6	Signal peptidase I OS=Lactobacillus helveticus GN=T	37.63	13.76	7	3	3	4	1.144
U6FME3	Glycerophosphodiester phosphodiesterase OS=Lacto	37.48	3.35	6	1	1	1	0.976
F3MMZ9	Cell wall-associated hydrolase OS=Lactobacillus helv	37.22	26.13	16	9	9	16	1.214
A0A0P0A3Z2	Copper-transporting ATPase OS=Lactobacillus helve	37.17	4.13	7	1	2	2	1.195
A0A0D5MHS0	Acylphosphatase OS=Lactobacillus helveticus GN=H	36.79	28.89	5	2	2	2	1.212
A0A0D5MJD0	Phosphopantetheine adenylyltransferase OS=Lactob	36.74	15.85	3	3	3	7	0.983
A8YVV9	Serine-threonine protein kinase OS=Lactobacillus he	36.41	5.41	10	3	3	3	0.981
A0A0D5ML55	Pyridoxal kinase OS=Lactobacillus helveticus GN=HU	36.27	10.62	12	3	3	4	0.985
J3XAV7	Cation-transporting P-type ATPase OS=Lactobacillus	36.20	1.57	2	1	2	8	0.764
A0A0N9ZSI3	Uncharacterized protein OS=Lactobacillus helveticus	35.87	10.66	1	1	1	1	0.971
A0A0D5MKK8	D-alanine/D-serine/glycine permease OS=Lactobacil	35.80	8.10	10	6	6	8	1.083
J3XAR4	Uncharacterized protein OS=Lactobacillus helveticus	35.62	41.67	5	3	3	5	0.966
A0A0N9ZAY8	Glutamine ABC transporter permease OS=Lactobaci	35.25	10.60	6	2	2	3	1.271
A0A0D5ML27	Sugar ABC transporter permease OS=Lactobacillus l	35.22	6.94	6	1	1	1	0.988
A0A0N9ZBX6	N-acetylmuramidase OS=Lactobacillus helveticus GN	34.78	17.42	8	2	3	3	0.869
A0A0D5MGX5	Restriction endonuclease subunit R OS=Lactobacillu	34.74	9.78	3	6	6	6	0.936
A8YTR0	50S ribosomal protein L34 OS=Lactobacillus helvetic	34.51	28.26	1	2	2	13	0.794
A0A0D5MKV0	Multidrug ABC transporter ATP-binding protein OS=	34.50	1.56	12	1	1	1	0.954
A0A0D5MKY4	Glycerol-3-phosphate cytidyltransferase OS=Lactoba	34.49	41.41	2	5	5	8	0.944
W5XFF7	GntR family transcriptional regulator OS=Lactobacill	34.36	25.25	5	2	2	2	1.047
A0A0R2MLL8	Membrane protein insertase YidC OS=Lactobacillus	34.21	7.22	11	2	2	6	1.021
U6F3Y5	Putative cellwall teichoic acid glycosylation protein C	34.12	11.17	8	2	2	2	1.039
A0A0F7H2A9	Uncharacterized protein OS=Lactobacillus helveticus	34.04	10.53	6	1	1	4	1.078
A0A0D5MJE4	Endonuclease III OS=Lactobacillus helveticus GN=n	33.82	16.99	8	4	5	9	1.062
A0A0D5MIF9	ATPase P OS=Lactobacillus helveticus GN=HUO_07	33.67	3.35	10	3	3	4	1.062
F3MM83	Sugar ABC transporter permease protein OS=Lactob	33.57	4.70	8	2	2	3	0.997
A0A0F7H492	50S ribosomal protein L28 OS=Lactobacillus helvetic	33.52	72.13	3	8	8	40	0.637
A8YXC7	ABC transporter OS=Lactobacillus helveticus (strain	33.47	5.52	15	3	3	4	0.903
A0A0F7H667	TetR family transcriptional regulator OS=Lactobacillu	33.07	19.32	9	4	4	5	0.933
A0A0D5MJQ9	Regulatory protein Spx OS=Lactobacillus helveticus	32.82	43.94	6	6	6	10	0.983
A0A0D5MJC9	Uncharacterized protein OS=Lactobacillus helveticus	32.81	71.43	2	3	3	3	1.124
U6F838	Uncharacterized protein OS=Lactobacillus helveticus	32.69	42.24	9	7	7	12	1.127
A0A0C5KQ31	Excision endonuclease subunit (Fragment) OS=Lacto	32.65	16.56	21	3	3	3	1.030
U6F5J1	Uncharacterized protein OS=Lactobacillus helveticus	32.43	12.07	1	1	1	2	1.093
F3MQM0	Small-conductance mechanosensitive channel OS=L	32.27	7.72	8	2	2	2	0.914
A0A0D5MGU6	23S rRNA pseudouridylate synthase OS=Lactobacillu	32.25	9.12	6	2	2	2	1.036
A0A0N9ZGL7	tRNA(Ile)-lysidine synthase OS=Lactobacillus helveti	32.11	6.22	8	3	3	3	1.040
A0A0F7CZS3	Uncharacterized protein OS=Lactobacillus helveticus	32.06	13.14	7	4	4	4	1.040
U6F4Q8	EpsI OS=Lactobacillus helveticus CIRM-BIA 951 GN=	31.61	4.33	1	1	2	6	0.886
A0A0D5MKF0	Beta-lactamase OS=Lactobacillus helveticus GN=HU	31.52	23.10	10	8	8	14	1.168
A8YWK7	Putative histidine kinase OS=Lactobacillus helveticus	31.46	5.99	12	4	4	5	1.107
A0A0R2MEI5	Phosphotransferase enzyme iia component OS=Lact	31.39	7.61	11	2	2	2	0.859
A8YVT8	Amino acid transporter OS=Lactobacillus helveticus	30.92	3.41	10	2	2	4	1.162
A0A0F7H433	Uracil permease OS=Lactobacillus helveticus GN=TU	30.56	7.01	11	4	4	7	0.896
A0A0D5MLG1	Uncharacterized protein OS=Lactobacillus helveticus	30.56	17.43	8	2	2	2	1.084
J3X9S2	Toxin-antitoxin system OS=Lactobacillus helveticus	30.51	21.84	2	1	2	15	1.053
A0A0F7H5U5	DNA replication initiation protein OS=Lactobacillus h	30.21	9.16	12	3	3	3	1.107
A0A0D5MGA0	Metal ABC transporter ATPase OS=Lactobacillus helv	30.06	6.09	14	5	5	5	0.997
A0A0D5MJP1	Cell division protein SepF OS=Lactobacillus helveticu	30.02	35.86	4	7	7	13	0.918
A0A0N9Z9X0	Uncharacterized protein OS=Lactobacillus helveticus	30.01	8.54	10	2	2	5	0.764
A0A0D5MKJ5	Uncharacterized protein OS=Lactobacillus helveticus	29.59	45.45	2	4	4	7	1.341
A0A0D5MHJ6	Preprotein translocase subunit SecE OS=Lactobacillu	28.64	27.27	2	2	2	8	1.062
A8YV38	Putative membrane protein OS=Lactobacillus helvet	28.33	25.76	8	9	10	14	1.042
A0A0D5MIH8	DNA primase OS=Lactobacillus helveticus GN=dnaG	28.30	8.02	10	5	5	5	1.004
A0A0D5MGY4	Dephospho-CoA kinase OS=Lactobacillus helveticus	28.11	6.00	3	1	1	1	1.152

A0A0R2MTZ6	Uncharacterized protein OS=Lactobacillus helveticus	27.80	11.35	8	2	2	3	1.032
A0A0F7H1Y0	Membrane protein OS=Lactobacillus helveticus GN=	27.43	8.14	9	3	3	3	1.030
A0A0N9ZGU6	Uncharacterized protein OS=Lactobacillus helveticus	27.07	20.25	5	2	2	4	1.062
A0A0D5MGS8	Phospho-beta-glycosidase OS=Lactobacillus helvetic	26.94	12.46	14	3	4	5	0.865
A0A0D5MKT8	Probable potassium transport system protein kup O!	26.82	2.83	11	1	2	2	1.079
B3EYD4	ABC3ATP3 OS=Lactobacillus helveticus CNRZ32 PE=	26.82	5.53	3	1	2	5	1.019
F0NWY5	ABC superfamily ATP binding cassette transporter p	26.56	12.32	10	6	6	8	1.112
A0A0F7H472	Uncharacterized protein OS=Lactobacillus helveticus	24.22	4.17	10	1	2	4	0.960
A0A0R2MA06	Transporter-membrane protein OS=Lactobacillus he	23.92	2.47	9	1	1	1	1.152
F0NSJ2	DNA repair protein RecO OS=Lactobacillus helveticu	22.28	3.51	7	1	1	1	1.086
A0A0D5MHD1	RNA-binding protein OS=Lactobacillus helveticus GN	22.00	31.67	6	4	4	4	1.064
U6F4N3	Uncharacterized protein OS=Lactobacillus helveticus	21.41	27.49	5	5	5	8	0.899
A0A0N9Z7G9	Enolase OS=Lactobacillus helveticus GN=eno PE=3	20.64	8.88	4	3	3	3	1.026
A0A0P0A588	Glucan phosphorylase OS=Lactobacillus helveticus G	20.64	0.75	2	1	1	2	0.910
A0A0D5MGY0	Membrane protein OS=Lactobacillus helveticus GN=	19.50	5.04	10	3	3	4	1.078
U6F9K9	Uncharacterized protein OS=Lactobacillus helveticus	19.43	6.36	1	2	2	4	1.057
U6F2Q7	Putative surface exclusion protein OS=Lactobacillus	17.95	9.79	10	3	3	3	1.093
A0A0D5MH32	Septation inhibitor protein OS=Lactobacillus helvetic	17.62	9.60	6	1	2	4	1.105
A0A0D5MKH8	Uncharacterized protein OS=Lactobacillus helveticus	15.31	13.48	10	6	6	7	1.149
A8YXG4	Xanthine phosphoribosyltransferase OS=Lactobacillu	0.00	17.19	6	3	3	5	0.948
A8YTD9	50S ribosomal protein L33 2 OS=Lactobacillus helve	0.00	48.98	1	3	3	7	0.921
D6QTD5	60 kDa chaperonin OS=Bifidobacterium longum sub	0.00	1.66	1	1	1	1	1.871
M4SQU3	Phage coat protein OS=Cyanophage KBS-S-2A GN=	0.00	2.34	1	1	1	1	1.029
L0P8M2	Putative phage major capsid protein OS=Lactobacill	0.00	1.96	3	1	1	1	0.906
F3MRJ5	Uncharacterized protein OS=Lactobacillus helveticus	0.00	11.07	9	2	2	4	1.078
A0A0R2MMC1	N-acetylmuramidase OS=Lactobacillus helveticus GN	0.00	5.66	7	1	1	1	1.068
A0A0F7H5J4	Methyltransferase OS=Lactobacillus helveticus GN='	0.00	5.54	7	2	2	2	0.984
F0NUZ8	Cation-transporting ATPase PacL OS=Lactobacillus h	0.00	2.12	9	2	2	2	1.100
F3MNC9	Pyrimidine-nucleoside phosphorylase OS=Lactobacil	0.00	5.81	1	1	1	1	0.875
Q8RQV6	Xaa-Pro dipeptidyl-peptidase OS=Lactobacillus helve	0.00	1.87	1	1	1	1	1.042
A0A0F7H205	Uncharacterized protein OS=Lactobacillus helveticus	0.00	5.12	10	2	2	2	0.971
U6F5L7	Bacteriophage integrase OS=Lactobacillus helveticus	0.00	7.07	2	2	2	2	0.890
U4QAC8	6-phospho-beta-glucosidase OS=Lactobacillus helve	0.00	2.46	8	1	1	1	1.076
U6F341	Transcriptional regulator OS=Lactobacillus helveticu	0.00	7.56	3	1	1	2	1.165
F3MNW0	Integrase (Fragment) OS=Lactobacillus helveticus M	0.00	7.57	1	1	1	1	1.276
A0A0D5MGQ7	Membrane protein OS=Lactobacillus helveticus GN=	0.00	6.94	4	1	2	2	0.907
A8YW16	Type I restriction modification system OS=Lactobac	0.00	0.93	6	1	1	1	1.247
A0A0D5MHY8	Thiamine pyrophosphokinase OS=Lactobacillus helve	0.00	4.82	6	2	2	2	1.201
A0A0R2MR56	Uncharacterized protein OS=Lactobacillus helveticus	0.00	1.42	2	1	1	1	1.233
A0A0D5MK68	Bacitracin ABC transporter ATP-binding protein OS=	0.00	10.04	6	2	2	5	0.821
A0A0P0A7C5	DNA invertase OS=Lactobacillus helveticus GN=ALV	0.00	8.70	2	1	1	1	0.567
U6FQS9	Elongation factor EF2 OS=Lactobacillus helveticus C	0.00	1.01	7	1	1	1	0.974
A0A0D5MKF1	Multidrug ABC transporter permease OS=Lactobacill	0.00	2.55	11	2	2	2	0.988
A0A0R2MR07	Uncharacterized protein OS=Lactobacillus helveticus	0.00	4.10	7	1	1	1	1.048
F0NVZ5	Phosphatidylserine decarboxylase OS=Lactobacillus	0.00	2.05	2	1	1	1	0.900
U4QIS8	Uncharacterized protein OS=Lactobacillus helveticus	0.00	2.30	1	1	1	3	1.258
A0A0P0A496	MFS transporter OS=Lactobacillus helveticus GN=AL	0.00	1.23	2	1	1	1	0.905
A0A0R2MQC4	Cell division protein DivIB OS=Lactobacillus helvetic	0.00	3.96	7	1	1	1	1.056
U6FB35	TYPE III RESTRICTION-MODIFICATION SYSTEM RE	0.00	0.50	1	1	1	1	1.192
U4QIQ5	Uncharacterized protein OS=Lactobacillus helveticus	0.00	3.15	1	1	1	1	0.714
A0A0R2MI70	Spermidine and putrescine periplasmatic abc transp	0.00	4.65	7	2	2	3	1.036
A0A0D5MKX7	Transposase OS=Lactobacillus helveticus GN=HUO_	0.00	9.18	24	1	2	4	0.675
A8YTM7	Uncharacterized protein OS=Lactobacillus helveticus	0.00	5.98	8	1	1	2	0.916
F3ML00	Putative integrase-recombinase OS=Lactobacillus he	0.00	18.40	7	4	4	4	1.124
U4QNE9	Putative permease protein OS=Lactobacillus helvetic	0.00	1.87	1	1	1	1	0.926

U6FRQ7	Uncharacterized protein OS=Lactobacillus helveticus	0.00	3.74	1	1	1	8	0.768
F3MQ83	Gluconate kinase OS=Lactobacillus helveticus MTCC	0.00	2.98	10	1	1	1	0.761
U6F522	Uncharacterized protein OS=Lactobacillus helveticus	0.00	8.85	1	4	4	4	0.968
U6FJL9	Uncharacterized protein OS=Lactobacillus helveticus	0.00	1.85	7	1	1	1	1.081
U6F5K3	Eps4F OS=Lactobacillus helveticus CIRM-BIA 951 GI	0.00	4.88	1	1	2	4	0.957
W5XPC1	Uncharacterized protein OS=Lactobacillus helveticus	0.00	6.88	1	1	2	4	1.076
A0A0R2MVH6	Transcriptional regulator MraZ OS=Lactobacillus hel	0.00	24.56	5	3	3	5	1.086
F3MKF4	Phospho-N-acetylmuramoyl-pentapeptide-transferas	0.00	3.79	8	1	1	1	1.462
J3ZDS2	Uncharacterized protein OS=Lactobacillus helveticus	0.00	7.84	11	2	2	2	1.080
F3MRB5	Transposase OS=Lactobacillus helveticus MTCC 546	0.00	8.99	1	1	1	1	1.010
A0A0R2MM41	Uncharacterized protein OS=Lactobacillus helveticus	0.00	4.12	6	1	1	1	1.168
U6FEW3	Uncharacterized protein OS=Lactobacillus helveticus	0.00	1.44	2	1	1	1	1.062
U6F4R7	Eps3J OS=Lactobacillus helveticus CIRM-BIA 951 GN	0.00	2.72	1	1	1	1	1.024
J7LNA3	Transposase OS=Lactobacillus helveticus R0052 GN	0.00	10.34	1	1	1	1	1.211
A0A0N9ZHL9	Uncharacterized protein OS=Lactobacillus helveticus	0.00	1.39	2	1	1	1	0.897
A0A0D5MI69	ATP-dependent DNA helicase RecG OS=Lactobacillu	0.00	1.63	4	1	1	1	0.811
A0A0F7H1W2	Uncharacterized protein OS=Lactobacillus helveticus	0.00	10.40	9	7	7	8	1.068
U6F5V1	Muramidase OS=Lactobacillus helveticus CIRM-BIA	0.00	6.08	1	1	1	2	1.046
F3MPV8	Uncharacterized protein OS=Lactobacillus helveticus	0.00	3.71	7	1	2	6	0.979
A0A0R2MPG0	Signal peptidase I OS=Lactobacillus helveticus GN=	0.00	17.37	4	5	5	11	1.069
U6FCU4	Putative Type II restriction enzyme, methylase subu	0.00	4.11	1	1	1	2	0.946
U6FEK5	Uncharacterized protein OS=Lactobacillus helveticus	0.00	9.62	12	6	6	10	1.040
J4BUM1	Uncharacterized protein OS=Lactobacillus helveticus	0.00	5.66	3	1	1	1	1.217
J4BQ46	Uncharacterized protein OS=Lactobacillus helveticus	0.00	9.52	9	3	3	3	0.846
A0A0F7H2S5	DNA replication and repair protein RecF OS=Lactoba	0.00	8.27	5	2	2	2	0.978
U6F5N5	Single-stranded DNA-binding protein OS=Lactobacill	0.00	6.38	2	1	1	1	1.243
A0A0R2MH94	Uncharacterized protein OS=Lactobacillus helveticus	0.00	8.67	7	1	1	1	0.885
A0A0P0A4Z8	DeoR faimly transcriptional regulator OS=Lactobacil	0.00	10.40	5	2	2	3	1.287
A0A0D5MI38	Histidine kinase OS=Lactobacillus helveticus GN=AL	0.00	8.33	3	1	1	1	1.064
A0A0F7H452	Uncharacterized protein OS=Lactobacillus helveticus	0.00	9.49	1	1	1	2	0.825
A0A0D5MJP8	Cell division protein FtsK OS=Lactobacillus helveticu	0.00	2.11	9	2	2	3	1.067
U6F8G7	Alpha/beta hydrolase superfamily enzyme OS=Lacto	0.00	7.80	3	1	1	3	1.066
A0A0R2MKL8	Amino acid ABC transporter permease OS=Lactobac	0.00	5.04	5	1	1	2	1.083
J7LIB0	Alpha amylase, catalytic domain protein OS=Lactoba	0.00	3.98	5	1	1	2	0.705
J4BPI7	FAD binding domain protein OS=Lactobacillus helve	0.00	2.74	1	1	1	1	1.079
A0A0F7H3B4	Phosphate ABC transporter permease OS=Lactobaci	0.00	8.89	3	1	1	1	0.633
A0A0D5MJ59	RNA-binding protein OS=Lactobacillus helveticus GN	0.00	12.78	6	3	3	3	0.992
A0A0R2MRA6	DASS family divalent anion sodium (Na+) symporter	0.00	3.12	8	1	1	1	0.977
W5XGR7	Alpha(1,2)galactosyltransferase EpsI OS=Lactobacill	0.00	2.67	1	1	1	1	0.895
A0A0D5MI20	Transcriptional regulator OS=Lactobacillus helveticu	0.00	3.55	3	1	1	1	1.144
A0A0N9ZD44	ABC transporter OS=Lactobacillus helveticus GN=AL	0.00	4.68	10	2	2	2	0.943
A0A0D5MJP9	Membrane protein OS=Lactobacillus helveticus GN=	0.00	3.19	10	2	2	2	1.103
U6F6E5	Essential recombination function protein OS=Lactob	0.00	4.55	1	1	1	1	1.057
U4QC43	Replication-associated protein RepA OS=Lactobacillu	0.00	8.62	1	1	1	2	1.050
U6F4T4	Uncharacterized protein OS=Lactobacillus helveticus	0.00	9.48	1	1	1	1	1.105
U6F3B7	Structural protein OS=Lactobacillus helveticus CIRM	0.00	9.34	1	2	2	2	1.216
F3MQ40	Uncharacterized protein OS=Lactobacillus helveticus	0.00	4.44	1	1	1	4	1.070
A8YVI1	Phage integrase-recombinase OS=Lactobacillus helv	0.00	9.30	6	3	3	3	1.018
A0A0N9ZZD2	Esterase OS=Lactobacillus helveticus GN=ALV80_07	0.00	5.93	5	2	3	4	1.085
U6F7N2	Putative transcriptional regulator OS=Lactobacillus h	0.00	4.83	1	1	1	1	1.082
A0A0R2MQ86	Aluminum (Al2+) resistance protein OS=Lactobacillu	0.00	3.69	2	1	1	1	0.483
U4QG46	Alpha/beta hydrolase superfamily protein OS=Lactol	0.00	15.30	12	3	3	4	1.083
F3MNX1	Uncharacterized protein OS=Lactobacillus helveticus	0.00	4.80	6	1	1	1	1.101
U6F729	Recombination protein J OS=Lactobacillus helveticus	0.00	6.21	15	4	5	5	0.905
A0A0R2M0G7	Hexulose OS=Lactobacillus helveticus GN=IV62_GL(	0.00	2.28	4	1	1	1	0.923

U6F8W5	Recombinase for Bh.Int OS=Lactobacillus helveticus	0.00	10.58	3	1	2	5	0.724
A0A0N9ZXJ1	Chromosome replication initiation protein OS=Lactol	0.00	2.48	5	1	1	1	1.445
U6F4F9	Integrase OS=Lactobacillus helveticus CIRM-BIA 95	0.00	1.49	1	1	1	4	0.877
F3MM24	Mobile genetic element (Fragment) OS=Lactobacillu	0.00	29.47	30	4	4	4	0.971
A0A0N9ZAC3	Acetyltransferase OS=Lactobacillus helveticus GN=A	0.00	6.98	3	1	1	1	1.068
U6F3T0	Uncharacterized protein OS=Lactobacillus helveticus	0.00	8.92	5	3	3	4	0.957
A0A0D5MH57	ATP synthase F0 subunit A OS=Lactobacillus helveti	0.00	1.16	5	1	1	1	1.247
U6FQ93	DNA topoisomerase OS=Lactobacillus helveticus CIF	0.00	1.13	1	1	1	2	1.157
A0A0D5MH77	Cobalt transporter OS=Lactobacillus helveticus GN=	0.00	5.35	6	1	1	1	1.150
A0A0N7HHH0	Metal ABC transporter substrate-binding protein OS=	0.00	6.42	8	1	1	1	0.930
U6F203	Uncharacterized protein OS=Lactobacillus helveticus	0.00	6.76	9	2	2	3	1.085
U6F9C5	Glutamine ABC superfamily ATP binding cassette tra	0.00	11.27	8	2	2	6	1.021
A0A0P0A2Y7	Phosphoribosylformylglycinamidine cyclo-ligase OS=	0.00	1.74	4	1	1	1	1.039
A0A0P0A543	ATP synthase subunit c OS=Lactobacillus helveticus	0.00	17.57	6	2	2	4	0.848
A0A0R2M9Y9	Putative fluoride ion transporter CrcB OS=Lactobacil	0.00	9.09	7	1	1	2	1.079
A0A0D5MH52	Uracil phosphoribosyltransferase OS=Lactobacillus h	0.00	4.11	7	1	1	2	1.115
A0A0D5MHA4	Phosphate transport system permease protein PstA	0.00	7.12	4	2	2	2	1.135
A8YW17	Uncharacterized protein OS=Lactobacillus helveticus	0.00	2.90	8	2	2	2	1.052
U6F8X5	Uncharacterized protein OS=Lactobacillus helveticus	0.00	6.40	1	1	1	1	1.009
A0A0D5MK00	Uncharacterized protein OS=Lactobacillus helveticus	0.00	2.43	9	1	1	1	0.983
A8YV42	UPF0346 protein lhv_1069 OS=Lactobacillus helvetic	0.00	38.16	1	2	2	2	1.059
F3MPT2	ABC transporter, ATP-binding/permease protein OS=	0.00	12.50	5	1	1	1	1.160
A0A0R2MTZ5	Citrate lyase regulator OS=Lactobacillus helveticus C	0.00	1.60	4	1	1	1	1.094
Q8VU20	S-layer protein (Fragment) OS=Lactobacillus helveti	0.00	13.48	3	1	2	2	0.938
U6F346	Uncharacterized protein OS=Lactobacillus helveticus	0.00	9.28	1	1	1	1	1.043
A0A0R2MU11	Uncharacterized protein OS=Lactobacillus helveticus	0.00	2.95	9	1	1	1	1.035
A0A0N9ZTN7	Regulatory protein RecX OS=Lactobacillus helveticus	0.00	5.19	12	1	1	1	1.184
A0A0D5MJC3	MarR family transcriptional regulator OS=Lactobacill	0.00	11.76	7	2	2	2	0.949
A0A0D5MJM5	ABC transporter OS=Lactobacillus helveticus GN=HU	0.00	1.34	8	1	1	2	1.069
U6FT85	Cobalt ABC superfamily ATP binding cassette transp	0.00	7.32	8	4	4	4	1.003
U6F5G8	Uncharacterized protein OS=Lactobacillus helveticus	0.00	12.82	5	1	1	1	0.918
F0NVS3	GntR family transcriptional regulator OS=Lactobacill	0.00	4.37	6	1	1	1	1.283
U4QLS8	tRNA-specific adenosine deaminase OS=Lactobacillu	0.00	9.49	7	1	1	1	0.911
U6FM30	Na(+)/H(+) antiporter OS=Lactobacillus helveticus (	0.00	5.48	9	1	1	1	0.936
A0A0P0A4E7	Uncharacterized protein OS=Lactobacillus helveticus	0.00	8.33	5	2	2	3	1.302
A0A0F7H4B1	Restriction endonuclease OS=Lactobacillus helveticu	0.00	3.41	1	1	1	1	0.996
A0A0N9ZA26	Poly-gamma-glutamate biosynthesis protein OS=Lac	0.00	1.23	2	1	1	1	1.463
U6F6J7	Uncharacterized protein OS=Lactobacillus helveticus	0.00	17.01	1	2	2	3	1.013
F3MLJ8	Uncharacterized protein OS=Lactobacillus helveticus	0.00	7.46	10	1	2	8	0.808
A0A0D5MJX0	MFS transporter OS=Lactobacillus helveticus GN=HU	0.00	2.45	11	1	1	1	0.857
A0A0F7H4D3	Uncharacterized protein OS=Lactobacillus helveticus	0.00	13.86	8	1	1	2	1.114
F3ML13	Uncharacterized protein OS=Lactobacillus helveticus	0.00	9.09	4	1	1	2	0.417
A0A0R2M609	Uncharacterized protein OS=Lactobacillus helveticus	0.00	8.70	1	1	1	1	0.874
A0A0R2LQL0	Uncharacterized protein OS=Lactobacillus helveticus	0.00	8.41	16	1	2	2	1.447
A0A0P0A5V9	Ribonucleotide reductase OS=Lactobacillus helveticu	0.00	18.24	1	1	1	8	0.859
U4QDU3	Ribonucleotide reductase stimulatory protein NrdI O	0.00	3.38	1	1	1	1	1.089
A0A0D5MJS3	Membrane protein OS=Lactobacillus helveticus GN=	0.00	14.23	8	4	4	6	1.120
J7LLG6	Cell wall-associated hydrolase OS=Lactobacillus helv	0.00	5.37	7	1	1	1	1.155
A0A0N9ZUA5	Uncharacterized protein OS=Lactobacillus helveticus	0.00	5.08	3	2	2	2	1.031
A0A0F7H4W9	DNA replication protein DnaD OS=Lactobacillus helv	0.00	10.28	3	2	2	2	0.907
U4QLL0	Single-stranded DNA-binding protein OS=Lactobacill	0.00	11.50	1	1	1	1	0.925
J4BTQ9	ABC superfamily ATP binding cassette transporter, p	0.00	13.01	11	6	6	12	1.033
U6FHU3	Uncharacterized protein OS=Lactobacillus helveticus	0.00	2.07	11	1	1	1	1.108
A0A0R2M6S8	Integral membrane protein OS=Lactobacillus helveti	0.00	10.55	7	2	2	3	1.096
A0A0D5MH23	ABC transporter OS=Lactobacillus helveticus GN=HU	0.00	5.98	9	2	2	6	1.096

A0A0D5MIP7	Uncharacterized protein OS=Lactobacillus helveticus	0.00	6.72	4	2	2	2	1.266
A0A0D5MI49	UPF0122 protein ALV80_04365 OS=Lactobacillus he	0.00	30.97	1	3	3	4	1.215
A0A0D5MI94	Segregation and condensation protein A OS=Lactob	0.00	6.10	6	2	2	2	1.002
F3MNR0	Transposase OS=Lactobacillus helveticus MTCC 546	0.00	8.00	1	1	1	1	1.118
U6FRF5	Uncharacterized protein OS=Lactobacillus helveticus	0.00	9.58	5	1	1	14	0.373
A8YXC6	ABC transporter OS=Lactobacillus helveticus (strain	0.00	3.07	12	2	2	3	1.000
U4QLE3	CAAX amino terminal protease family protein OS=La	0.00	3.33	6	1	1	1	1.095
U6F312	Bifunctional ligase/repressor BirA OS=Lactobacillus I	0.00	19.45	5	7	7	8	0.855
A0A0D5MIR2	Uncharacterized protein OS=Lactobacillus helveticus	0.00	19.83	5	2	2	2	1.023
A0A0R2MS63	Uncharacterized protein OS=Lactobacillus helveticus	0.00	4.07	9	1	1	1	0.972
A0A0R2MNH3	Uncharacterized protein OS=Lactobacillus helveticus	0.00	5.00	7	1	1	1	0.994
U6FM83	Uncharacterized protein OS=Lactobacillus helveticus	0.00	18.42	5	1	1	1	1.004
U6FEM0	PemK protein OS=Lactobacillus helveticus CIRM-BIA	0.00	10.53	1	1	1	2	0.776
A0A0F7H3H1	Amino acid permease OS=Lactobacillus helveticus G	0.00	1.95	7	1	1	1	1.242
F0NWP4	Conserved domain protein OS=Lactobacillus helvetic	0.00	10.96	5	1	1	1	0.907
A0A0D5MKQ6	Metalloprotease OS=Lactobacillus helveticus GN=HU	0.00	6.58	8	2	2	2	1.171
A0A0F7H6G6	Guanine permease OS=Lactobacillus helveticus GN=	0.00	1.61	6	1	1	1	0.913
A4ZGY0	Glycerophosphoryl diester phosphodiesterase OS=La	0.00	11.69	7	3	3	3	0.950
A0A0F7H6F6	Multidrug MFS transporter OS=Lactobacillus helvetic	0.00	1.65	8	1	1	1	1.046
U4QCM2	Outer surface protein OS=Lactobacillus helveticus C	0.00	6.34	1	1	1	2	0.892
U6F537	Prolipoprotein diacylglyceryl transferase OS=Lactoba	0.00	5.80	10	2	2	3	1.182
A0A0D5MGZ9	Prolyl-tRNA synthetase OS=Lactobacillus helveticus	0.00	20.99	7	3	3	4	1.053
A0A0D5MFL3	Copper-binding protein OS=Lactobacillus helveticus	0.00	27.20	5	3	3	4	1.013
F3MNN7	Na-H antiporter OS=Lactobacillus helveticus MTCC 5	0.00	3.44	11	1	1	2	0.934
A4ZGY9	ABC transporter ATP-binding protein ABC2ATP2 OS=	0.00	4.62	2	1	1	1	1.052
F3MRA0	Uncharacterized protein OS=Lactobacillus helveticus	0.00	3.19	7	1	1	1	1.082
F3MNG1	Transcriptional regulator (Fragment) OS=Lactobacill	0.00	10.75	1	1	1	1	0.842
A0A0D5MHD5	Peptide deformylase OS=Lactobacillus helveticus GN	0.00	8.03	7	1	1	1	0.969
U6F6Y3	Uncharacterized protein OS=Lactobacillus helveticus	0.00	5.14	1	1	1	1	1.303
F3MLP8	Glycerophosphodiester phosphodiesterase OS=Lacto	0.00	27.59	6	1	1	3	0.748
U6FRB4	Antibacterial protein OS=Lactobacillus helveticus CI	0.00	82.86	1	1	1	2	0.494
J3ZFF8	Integral membrane protein OS=Lactobacillus helveti	0.00	3.02	8	1	1	2	1.063
A8YV49	Integrase-recombinase OS=Lactobacillus helveticus	0.00	3.72	7	1	1	3	0.986
U6FR60	Competence protein OS=Lactobacillus helveticus CI	0.00	4.35	1	1	1	1	1.234
F3MQW3	Uncharacterized protein (Fragment) OS=Lactobacillu	0.00	15.38	1	1	1	1	1.095
F3MPG7	Bacteriocin helveticin OS=Lactobacillus helveticus M	0.00	3.61	10	1	1	2	0.999
A0A0D5MIT6	Hemolysin III OS=Lactobacillus helveticus GN=ALV8	0.00	3.48	5	1	1	1	1.167
A0A0N9Z9R8	Uncharacterized protein OS=Lactobacillus helveticus	0.00	3.68	3	1	1	1	0.762
A0A0P0A6V1	Uncharacterized protein OS=Lactobacillus helveticus	0.00	5.98	6	1	1	1	1.134
A0A0F7H729	Uncharacterized protein OS=Lactobacillus helveticus	0.00	5.00	8	1	1	1	1.263
F3MLA7	Amino acid permease OS=Lactobacillus helveticus M	0.00	2.17	8	1	1	1	0.978
F3MQ66	Acetate kinase (Fragment) OS=Lactobacillus helveti	0.00	11.43	1	1	1	2	0.872
U6F8J5	Integral membrane protein OS=Lactobacillus helveti	0.00	5.00	10	2	2	2	1.170
F3MQ73	Uncharacterized protein OS=Lactobacillus helveticus	0.00	9.68	3	1	1	1	1.316
F3MN28	L-lactate permease OS=Lactobacillus helveticus MTC	0.00	77.78	1	1	1	2	2.155
U6F5H4	Uncharacterized protein OS=Lactobacillus helveticus	0.00	20.00	1	1	1	3	1.117
F3MNT2	Uncharacterized protein OS=Lactobacillus helveticus	0.00	20.00	1	1	1	1	1.576
U6FQG9	Uncharacterized protein OS=Lactobacillus helveticus	0.00	37.78	1	1	1	1	0.863

A2/all	A3/all	A4/all	B1/all	B2/all	B3/all	B4/all	A1/B	A2/B	A3/B
0.872	1.070	1.107	0.881	0.946	1.080	1.240	0.902	0.870	1.053
0.999	0.885	0.925	1.039	1.125	0.850	1.048	1.073	0.943	0.885
0.931	0.977	1.040	0.938	0.970	1.068	1.257	0.930	0.899	0.958
1.033	1.110	1.070	1.021	1.020	1.046	0.709	1.119	1.141	1.237
0.885	1.035	1.006	0.908	0.963	0.945	1.418	0.854	0.830	0.998
0.961	0.893	0.985	1.005	1.166		1.006	1.100	0.937	0.878
0.998	0.892	1.044	1.005	0.995	0.873	1.054	1.063	1.020	0.901
0.978	0.881	1.411	1.090	0.939	0.778	0.655	1.174	1.091	0.988
1.089	0.883	0.875	1.055	1.069	0.979	1.052	1.008	1.033	0.842
1.372	0.662	0.720	**2.038**	0.764	**3.352**	**0.353**	0.679	1.185	0.577
0.940	0.960	1.051	0.969	0.970	0.819	1.254	1.122	0.998	0.979
0.915	0.947	1.117	1.047	1.020	1.776	0.830	1.053	0.914	0.955
0.917	1.043	1.057	0.943	0.955	1.064	1.097	0.986	0.921	1.051
0.945	0.984	0.947	0.969	0.969	1.078	1.250	0.993	0.898	0.946
0.977	1.006	1.079	0.868	0.948	1.271	1.064	1.075	1.005	1.044
1.003	0.759	0.995	1.039	1.209		1.218	0.827	0.902	0.689
0.919	0.915	0.985	0.963	1.025	0.953	1.172	0.979	0.871	0.897
0.979	1.226	1.018	1.046	0.887	0.865	0.946	1.119	1.028	1.289
1.030	0.883	0.902	1.119	0.990	1.121	1.086	1.005	0.977	0.838
0.960	1.007	1.022	0.972	0.945	0.829	1.163	0.990	0.944	1.045
0.595	1.507	1.347	0.606	0.644	1.200	1.289	0.708	0.667	1.696
0.695	1.204	1.560	0.617	0.667		0.707	1.053	1.188	1.419
0.938	1.160	1.084	0.943	0.955	1.011	0.958	1.028	0.995	1.232
1.090	0.932	1.013	0.749	1.258		1.010	1.098	1.119	0.965
1.029	0.992	1.071	1.044	1.032	0.971	0.867	1.011	1.050	1.028
1.992	0.653	0.595	1.816	0.700		0.517	0.829	**2.002**	0.662
0.818	0.889	1.128	0.978	0.984	1.077	1.160	1.051	0.787	0.864
1.071	0.958	0.835	1.150	1.029	1.219	0.795	1.222	1.064	0.960
1.015	0.964	1.075	1.054	1.084	1.133	0.724	1.078	1.043	0.949
0.942	1.085	1.016	0.890	1.007	0.760	1.157	0.924	0.947	1.071
0.659	1.473	1.417	0.706	0.695	1.120	1.179	0.853	0.773	1.753
0.945	1.187	1.040	0.945	1.031		1.070	0.957	0.964	1.266
0.941	0.990	0.945	0.970	0.965	0.971	1.245	0.949	0.899	0.948
0.902	1.003	1.029	0.925	0.952	0.980	1.317	0.901	0.869	0.987
1.005	1.025	0.940	1.008	1.034	0.841	1.024	1.008	0.998	1.025
0.856	1.008	1.338	0.758	0.963	1.661	1.075	0.878	0.889	1.040
0.835	0.928	1.375	0.833	0.844		1.311	1.063	0.880	0.987
0.657	1.516	1.341	0.707	0.696	1.064	1.298	0.822	0.744	1.727
1.255	0.667	0.643	1.172	1.218	0.909	0.873	1.190	1.156	0.622
1.011	0.850	0.969	1.037	1.052	1.008	1.116	0.937	0.965	0.813
0.913	1.144	0.975	0.804	0.977	0.750	1.411	0.777	0.842	1.103
0.866	1.210	1.169	0.863	0.942	0.796	1.235	0.918	0.901	1.218
1.012	1.007	0.873	0.981	1.059	0.803	1.201	0.995	0.954	0.962
0.941	1.202	1.043	0.993	0.942	1.031	0.975	1.020	0.980	1.267
0.867	1.121	1.167	0.833	0.994	0.762	1.192	0.888	0.884	1.119
0.897	1.028	1.036	0.899	0.934	1.230	1.249	0.943	0.878	1.012
0.989	0.901	0.917	1.016	1.014	0.949	1.196	0.950	0.926	0.849
0.988	0.902	0.926	1.015	1.085		1.122	1.130	0.956	0.881
0.907	1.071	1.084	0.943	0.963	1.020	1.185	0.893	0.871	1.053
0.868	1.058	1.063	0.890	0.887	1.036	1.329	0.897	0.852	1.063
0.728	1.251	0.897	0.761	0.922	1.361	1.489	0.952	0.690	1.198
0.943	0.930	0.931	0.961	0.975	1.017	1.296	0.928	0.886	0.883
0.815	1.506	1.148	0.749	0.798	0.889	1.246	0.864	0.871	1.688
1.030	1.161	0.949	0.954	0.961	1.125	0.930	1.095	1.077	1.225

0.813	1.332	1.227	0.927	1.029		0.623	1.396	0.970	1.604
0.979	1.007	0.932	0.980	1.000	0.843	1.217	0.916	0.962	0.980
0.868	1.145	1.167	0.899	0.945	1.065	1.112	0.946	0.892	1.213
1.052	0.835	0.850	1.058	1.071	0.946	1.140	0.986	0.979	0.782
0.911	1.269	1.172	0.946	0.935	0.980	0.844	1.005	1.003	1.419
0.569	1.797	1.506	0.614	0.602	1.065	1.152	0.829	0.714	2.292
0.975	1.112	1.056	0.857	1.026	0.940	1.093	0.932	1.037	1.137
0.860	1.208	1.110	0.886	0.873	1.033	1.170	0.922	0.893	1.270
0.862	1.265	1.110	0.872	0.878	0.984	1.166	0.917	0.900	1.349
0.684	1.305	1.263	0.758	0.740	1.132	1.445	0.814	0.713	1.365
0.705	1.353	1.373	0.750	0.746	1.125	1.263	0.797	0.777	1.493
0.608	1.605	1.317	0.752	0.861	0.794	1.062	0.854	0.689	1.834
0.915	1.226	1.099	0.900	0.891	0.959	1.052	0.973	0.963	1.310
0.833	1.418	1.111	0.816	0.757	0.634	1.203	0.778	0.910	1.588
1.034	0.854	0.855	1.032	1.081	1.044	1.169	0.995	0.979	0.813
0.851	1.041	1.146	0.878	0.881	0.977	1.220	0.940	0.843	1.082
0.858	1.051	1.056	0.909	0.909	1.122	1.282	0.890	0.840	1.035
0.999	0.864	0.892	1.034	1.103	0.977	1.149	0.992	0.937	0.802
0.953	0.967	1.014	0.982	0.927	2.300	1.113	0.900	0.953	0.997
1.045	0.982	0.913	0.992	1.050	0.922	1.156	0.975	0.999	0.961
1.149	0.915	0.925	1.095	1.119	0.956	0.712	1.129	1.160	0.939
0.953	1.109	1.088	0.957	0.972	0.962	0.943	1.043	1.002	1.172
1.046	1.367	0.795	0.928	0.958	0.666	1.014	1.033	1.095	1.445
1.248	0.684	0.805	1.061	1.433	0.748	0.882	0.960	1.124	0.616
0.909	1.177	1.082	0.907	0.954	1.066	1.052	0.977	0.947	1.230
1.091	0.869	0.929	0.882	1.207	0.718	1.178	0.830	1.031	0.792
0.936	1.193	1.056	0.955	0.937	0.991	0.988	0.990	0.986	1.271
0.944	0.858	0.961	1.093	1.038	1.053	1.050	1.059	0.890	0.816
0.957	0.911	0.951	1.011	1.000	0.920	1.299	0.907	0.892	0.850
1.036	1.090	1.125	1.027	1.032	0.997	0.736	0.982	1.120	1.183
0.896	1.224	1.105	0.885	0.879	1.006	1.166	0.873	0.927	1.267
0.866	1.224	1.115	0.897	0.909	1.124	1.097	0.935	0.903	1.288
0.802	1.225	1.195	0.831	0.845	1.068	1.267	0.858	0.831	1.289
1.031	1.044	0.971	1.020	1.050	0.884	0.936	1.004	1.036	1.064
0.509	1.504	1.340	0.557	0.555	1.062	1.919	0.592	0.515	1.580
0.861	1.278	1.148	0.896	0.885		1.084	1.102	0.918	1.341
1.130	0.798	0.696	1.043	1.147	1.236	1.129	0.915	1.013	0.724
0.774	1.448	1.399	0.753	0.763	1.041	0.912	1.017	0.951	1.746
0.770	1.384	1.184	0.752	0.777	1.197	1.290	0.846	0.830	1.484
0.951	0.972	1.057	1.010	0.986	1.183	1.095	0.947	0.935	0.955
1.294	0.742	0.725	1.106	1.117		0.971	1.186	1.238	0.717
0.962	0.950	1.072	0.901	1.044	0.752	1.071	1.051	0.944	0.946
0.820	1.288	1.138	0.859	0.828	1.057	1.284	0.835	0.862	1.346
0.929	0.980	0.987	0.978	0.963	1.124	1.238	0.910	0.884	0.930
0.908	1.018	1.012	0.931	0.919	1.103	1.304	0.905	0.875	0.977
0.710	1.302	1.323	0.781	0.768	1.048	1.277	0.861	0.762	1.389
0.892	1.038	1.027	0.937	0.926	1.004	1.238	0.945	0.876	1.014
1.016	0.875	0.855	1.044	1.148	1.055	1.091	0.993	0.939	0.816
1.037	0.858	0.906	1.063	1.069	1.024	1.082	1.016	0.978	0.809
0.952	0.943	0.964	1.038	0.972	2.432	1.166	0.794	0.899	0.901
0.950	0.769	1.264	1.009	1.066	2.067	0.848	0.931	0.931	0.761
1.020	1.200	1.095	0.911	0.949	1.057	0.848	1.079	1.124	1.332
1.115	0.917	0.961	0.987	1.103	0.885	0.979	1.041	1.114	0.910
0.984	1.113	1.028	0.988	0.977	0.964	0.907	1.020	1.016	1.175
0.811	1.271	1.252	0.816	0.831	1.089	1.178	0.847	0.874	1.381

1.124	0.945	0.852	1.031	0.883	1.563	0.966	1.276	1.144	0.971
1.031	0.948	0.928	1.019	1.010	0.899	1.082	0.985	0.995	0.919
0.942	1.007	1.021	0.977	0.994	1.009	1.086	0.992	0.925	1.004
1.046	0.921	0.878	1.040	1.098	1.084	1.005	1.011	1.001	0.881
1.344	0.642	0.963	0.960	1.107	1.146	0.793	1.304	1.391	0.677
1.009	0.761	0.972	0.936	1.180	1.176	1.071	1.087	1.020	0.699
0.805	1.393	1.330	0.846	0.841	0.937	1.029	0.915	0.880	1.570
1.139	0.832	0.854	1.140	1.168	1.251	0.895	1.077	1.107	0.804
0.754	1.242	1.185	0.860	0.767		1.427	0.854	0.767	1.274
1.078	0.931	0.834	0.966	1.049	0.878	1.245	0.919	1.012	0.870
0.878	1.268	1.142	0.793	0.885		1.313	0.918	0.883	1.339
0.944	1.143	1.007	0.969	0.953	0.897	1.058	0.995	0.964	1.181
0.766	1.373	1.263	0.803	0.786	0.952	1.297	0.752	0.853	1.450
1.332	0.652	0.571	1.199	1.193	0.976	0.890	1.226	1.223	0.604
0.919	1.171	1.065	0.972	0.915	1.013	1.003	1.007	0.968	1.218
1.337	0.581	0.819	1.044	1.439		0.783	1.118	1.259	0.552
0.906	1.137	1.088	0.939	0.912	1.015	1.088	0.975	0.934	1.192
1.093	0.819	0.817	1.103	1.086	0.987	1.073	1.084	1.024	0.766
0.977	1.106	0.931	1.013	1.047	0.747	0.998	0.970	0.965	1.120
0.845	1.172	1.156	0.905	0.880	1.046	1.115	0.943	0.885	1.237
0.719	1.381	1.434	0.756	0.753	1.004	1.218	0.812	0.801	1.546
1.107	1.032	1.051	1.011	1.108	0.984	0.743	1.040	1.171	1.112
1.215	0.907	0.658	1.062	1.129		1.071	0.978	1.151	0.880
0.999	1.055	1.015	1.018	0.996	1.090	0.933	1.021	1.015	1.092
1.002	1.192	0.953	0.870	0.959	0.666	1.182	0.875	1.021	1.191
0.660	1.558	1.256	0.711	0.685	1.101	1.393	0.795	0.732	1.715
1.180	0.909	0.745	0.911	1.244		1.165	0.914	1.092	0.849
0.902	1.223	1.155	0.928	0.909	0.899	1.001	0.960	0.965	1.332
0.804	1.301	1.176	0.840	0.819	1.030	1.134	0.934	0.879	1.429
0.692	1.417	1.570	0.590	0.737	0.867	1.293	0.716	0.806	1.665
0.925	1.161	1.081	0.914	0.917	1.006	1.044	1.025	0.956	1.236
0.803	1.257	1.206	0.864	0.849	1.155	1.163	0.904	0.842	1.352
1.198	0.749	0.788	1.178	1.193	1.088	0.846	1.114	1.127	0.719
0.967	1.173	1.178	0.988	0.947	1.032	0.800	0.996	1.059	1.253
1.074	0.872	0.780	1.141	1.100	0.797	1.003	1.126	1.020	0.804
0.988	0.907	0.913	0.959	1.180		1.253	0.888	0.978	0.842
1.168	0.971	0.896	1.096	1.145	0.859	0.751	1.015	1.175	1.000
0.803	1.311	1.166	0.820	0.810		1.306	0.907	0.854	1.385
1.033	0.946	1.030	0.881	1.067	0.722	1.094	0.943	1.025	0.949
0.993	0.908	0.953	1.000	1.020	0.872	1.111	0.988	0.938	0.866
0.691	1.951	1.572	0.588	0.624		0.920	0.799	0.993	2.828
0.772	1.386	1.279	0.813	0.815	1.099	1.094	0.914	0.868	1.563
0.764	0.996	1.229	0.802	0.847	1.565	1.526	0.730	0.720	0.947
1.236	0.688	0.718	1.163	1.325	1.336	0.822	1.150	1.143	0.632
1.060	1.006	0.963	1.068	1.048	0.988	0.852	1.099	1.067	1.045
1.133	0.823	0.720	1.092	1.150	0.874	1.019	1.097	1.035	0.775
1.135	0.878	0.826	1.165	1.119	0.800	0.867	1.062	1.089	0.844
0.819	1.191	1.133	0.741	0.997	0.895	1.330	0.790	0.812	1.192
0.951	0.949	0.924	1.014	1.043	1.018	1.138	1.031	0.911	0.911
0.597	1.601	1.429	0.638	0.631	1.272	1.404	0.743	0.671	1.822
1.138	1.144	1.015	0.963	0.997	0.810	0.798	1.058	1.236	1.254
0.939	1.062	1.011	0.940	0.941	0.991	1.189	0.948	0.923	1.055
1.076	0.833	0.921	1.110	1.122	0.973	0.970	0.961	1.013	0.788
0.756	1.261	1.277	0.750	0.766	1.171	1.295	0.850	0.820	1.383
1.010	0.999	0.972	0.981	1.029	0.894	1.110	0.942	0.983	0.978

0.774	1.326	1.189	0.777	0.827	1.230	1.295	0.870	0.833	1.407
0.927	0.999	1.038	0.865	1.034		1.223	1.007	0.913	0.992
1.014	0.977	0.950	1.035	1.031	0.971	1.061	0.949	0.986	0.963
0.860	1.142	1.140	0.902	0.887	1.181	1.199	0.870	0.868	1.155
0.958	1.143	0.998	0.967	0.979	1.010	1.041	0.961	0.983	1.173
0.763	1.208	1.639	0.758	0.723	0.697	1.078	0.953	0.922	1.474
0.919	1.173	1.227	0.924	1.020	0.920	0.883	0.956	0.988	1.239
0.833	1.166	1.184	0.862	0.836	1.042	1.236	0.891	0.880	1.204
1.575	0.638	0.660	0.794	1.063	1.134	0.897	1.573	1.767	0.676
0.947	1.114	1.041	0.959	0.954	0.959	1.092	0.949	0.957	1.135
1.041	1.160	0.814	1.198	1.130		0.751	0.955	1.037	1.166
1.054	0.866	0.951	1.063	1.097	1.174	0.910	1.065	0.999	0.826
0.976	1.017	0.982	0.991	0.988	0.978	1.139	0.951	0.953	0.998
1.016	0.824	0.878	1.032	1.094	1.057	1.137	1.001	0.943	0.760
1.349	0.349	0.581	1.469	1.427		0.697	1.002	1.125	0.294
0.919	1.218	1.110	0.946	0.914	0.950	0.996	1.023	0.979	1.311
0.885	1.122	1.094	0.913	0.899	0.962	1.223	0.888	0.891	1.120
0.915	1.187	1.078	0.931	0.892	0.998	1.178	0.994	0.941	1.233
0.958	0.959	1.055	0.999	0.977	0.984	1.082	0.968	0.932	0.954
1.012	0.937	0.916	1.030	1.007	0.974	1.126	1.026	0.971	0.907
0.741	1.197	1.339	0.762	0.735	1.140	1.391	0.829	0.783	1.286
1.025	0.899	0.924	1.007	1.033	0.952	1.208	0.892	0.953	0.859
1.124	0.813	0.832	1.124	1.125	1.023	0.991	0.926	1.033	0.751
0.896	1.079	1.112	0.934	0.967	0.945	1.163	0.924	0.896	1.128
0.979	0.977	0.953	1.014	0.979	0.940	1.156	0.966	0.943	0.950
1.073	0.875	0.892	1.085	1.070	0.917	1.004	1.037	1.042	0.838
0.918	1.221	1.067	0.950	0.930	0.901	1.040	0.973	0.964	1.286
0.900	1.094	1.073	0.921	0.910	0.990	1.210	0.934	0.909	1.111
0.985	1.159	1.151	1.012	0.999	0.923	0.723	1.020	1.086	1.276
1.042	0.915	0.929	1.044	1.057	1.032	1.036	0.943	0.991	0.878
0.891	1.132	0.995	0.968	0.942	0.868	1.180	0.938	0.877	1.124
1.115	1.000	0.841	1.114	0.960	1.058	0.979	1.029	1.092	0.988
1.052	1.042	0.901	1.054	1.045	0.938	0.898	1.128	1.057	1.056
1.031	1.037	0.985	1.005	1.013	1.104	0.887	1.089	1.056	1.074
0.928	1.156	1.234	0.943	0.921	0.925	0.922	0.965	1.004	1.269
0.891	1.116	1.074	0.922	0.913	1.002	1.162	0.952	0.909	1.119
1.920	0.568	0.616	0.748	1.255	0.926	0.616	1.688	2.202	0.633
1.062	1.137	0.957	1.040	1.018	0.736	0.890	1.008	1.097	1.215
0.943	1.203	1.272	0.977	0.946	1.008	0.739	0.998	1.059	1.342
1.093	1.006	1.010	1.058	0.984	1.294	0.750	1.047	1.214	1.030
0.667	1.325	1.385	0.753	0.723	1.174	1.298	0.816	0.738	1.481
0.641	1.628	1.597	0.681	0.648	1.200	1.055	0.936	0.785	2.062
1.089	0.818	0.896	1.095	1.103		1.036	1.065	1.063	0.776
0.954	0.825	0.903	1.142	1.078	1.549	1.033	0.966	0.861	0.751
0.909	1.257	0.868	0.937	0.903		1.333	0.922	0.900	1.255
1.024	1.043	1.080	1.043	1.006	0.950	0.791	1.075	1.081	1.128
0.932	0.977	0.991	0.952	0.972	1.003	1.207	0.982	0.900	0.966
1.051	0.959	1.012	1.071	1.064	0.886	0.901	0.969	1.045	0.967
0.987	0.909	0.928	1.011	1.076	0.988	1.076	1.042	0.954	0.877
0.840	1.126	1.069	0.813	0.981	0.868	1.218	0.938	0.832	1.152
0.913	1.229	1.171	0.897	0.918	1.036	0.979	0.942	0.986	1.336
0.945	1.129	1.068	0.959	0.955	1.057	0.955	1.040	0.990	1.196
1.006	0.985	0.959	1.025	1.022	1.003	1.054	0.943	0.970	0.948
0.738	1.378	1.213	0.798	0.725	1.115	1.357	0.790	0.785	1.473
1.027	0.924	0.937	1.018	1.054	1.148	1.003	1.047	1.004	0.905

0.903	1.169	1.128	0.913	0.919	0.966	1.080	0.937	0.927	1.233
1.000	0.957	0.969	1.036	1.016	1.033	1.023	1.053	0.993	0.965
0.597	1.367	1.517	0.637	0.639	1.111	1.491	0.700	0.634	1.519
0.949	0.951	0.990	1.007	0.983	0.945	1.180	0.944	0.912	0.916
1.001	0.916	0.923	1.013	1.069	1.045	1.111	1.021	0.964	0.870
1.042	0.926	0.958	1.066	1.048	0.996	0.974	1.054	1.020	0.909
0.961	1.020	1.006	0.969	0.984	0.922	1.165	0.912	0.937	0.997
1.008	1.074	1.019	0.974	0.987	1.105	1.009	0.980	1.013	1.109
0.844	1.175	1.140	0.889	0.886	1.075	1.130	0.962	0.889	1.228
1.044	0.871	0.871	1.056	1.090	0.874	1.060	1.022	0.991	0.829
0.861	1.230	1.156	0.806	0.808	1.148	1.241	0.946	0.918	1.307
0.772	1.275	1.253	0.800	0.797	1.201	1.270	0.855	0.822	1.358
1.027	0.986	0.947	0.971	1.053	0.704	1.018	0.970	1.018	1.002
0.957	1.155	1.102	0.959	0.959	1.062	0.949	0.950	1.001	1.234
1.101	0.885	0.944	1.096	1.123	0.894	0.867	0.993	1.073	0.881
0.969	0.899	0.956	1.037	1.038	0.989	1.106	0.974	0.904	0.838
0.994	0.929	0.943	0.997	0.984	1.095	1.181	1.012	0.964	0.910
0.915	1.188	1.122	0.945	0.910	1.171	1.003	0.992	0.962	1.263
0.957	1.023	1.051	0.929	1.035	0.786	1.052	0.996	0.967	1.012
1.008	0.890	0.997	1.083	1.073	0.852	1.041	0.938	0.972	0.860
0.972	1.189	1.125	0.966	0.962	0.943	0.815	1.052	1.048	1.319
0.921	1.085	1.008	0.941	0.959	1.063	1.149	0.975	0.910	1.062
0.958	1.208	1.215	0.934	0.956	1.025	0.823	0.968	1.095	1.327
0.956	1.171	1.256	0.944	0.924	0.979	0.770	1.081	1.093	1.342
0.899	1.244	1.128	0.922	0.909	0.920	1.003	0.962	0.963	1.349
0.920	1.047	1.066	0.953	0.958	1.002	1.124	1.004	0.933	1.067
1.016	1.006	1.007	1.082	1.024	1.076	0.839	1.035	1.051	1.038
1.114	0.669	0.835	1.047	1.283	0.651	0.985	1.104	1.018	0.617
1.096	0.836	0.891	1.091	1.100	0.994	1.067	0.933	1.025	0.781
1.283	0.732	0.740	1.215	1.256	0.788	0.639	1.221	1.246	0.707
1.144	0.735	0.741	1.152	1.123	0.901	1.042	1.098	1.045	0.668
0.983	0.891	0.963	0.933	1.154	1.101	1.067	1.046	0.971	0.851
0.945	1.110	1.206	0.976	0.944	0.992	0.999	0.876	0.963	1.178
1.130	0.906	0.915	1.026	1.030	1.146	0.962	1.069	1.117	0.905
0.889	1.180	1.072	0.821	0.793	1.068	1.235	1.005	0.957	1.283
0.876	1.372	1.284	0.871	0.857	0.969	0.844	1.009	1.007	1.600
0.929	0.950	0.943	0.933	0.996	1.137	1.296	0.916	0.877	0.899
1.228	0.649	0.704	1.278	1.261	0.817	0.854	0.995	1.067	0.562
1.204	0.761	0.746	1.175	1.227	0.922	0.910	0.994	1.106	0.703
0.988	1.054	0.994	1.009	0.973	0.941	1.044	0.951	0.966	1.078
0.994	0.888	0.920	1.034	1.170	0.642	0.962	0.992	0.939	0.851
1.104	0.827	0.905	1.151	1.128	1.041	0.863	0.958	1.050	0.801
1.078	0.897	0.978	1.131	1.087	1.133	0.935	0.887	1.026	0.863
0.937	1.160	1.124	0.929	0.938	1.011	0.973	0.983	1.007	1.223
0.791	1.369	1.220	0.681	0.739	1.052	1.375	0.855	0.858	1.517
0.883	1.041	1.024	0.917	0.908	1.054	1.321	0.911	0.857	1.015
0.915	1.136	0.997	0.915	0.923	1.075	1.139	1.001	0.923	1.158
1.051	1.167	1.094	1.056	1.031	0.910	0.644	1.073	1.161	1.313
0.881	1.099	1.200	0.900	0.928	1.123	1.046	0.959	0.926	1.154
1.137	0.795	0.913	1.177	1.163	0.972	0.848	0.943	1.069	0.755
0.789	1.258	1.296	0.793	0.735	1.014	1.305	0.865	0.828	1.362
0.965	1.131	1.091	0.972	0.946	1.016	0.994	0.929	1.000	1.183
0.724	1.289	1.255	0.744	0.751	1.158	1.243	0.911	0.774	1.342
1.130	0.840	0.834	1.132	1.159	0.940	0.935	0.958	1.057	0.776
1.158	1.145	0.964	0.955	1.041	0.900	0.809	1.043	1.253	1.277

1.061	1.118	0.981	1.037	1.009	0.929	0.802	1.145	1.121	1.169
1.591	0.604	0.924	0.852	1.015	1.071	0.876	1.333	1.725	0.661
0.630	1.624	1.359	0.627	0.661	1.380	1.431	0.807	0.719	1.889
0.899	1.209	1.266	0.920	0.897	0.993	0.944	0.953	0.979	1.342
0.691	1.315	1.359	0.733	0.805	1.212	1.252	0.852	0.748	1.453
0.983	0.993	0.935	0.999	1.013	0.809	1.213	0.909	0.935	0.950
0.753	0.762	1.243	0.819	0.848	1.069	1.788	0.740	0.673	0.687
0.921	1.099	1.111	0.940	0.911	0.866	1.059	0.933	0.961	1.150
1.012	1.011	1.080	0.981	1.030	1.111	0.805	1.160	1.102	1.061
0.895	0.959	1.106	0.965	0.979	0.813	1.155	1.007	0.920	0.952
0.873	1.228	1.381	0.860	0.867	0.951	0.957	0.922	0.965	1.377
0.842	1.331	1.196	0.863	0.855	1.125	1.074	0.851	0.915	1.455
1.006	1.033	1.012	1.036	1.038	0.961	0.924	0.993	1.002	1.057
1.137	0.677	0.799	1.381	1.106		1.026	0.869	0.990	0.595
0.755	1.378	1.257	0.779	0.761	1.221	1.258	0.824	0.820	1.472
0.985	0.816	0.942	1.023	0.976	1.350	1.171	1.189	0.926	0.775
0.889	1.087	1.026	0.917	0.901	0.909	1.284	0.887	0.881	1.067
0.713	1.492	1.598	0.721	0.727	1.037	0.884	0.937	0.929	1.922
1.068	0.833	0.845	1.083	1.155	0.889	1.053	0.950	1.012	0.784
0.878	1.238	1.119	0.923	0.876	1.269	1.065	0.947	0.925	1.330
1.128	1.058	1.008	1.105	1.031	0.854	0.659	1.070	1.205	1.157
0.959	1.055	0.973	0.965	0.951	0.957	1.163	0.957	0.937	1.040
0.752	1.290	1.234	0.808	0.760	1.118	1.342	0.825	0.779	1.360
0.902	1.158	1.105	0.902	0.919	0.798	1.139	0.957	0.922	1.204
1.000	1.008	0.969	1.007	1.016	0.982	0.989	1.095	1.006	1.022
1.027	0.681	0.956	1.143	1.086	1.234	1.005	1.062	0.945	0.633
0.957	0.938	1.060	0.829	1.163	0.880	1.091	0.922	0.910	0.923
1.030	0.890	1.041	1.052	1.013	0.957	0.998	1.010	0.982	0.871
0.848	0.946	1.195	0.989	1.022		1.144	0.885	0.830	0.934
1.138	0.837	0.876	0.967	1.090	1.762	0.949	1.103	1.101	0.818
0.915	0.917	0.967	0.980	0.949	1.115	1.007	1.353	0.928	0.938
0.873	1.249	1.148	0.888	0.876	1.069	1.074	0.945	0.939	1.324
1.044	1.090	1.075	1.036	1.006	0.968	0.723	1.139	1.147	1.213
0.756	1.362	1.249	0.758	0.753	1.147	1.297	0.842	0.803	1.522
0.761	1.347	1.269	0.797	0.768	1.210	1.242	0.818	0.825	1.452
0.941	1.059	1.060	0.968	1.007	0.928	0.995	1.030	0.956	1.078
1.081	0.679	0.800	1.216	1.409		1.012	0.811	0.919	0.582
0.923	0.698	0.849	1.283	1.039	1.108	0.932	1.295	0.846	0.646
1.100	0.889	0.840	1.060	1.128	0.849	0.902	1.127	1.092	0.882
0.660	1.405	1.338	0.786	0.627	0.638	1.366	0.948	0.733	1.574
0.956	1.181	1.135	0.948	0.794	1.691	0.853	1.212	1.067	1.330
0.834	1.236	1.168	0.860	0.860	0.992	1.145	0.949	0.885	1.308
1.078	1.306	1.140	0.972	0.994	0.707	0.722	1.070	1.248	1.477
0.967	1.081	0.992	0.974	0.963	0.840	1.083	1.023	0.989	1.106
0.892	0.902	1.140	0.933	0.982	1.000	1.251	0.867	0.885	0.870
0.870	1.122	1.005	0.881	0.964	1.325	1.152	0.878	0.875	1.138
0.969	1.119	1.000	0.996	0.968	1.038	1.011	1.026	0.973	1.144
0.767	1.265	1.283	0.834	0.797	1.055	1.078	0.893	0.845	1.419
1.098	1.259	0.999	1.078	1.174		0.533	0.939	1.185	1.371
0.935	0.993	0.966	0.946	1.006	1.004	1.209	0.947	0.915	0.965
0.988	1.029	1.030	1.011	1.003	0.877	1.000	0.970	1.004	1.030
1.293	0.719	0.899	0.935	1.076	1.210	0.816	1.337	1.342	0.759
0.965	1.090	1.052	0.916	0.976	0.833	0.985	1.018	1.017	1.135
0.953	1.255	1.044	0.958	1.101	0.649	0.835	0.905	0.994	1.320
1.128	0.942	1.041	1.124	1.042	0.816	0.715	1.083	1.162	1.001

0.977	1.065	1.157	1.031	0.998	0.921	0.817	0.959	1.020	1.114
1.034	1.038	1.065	1.014	1.044	0.979	0.825	1.147	1.089	1.125
0.700	1.319	1.355	0.731	0.699	1.367	1.356	0.819	0.751	1.462
0.730	1.357	1.294	0.796	0.778	1.021	1.219	0.915	0.821	1.498
1.045	0.933	0.856	1.046	1.094	0.938	1.047	1.025	0.995	0.905
1.040	0.975	1.008	1.071	1.061	1.015	0.871	0.999	1.038	0.986
1.027	0.928	0.926	1.058	1.059	1.043	1.036	0.978	0.992	0.912
0.897	1.098	1.164	0.938	0.923	0.970	1.124	0.908	0.925	1.141
0.918	1.249	1.065	0.867	0.879	1.029	1.163	0.944	0.933	1.335
1.014	1.043	0.987	1.038	1.028	0.833	0.904	1.012	1.033	1.071
0.892	1.099	1.063	0.925	0.925	1.112	1.165	0.987	0.878	1.107
0.981	1.161	1.058	0.980	0.966	1.065	0.890	1.026	1.040	1.229
0.956	0.867	1.025	1.077	0.885		1.319	1.016	0.915	0.836
0.907	0.966	1.050	0.981	0.960	1.123	1.248	0.944	0.850	0.934
1.475	0.564	0.569	1.307	1.471	0.977	0.614	1.087	1.316	0.519
0.923	0.999	0.978	0.967	0.963	0.982	1.252	0.925	0.908	0.988
0.760	1.573	1.244	0.775	0.726	1.918	0.984	0.871	0.860	1.880
0.837	1.278	0.944	1.074	1.009		0.945	1.022	0.845	1.302
0.950	1.028	1.107	0.932	0.987	1.099	1.045	0.989	0.970	1.019
0.725	1.507	1.588	0.670	0.671	1.156	1.083	0.758	0.893	1.874
0.928	1.229	1.110	0.883	0.897	0.999	1.102	0.935	0.975	1.303
0.932	0.981	0.964	0.966	0.974	1.095	1.168	0.958	0.897	0.935
0.887	1.080	1.058	0.921	0.917	1.097	1.173	0.962	0.895	1.093
0.862	1.172	1.180	0.881	0.842	1.070	1.167	0.882	0.896	1.228
1.016	1.003	0.947	1.033	1.053	0.890	0.947	1.068	1.028	1.007
0.981	1.100	1.080	0.950	0.995	1.004	0.881	1.054	1.056	1.181
0.992	0.982	1.085	0.992	1.004	0.985	1.122	0.891	0.960	0.978
0.988	1.007	1.042	1.108	1.019	1.170	0.937	1.031	1.028	1.002
0.910	1.144	1.195	0.974	0.930	0.999	0.981	0.952	0.958	1.221
0.949	1.118	0.941	0.943	0.990	1.032	1.106	0.969	0.958	1.139
0.803	1.300	1.213	0.773	0.776	1.259	1.330	0.822	0.849	1.420
1.128	0.990	0.829	1.082	1.131	0.857	0.817	1.059	1.106	0.994
0.955	1.222	0.980	0.934	0.974		1.199	0.816	0.950	1.228
1.005	1.034	1.002	1.027	0.957	1.078	0.956	1.066	1.036	1.074
1.029	1.001	0.922	0.996	1.010	1.110	1.086	0.995	1.023	1.005
1.268	0.620	0.679	1.249	1.276	0.947	0.798	1.091	1.142	0.567
1.007	0.960	0.960	1.027	0.976	0.980	1.054	1.009	1.000	0.961
1.027	0.924	0.934	1.055	1.070	0.998	1.078	0.891	0.956	0.883
0.882	1.040	1.040	0.907	0.917	1.110	1.349	0.897	0.855	1.012
0.770	1.157	1.291	0.880	0.891	1.051	1.157	0.883	0.817	1.218
0.894	1.201	0.974	0.928	0.834	1.653	1.336	0.740	0.855	1.160
0.969	0.978	0.981	0.994	0.975	0.944	1.166	1.013	0.965	0.975
1.148	0.894	0.924	1.139	1.112	0.950	0.792	0.975	1.129	0.883
1.042	0.824	0.899	1.099	1.107	1.087	1.080	0.909	0.980	0.777
1.067	1.013	0.940	1.061	0.996	1.027	0.965	1.055	1.054	1.012
0.908	0.940	1.095	0.779	1.059	0.740	1.141	1.214	0.927	0.968
1.224	0.840	0.758	1.248	1.050	0.651	0.905	1.031	1.156	0.801
1.115	1.093	1.089	0.988	0.985		0.854	0.969	1.194	1.181
1.192	0.981	1.034	0.966	1.034	0.954	0.741	1.179	1.292	1.074
1.085	0.888	0.877	1.140	1.095	0.976	0.921	1.042	1.038	0.866
1.120	0.818	1.125	0.833	1.028		1.307	0.898	1.109	0.817
0.771	1.267	1.232	0.811	0.777	1.209	1.221	0.831	0.803	1.326
0.909	1.025	1.034	0.937	0.954	1.131	1.157	0.975	0.923	1.046
0.824	1.132	1.210	0.832	0.834	1.219	1.234	0.968	0.871	1.221
0.907	1.317	1.237	0.880	0.865	0.981	0.864	1.030	1.045	1.504

0.648	1.352	1.397	0.715	0.711	1.114	1.392	0.775	0.688	1.454
0.952	1.053	1.026	0.973	0.963	0.924	1.051	1.016	0.965	1.090
0.991	0.872	0.942	0.989	1.045	1.059	1.159	0.985	0.937	0.822
0.892	1.072	1.027	0.944	0.923	1.050	1.295	0.918	0.875	1.066
0.984	0.993	0.967	0.973	0.985	0.994	1.135	0.985	0.978	1.004
0.903	1.195	1.076	0.938	0.918	1.103	1.049	1.022	0.944	1.262
1.187	1.011	0.822	1.054	1.097	0.726	0.676	1.221	1.238	1.104
0.913	1.140	1.091	0.899	0.844	1.057	1.165	1.004	0.945	1.181
0.846	1.295	1.231	0.867	0.835	0.971	1.022	0.981	0.930	1.405
1.040	1.076	0.959	1.010	1.032	0.943	0.912	1.120	1.085	1.117
0.940	1.089	1.162	0.977	0.943	0.935	0.942	0.963	0.995	1.162
1.000	1.178	1.117	1.016	0.965	0.967	0.809	0.963	1.086	1.281
0.974	1.081	0.961	0.950	0.980	0.886	1.107	0.969	0.969	1.094
1.020	1.062	0.968	1.007	1.006	1.006	0.925	1.073	1.032	1.092
0.968	1.275	1.061	0.919	0.930		0.981	1.001	1.028	1.394
0.953	1.119	1.117	0.951	0.946	1.136	0.946	1.014	1.026	1.187
1.134	0.972	0.783	1.111	1.185	0.912	0.831	1.037	1.132	0.981
1.056	0.863	0.908	1.067	1.096	1.133	1.080	0.936	0.994	0.823
0.804	1.287	1.154	0.810	0.768	1.200	1.284	0.879	0.849	1.365
0.944	1.064	1.174	0.975	1.000	1.308	0.886	0.985	0.985	1.121
0.985	1.038	1.034	1.009	0.947	1.041	0.965	1.028	1.003	1.063
1.125	0.895	0.871	1.015	1.166	0.825	0.846	1.176	1.115	0.917
1.013	0.786	0.887	1.071	1.075	1.039	1.131	1.046	0.907	0.743
0.613	1.855	0.779	0.624	0.622	0.801	1.409	1.502	0.710	2.168
1.014	1.087	1.049	1.020	1.019	1.162	0.853	1.085	1.090	1.156
0.951	1.189	1.044	0.960	0.914	1.148	0.931	1.055	1.021	1.265
0.942	0.997	0.971	0.975	0.967	0.871	1.282	0.950	0.895	0.970
0.879	1.127	1.152	0.928	0.903	0.962	1.002	0.980	0.948	1.173
0.857	1.145	1.175	0.915	0.894	1.217	1.152	0.881	0.874	1.187
0.958	1.012	1.039	0.989	0.983	1.173	1.063	0.907	0.942	1.000
1.219	0.613	0.627	1.223	1.299	0.859	0.886	1.131	1.075	0.563
0.993	0.879	0.983	1.031	1.086	1.136	1.076	0.883	0.933	0.849
1.012	1.142	1.164	0.960	0.970	0.966	0.735	1.092	1.136	1.253
1.090	0.788	0.845	1.111	1.130	0.919	1.021	1.114	1.049	0.744
0.994	1.043	0.954	0.955	0.971	0.923	1.231	0.916	0.966	1.006
0.947	1.055	1.063	1.058	0.999	0.715	1.082	0.900	0.961	1.062
0.955	1.023	1.046	0.985	0.995	1.057	1.053	0.933	0.951	1.026
1.131	0.802	0.832	1.049	1.144	0.926	1.044	1.008	1.044	0.736
0.887	1.156	1.140	0.923	0.894	0.934	1.085	0.885	0.925	1.206
1.046	1.026	1.047	1.012	1.029	1.743	0.707	0.999	1.074	1.063
1.041	1.061	0.984	1.084	1.021	0.861	0.930	0.963	1.036	1.077
1.027	0.803	1.084	0.948	1.115		0.939	1.328	1.062	0.838
1.004	1.020	0.959	0.966	1.003	0.790	1.036	1.078	1.051	1.056
1.013	0.931	0.940	1.017	1.032	0.864	1.125	0.966	0.969	0.913
1.021	1.006	1.059	1.016	1.039	1.009	0.957	0.893	1.020	1.029
1.057	0.956	0.943	1.083	1.065	1.143	0.891	1.049	1.038	0.962
0.897	1.266	1.141	0.903	0.906	0.911	1.041	0.923	0.968	1.380
1.026	0.891	0.887	1.015	1.048	0.972	1.188	1.039	0.965	0.848
0.527	2.025	1.523	0.543	0.554	1.037	1.101	0.793	0.715	2.765
0.884	1.072	1.106	0.891	0.879	1.013	1.205	0.921	0.906	1.100
0.916	1.248	1.214	0.926	0.915	0.942	0.891	1.023	0.992	1.442
0.577	1.556	1.346	0.613	0.585	1.131	1.569	0.772	0.642	1.738
0.976	1.322	0.949	1.008	1.040		0.866	0.956	1.023	1.399
1.283	0.697	0.945	0.968	1.066	1.327	0.913	1.183	1.285	0.712
0.918	1.235	1.294	0.906	0.840	1.171	0.875	0.999	1.037	1.391

1.022	1.063	0.920	0.997	1.221	0.785	0.894	0.835	0.979	1.029
0.995	1.076	0.885	1.050	0.887	0.745	1.180	1.058	0.970	1.066
0.854	0.917	1.274	1.000	1.052	1.119	0.965	0.884	0.844	0.914
0.999	1.131	0.938	1.018	1.038	1.168	0.861	0.972	1.060	1.187
0.843	1.348	1.098	0.797	0.811	1.194	1.314	0.842	0.876	1.441
0.938	1.080	1.106	0.968	0.934	1.051	1.039	0.999	0.953	1.110
1.108	0.832	0.939	1.121	1.158	1.008	0.867	0.974	1.071	0.827
1.333	0.606	0.685	1.315	1.261	1.046	0.762	1.016	1.184	0.553
1.094	1.092	0.980	1.052	1.024	0.886	0.680	1.121	1.130	1.143
0.946	1.089	1.120	0.986	0.892	1.069	1.005	0.981	0.971	1.147
0.963	1.098	1.000	0.984	0.994	0.975	0.953	1.003	0.982	1.133
0.974	1.010	0.992	0.995	1.018	0.988	1.084	0.974	0.971	0.998
0.905	1.272	1.169	0.909	0.917	0.860	0.866	1.018	0.998	1.447
1.126	0.784	0.821	1.121	1.097	0.805	1.062	1.007	1.034	0.734
1.026	1.112	1.060	1.062	1.002	0.882	0.774	1.072	1.094	1.232
1.160	0.842	0.818	1.150	1.130	1.262	0.859	1.053	1.126	0.822
1.020	1.083	1.275	1.009	0.989	1.039	0.699	1.005	1.145	1.204
0.931	1.234	1.312	0.901	0.967	0.942	0.755	1.073	1.142	1.463
0.943	1.184	1.052	0.925	0.969	0.988	1.062	0.894	0.981	1.230
1.096	0.998	0.989	1.091	1.031	0.892	0.787	1.120	1.130	1.037
1.057	0.942	0.775	1.044	1.022	1.033	1.155	1.054	1.000	0.919
1.167	0.844	0.799	1.147	1.179	0.811	0.811	1.189	1.125	0.817
0.893	1.055	1.045	0.953	0.941	1.024	1.265	0.928	0.875	1.045
0.955	1.156	1.055	0.918	0.919	0.995	1.124	0.917	0.966	1.182
0.999	0.959	1.018	1.053	1.016	1.038	0.993	0.994	0.970	0.935
0.968	0.999	1.035	1.008	1.105	1.201	1.000	0.965	0.988	0.975
0.656	1.370	1.387	0.754	0.711	0.992	1.291	0.849	0.709	1.490
0.982	1.001	1.043	1.000	1.018	0.893	1.023	1.020	1.006	1.033
0.960	1.166	1.024	1.004	0.972	1.136	0.902	1.109	0.992	1.267
1.093	0.857	0.926	1.259	1.132	1.000	0.875	0.795	0.998	0.792
0.845	1.150	1.076	0.868	0.827	1.263	1.239	0.983	0.862	1.177
0.942	1.008	1.013	0.954	1.000	1.144	1.070	1.004	0.937	1.019
1.082	0.947	1.057	1.051	1.036	0.833	0.908	0.960	1.079	0.972
1.139	0.958	0.940	1.115	1.061	0.837	0.715	1.175	1.183	0.993
1.067	0.975	0.933	1.089	1.025	0.927	0.915	1.097	1.059	0.970
0.983	1.134	0.959	0.962	0.954	1.049	1.050	1.007	0.983	1.172
1.057	1.070	0.998	1.017	1.024	0.927	0.908	1.031	1.097	1.123
1.031	1.110	1.048	1.021	0.989	0.929	0.832	1.012	1.094	1.154
0.884	1.359	1.033	0.895	0.863	0.913	1.102	0.966	0.925	1.462
0.888	1.234	1.115	0.873	0.871	1.040	1.170	0.879	0.923	1.291
1.304	0.905	0.698	1.127	1.149	0.819	0.679	1.224	1.321	0.921
1.079	0.846	0.893	1.070	1.126	0.865	0.976	1.004	1.037	0.818
0.931	1.216	1.064	0.901	0.893	0.898	1.019	1.018	0.988	1.329
0.957	0.980	1.023	1.041	0.998	1.035	1.118	0.974	0.923	0.957
0.847	1.224	1.112	0.852	0.802	1.162	1.210	0.964	0.882	1.316
1.003	1.142	1.149	0.983	0.948	1.052	0.835	1.034	1.088	1.231
0.966	1.056	1.044	1.008	0.959	1.040	0.981	1.034	0.987	1.082
0.698	0.985	1.574	0.850	0.811	0.961	1.153	0.933	0.773	1.051
0.962	0.945	0.981	1.003	0.968	1.008	1.184	1.017	0.927	0.907
1.259	0.664	0.691	1.277	1.363	0.765	0.691	0.978	1.136	0.602
0.970	1.020	0.968	0.977	1.003	0.798	1.072	0.939	0.973	0.997
1.112	0.897	0.949	1.144	1.112	0.857	0.859	0.940	1.058	0.876
0.854	1.188	1.154	0.873	0.863	1.051	1.156	0.911	0.898	1.249
1.075	0.900	0.801	1.067	1.091	0.641	1.087	1.049	1.011	0.855
1.040	1.085	1.071	1.039	0.967	1.000	0.964	1.003	1.067	1.121

1.070	1.134	1.005	1.078	1.031	0.943	0.686	1.130	1.160	1.262
0.956	0.935	0.981	0.982	1.012	1.077	1.143	0.969	0.919	0.919
0.974	1.051	0.938	0.958	0.909	1.113	1.148	1.005	0.986	1.075
1.316	0.667	0.684	1.178	1.256	0.769	0.903	1.111	1.190	0.614
0.981	1.138	1.107	1.028	0.966	1.278	0.819	1.052	1.054	1.231
1.028	0.974	1.146	0.969	0.935	0.842	0.815	1.076	1.108	1.020
0.932	1.087	1.126	0.956	0.940	0.964	0.948	1.004	0.980	1.154
1.112	1.207	0.788	0.959	1.252		0.888	0.826	1.086	1.190
1.066	0.958	0.958	1.062	1.003	0.828	0.934	1.067	1.066	0.966
1.045	1.069	0.905	1.088	1.128	1.278	0.885	1.004	1.041	1.082
0.969	1.058	1.108	1.002	0.966	1.006	1.009	0.874	0.995	1.083
0.913	1.075	1.066	1.054	0.923	0.853	1.033	1.010	0.924	1.098
0.979	1.096	1.035	0.990	0.973	0.887	0.979	1.022	1.023	1.155
1.090	0.822	0.871	1.072	1.212	1.098	1.009	0.903	1.000	0.747
1.082	0.821	0.895	1.213	0.799	0.782	0.806	1.697	1.153	0.884
0.983	0.971	0.972	1.027	1.034	0.984	0.959	0.996	0.962	0.953
0.966	0.995	0.963	0.969	0.984	0.955	1.136	0.927	0.940	0.968
0.921	1.010	0.942	0.922	0.874	1.099	1.341	0.994	0.904	0.967
0.921	1.124	1.104	0.918	0.907	1.006	1.140	0.970	0.985	1.154
0.909	1.122	1.061	0.918	0.897	0.879	1.173	0.940	0.918	1.149
0.919	1.131	1.060	0.974	0.969	1.134	1.097	0.916	0.923	1.153
1.100	0.842	0.836	1.124	1.056	0.797	1.052	1.062	1.053	0.822
0.933	1.159	1.192	0.984	0.972	0.832	0.805	1.014	1.012	1.286
0.866	1.274	0.544	0.861	0.719		1.737	0.570	0.769	1.141
1.122	0.871	0.781	1.099	1.182	0.931	0.901	1.082	1.057	0.834
1.228	0.894	0.832	1.148	1.196	1.010	0.734	1.064	1.193	0.873
0.967	1.030	1.057	1.005	0.964	0.863	1.035	0.980	0.968	1.071
1.080	0.842	0.903	1.058	1.076	1.007	1.024	1.045	1.033	0.809
0.967	1.073	1.039	1.032	0.935	0.840	0.974	1.049	0.979	1.141
0.986	1.098	1.039	0.909	0.949	1.096	1.086	0.948	1.001	1.118
0.985	1.073	1.083	1.008	0.977	0.986	0.951	0.955	1.011	1.123
1.022	1.044	1.038	1.018	1.011	0.845	0.880	1.079	1.071	1.077
0.884	1.070	1.207	0.929	0.957	0.729	1.012	1.089	0.914	1.061
1.001	0.874	0.946	1.113	1.180		0.974	0.959	0.938	0.826
0.984	1.246	1.185	0.908	1.057	0.900	0.844	1.037	1.089	1.370
1.008	1.080	1.143	0.984	1.000	0.958	0.875	0.933	1.024	1.141
0.971	1.034	1.124	1.009	1.000	1.090	1.003	0.895	0.975	1.038
0.961	0.880	1.011	1.047	1.021	1.369	1.088	0.843	0.901	0.826
0.858	1.103	1.248	0.834	0.871	1.008	1.151	0.930	0.900	1.159
0.879	0.983	1.125	0.929	0.876	1.050	1.244	1.029	0.910	0.969
1.000	1.067	1.096	1.021	0.962	0.967	0.910	0.990	1.025	1.114
0.956	1.090	1.104	0.972	0.898	1.142	1.019	0.965	0.995	1.136
1.252	0.748	0.758	1.217	1.275	0.946	0.764	1.008	1.153	0.717
0.959	0.956	0.977	0.998	0.960	0.931	1.183	0.926	0.907	0.934
0.957	1.067	0.982	0.950	0.960	1.007	1.175	0.944	0.954	1.067
0.929	0.836	0.997	1.038	0.899	2.851	1.087	0.998	0.866	0.786
1.065	0.950	1.047	1.060	1.047	1.078	0.831	0.999	1.076	0.975
0.919	1.146	1.038	0.920	0.889	1.080	1.179	0.926	0.934	1.186
1.026	0.957	0.969	1.062	1.053	0.990	1.023	0.923	0.980	0.927
1.188	0.765	0.754	1.187	1.188	0.800	0.856	1.087	1.097	0.715
0.813	1.545	1.300	0.806	0.754	0.869	0.943	0.931	1.009	1.900
0.941	1.132	1.141	0.927	0.912	1.109	0.970	1.001	1.008	1.217
0.851	1.252	1.196	0.856	0.866	1.520	1.164	0.938	0.903	1.302
1.020	0.985	1.016	1.028	1.042	0.914	0.910	1.012	1.019	0.982
0.741	1.340	1.240	0.808	0.788	1.164	1.162	0.862	0.799	1.460

1.042	0.986	0.925	1.059	0.989	0.920	0.988	1.062	1.031	0.993
0.986	0.978	0.973	1.010	1.029	1.055	1.033	1.045	0.964	0.963
1.055	1.082	0.948	1.034	1.063	0.962	0.889	1.015	1.071	1.111
0.988	1.081	1.030	1.023	0.995	0.972	0.932	1.004	1.026	1.125
0.983	1.025	1.113	0.970	1.003	0.986	0.895	1.005	1.021	1.081
0.990	1.075	1.057	0.976	0.910		1.163	0.926	1.000	1.095
0.842	1.568	1.268	0.808	0.825	1.082	0.808	0.991	1.052	1.904
0.857	1.045	1.090	0.912	0.889	0.945	1.316	0.915	0.845	1.000
0.994	1.134	1.078	0.978	0.914	0.984	0.850	1.027	1.043	1.202
0.968	1.272	1.087	0.969	0.942	1.404	0.828	1.162	1.088	1.463
1.032	1.017	1.006	1.013	1.042	0.817	0.883	1.073	1.032	1.059
1.096	0.996	1.084	1.081	1.011	0.907	0.780	0.994	1.143	1.054
1.002	1.062	1.095	1.001	0.999	0.929	0.867	1.012	1.026	1.097
0.985	0.941	1.004	1.016	1.050	0.941	1.052	0.969	0.967	0.956
0.893	1.036	1.036	0.926	0.921	0.966	1.221	0.955	0.858	1.025
1.037	1.038	0.998	1.049	0.995	0.856	0.893	1.034	1.068	1.074
0.886	1.106	1.092	0.895	0.897	1.140	1.218	0.985	0.877	1.116
0.956	1.220	1.162	0.984	0.946	0.977	0.749	1.073	1.062	1.309
1.196	0.838	0.801	1.203	1.247	0.905	0.645	1.114	1.152	0.847
0.944	1.067	1.006	0.973	0.985	1.255	1.048	0.975	0.928	1.041
1.655	0.660	0.967	0.697	0.897	2.330	0.657	1.559	2.063	0.798
1.189	0.876	0.859	1.130	1.165	0.820	0.842	1.047	1.137	0.879
1.055	0.892	0.949	1.126	1.087	0.981	0.891	1.031	1.022	0.857
0.777	1.417	1.322	0.825	0.760	1.191	1.018	0.850	0.893	1.694
0.983	0.984	1.014	1.006	0.978	0.981	1.052	0.962	0.969	0.976
0.910	1.004	0.959	0.961	0.996	0.928	1.252	0.922	0.864	0.942
1.046	1.025	0.880	1.043	1.069	0.958	0.899	1.166	1.082	1.061
1.135	0.752	0.740	1.160	1.189	0.863	0.952	1.058	1.046	0.692
1.025	0.958	0.919	0.998	1.019	0.886	0.996	1.157	1.027	0.959
1.094	1.045	1.050	1.051	1.074	0.816	0.742	1.091	1.137	1.059
0.999	0.957	0.895	0.977	1.012	0.965	1.221	0.969	0.950	0.901
1.015	0.911	0.953	1.045	1.060	1.070	1.071	0.973	0.958	0.865
1.038	0.898	0.877	1.049	1.092	1.001	1.132	0.976	0.987	0.831
1.039	1.067	1.074	0.875	1.043	0.891	0.916	0.915	1.138	1.181
0.871	1.179	1.197	0.758	0.821	0.987	1.260	0.956	0.933	1.260
1.082	0.850	0.829	1.125	1.053	1.214	1.047	0.923	1.003	0.795
0.809	1.415	1.516	0.768	0.779	0.952	0.855	0.973	1.001	1.734
0.866	1.339	1.252	0.859	0.867	1.066	0.861	1.009	0.999	1.572
1.129	0.938	0.872	1.023	1.123	1.015	0.957	1.028	1.131	0.930
0.907	1.118	1.036	0.915	0.917	1.053	1.198	0.960	0.919	1.144
1.001	1.012	0.978	1.009	1.029	1.032	1.001	1.034	0.989	0.988
1.127	0.875	0.867	1.162	1.148	0.864	0.716	1.141	1.141	0.874
1.103	0.918	1.021	1.176	1.045		0.764	1.046	1.115	0.937
0.928	1.346	1.190	0.859	0.872	0.956	0.910	0.993	1.050	1.574
1.004	1.051	0.954	0.927	1.020	0.947	0.956	1.062	1.053	1.071
1.052	0.877	0.907	1.132	1.105	1.061	0.983	0.927	0.980	0.842
0.892	1.345	1.181	0.850	0.852	1.113	0.962	0.963	0.982	1.541
1.089	0.840	0.920	1.038	1.091	1.078	0.953	1.007	1.006	0.810
1.232	0.706	0.687	1.251	1.264	0.894	0.741	1.123	1.129	0.659
1.054	0.912	0.901	1.087	1.082	0.956	0.932	1.047	1.012	0.876
0.906	1.102	1.119	0.905	0.966	0.776	1.159	0.953	0.929	1.128
1.043	1.026	1.030	1.046	0.994	1.065	0.857	1.056	1.123	1.080
1.040	1.059	1.010	1.096	1.022	0.996	0.889	0.921	1.024	1.062
0.990	0.814	1.005	1.079	1.123	0.790	1.075	0.912	0.942	0.757
0.941	1.129	1.066	0.981	0.947	1.076	1.078	0.966	0.972	1.172

1.093	0.941	0.893	1.106	1.061	0.862	0.820	1.041	1.085	0.952
1.094	1.042	0.900	1.089	1.032	1.026	0.796	1.115	1.134	1.087
1.049	0.827	0.967	1.125	1.095	1.323	0.944	1.022	1.003	0.786
0.904	1.365	1.222	0.905	0.892	0.891	0.810	0.980	1.049	1.552
0.953	1.011	1.034	0.960	0.951	0.871	1.214	0.960	0.933	0.996
1.294	0.714	0.641	1.307	1.278	0.725	0.696	1.087	1.206	0.687
1.025	1.088	0.878	1.079	0.999	1.153	1.030	0.878	0.985	1.055
0.923	1.100	1.006	0.924	0.967	0.833	1.164	1.019	0.893	1.131
1.150	0.881	0.749	1.306	1.100	0.990	0.777	1.055	1.084	0.864
0.821	1.090	1.086	0.857	0.893	1.124	1.311	0.945	0.814	1.088
1.062	1.000	0.940	1.047	1.051	0.868	0.934	1.023	1.055	1.012
1.109	0.880	0.914	1.130	1.127	0.834	0.887	1.003	1.075	0.844
0.885	1.114	1.122	0.878	0.936	0.894	1.228	0.981	0.898	1.168
0.916	1.377	1.237	0.906	0.827	0.859	0.833	0.988	1.030	1.596
0.979	1.019	0.991	1.020	1.041	1.056	0.944	1.059	0.983	0.979
0.753	1.409	1.266	0.748	0.777	1.257	1.143	0.931	0.841	1.613
1.150	0.819	0.799	1.146	1.055	1.170	1.024	0.976	1.069	0.756
1.003	0.926	1.035	0.918	0.903	1.464	1.077	1.023	0.990	0.966
0.965	1.137	1.045	0.987	0.997	0.975	0.916	1.097	1.021	1.173
0.869	1.164	1.045	0.913	0.950	0.948	1.067	1.002	0.902	1.187
1.025	1.155	0.990	0.956	0.987	0.920	0.922	1.024	1.093	1.227
0.974	0.953	0.958	1.023	1.032	0.923	1.153	0.929	0.909	0.917
1.086	0.886	1.001	1.109	1.125	0.975	0.784	1.065	1.057	0.906
0.982	0.992	1.030	1.056	0.994	1.009	1.019	0.914	0.973	0.976
1.040	0.924	0.956	1.048	1.142	0.973	0.851	0.993	1.030	0.908
1.021	1.017	1.003	1.006	1.030	1.022	0.906	1.029	1.024	1.050
1.113	0.774	0.936	1.092	1.061	1.163	0.989	1.064	1.076	0.741
1.026	1.077	1.008	1.044	0.990	0.895	0.760	1.089	1.087	1.185
0.814	1.512	1.297	0.746	0.793	1.075	1.036	0.992	0.946	1.794
1.029	1.089	1.100	0.996	0.981	1.020	0.842	1.011	1.087	1.156
0.911	1.105	1.039	0.986	0.951	0.851	1.064	0.918	0.906	1.142
0.884	1.081	1.297	0.930	1.138	0.952	0.785	0.853	0.923	1.139
1.107	0.920	0.868	1.092	1.034	1.049	0.997	1.059	1.064	0.894
1.048	0.900	0.939	1.061	1.131	1.014	1.020	0.927	0.995	0.873
1.016	0.922	0.893	1.033	1.032	1.058	1.108	1.003	0.954	0.922
1.046	1.013	0.969	1.034	1.042	0.868	0.885	1.103	1.068	1.036
1.038	0.864	0.887	1.101	1.048	0.997	1.018	1.085	1.004	0.819
0.978	0.923	0.990	1.005	0.921	0.900	1.117	0.929	0.961	0.869
1.105	0.857	0.790	1.217	1.128	0.865	0.920	0.945	1.017	0.812
1.296	0.762	0.690	1.178	1.204	0.773	0.680	1.398	1.276	0.782
1.085	1.015	0.963	1.066	1.080	0.780	0.796	1.127	1.129	1.036
1.071	0.976	1.017	1.073	1.045	0.955	0.843	1.012	1.065	0.999
0.945	1.250	1.321	0.918	0.856	1.054	0.735	1.106	1.104	1.484
0.861	1.350	1.173	0.833	0.871	0.866	1.033	0.974	0.959	1.517
1.052	0.837	0.970	1.093	1.097	1.176	0.983	0.953	1.006	0.821
1.212	0.752	0.723	1.171	1.205	0.849	0.964	1.026	1.100	0.695
1.036	1.025	0.990	1.056	1.084	0.877	0.807	1.018	1.062	1.045
1.024	1.023	0.968	1.006	1.017	0.906	0.973	0.988	1.056	1.020
0.742	1.149	1.161	0.815	0.789	0.983	1.405	0.842	0.760	1.141
1.110	0.837	0.855	1.109	1.084	0.948	0.905	1.111	1.105	0.854
0.827	1.376	1.259	0.861	0.830	0.855	1.086	0.922	0.918	1.525
0.935	1.067	1.084	0.960	0.907	0.932	1.162	0.922	0.936	1.085
0.985	1.035	0.956	0.982	0.995	1.010	1.041	1.068	0.995	1.050
1.147	0.885	0.831	1.118	1.136	0.901	0.841	1.055	1.093	0.874
1.079	0.956	0.959	1.065	1.082	1.020	0.795	1.066	1.071	0.986

1.121	1.008	0.974	1.078	1.001	0.940	0.773	1.138	1.176	1.072
0.952	1.051	1.070	0.986	0.957	1.158	1.035	1.042	0.970	1.097
1.104	0.886	0.929	1.144	1.090	1.073	0.822	1.052	1.077	0.831
0.993	0.958	1.050	1.029	1.002	0.963	0.983	0.988	1.003	0.997
0.866	1.052	1.062	0.938	0.892	1.082	1.238	0.913	0.846	1.013
1.075	0.882	0.916	1.113	1.100	0.834	0.983	0.964	0.998	0.849
1.114	0.884	0.896	1.118	1.141	1.042	0.795	1.026	1.114	0.879
1.026	0.874	0.971	1.037	1.036	1.245	1.066	1.007	0.982	0.852
1.119	0.791	0.894	1.098	1.100	0.990	1.000	0.995	1.046	0.757
0.998	1.009	0.973	1.089	1.021	1.101	0.912	1.070	0.979	0.982
1.121	1.027	1.025	1.085	1.009	0.988	0.796	1.043	1.162	1.084
1.004	0.945	0.972	1.035	1.022	1.066	1.078	0.926	0.982	0.906
1.057	0.955	0.958	1.060	1.011	0.860	0.932	1.023	1.055	0.970
0.983	0.989	0.997	0.970	1.001	1.184	1.092	1.025	0.977	0.969
1.075	1.020	0.920	1.032	1.032	1.082	0.995	1.092	1.071	1.016
0.996	0.898	0.955	1.023	1.016	0.857	1.173	1.043	0.961	0.861
1.138	0.936	0.857	1.101	1.069	0.879	0.827	1.103	1.199	0.946
0.886	1.096	1.134	0.881	0.872	0.965	1.250	0.845	0.884	1.125
0.944	1.012	1.048	1.017	1.008	0.980	1.064	0.964	0.922	1.003
1.256	0.695	0.815	1.236	1.158	1.274	0.612	1.163	1.257	0.691
1.073	0.893	0.919	1.088	1.033	0.982	0.973	0.941	1.066	0.851
0.892	1.279	1.131	0.866	0.840	0.981	1.122	0.936	0.963	1.371
1.090	0.788	0.873	1.130	1.097	0.924	0.906	0.984	1.038	0.742
1.003	1.134	1.141	0.978	0.932	1.123	0.824	0.983	1.103	1.249
1.048	0.830	0.877	1.097	1.122	1.343	1.029	0.886	0.971	0.774
0.918	1.138	1.035	0.927	0.925	0.896	1.144	0.905	0.914	1.133
1.061	0.982	1.036	1.043	1.101	1.267	0.811	1.025	1.074	0.989
0.669	1.535	1.464	0.692	0.658	1.237	1.260	0.746	0.766	1.805
1.058	0.911	1.031	1.167	1.079	0.793	0.851	0.963	1.038	0.898
0.636	1.748	1.550	0.678	0.636	0.946	0.907	0.872	0.811	2.353
1.007	1.057	0.947	0.985	0.944	0.956	1.049	1.012	1.007	1.069
1.103	1.116	1.043	1.049	0.999	0.881	0.756	1.049	1.176	1.198
0.853	1.076	1.073	0.854	0.918	0.913	1.312	0.998	0.833	1.100
0.980	0.980	1.027	1.056	1.044	1.020	0.914	1.020	1.019	1.002
1.213	0.777	0.846	1.103	1.252	0.842	0.761	1.048	1.192	0.758
1.081	0.796	0.842	1.073	1.143	0.943	0.977	1.005	1.054	0.757
1.119	0.769	0.962	1.104	1.186	1.102	0.958	1.078	1.016	0.723
1.115	0.870	0.956	1.063	1.050	1.076	0.930	0.980	1.074	0.852
1.130	0.695	0.816	1.214	1.142	0.920	0.897	0.990	1.048	0.639
1.183	0.984	0.884	0.963	1.076	0.857	0.930	0.955	1.224	1.013
0.901	1.122	1.040	0.824	0.929	0.699	1.366	0.874	0.886	1.114
0.931	1.090	1.034	0.963	0.938	1.040	1.219	0.869	0.917	1.070
1.099	0.831	0.911	1.063	1.018	0.900	1.079	0.981	1.070	0.800
1.058	0.927	1.093	1.055	0.919	0.762	1.045	0.951	1.062	0.938
1.022	1.025	0.900	1.037	1.020	0.854	1.018	1.062	1.004	1.025
1.086	0.704	0.802	1.169	1.235	1.264	0.941	0.989	1.011	0.659
0.969	1.059	1.013	0.946	1.042	0.899	1.060	0.951	0.955	1.069
1.091	0.821	0.897	1.126	1.127	0.750	0.914	0.971	1.044	0.786
1.053	0.954	0.950	1.049	1.042	0.974	0.916	0.945	1.017	0.962
1.000	0.922	1.079	1.063	0.978	0.785	1.038	0.953	0.982	0.922
0.987	1.070	0.954	0.936	0.975	1.040	1.067	0.978	0.986	1.040
1.160	0.873	0.770	1.124	1.115	0.922	0.883	1.110	1.108	0.855
1.020	0.906	1.007	1.079	1.033	0.964	0.946	0.972	0.991	0.892
1.022	1.150	1.098	0.969	0.963	0.973	0.773	1.078	1.118	1.261
1.051	0.856	0.840	1.096	1.071	0.785	1.093	1.058	0.986	0.798

0.931	1.110	1.092	0.919	0.869	1.094	1.110	0.996	0.973	1.162
0.964	1.010	1.040	1.001	1.028	1.026	1.104	0.862	0.917	1.002
1.051	0.938	0.900	1.043	1.016	1.133	1.055	1.047	1.002	0.891
0.991	1.132	1.043	0.961	1.069	0.642	0.910	1.017	1.023	1.182
0.991	0.939	1.001	0.961	1.012	0.969	1.150	0.991	0.955	0.936
1.160	1.017	0.893	0.995	1.073	0.719	0.975	0.973	1.173	1.020
1.096	0.921	0.931	1.086	1.061	0.895	0.943	1.023	1.079	0.924
0.983	0.987	1.072	1.051	1.002	1.104	1.029	0.994	1.013	1.002
0.791	1.393	1.279	0.801	0.817	1.111	0.994	1.017	0.938	1.515
1.054	1.063	1.053	1.064	1.002	0.775	0.802	1.016	1.110	1.108
0.866	1.200	1.227	0.872	0.865	0.887	1.206	0.896	0.897	1.218
1.027	0.856	0.885	1.107	1.106	0.852	1.270	0.739	0.894	0.782
1.121	0.876	0.854	1.073	1.116	1.006	0.896	1.053	1.068	0.861
1.099	0.946	1.045	1.088	1.101	0.896	0.778	0.938	1.090	0.980
0.969	0.973	0.975	1.007	0.982	1.027	1.165	0.988	0.961	0.959
0.900	1.127	1.046	0.930	0.893	0.879	1.069	1.037	0.877	1.225
0.903	1.069	1.024	0.883	0.975	0.979	1.257	0.913	0.896	1.060
0.904	1.085	1.161	0.922	0.918	1.008	1.091	1.026	0.907	1.115
1.182	0.784	0.808	1.113	1.125	1.387	0.957	1.021	1.114	0.735
1.026	0.925	0.959	1.070	1.024	1.128	0.948	1.026	0.989	0.904
0.930	1.104	0.977	0.943	1.003	1.342	1.077	0.960	0.932	1.117
0.995	1.013	1.024	1.021	1.083	0.878	0.968	1.015	1.006	0.972
0.891	1.362	1.188	0.908	0.897	0.915	0.830	1.069	1.043	1.627
0.895	1.060	1.023	0.965	0.964	1.258	1.125	0.953	0.875	1.045
1.017	1.023	0.965	1.009	1.017	1.034	0.884	1.029	1.053	1.021
1.098	0.963	0.909	1.106	1.039	0.794	0.787	1.084	1.124	0.973
1.114	0.982	0.846	1.074	1.147	0.846	0.799	1.014	1.111	0.982
1.025	1.040	0.918	1.049	0.999	0.972	1.033	1.011	1.003	1.042
1.086	0.840	0.825	1.110	1.105	1.053	0.945	0.978	1.056	0.797
1.053	0.974	1.009	1.103	1.020	1.016	0.837	0.977	1.060	0.956
1.008	1.071	0.835	1.054	1.086	0.769	0.911	1.063	0.955	1.030
0.921	1.097	0.987	0.997	0.974	0.864	1.099	0.967	0.925	1.085
0.932	1.197	1.064	0.877	0.889	1.012	1.127	0.927	0.947	1.249
1.095	0.906	0.921	1.122	1.073	0.970	0.738	1.095	1.062	0.883
0.891	1.276	1.081	0.849	0.890	1.000	1.090	0.965	0.935	1.446
0.958	1.203	1.162	0.907	0.931	0.993	0.824	1.051	1.080	1.349
0.980	1.004	1.106	0.916	0.941	1.150	0.999	1.039	0.987	1.062
1.145	0.665	0.814	1.201	1.232	1.062	1.049	0.851	0.991	0.579
0.779	1.335	1.184	0.766	0.865	1.037	1.230	0.835	0.826	1.424
0.922	1.104	1.096	0.935	0.946	0.802	1.076	0.915	0.944	1.127
1.171	0.751	0.869	1.105	1.148	1.002	0.958	1.013	1.104	0.705
1.073	0.915	0.979	1.071	1.079	0.957	0.934	1.047	1.043	0.905
1.115	0.803	0.842	1.130	1.145	1.025	0.939	0.953	1.080	0.755
0.686	1.400	1.333	0.720	0.617	1.316	1.325	0.833	0.755	1.558
0.929	0.908	0.987	0.882	1.137		1.219	1.099	0.896	0.884
1.059	1.138	1.124	0.994	0.972	0.715	0.752	1.064	1.164	1.290
0.972	1.010	0.977	1.000	0.999	0.955	1.108	0.994	0.945	0.990
1.029	0.983	0.953	0.966	0.974	0.747	1.032	1.160	1.043	1.015
1.105	0.822	0.906	1.053	1.070	0.889	0.947	1.095	1.064	0.823
1.001	0.934	0.896	1.040	1.055	0.786	1.047	0.969	0.961	0.904
0.974	0.897	0.981	1.011	1.003	0.912	1.126	0.927	0.934	0.868
0.824	1.185	1.185	0.883	0.872	1.218	1.217	0.940	0.863	1.208
0.852	1.451	1.197	0.847	0.823	1.032	0.869	1.029	1.003	1.671
1.101	0.829	0.890	1.094	1.118	1.118	0.928	1.023	1.058	0.782
1.087	0.989	0.954	1.055	1.059	0.834	0.941	1.101	1.100	1.006

1.063	0.892	0.857	1.114	1.030	0.936	1.021	1.036	1.014	0.864
1.064	0.855	0.993	1.047	1.085	1.061	0.994	1.014	1.023	0.840
0.983	1.220	1.064	1.011	0.999	0.827	0.755	1.081	1.077	1.301
1.204	0.650	0.744	1.253	1.331	1.132	0.821	0.929	1.111	0.590
0.842	1.053	1.153	0.909	0.923	1.892	1.162	0.940	0.851	1.064
1.080	0.914	0.865	1.063	1.016		0.929	1.305	1.089	0.929
1.150	0.899	0.851	1.212	0.984	1.118	0.878	1.034	1.113	0.878
1.027	1.077	0.950	1.021	1.030	0.932	0.880	1.081	1.045	1.088
0.987	0.884	1.016	1.152	0.970	1.103	1.005	0.883	0.933	0.858
1.007	0.974	0.963	1.004	0.990	1.330	1.071	1.038	0.995	0.975
0.992	0.999	1.037	1.003	0.984	1.269	1.013	0.934	0.977	1.003
0.985	1.157	1.046	0.988	1.058	1.298	0.738	1.198	1.064	1.251
0.971	0.945	0.864	1.001	1.031	0.991	1.094	1.003	0.924	0.909
1.064	1.007	1.080	1.049	0.998	0.825	0.859	0.977	1.075	1.056
1.024	1.098	1.160	0.994	0.990	0.940	0.821	1.001	1.110	1.199
1.001	0.894	0.928	1.027	0.964	1.090	1.168	0.988	0.944	0.874
1.004	0.977	0.895	1.160	0.950	0.940	0.931	1.051	0.984	0.994
1.031	0.920	0.948	0.982	1.023	1.076	1.002	1.010	1.007	0.875
1.229	0.782	0.737	1.185	1.215	0.878	0.826	1.121	1.162	0.738
0.906	1.121	1.173	1.000	0.942	0.843	0.912	1.051	0.977	1.225
1.114	0.790	0.924	1.116	1.123	1.107	0.912	1.030	1.057	0.747
1.138	0.853	0.812	1.067	1.092	1.068	1.156	0.824	1.030	0.779
1.277	0.813	1.143	1.010	1.432		0.500	0.956	1.359	0.827
0.984	1.015	1.046	0.950	0.948	0.902	1.175	0.963	0.999	1.028
1.111	0.946	0.914	1.014	1.041	0.939	0.904	1.037	1.119	0.972
1.089	0.937	0.955	1.089	1.063	0.887	0.918	1.006	1.077	0.987
1.167	0.924	0.847	1.097	1.056	0.889	0.859	1.096	1.140	0.962
0.962	1.051	0.995	1.007	1.016	1.250	0.989	1.069	0.964	1.054
0.999	0.941	0.889	1.161	1.110	0.797	0.966	0.988	0.933	0.905
0.968	0.944	0.990	1.007	1.048	1.093	1.067	0.997	0.932	0.940
0.388	3.040	1.490	0.406	0.383	1.067	0.660	0.860	0.760	6.690
1.241	1.025	1.055	0.818	0.920	1.397	0.888	1.160	1.423	1.222
0.976	1.126	1.006	0.915	0.958	1.612	1.110	0.952	0.979	1.150
1.096	1.133	1.181	0.926	0.905	0.815	0.742	1.125	1.303	1.308
1.075	0.836	0.969	1.094	1.057	0.804	0.996	1.136	1.064	0.842
0.948	1.044	1.046	1.011	0.913	1.007	1.018	1.016	1.000	1.101
0.906	1.045	1.212	0.940	0.953	1.326	0.965	0.989	0.936	1.145
0.878	1.261	1.111	0.841	0.853	1.237	1.107	0.917	0.934	1.387
1.086	0.837	0.950	1.020	1.096	0.815	1.007	0.956	1.068	0.823
0.950	0.856	1.052	1.036	0.999	1.223	0.923	1.045	0.924	0.854
1.114	0.921	0.960	1.035	1.069	1.173	0.869	1.069	1.131	0.953
1.015	0.981	0.907	1.071	0.987	1.036	1.017	1.092	1.003	0.960
1.082	0.913	0.865	1.008	1.053	0.834	1.071	1.079	1.056	0.883
1.115	0.790	0.846	1.074	1.165	1.400	1.006	0.970	1.022	0.727
0.921	0.977	1.100	0.948	0.923	1.021	0.966	1.044	0.936	0.944
0.959	1.157	1.136	0.917	0.929	0.909	1.064	1.046	1.024	1.187
0.964	1.141	1.051	1.002	0.944	0.926	0.910	1.022	1.009	1.161
1.077	0.888	0.918	1.085	1.072	0.737	0.898	1.062	1.048	0.868
0.969	0.972	1.000	0.990	0.976	1.256	1.116	0.955	0.908	0.944
1.061	1.168	1.005	1.028	1.013	0.883	0.731	1.094	1.121	1.295
1.103	0.939	0.950	1.128	1.107	0.948	0.757	1.153	1.144	0.950
0.851	1.203	1.133	0.902	0.904	1.301	1.130	0.966	0.871	1.242
0.861	1.175	1.254	0.910	0.878	1.075	0.894	1.020	0.970	1.260
0.955	0.900	0.939	0.998	1.111	1.456	1.110	0.915	0.856	0.817
0.989	0.985	0.939	0.982	0.994	1.084	1.126	0.937	0.951	0.962

1.362	0.733	0.708	1.092	1.281	0.869	0.612	1.125	1.252	0.726
0.906	0.897	1.123	0.981	0.930	1.647	1.220	0.939	0.864	0.869
1.004	1.049	0.999	1.060	1.059	1.000	0.941	1.015	1.032	1.072
1.140	0.772	0.773	1.194	1.241	0.970	0.797	0.977	1.062	0.714
1.035	1.195	1.277	0.999	0.889	1.154	0.826	1.012	1.140	1.342
0.918	1.480	1.214	0.826	0.807	1.004	0.813	1.201	1.233	1.753
0.901	1.035	1.127	0.930	0.982	1.099	1.258	0.880	0.884	1.077
0.852	1.178	1.198	0.843	0.930	0.970	0.941	1.043	0.975	1.282
1.140	0.797	0.899	1.218	1.081	1.224	0.840	0.974	1.061	0.777
1.025	0.852	0.970	1.149	1.061	1.325	1.003	0.931	0.961	0.824
1.094	1.191	1.007	1.039	0.976	0.830	0.805	1.019	1.169	1.277
1.059	0.927	0.988	1.069	0.997	1.170	0.944	1.032	1.075	0.907
0.722	0.962	1.154	0.819	0.754	1.233	1.563	0.890	0.690	0.918
0.925	1.159	1.043	0.905	0.861	1.075	1.080	1.031	1.001	1.216
1.026	1.097	0.961	0.942	0.987	1.355	1.042	1.006	1.041	1.123
0.930	0.835	1.115	1.000	0.987	0.968	0.990	1.233	0.938	0.849
1.143	0.875	0.888	1.105	1.130	1.037	0.871	1.038	1.103	0.851
0.995	1.031	1.029	0.985	1.037	0.847	0.928	1.055	1.006	1.040
1.176	0.732	0.765	1.053	1.211	0.936	0.921	1.072	1.072	0.681
0.991	1.168	1.069	0.980	0.951	2.183	0.935	1.090	1.059	1.299
0.941	1.161	1.029	0.906	0.889	1.042	1.049	0.973	0.955	1.218
0.972	0.932	1.004	1.096	1.033	1.600	0.887	1.177	0.979	0.947
1.095	0.863	0.929	1.151	1.105	0.694	0.888	0.983	1.020	0.853
1.042	1.082	1.076	1.056	1.011	0.691	0.962	1.007	1.043	1.085
1.120	0.834	0.929	1.020	1.093	0.899	1.006	1.041	1.076	0.808
0.964	0.949	0.874	0.973	1.016	1.110	1.010	0.965	1.000	1.008
0.921	1.048	1.200	0.897	0.943	0.817	1.126	0.913	0.948	1.066
0.865	1.164	1.264	0.877	0.822	1.104	1.130	0.979	0.941	1.235
1.204	0.773	0.870	1.147	1.045	1.246	0.873	1.022	1.153	0.728
1.015	1.042	0.954	1.026	0.970	0.857	0.966	1.007	1.040	1.038
0.921	1.133	1.034	1.003	0.956	0.847	0.785	1.006	1.010	1.126
1.027	0.877	0.969	1.014	1.024	1.197	0.886	0.945	1.004	0.859
0.990	0.938	1.045	1.036	1.022	0.790	0.971	0.999	1.031	0.951
0.942	1.020	1.025	0.944	1.044	1.165	1.046	0.948	0.916	0.993
0.921	0.793	1.026	1.049	1.081		1.066	1.139	0.878	0.763
1.616	0.574	0.512	1.257	1.390	0.857	0.546	1.115	1.540	0.544
1.055	0.760	0.949	1.057	1.194	0.755	1.000	0.974	1.014	0.739
0.990	1.116	1.104	0.923	0.975	0.991	0.875	0.941	1.063	1.206
1.010	1.116	1.121	1.008	0.948	0.953	0.858	1.080	1.077	1.200
1.015	1.042	1.005	1.029	1.013	0.753	0.989	0.971	1.031	1.036
1.094	0.836	0.861	1.135	1.222	0.917	0.863	1.027	1.034	0.792
0.946	0.998	0.973	1.068	0.982	0.864	0.946	1.034	0.960	0.991
0.920	1.136	1.046	0.956	0.931	0.835	1.128	0.975	0.938	1.150
1.084	0.991	0.938	1.012	0.909	0.659	1.262	0.850	1.043	0.962
1.050	0.860	0.902	1.048	1.065	1.120	1.017	0.995	0.991	0.827
1.009	0.985	0.956	0.984	1.034	1.084	1.080	0.949	0.975	0.961
1.041	0.805	0.855	1.083	1.129	0.992	1.021	1.024	0.974	0.763
0.950	1.129	1.097	0.933	0.930	1.104	1.033	0.981	0.984	1.181
1.098	0.756	0.791	1.097	1.169	0.867	1.116	0.997	1.012	0.675
0.917	1.110	1.128	0.923	0.894	0.934	1.097	1.010	0.946	1.132
1.163	0.698	0.795	1.168	1.170	0.996	0.982	1.027	1.047	0.641
1.209	0.805	0.988	1.039	1.038	0.823	0.887	1.126	1.226	0.823
0.911	1.374	1.265	0.849	0.914	1.262	0.846	0.985	1.059	1.636
1.009	1.181	1.030	1.033	0.870	0.885	0.813	1.047	1.103	1.309
0.948	1.187	1.114	1.008	0.931	1.072	0.920	1.041	1.049	1.241

1.096	1.001	0.961	1.028	1.028	1.044	0.922	1.051	1.148	1.030
1.026	0.965	1.054	1.102	1.076	1.015	0.865	1.018	1.009	0.963
0.867	1.269	1.405	0.880	0.825	0.928	0.929	0.933	0.979	1.461
1.148	0.772	0.804	0.989	1.043	1.273	0.793	1.130	1.105	0.788
1.013	0.977	0.989	1.111	0.933	1.142	0.945	1.069	1.065	1.081
1.131	0.781	0.878	1.136	1.100	0.974	1.035	1.004	1.048	0.731
0.533	1.330	1.502	0.658	0.560	1.408	1.435	0.667	0.587	1.452
0.935	0.761	1.025	0.940	1.282	0.772	0.849	1.215	0.913	0.750
0.964	1.243	1.216	0.934	0.909	0.871	0.867	1.157	1.160	1.398
1.044	0.932	0.967	1.040	1.070	1.064	0.973	1.016	1.011	0.925
1.088	0.860	0.865	1.181	1.127	0.890	0.891	1.012	1.015	0.814
1.269	0.969	0.869	0.936	1.064	1.088	0.867	1.046	1.296	1.018
1.101	0.795	0.870	1.162	1.116	0.704	0.942	1.034	1.030	0.741
1.064	0.865	0.946	1.016	1.088	0.673	1.038	1.001	1.007	0.826
1.119	0.790	0.898	1.142	1.121	0.857	0.928	0.997	1.035	0.759
0.907	1.132	1.081	0.924	0.894	1.017	1.157	0.843	0.923	1.203
1.156	0.868	0.832	1.096	1.095	0.853	0.897	1.154	1.105	0.840
0.877	1.182	1.105	0.909	0.818	0.942	1.076	0.989	0.971	1.235
1.042	1.199	1.098	1.003	0.966	1.034	0.750	1.096	1.146	1.307
0.967	0.858	0.993	0.994	1.228		0.905	1.129	0.938	0.840
0.847	0.786	1.398	0.957	0.942	1.063	0.894	1.080	0.876	0.802
0.719	1.517	1.778	0.702	0.674	0.926	0.806	0.932	0.968	2.061
1.404	0.779	0.723	1.157	1.282	0.833	0.615	1.076	1.368	0.741
1.220	0.571	0.619	1.036	1.006	1.955	0.576	1.996	1.373	0.596
1.113	0.838	0.842	1.106	1.134	1.117	1.011	0.968	1.034	0.777
0.998	1.013	1.074	0.994	1.010	0.936	0.915	0.977	1.003	1.040
1.104	0.821	0.832	1.132	1.115	0.979	0.833	1.084	1.063	0.797
1.002	0.882	0.897	1.119	1.116	0.936	0.868	1.051	0.993	0.849
0.980	0.984	1.099	0.981	0.998	0.997	1.038	0.897	0.981	0.954
1.081	0.904	0.872	1.093	1.078	0.731	0.891	1.043	1.060	0.893
1.114	0.835	0.734	1.182	1.233	1.012	0.895	1.055	1.042	0.774
1.114	1.043	0.833	1.096	1.080	0.916	0.717	1.165	1.149	1.100
1.022	1.010	0.921	1.007	0.997	1.003	1.155	1.083	1.017	0.982
1.029	0.884	0.927	1.113	1.082	1.080	0.845	1.047	1.000	0.901
1.018	1.007	0.971	1.137	0.942	1.265	0.975	0.909	0.989	0.987
0.725	1.369	1.741	0.738	0.682	1.113	0.806	0.989	0.923	1.796
1.115	0.823	0.949	1.154	1.167	1.343	0.873	0.979	1.099	0.805
0.892	1.155	1.060	0.942	0.826	1.357	1.171	0.885	0.898	1.229
0.971	1.166	1.095	0.852	0.924	1.387	1.070	0.996	0.996	1.192
1.126	1.042	0.948	0.980	0.994	0.847	0.828	1.176	1.212	1.200
1.040	1.002	0.956	1.067	1.009	1.049	0.899	1.007	1.028	0.969
1.119	0.706	0.874	1.084	1.246	1.037	0.859	1.130	1.104	0.686
1.017	0.996	1.052	1.037	1.070	0.858	0.901	1.026	1.067	0.974
0.951	1.028	0.956	0.928	0.951	1.015	1.337	0.834	0.891	0.964
1.011	0.917	0.907	1.014	1.073	0.870	0.990	1.116	0.971	0.885
0.902	1.151	1.255	0.905	0.877	1.260	0.963	0.958	1.027	1.254
1.071	0.896	0.884	1.077	0.980	0.895	0.999	1.072	1.028	0.899
1.058	0.968	0.885	0.973	1.045	0.796	1.086	0.967	1.063	0.962
0.921	1.154	1.094	0.987	0.949	0.875	1.021	0.918	0.946	1.218
1.026	0.999	0.936	1.013	0.888		1.236	1.047	1.013	0.995
1.375	0.633	0.624	1.432	1.363		0.600	1.016	1.229	0.574
1.144	0.795	0.702	1.186	1.183	0.726	0.820	1.139	1.091	0.729
1.140	0.820	0.834	1.157	1.184	0.659	0.870	1.089	1.095	0.783
1.028	0.981	0.958	1.030	1.106	1.060	0.908	1.017	0.985	1.020
1.065	0.845	0.923	1.051	1.108	1.012	1.013	0.906	1.034	0.786

1.127	0.775	0.826	1.156	1.143	1.108	0.991	0.924	1.028	0.703
1.081	0.884	0.936	1.016	1.090	0.815	0.886	1.040	1.089	0.851
0.971	0.982	0.896	1.075	0.924	1.108	1.085	1.124	0.949	0.935
1.054	0.943	1.009	1.121	1.104	1.102	0.893	0.863	1.016	0.955
0.911	0.959	1.123	1.018	0.948	0.945	1.118	0.924	0.903	0.976
1.050	0.889	0.883	1.109	1.070	0.994	0.971	1.044	1.011	0.876
0.984	1.187	1.075	0.936	0.934	0.980	0.868	1.076	1.063	1.324
1.085	0.798	0.781	1.156	1.209	0.899	0.893	1.000	1.026	0.723
0.948	1.066	1.043	0.959	1.074	1.494	1.184	0.831	0.902	1.029
0.951	0.920	1.050	1.052	0.904	1.066	0.956	1.206	0.953	0.926
0.957	0.982	0.979	0.991	0.991	1.122	1.168	0.926	0.953	0.958
0.950	1.061	1.104	0.964	0.982	0.999	0.945	1.074	0.962	1.128
0.946	1.206	1.087	0.915	0.927	0.970	1.006	1.017	1.030	1.280
1.130	0.642	1.102	1.115	1.177	1.594	0.644	0.965	1.112	0.608
1.133	0.786	0.849	1.151	1.132	0.805	0.927	1.016	1.066	0.732
1.135	1.033	0.890	1.030	1.119	0.920	0.879	0.933	1.122	1.030
1.014	1.155	1.056	0.949	1.011	0.941	0.787	1.165	1.100	1.252
1.359	1.048	0.870	0.824	1.068	1.084	0.816	1.104	1.400	1.115
1.059	0.998	0.979	1.069	0.984	1.166	1.001	0.959	1.063	1.010
1.042	0.844	0.872	1.074	1.084	0.836	1.138	0.972	0.966	0.826
1.005	1.080	1.034	1.069	0.960	1.007	0.952	1.043	1.027	1.105
1.029	1.002	0.977	1.012	1.048	0.733	1.015	1.008	1.022	1.008
1.022	0.822	0.995	1.113	0.986	1.004	0.933	0.973	0.986	0.778
0.938	0.996	1.010	0.895	1.028	0.816	1.241	0.971	0.912	0.977
1.344	0.590	0.687	1.224	1.420	1.003	0.646	1.115	1.154	0.541
1.019	0.997	1.079	1.021	0.939	0.885	1.058	0.993	1.042	1.023
1.171	0.866	0.886	1.170	1.046	0.945	0.837	1.144	1.157	0.869
1.043	1.256	0.994	1.022	0.956	1.178	0.819	1.075	1.135	1.386
0.821	0.868	1.140	0.994	1.162		1.144	0.922	0.773	0.825
0.953	1.081	0.996	0.955	0.944	1.086	0.999	1.084	0.988	1.142
1.003	0.927	1.086	0.947	0.913	1.041	1.206	0.896	0.982	0.915
1.134	0.687	0.915	1.125	1.142	1.039	0.893	0.878	0.979	0.628
0.942	1.178	0.959	0.866	1.073	1.003	1.094	0.978	0.926	1.142
0.885	1.479	1.295	0.862	0.829	0.902	0.756	1.079	1.063	1.862
1.225	0.669	0.774	1.277	1.214	0.904	0.902	0.899	1.092	0.601
1.034	1.013	1.093	0.885	0.966	0.981	1.120	0.900	0.993	1.033
1.184	0.684	0.806	1.122	1.260	0.957	0.759	1.094	1.116	0.649
1.160	0.848	0.896	1.083	1.129	0.802	0.932	0.935	1.105	0.814
0.982	0.951	0.950	0.957	1.053	1.048	1.156	0.962	0.937	0.912
0.981	1.413	1.069	0.873	0.843	0.858	0.880	1.097	1.132	1.645
1.173	0.659	0.797	1.207	1.254	1.162	0.826	0.954	1.084	0.639
0.933	1.147	1.139	0.916	0.972	1.098	0.922	1.045	1.042	1.216
1.069	0.996	0.951	1.031	1.066	1.225	0.870	1.065	1.076	1.011
1.030	0.825	0.941	1.072	1.054	1.122	1.053	1.027	0.971	0.765
1.039	1.001	1.001	1.075	1.069	0.777	0.752	1.104	1.067	1.037
1.039	1.089	0.974	0.925	1.083	0.718	0.891	1.179	1.094	1.157
1.075	0.919	0.893	1.124	1.076	1.198	0.814	1.042	1.046	0.926
1.161	0.890	0.953	1.170	1.039	0.874	0.708	1.042	1.117	0.912
1.018	0.970	1.112	1.050	1.024	0.893	0.829	1.035	1.063	1.010
1.226	0.853	0.826	1.086	1.026	0.926	0.944	1.027	1.185	0.810
0.912	1.060	1.088	0.917	0.928	1.041	1.213	0.871	0.896	1.045
1.014	0.978	1.000	1.046	1.027	1.000	0.868	1.046	1.024	1.002
1.033	0.910	0.919	1.012	1.031	1.172	0.948	1.022	0.999	0.888
1.112	0.863	0.912	1.048	1.142	0.820	0.925	0.980	1.069	0.873
1.181	0.872	0.727	1.251	1.166	0.888	0.762	1.115	1.145	0.861

1.006	0.851	0.978	1.047	1.091	1.040	1.017	0.935	0.962	0.850
1.104	0.926	1.009	1.037	1.120	0.962	0.864	0.947	1.095	0.931
0.925	1.120	1.203	0.940	0.912	1.247	0.927	0.950	0.998	1.180
1.111	0.713	0.807	1.109	1.208	1.120	1.006	0.906	1.003	0.632
0.897	1.268	1.305	0.896	0.851	1.021	0.850	1.103	1.071	1.533
1.200	0.870	0.847	1.013	1.119	1.034	0.864	1.045	1.185	0.837
0.828	1.194	1.087	0.845	0.880	1.446	1.254	0.914	0.838	1.238
1.014	1.006	0.962	0.975	0.987	1.047	1.003	1.037	1.010	1.032
0.860	1.049	1.156	0.907	0.946	1.730	1.077	1.053	0.883	1.096
1.104	0.986	1.166	1.085	1.078	0.972	0.658	1.095	1.224	1.039
0.983	1.035	1.040	0.837	0.924	1.464	1.104	1.006	1.081	1.143
1.373	0.806	0.883	0.916	0.941		1.098	1.169	1.421	0.842
1.002	0.782	1.098	0.955	1.126	0.647	1.079	1.028	0.975	0.768
0.989	0.989	1.000	0.949	1.017	0.787	1.076	1.104	0.985	1.052
1.019	0.996	0.990	1.070	0.998	1.073	0.808	1.037	1.040	1.026
0.919	0.898	0.987	1.084	1.199		0.955	1.017	0.870	0.858
1.061	0.857	0.908	0.975	1.036	1.291	0.989	1.201	1.038	0.840
1.304	0.765	0.700	1.223	1.236	0.920	0.706	1.209	1.210	0.721
1.074	0.894	0.850	1.085	1.140	0.973	0.939	0.976	1.023	0.831
1.055	0.877	0.956	1.068	0.955	1.375	1.114	1.066	1.036	0.869
1.184	0.974	0.886	1.097	1.008	0.967	0.854	1.139	1.221	1.010
0.884	1.087	1.138	0.901	0.932	0.963	0.959	1.016	0.937	1.151
1.050	0.819	0.871	1.146	1.084	0.857	0.958	0.998	0.998	0.775
1.031	0.904	0.946	1.180	1.069	1.062	0.831	1.066	1.018	0.902
0.919	1.267	1.079	0.984	0.838	1.169	0.945	0.962	1.028	1.391
0.970	0.833	1.149	1.084	0.964	0.984	1.000	0.918	0.955	0.840
1.272	0.717	0.843	1.120	1.201	0.631	0.750	1.201	1.247	0.710
0.945	0.936	1.001	0.920	0.928	1.112	1.188	0.989	0.949	0.910
1.019	0.960	0.935	1.008	0.975		1.119	1.089	1.004	0.954
1.141	0.670	0.872	1.141	1.190	1.250	0.836	1.046	1.037	0.611
0.744	1.452	1.114	0.793	0.732	1.940	0.833	1.166	0.966	1.634
1.124	0.992	1.079	0.965	0.910	1.134	0.853	1.034	1.135	1.035
1.014	1.038	1.037	1.021	0.961	0.913	0.841	1.101	1.067	1.060
1.082	0.782	0.951	1.091	1.118	1.301	1.012	0.844	0.972	0.709
1.191	1.016	1.075	1.045	0.956	1.446	0.717	1.235	1.294	1.117
1.150	0.990	0.851	1.034	1.076	1.164	0.885	1.188	1.123	0.976
1.044	0.938	1.018	1.056	1.025	1.006	0.784	1.073	1.071	0.957
1.242	0.727	0.789	1.174	1.186	1.312	0.857	1.048	1.151	0.710
0.987	1.026	0.930	1.027	0.983	1.093	1.124	0.917	0.955	0.986
1.211	0.721	0.926	1.085	1.122	0.753	0.906	1.205	1.208	0.698
0.937	0.940	1.014	0.993	1.037	1.172	0.951	1.008	0.952	0.915
0.489	1.424	1.822	0.484	0.431	1.678	1.535	0.651	0.574	1.731
1.018	0.938	0.986	1.036	1.030	0.780	1.015	0.984	0.977	0.955
0.976	1.036	0.916	0.974	1.060	1.475	0.871	1.077	0.988	1.019
1.001	0.806	0.959	1.083	0.996	1.238	1.048	1.058	0.943	0.802
1.031	1.067	1.040	0.933	1.131		0.928	1.039	1.068	1.116
1.035	0.938	1.071	0.951	0.963	1.016	0.946	0.933	1.072	0.934
0.864	1.021	1.152	0.994	0.959	0.892	1.035	1.075	0.865	1.047
0.965	1.025	1.052	0.986	0.985	1.228	0.893	1.054	0.980	1.060
1.066	0.949	1.037	1.130	1.048	1.174	0.784	0.949	1.086	0.942
0.975	1.003	0.967	0.994	0.969	1.227	1.045	1.007	0.977	1.006
0.986	0.782	0.929	1.077	1.057	1.159	0.920	1.042	0.963	0.730
1.050	0.921	0.890	1.106	1.089	0.946	0.996	1.108	0.994	0.886
1.192	0.777	0.743	1.150	1.201	1.044	0.757	1.196	1.150	0.760
0.955	1.058	1.046	1.000	0.979	0.948	1.265	0.866	0.917	1.055

1.121	0.831	0.761	1.174	1.245	1.320	0.790	1.040	1.069	0.780
1.000	0.978	1.144	0.775	1.064	1.054	1.077	1.002	1.028	1.014
1.208	0.891	0.788	0.980	1.125	0.920	0.860	1.216	1.219	0.880
1.071	0.959	1.077	0.965	0.890		0.965	1.287	1.156	1.044
1.059	1.011	0.896	0.950	0.904	1.073	1.045	1.242	1.087	1.047
0.961	0.880	0.961	1.018	1.023	1.244	1.192	0.911	0.890	0.836
1.011	0.956	0.988	0.993	1.147	1.822	0.937	0.931	0.988	0.916
1.045	0.992	1.111	1.023	1.027	1.513	0.750	1.008	1.091	1.020
0.794	0.932	1.112	0.789	1.093	0.727	1.560	0.682	0.710	0.841
0.854	1.248	1.147	0.982	0.905		0.950	1.041	0.917	1.352
1.131	0.796	0.793	1.110	1.234	1.394	0.846	1.012	1.032	0.753
0.956	1.141	1.109	0.997	1.016	0.971	0.855	1.033	1.025	1.235
1.105	0.765	0.900	1.112	1.064	0.919	0.994	1.200	1.058	0.729
0.857	1.000	1.096	0.930	0.944	0.677	1.267	0.961	0.835	0.983
0.909	0.911	1.014	0.973	1.029	1.030	1.247	0.798	0.836	0.845
0.939	0.981	1.048	0.880	0.986	1.185	1.103	0.952	0.947	0.980
0.735	1.282	1.264	0.742	0.671	1.724	1.312	0.798	0.735	1.370
1.155	0.907	0.863	1.086	1.173		0.957	0.903	1.096	0.868
0.875	1.099	1.171	0.941	0.922	1.030	1.095	0.970	0.910	1.104
1.101	0.963	0.964	1.012	0.957	0.981	1.011	1.052	1.109	0.979
1.078	0.886	0.928	1.049	1.071	1.018	1.047	0.936	1.038	0.845
1.042	0.874	1.042	1.058	0.938	0.956	1.056	1.022	1.027	0.869
0.977	1.006	0.975	0.939	0.970	0.816	1.215	1.091	0.988	0.994
1.017	1.075	0.952	1.002	0.941	1.356	0.914	1.082	1.047	1.115
1.102	0.817	0.878	1.061	1.140	0.822	1.045	0.994	1.033	0.781
1.040	0.846	1.026	1.055	1.052	1.154	1.090	0.918	1.004	0.811
0.524	1.411	1.535	0.599	0.572	1.582	1.445	0.706	0.594	1.610
0.959	1.000	0.963	0.926	1.072	1.359	0.977	0.868	0.906	0.972
0.931	1.204	1.196	0.936	0.899	0.792	1.052	1.000	0.968	1.265
0.960	1.087	0.990	0.993	0.972	1.042	1.066	0.984	0.968	1.104
0.963	0.900	1.051	1.048	0.813	1.298	1.075	1.090	0.997	0.882
1.003	0.906	0.926	1.024	1.000	1.035	1.040	1.108	0.992	0.904
0.896	1.184	1.185	0.827	0.876	0.973	0.984	1.130	0.985	1.313
1.449	0.486	0.466	1.565	1.598	1.050	0.424	0.903	1.200	0.406
0.930	0.823	1.065	0.982	1.126	1.097	1.150	0.837	0.854	0.763
0.940	1.006	1.010	1.002	1.029	1.161	0.993	1.017	0.925	0.998
1.077	1.097	1.047	0.880	0.966	0.792	0.914	1.153	1.196	1.224
1.138	0.821	0.770	1.159	1.141	0.852	1.085	0.932	1.033	0.751
1.150	1.082	1.205	1.076	0.813		0.843	0.995	1.295	1.229
1.361	0.747	0.717	1.045	1.204	0.936	0.884	1.111	1.290	0.729
1.047	0.874	0.859	1.090	1.141	1.091	0.881	1.078	0.982	0.820
0.966	0.967	0.777	1.377	0.911	1.250	1.023	0.764	0.862	0.870
1.165	0.700	0.825	1.167	1.175	0.987	0.865	1.077	1.082	0.656
0.899	0.980	1.078	0.906	0.890	1.172	1.309	0.858	0.882	0.957
1.094	0.858	0.949	1.039	0.974	0.700	1.091	1.059	1.071	0.847
0.912	0.966	1.130	1.065	0.928		1.101	1.060	0.919	0.984
0.948	1.039	1.189	0.961	0.948	0.925	0.846	1.204	1.019	1.187
1.002	0.930	1.077	1.019	0.939	0.977	1.070	0.981	1.033	0.930
0.849	1.173	1.108	0.865	0.833	1.286	1.184	0.919	0.874	1.235
0.803	1.014	1.242	0.882	0.819	0.894	1.461	0.761	0.749	0.954
1.121	0.624	0.713	1.297	1.279	0.845	0.809	1.212	1.053	0.579
1.104	0.713	0.908	1.202	1.148	1.312	0.836	0.989	0.967	0.660
1.108	0.910	0.928	1.084	1.045	1.142	0.975	1.021	1.051	0.895
1.013	1.097	1.105	1.010	0.917	0.860	0.890	1.063	1.078	1.187
1.017	1.005	0.916	0.903	1.044	0.768	1.080	1.151	1.018	1.015

1.147	0.844	0.897	1.094	1.020	1.350	1.021	0.990	1.126	0.836
1.042	0.775	0.889	1.033	1.183	0.871	1.165	0.968	0.941	0.707
1.133	1.008	0.923	1.035	1.094	0.771	0.868	1.051	1.147	1.016
1.123	0.931	1.001	1.052	0.987	1.023	0.814	0.867	1.157	0.951
1.333	0.840	0.799	1.117	1.052		0.970	1.065	1.318	0.838
0.983	1.217	0.919	0.917	0.955		1.093	1.047	1.011	1.263
1.224	0.724	0.801	1.145	1.123	1.165	0.943	1.067	1.121	0.702
0.984	1.157	0.922	1.149	0.906	0.792	0.945	0.960	0.985	1.170
1.059	0.804	0.806	1.132	1.202	0.902	0.946	1.052	0.969	0.743
0.916	1.170	1.204	0.834	0.913	1.196	0.878	1.222	1.032	1.331
1.117	0.919	1.071	1.089	1.129	0.836	0.784	1.103	1.154	0.963
0.975	1.164	1.065	0.904	0.923	1.125	1.081	0.901	1.003	1.223
1.005	0.773	1.044	1.106	1.061	0.995	1.009	0.966	0.948	0.736
1.072	0.911	0.846	1.077	1.101	1.143	1.100	0.829	0.978	0.839
1.143	0.876	0.899	1.076	1.054	0.992	0.970	1.071	1.148	0.856
1.021	0.992	0.966	1.038	0.978	0.996	1.023	1.048	1.038	1.007
1.009	1.012	0.942	1.039	0.984	1.195	0.987	1.073	1.001	1.004
1.255	0.739	0.833	1.176	1.176	0.846	0.797	1.048	1.193	0.708
1.230	0.696	0.789	1.050	1.185	1.216	0.784	1.097	1.225	0.635
0.891	1.245	1.254	0.918	0.895	1.444	0.988	1.000	0.953	1.366
0.907	1.255	1.123	0.861	0.781	0.865	1.037	0.964	1.013	1.394
0.766	1.088	1.091	0.758	0.776	1.246	0.859	2.299	0.942	1.351
0.994	0.976	0.909	0.969	0.928	1.391	1.231	0.979	0.948	0.939
1.181	1.298	1.079	0.987	1.048		0.516	1.065	1.391	1.543
0.952	0.988	1.177	0.959	0.879	1.321	1.034	1.139	1.008	1.055
0.964	0.794	0.942	1.008	1.086	1.728	1.128	0.972	0.879	0.730
0.924	1.241	1.151	0.871	0.901	1.220	0.902	1.087	1.022	1.385
1.184	0.827	0.880	1.121	1.088	0.963	0.811	1.080	1.164	0.821
0.874	0.866	0.776	1.168	1.455		1.128	0.722	0.722	0.721
1.154	0.845	0.943	0.998	1.056		1.141	1.015	1.127	0.832
0.901	0.666	1.234	0.949	0.936	1.337	1.196	0.927	0.861	0.642
0.812	1.313	1.203	0.815	0.954	1.058	0.886	0.983	0.898	1.466
0.933	1.036	0.995	0.928	1.169	2.166	0.761	1.069	0.928	1.040
0.885	1.021	1.059	0.922	0.933	1.177	1.073	1.185	0.902	1.051
1.523	0.660	0.543	1.281	1.179		0.793	1.207	1.443	0.631
1.062	1.255	0.908	0.939	0.947		1.130	0.938	1.101	1.313
1.179	0.847	0.594	1.191	1.276	0.626	0.835	1.138	1.078	0.782
1.086	0.818	0.938	1.088	1.041	1.289	0.836	1.181	1.070	0.813
1.089	0.682	0.728	1.150	1.144	0.957	1.117	1.088	0.962	0.608
0.988	1.104	0.952	0.974	1.121	0.989	0.800	0.840	1.025	1.086
0.450	2.300	1.538	0.470	0.474	0.710	1.106	0.846	0.673	3.467
1.318	0.852	0.680	1.289	1.379		0.589	0.898	1.217	0.794
1.032	1.035	0.923	1.080	0.961	1.206	0.976	0.969	1.014	1.026
1.009	0.939	1.131	0.995	1.064		0.899	1.079	1.041	0.977
1.021	1.176	0.800	0.810	0.842	0.700	1.577	0.859	0.977	1.135
0.837	0.684	1.063	1.056	1.051	1.701	1.008	1.210	0.823	0.658
0.871	1.015	1.126	0.963	0.914	1.440	1.171	0.880	0.848	0.997
1.065	0.734	1.007	1.042	1.048	0.814	1.131	0.992	1.001	0.696
1.088	1.056	0.966	0.909	0.919	1.251	0.927	1.279	1.169	1.145
1.638	0.687	0.650	2.035	0.601	1.639	0.582	0.643	1.478	0.626
1.105	0.866	0.959	0.981	1.041	1.021	0.831	1.032	1.103	0.924
0.654	1.353	1.312	0.534	0.747	1.410	1.661	0.692	0.672	1.403
0.925	1.008	1.017	0.954	1.136		1.180	0.872	0.883	0.970
1.044	0.890	1.016	1.132	0.872	1.162	0.966	1.129	1.063	0.901
0.884	0.991	1.098	1.033	1.283		0.883	0.893	0.854	0.966

1.063	0.991	0.955	0.901	1.071	1.131	1.401	0.716	0.994	0.916
0.961	1.351	1.021	0.878	0.941	0.625	1.112	0.791	1.000	1.420
0.917	1.078	1.051	0.956	0.955	1.493	1.093	0.984	0.944	1.091
1.261	0.818	0.719	1.055	1.226	1.253	0.890	1.007	1.177	0.771
1.013	1.177	1.077	1.037	0.872	0.736	0.906	1.025	1.086	1.274
0.986	0.889	1.313	0.882	1.066		0.907	1.167	1.072	0.976
0.990	0.930	0.786	1.133	1.049	1.078	0.998	1.017	0.905	0.912
1.031	0.890	0.895	1.155	0.855	1.182	0.845	1.514	1.069	0.932
0.952	0.785	0.999	0.992	1.000	0.627	1.312	1.000	0.884	0.735
1.006	0.873	0.804	1.118	1.192	0.685	1.097	0.899	0.897	0.785
0.882	1.177	1.005	0.942	0.844	1.263	1.036	1.228	0.930	1.251
1.066	1.040	0.961	0.931	0.974	1.168	1.006	1.085	1.091	1.074
1.097	0.892	0.897	1.091	1.013	1.231	1.016	0.974	1.046	0.859
0.822	1.299	1.197	0.697	0.790		1.180	1.427	0.971	1.547
0.765	1.172	1.233	0.742	0.904	1.973	1.177	0.922	0.787	1.218
0.778	1.172	1.292	0.842	0.877	1.197	1.175	0.837	0.805	1.224
1.020	0.921	0.972	1.035	1.056	1.325	0.932	1.024	1.016	0.895
1.073	1.069	0.974	0.775	1.055	1.056	1.059	1.083	1.113	1.118
1.047	0.862	0.757	1.094	1.141	0.788	1.213	0.883	0.908	0.758
1.106	0.851	0.898	1.095	1.081	0.969	0.928	1.024	1.054	0.825
0.870	1.284	1.037	0.611	1.019		1.399	0.984	0.906	1.350
1.005	0.896	0.889	1.108	1.104	1.227	0.905	0.972	0.966	0.846
1.026	1.000	1.099	0.988	0.851	1.486	0.833	1.328	1.121	1.103
1.035	0.949	1.067	0.981	0.951	1.024	0.969	0.879	1.052	0.935
1.297	0.985	1.049	0.903	0.924	0.656	0.943	1.081	1.437	1.101
1.021	0.882	0.984	0.980	0.987	1.375	0.958	1.252	1.030	0.898
0.933	1.049	1.011	0.905	0.925	0.641	1.373	0.848	0.896	1.016
1.137	0.845	0.781	1.066	1.120	0.755	1.028	1.220	1.097	0.823
1.007	0.933	1.063	1.016	1.031	1.007	0.921	1.069	1.014	0.948
0.960	1.194	0.683	0.994	0.919		1.620	0.735	0.856	1.075
1.056	0.886	0.987	1.066	1.001	1.487	0.993	1.031	1.072	0.872
0.998	0.959	0.917	1.007	1.037	0.939	1.189	1.016	0.963	0.934
1.081	0.784	1.061	1.103	1.050	1.025	0.872	1.066	1.066	0.780
1.388	0.449	0.930	1.139	1.858		0.558	0.603	1.189	0.389
1.084	0.924	0.791	1.159	1.000	0.720	1.084	1.008	1.015	0.874
0.947	0.839	1.225	0.938	1.280		1.176	0.580	0.869	0.777
0.929	1.063	0.975	0.981	0.987	0.925	1.110	1.001	0.931	1.077
1.052	1.113	1.024	0.892	0.953		1.076	1.021	1.101	1.175
0.938	1.200	1.007	0.927	0.941	1.150	1.091	0.903	0.948	1.224
0.918	1.053	1.051	0.986	0.888	1.904	0.920	1.180	0.949	1.098
0.904	1.058	1.142	0.970	0.919	0.924	1.121	0.958	0.921	1.087
1.072	0.874	0.908	1.093	1.029	1.208	0.964	1.060	1.032	0.849
1.000	1.073	1.013	0.987	0.881	0.785	1.071	1.088	1.031	1.116
1.082	1.108	0.944	1.006	0.979	1.110	0.858	1.095	1.130	1.168
0.956	1.073	1.270	1.026	0.816		0.813	1.256	1.089	1.234
0.970	0.928	0.919	1.134	1.025	0.792	0.927	1.191	0.951	0.919
1.075	0.993	0.829	1.121	1.142		0.947	1.030	1.038	0.966
1.000	1.076	1.181	0.904	0.827		0.819	1.109	1.097	1.185
1.088	0.817	1.040	1.146	0.915	1.727	0.846	1.075	1.097	0.827
0.846	0.638	1.603	0.864	1.001	1.840	0.843	1.149	0.900	0.685
0.744	0.736	2.077	0.800	0.172		1.948	0.529	0.815	0.814
1.007	0.879	1.061	1.077	1.023	0.801	0.917	1.055	1.004	0.866
1.062	0.834	1.040	1.114	0.917	1.984	0.870	1.090	1.054	0.835
0.996	1.045	1.173	0.995	0.968	1.061	0.988	0.919	1.060	1.110
1.011	1.050	0.853	1.051	1.042		1.199	0.865	0.949	0.995

0.578	1.468	1.452	0.560	0.583	0.983	1.537	0.821	0.657	1.685
0.914	0.920	0.967	1.063	0.844	1.434	0.947	1.488	0.942	0.958
0.886	1.252	1.011	0.885	0.909	1.419	1.195	0.853	0.880	1.279
0.921	1.228	1.015	0.857	0.834	0.967	1.103	1.033	0.958	1.289
1.039	0.924	0.993	1.084	1.049	1.285	0.851	1.055	1.028	0.922
1.005	0.989	1.065	1.007	0.979	0.944	0.978	0.971	0.969	0.963
1.150	0.801	0.908	1.119	0.982		0.965	1.241	1.146	0.805
1.118	1.069	0.898	1.116	1.106	0.750	0.571	1.218	1.179	1.138
1.252	0.840	0.822	1.170	1.197		0.751	1.134	1.236	0.837
0.846	1.038	1.123	0.811	0.974	0.639	1.354	0.908	0.827	1.026
1.214	0.741	0.771	1.149	1.203	0.761	0.934	1.004	1.096	0.678
1.048	0.949	0.958	1.045	0.972	0.902	1.072	1.012	0.926	0.951
1.018	1.054	0.933	0.830	1.066	0.882	1.139	1.033	1.014	1.060
0.908	0.769	1.083	1.029	0.899	0.984	1.466	0.776	0.823	0.698
1.088	0.783	0.864	1.172	1.194		0.935	0.992	1.002	0.728
1.100	1.065	0.813	1.138	1.061	0.870	0.780	1.115	1.103	1.077
0.877	0.715	1.253	1.021	0.915	1.121	1.063	1.127	0.873	0.718
1.028	0.974	0.923	0.969	1.006	1.031	1.105	1.023	1.002	0.957
0.863	1.013	1.157	0.819	0.792	1.428	1.357	1.014	0.870	1.030
0.917	1.105	1.154	0.840	0.930	1.172	1.073	1.030	0.963	1.171
0.915	1.228	1.167	0.862	0.800	0.894	1.014	1.197	1.036	1.402
1.092	1.045	0.881	0.958	1.009	1.020	0.937	1.192	1.124	1.085
0.806	0.729	1.151	0.882	0.987	3.232	1.159	1.005	0.742	0.677
1.009	1.088	1.156	1.068	0.938	1.030	0.781	0.998	1.076	1.170
1.115	0.983	0.849	1.062	1.153	0.871	0.859	1.015	1.087	0.967
0.996	1.007	0.946	0.996	0.985	0.979	1.098	1.010	0.974	0.993
1.017	0.869	1.068	1.069	1.037	0.784	0.839	1.206	1.038	0.895
0.960	1.164	0.941	0.916	0.990	0.931	1.120	0.943	0.956	1.170
1.083	0.866	0.991	1.042	1.068	1.711	0.800	1.057	1.073	0.865
1.045	0.930	1.066	1.059	1.015	0.919	0.806	1.021	1.072	0.985
1.018	1.174	1.135	0.991	1.112	1.493	0.550	0.996	1.107	1.288
1.176	0.766	0.693	1.288	1.146		0.825	1.190	1.092	0.718
0.953	1.323	1.085	0.781	0.838		1.250	0.997	1.045	1.464
1.047	0.980	1.045	0.858	1.022	0.839	1.160	0.931	1.044	0.987
1.092	0.828	0.799	1.129	1.193	0.680	0.795	1.258	1.056	0.808
1.085	1.093	0.896	1.029	1.044	1.101	0.879	1.002	1.093	1.111
1.030	0.841	1.003	1.056	0.931	1.178	0.792	1.553	1.095	0.902
1.104	0.943	0.958	0.977	1.066	1.006	0.997	1.005	1.139	0.956
0.773	1.403	1.366	0.797	0.813	1.093	1.010	0.923	0.885	1.619
0.852	1.207	1.038	1.033	0.965	0.775	1.072	0.844	0.841	1.202
0.898	1.141	1.186	0.943	0.898	2.059	0.820	1.238	0.999	1.282
0.438	2.137	1.629	0.507	0.488	1.207	1.157	0.574	0.604	2.973
1.119	0.826	0.838	1.279	0.908	0.746	1.231	0.778	0.998	0.743
1.679	0.530	0.777	0.871	1.136		0.789	1.569	1.824	0.582
1.103	0.971	0.941	0.910	1.271		1.066	0.821	1.055	0.937
0.968	0.995	0.908	1.024	1.028	0.720	1.096	1.049	0.934	0.969
1.113	0.748	0.903	1.142	1.106	1.389	0.917	1.081	1.065	0.723
1.014	1.015	0.858	0.956	1.169	1.184	0.891	1.133	0.996	1.007
1.070	1.028	1.017	0.984	1.035	1.370	0.860	1.068	1.111	1.077
1.005	1.059	1.051	0.988	0.912	1.178	1.097	0.916	1.018	1.082
1.015	0.998	0.958	0.910	1.190		1.146	0.887	0.974	0.966
1.070	0.858	0.886	1.024	1.086	1.133	1.024	0.969	1.043	0.833
1.042	0.856	0.842	1.063	1.095	1.613	1.008	1.026	0.966	0.801
1.026	0.801	0.949	1.026	1.116		1.085	1.033	0.968	0.763
1.204	0.839	0.822	1.097	1.061	0.920	0.933	1.065	1.148	0.824

0.938	0.931	0.952	0.995	0.963	0.853	1.095	1.258	0.934	0.936
1.090	0.852	0.902	1.083	1.145	0.776	0.816	1.157	1.086	0.856
1.009	1.049	1.178	1.005	0.963	0.945	0.780	1.081	1.091	1.144
0.843	1.112	1.070	0.921	1.043		1.066	1.148	0.867	1.155
0.714	1.970	0.433	0.609	0.489	1.038	2.577	0.342	0.631	1.732
0.971	1.020	0.945	0.967	1.036	0.836	1.112	0.969	0.943	0.999
1.105	0.874	0.832	1.199	1.064	0.835	0.922	1.032	1.043	0.833
0.896	1.344	1.226	0.886	0.867	0.911	0.894	0.992	0.995	1.541
0.962	0.935	1.006	0.993	0.889	1.292	1.214	0.985	0.927	0.910
1.064	1.113	1.038	0.807	0.911	0.957	1.141	1.024	1.124	1.186
0.890	1.138	0.804	0.923	1.052	1.937	1.166	0.922	0.827	1.067
1.104	1.032	1.029	1.008	0.988	0.930	0.866	1.048	1.154	1.088
1.401	0.699	0.724	1.834	0.769		0.833	0.683	1.236	0.622
1.135	0.819	1.018	0.990	0.932	1.591	0.887	1.288	1.179	0.859
0.870	1.337	0.898	0.903	1.219	0.976	0.873	0.902	0.867	1.344
1.249	0.725	0.824	1.201	0.994	1.242	0.887	1.118	1.195	0.700
0.899	0.911	1.043	0.916	0.953	1.535	1.348	0.844	0.833	0.851
0.899	1.089	0.961	1.004	1.053	0.807	1.186	0.881	0.830	1.015
1.113	0.879	0.763	1.130	1.069	0.900	1.095	0.956	1.020	0.812
0.772	1.096	1.175	0.857	0.733	0.721	1.548	0.875	0.759	1.088
1.143	0.848	0.944	1.087	1.089	1.905	0.951	1.149	1.112	0.833
0.859	1.180	1.119	0.891	0.886		0.955	1.315	0.960	1.331
1.018	0.901	0.946	1.046	0.987	1.467	1.154	0.989	0.973	0.892
0.911	0.985	1.271	0.865	0.818	0.986	1.228	0.969	0.946	1.033
1.036	0.775	0.977	1.066	1.284	0.966	0.847	0.980	0.967	0.730
1.020	0.702	0.972	1.138	1.088		1.186	0.987	0.932	0.647
0.758	0.925	1.044	0.763	0.677	0.930	2.099	0.738	0.666	0.820
0.912	1.022	1.195	0.947	0.948	0.838	1.034	0.997	0.940	1.064
1.049	0.678	1.033	1.094	1.056		0.931	1.280	1.033	0.673
0.600	1.390	1.392	0.709	0.672		1.531	0.803	0.645	1.507
1.111	0.924	1.181	0.932	1.395		0.949	0.465	1.049	0.880
1.092	1.058	0.646	1.067	1.078	1.024	1.092	0.984	1.013	0.991
0.975	1.142	1.201	0.887	0.900	0.727	0.941	1.090	1.080	1.276
1.042	0.937	0.817	1.046	1.006	1.356	0.997	1.195	1.011	0.918
1.158	0.924	0.712	1.164	1.217	1.270	0.769	1.022	1.083	0.872
0.921	1.033	1.016	0.955	0.977	0.907	1.150	0.977	0.903	1.022
1.157	0.746	0.827	1.308	0.982	0.851	0.924	1.090	1.083	0.704
0.807	1.628	0.810	0.790	0.802	0.727	1.473	0.764	0.811	1.650
0.993	0.823	1.034	0.940	1.072		1.129	1.100	0.965	0.808
1.145	0.913	0.756	0.971	1.103		1.062	1.236	1.123	0.904
1.104	0.940	0.898	1.116	0.974	1.044	1.032	0.937	1.060	0.911
0.840	1.097	1.172	0.869	0.864	1.023	1.292	0.870	0.840	1.107
1.101	0.880	0.977	1.028	1.088	0.994	0.847	1.186	1.118	0.902
0.965	1.178	0.769	1.017	0.927	0.924	0.978	1.350	0.992	1.222
1.622		3.331							
1.095	0.777	0.789	1.130	1.119	0.973	0.945	1.020	1.002	0.729
1.326	0.840	0.779	1.065	0.932	1.179	0.644	1.748	1.474	0.942
1.060	1.037	1.155	0.765	0.960		1.283	0.900	1.107	1.093

A4/B	A1/B1	A2/B2	A3/B3	A4/B4	# AAs	MW [kDa]	calc. pI
1.091	1.039	0.914	0.894	0.904	589	63.1	5.52
0.910	1.074	0.900	1.266	0.896	428	46.7	4.88
1.000	1.026	0.959	0.871	0.842	540	57.7	5.25
1.168	0.996	1.004	1.446	1.489	396	43.5	5.08
0.958	1.014	0.925	1.246	0.748	338	36.6	5.96
0.966	1.120	0.832		0.996	428	46.7	4.91
1.049	1.082	1.022	1.266	1.084	589	63.1	5.52
1.581	0.938	0.994	**2.216**	**2.207**	266	29.6	4.93
0.833	1.038	0.967	0.878	0.850	380	39.7	6.30
0.626	**0.385**	1.812	**0.158**	**2.075**	353	36.7	6.19
1.049	1.171	1.056	1.181	0.831	338	36.5	5.96
1.123	1.005	0.905	**0.427**	1.368	232	25.4	4.65
1.060	1.029	0.955	0.982	0.959	403	42.8	5.55
0.900	1.045	0.970	1.232	0.784	303	33.3	5.39
1.117	1.201	1.040	0.633	1.031	396	43.6	5.03
0.901	0.883	0.837		0.831	254	28.1	4.74
0.953	1.068	0.991	0.773	0.921	303	33.3	5.39
1.068	1.046	1.070	1.357	1.021	264	29.2	4.88
0.862	0.963	1.047	0.825	0.850	380	39.7	6.14
0.996	1.016	1.021	1.252	0.896	230	26.5	5.30
1.482	1.021	0.957	1.090	1.054	711	79.2	6.79
1.851	1.226	0.899		1.660	711	79.2	7.08
1.151	1.031	0.989	1.183	1.142	697	76.8	5.06
1.047	1.425	0.874		1.020	380	39.7	6.14
1.100	0.942	1.014	1.067	1.246	912	104.6	5.80
0.601	**0.453**	**2.872**	**2.096**	1.169	246	27.1	4.74
1.093	1.114	0.838	0.661	0.989	163	17.6	5.00
0.834	1.068	1.051	0.629	1.068	230	26.6	5.63
1.131	1.004	0.931	0.754	1.514	411	45.1	6.18
1.038	1.063	0.922	1.129	0.921	844	95.7	4.94
1.660	1.028	0.965	1.125	1.251	743	81.8	5.29
1.045	1.041	0.903	**2.012**	1.069	844	95.7	4.97
0.906	1.019	0.972	1.035	0.769	647	73.0	5.02
0.987	1.002	0.942	1.193	0.812	799	91.2	5.72
0.940	1.002	0.971	1.220	0.925	282	30.7	7.58
1.404	1.055	0.973	0.477	1.415	323	35.1	5.21
1.459	1.209	0.999		1.067	323	35.0	5.21
1.501	1.016	0.958	1.340	1.065	744	83.1	6.51
0.587	1.085	1.049	0.916	0.749	450	48.0	9.48
0.923	0.962	0.978	0.919	0.893	707	78.6	5.66
0.934	0.939	0.955	1.454	0.659	445	50.6	5.27
1.186	1.080	0.945	1.145	0.952	608	65.7	5.17
0.836	1.032	0.951	1.749	0.728	449	51.3	5.60
1.094	0.990	1.003	1.003	1.084	1217	135.6	6.62
1.189	1.077	0.871	1.286	0.979	120	12.5	4.50
1.017	1.054	0.965	0.905	0.837	252	27.6	4.91
0.862	0.987	0.975	1.029	0.775	479	52.2	4.91
0.902	1.148	0.919		0.840	445	50.6	5.27
1.023	0.930	0.892	1.082	0.890	574	64.3	5.58
1.044	1.020	0.985	1.005	0.808	320	34.3	5.87
0.856	1.316	0.796	0.735	0.613	798	91.1	5.81
0.863	1.007	0.978	0.996	0.735	446	49.6	5.08
1.216	1.017	1.029	1.850	0.959	722	81.4	8.72
0.999	1.095	1.081	0.826	1.038	1208	134.7	6.70

1.475	1.259	0.797		2.003	411	45.1	6.54
0.906	0.973	0.967	1.240	0.756	449	51.4	5.60
1.215	0.988	0.925	1.024	1.073	324	35.5	6.58
0.800	1.004	0.985	0.904	0.759	446	48.8	5.30
1.311	0.948	0.990	1.179	1.404	603	66.1	5.76
1.879	1.063	0.960	1.438	1.332	432	49.1	5.99
1.130	1.032	0.984	1.693	1.000	574	64.3	5.66
1.155	1.006	0.987	1.232	0.967	644	73.4	5.36
1.175	1.008	0.991	1.263	0.964	499	57.6	6.09
1.290	1.016	0.942	1.150	0.893	238	27.4	5.10
1.502	0.980	0.975	1.059	1.086	1062	117.3	4.97
1.501	1.001	0.713	1.617	1.261	619	66.7	5.17
1.159	1.005	1.024	1.309	1.068	257	28.7	4.97
1.167	0.872	1.093	2.934	0.962	722	81.5	8.75
0.799	1.068	0.978	1.700	0.779	793	90.5	5.86
1.138	1.036	0.987	0.874	0.908	307	32.2	5.15
1.018	1.026	0.954	0.860	0.832	577	63.7	4.97
0.825	1.021	0.912	1.004	0.777	437	50.3	5.26
1.023	0.912	1.074	0.220	0.871	337	37.7	5.44
0.864	1.029	0.988	1.355	0.809	337	37.7	5.77
0.953	0.997	1.026	0.957	1.323	341	38.0	7.06
1.149	1.024	0.996	1.138	1.156	647	73.5	5.12
0.839	1.060	1.102	1.642	0.798	337	37.7	5.64
0.726	1.020	0.854	0.937	0.945	121	13.3	5.49
1.134	1.051	0.958	1.114	1.052	341	37.7	5.58
0.860	0.991	0.956	1.020	0.768	793	90.4	5.80
1.118	0.983	1.006	1.293	1.104	1213	135.6	5.77
0.912	1.025	0.918	0.652	0.932	337	37.8	5.94
0.888	0.963	0.973	1.093	0.749	503	54.9	5.26
1.213	0.896	1.013	1.096	1.536	467	52.1	5.40
1.145	0.956	1.051	1.104	0.969	180	20.3	9.04
1.174	0.998	0.962	1.127	1.034	687	78.5	5.20
1.227	0.982	0.966	1.183	0.957	516	57.6	5.90
0.980	0.978	0.995	1.194	1.053	658	75.1	6.43
1.396	1.046	0.926	1.231	0.741	429	47.4	5.92
1.267	1.208	0.957	1.778	1.083	324	35.5	6.95
0.615	0.954	0.980	0.473	0.672	155	17.8	4.91
1.700	1.085	1.015	1.266	1.509	403	44.3	5.26
1.264	1.023	1.008	1.026	0.956	212	23.1	9.94
1.042	0.959	0.963	0.808	0.987	368	41.2	4.93
0.698	1.118	1.169	1.505	0.759	449	47.9	9.33
1.131	1.060	0.945	0.991	0.973	120	12.5	4.50
1.162	0.947	1.022	1.172	0.910	156	17.8	9.88
0.939	0.982	0.999	0.809	0.812	308	33.2	5.07
0.976	1.001	0.991	0.903	0.791	513	55.3	6.49
1.387	0.999	0.942	1.208	1.018	205	22.5	10.30
0.999	1.017	0.990	0.846	0.828	467	51.0	5.60
0.804	0.995	0.929	0.709	0.724	155	17.8	4.84
0.850	1.011	0.970	0.897	0.845	474	53.4	4.97
0.920	0.899	0.956	0.542	0.915	438	50.0	5.40
1.248	0.938	0.899	0.298	1.516	479	51.8	5.87
1.224	1.072	1.083	1.029	1.311	870	97.2	9.57
0.986	1.083	1.031	1.061	0.972	818	85.9	9.85
1.074	0.994	1.014	1.201	1.143	927	106.1	5.66
1.357	0.973	1.008	1.087	1.081	230	24.8	8.91

0.872	1.213	1.285	0.484	0.896	479	51.8	5.87
0.906	0.979	1.024	1.056	0.862	598	68.1	5.30
1.008	1.001	0.969	1.133	0.915	318	35.1	4.91
0.823	0.990	0.949	0.986	0.834	91	9.8	9.57
1.019	1.277	1.187	0.545	1.248	148	16.4	7.24
0.908	1.190	0.947	0.798	0.774	438	50.0	5.47
1.502	1.010	0.953	1.045	1.354	553	60.1	8.50
0.816	0.975	1.003	1.563	0.988	369	41.1	5.05
1.213	0.975	0.992	2.188	0.845	307	32.2	5.47
0.780	1.029	1.033	1.020	0.683	1843	198.6	8.59
1.191	0.939	0.975	2.253	0.874	479	51.8	6.05
1.029	1.013	0.992	1.274	0.973	432	50.2	5.55
1.317	0.947	0.989	1.039	1.038	425	46.3	5.86
0.527	1.111	1.127	0.721	0.652	482	52.1	9.41
1.117	0.989	1.009	1.045	1.070	458	49.6	8.16
0.776	1.135	0.937		1.064	121	13.4	5.49
1.122	0.991	1.009	1.245	1.010	234	25.2	6.80
0.772	1.042	0.995	0.988	0.787	103	11.7	4.88
0.919	0.943	0.928	1.202	0.931	804	92.3	6.21
1.200	1.007	0.986	0.974	1.023	180	20.0	6.71
1.537	0.956	0.958	1.136	1.173	335	38.2	4.89
1.101	0.956	0.993	1.009	1.476	334	35.1	5.11
0.612	0.972	1.055	1.305	0.599	121	13.3	5.86
1.038	0.977	1.021	0.946	1.113	461	50.1	5.86
0.977	0.970	0.997	2.599	0.890	804	92.3	6.14
1.379	1.005	0.970	1.233	0.927	381	42.3	6.58
0.694	1.082	0.957	1.803	0.651	369	41.0	5.12
1.231	0.976	0.994	1.370	1.168	516	59.7	5.24
1.260	1.026	1.005	1.101	1.035	168	18.0	10.35
1.840	1.040	0.949	1.307	1.235	425	46.3	6.05
1.130	1.048	0.999	1.226	1.044	452	50.4	5.03
1.260	1.000	0.973	1.034	1.052	207	23.0	6.81
0.750	1.008	0.994	0.834	0.950	269	29.3	5.30
1.295	0.946	1.013	1.040	1.516	879	98.2	5.58
0.747	1.069	1.020	1.226	0.839	648	73.5	5.21
0.845	1.101	0.983		0.741	818	85.9	9.83
0.909	0.918	1.010	1.219	1.215	761	87.3	6.14
1.238	1.013	0.998	3.170	0.906	617	70.2	5.99
1.039	1.075	0.972	1.326	0.923	311	34.3	4.87
0.931	1.015	0.989	1.526	0.908	617	70.2	5.81
2.272	0.944	1.118	3.076	1.738	553	60.1	8.72
1.446	1.009	0.987	1.184	1.213	373	41.1	6.74
1.166	0.964	0.910	0.509	0.820	307	32.1	5.24
0.669	1.044	0.953	1.054	0.877	134	14.6	4.68
0.987	1.008	1.021	1.134	1.193	439	46.6	4.82
0.669	1.063	0.981	1.090	0.745	648	73.6	5.25
0.809	0.955	1.031	1.112	0.959	420	47.5	5.71
1.131	1.073	0.829	1.065	0.867	427	46.4	5.76
0.898	1.092	0.918	0.807	0.791	116	13.0	4.86
1.628	1.043	0.938	1.025	1.026	418	46.1	5.63
1.109	1.009	1.152	1.130	1.294	334	35.0	5.25
1.011	1.020	1.001	1.144	0.865	879	101.3	5.76
0.882	0.910	0.987	0.983	0.978	154	16.8	8.00
1.410	1.043	1.008	0.940	0.992	278	30.3	10.43
0.950	1.003	0.986	1.245	0.876	435	49.4	5.64

1.274	1.053	0.968	0.991	0.958	170	18.6	4.83
1.028	1.180	0.905	1.519	0.863	311	34.3	4.92
0.933	0.969	0.980	0.998	0.928	215	25.1	5.39
1.155	0.946	0.978	0.866	0.954	561	63.2	5.76
1.015	0.978	0.979	1.107	0.984	312	35.0	4.89
1.995	1.038	1.065	1.229	1.547	207	23.1	6.54
1.380	0.938	0.909	1.384	1.453	879	98.1	5.58
1.222	0.989	0.997	1.130	0.981	203	23.4	9.76
0.726	1.776	1.562	0.484	0.743	464	49.2	9.76
1.047	0.984	0.988	1.398	0.961	427	50.3	6.20
0.816	0.798	0.930		1.103	761	87.3	6.23
0.930	1.034	0.929	0.740	1.032	504	56.9	5.22
0.964	0.981	0.987	1.022	0.892	339	36.6	5.39
0.833	1.041	0.940	0.796	0.793	454	50.1	5.92
0.488	0.816	0.954		0.848	648	73.7	5.19
1.197	1.004	1.017	1.165	1.138	318	36.9	6.25
1.105	0.973	1.001	1.014	0.910	804	89.3	5.38
1.123	0.999	1.051	1.026	0.948	132	14.5	9.33
1.047	0.976	0.989	0.990	0.989	553	59.7	4.64
0.876	1.031	1.007	1.116	0.819	475	54.1	4.86
1.354	1.026	0.981	1.014	0.937	146	15.8	10.35
0.868	0.952	0.999	0.916	0.791	589	65.8	9.54
0.773	0.887	0.996	0.730	0.854	357	37.7	9.82
1.122	0.990	0.982	1.550	0.966	487	55.4	6.09
0.927	1.022	1.005	1.049	0.838	473	54.9	5.19
0.853	0.995	0.988	1.080	0.911	467	51.4	4.89
1.127	0.976	0.999	1.317	1.066	565	63.2	5.49
1.068	1.028	1.006	1.120	0.882	476	53.8	6.57
1.251	0.924	0.993	1.139	1.621	388	42.5	5.74
0.889	0.941	0.979	0.812	0.912	427	47.1	9.74
0.992	0.989	0.966	1.464	0.873	276	30.4	7.37
0.829	0.940	1.172	0.756	0.874	115	12.2	8.44
0.921	1.053	1.017	1.369	1.053	827	92.0	6.46
1.017	1.043	1.038	1.049	1.131	215	23.4	4.86
1.332	0.960	1.012	1.160	1.381	393	45.2	6.27
1.088	1.029	0.994	1.066	0.935	523	57.6	6.23
0.725	2.000	1.585	0.513	1.032	376	40.5	10.04
0.984	0.926	1.048	2.023	1.069	520	59.2	6.64
1.423	0.918	1.011	1.239	1.737	539	60.5	5.82
1.129	1.000	1.103	0.972	1.476	520	59.1	6.64
1.571	1.004	0.945	1.111	1.122	223	24.4	5.25
2.030	1.073	0.985	1.145	1.465	477	52.7	6.39
0.852	0.993	1.053	2.226	0.896	449	49.6	7.08
0.844	0.933	0.926	0.349	0.851	793	90.4	5.67
0.865	0.992	1.016		0.662	116	13.0	4.94
1.122	0.987	1.008	1.061	1.337	241	25.9	9.09
0.959	1.066	0.966	0.990	0.840	307	32.9	4.97
1.024	0.912	1.002	1.150	1.154	333	37.0	5.62
0.899	1.064	0.932	1.019	0.875	394	43.8	5.97
1.066	1.001	0.905	1.618	0.929	487	55.4	5.91
1.277	0.972	0.993	1.248	1.198	614	68.5	5.50
1.123	1.049	0.985	1.021	1.140	455	50.5	5.47
0.933	0.941	0.990	1.012	0.941	467	52.8	5.62
1.300	0.955	1.018	1.206	0.924	147	16.5	9.52
0.921	1.049	1.002	0.836	0.942	88	9.4	4.84

1.169	0.996	0.983	1.137	1.068	393	43.6	4.61
0.952	1.014	0.982	0.941	0.960	232	26.1	6.55
1.710	0.989	0.944	1.151	1.036	349	38.1	6.46
0.951	0.985	0.980	1.011	0.850	182	20.0	5.05
0.892	1.054	0.963	0.906	0.834	668	74.3	5.16
0.943	0.993	1.009	1.052	0.987	157	17.5	6.87
0.990	0.962	0.972	1.067	0.907	360	40.4	5.29
1.034	0.996	1.017	0.983	1.042	450	48.9	5.20
1.200	1.012	0.965	1.140	1.038	176	19.9	5.74
0.838	1.043	0.955	1.040	0.853	276	31.0	5.40
1.224	1.091	1.073	1.036	0.956	176	19.1	9.29
1.273	1.038	0.985	0.850	0.979	141	15.0	9.47
0.951	0.995	0.977	1.566	0.930	449	49.6	7.28
1.172	0.959	1.003	1.086	1.195	649	75.7	6.98
0.941	0.907	0.993	1.127	1.095	344	38.5	5.17
0.904	0.999	0.937	0.999	0.868	194	21.3	5.66
0.916	1.049	1.016	0.930	0.825	460	50.8	5.41
1.213	0.991	1.009	0.957	1.158	328	36.5	5.96
1.064	1.065	0.915	1.234	1.017	161	18.2	4.45
0.962	0.897	0.956	1.022	0.980	376	41.7	4.87
1.234	0.986	1.017	1.363	1.359	799	91.5	5.29
1.000	1.035	0.989	0.924	0.916	360	40.1	5.21
1.357	0.908	1.018	0.968	1.443	209	22.8	6.81
1.451	0.993	1.086	1.318	1.688	729	81.6	5.52
1.222	0.994	1.001	1.518	1.125	559	61.7	7.12
1.070	1.037	0.970	1.073	0.961	175	19.3	6.55
1.021	0.965	1.005	1.091	1.199	399	44.1	5.12
0.768	1.151	0.877	0.822	0.862	504	56.9	5.07
0.826	0.930	1.002	0.887	0.834	363	40.3	5.90
0.726	1.040	1.039	0.951	1.179	251	27.7	5.17
0.687	1.022	1.006	1.005	0.747	106	12.3	5.03
0.942	1.199	0.901	0.654	0.879	307	33.4	5.01
1.269	0.873	1.003	1.111	1.209	435	48.8	6.68
0.910	1.051	1.087	0.716	0.957	300	32.7	9.80
1.131	1.124	1.141	1.005	0.916	81	9.1	8.91
1.478	1.002	1.018	1.329	1.556	397	45.7	5.85
0.877	1.034	0.927	0.809	0.742	94	10.2	5.54
0.628	0.873	0.967	0.879	0.827	413	42.5	9.41
0.683	0.931	1.003	0.887	0.832	332	35.3	9.20
0.992	0.945	1.008	1.110	0.956	394	43.5	6.55
0.872	1.064	0.812	1.352	0.997	163	18.4	5.58
0.872	0.886	0.999	0.815	1.053	325	37.5	8.18
0.948	0.831	0.995	1.213	1.061	280	31.1	5.80
1.143	1.000	1.009	1.143	1.184	459	50.5	5.66
1.315	1.105	1.072	1.066	0.934	101	11.7	10.64
0.999	1.024	0.988	1.068	0.785	304	33.6	5.10
1.016	1.021	0.997	1.423	0.899	98	11.3	5.58
1.229	0.938	1.027	1.573	1.709	304	33.7	5.58
1.262	1.001	0.949	1.126	1.166	428	48.9	5.25
0.861	0.846	1.000	0.757	1.091	252	27.1	5.71
1.350	1.000	1.053	1.010	1.003	115	13.0	10.56
1.137	0.945	1.023	1.001	1.139	434	47.6	6.77
1.316	1.095	0.984	1.068	1.013	513	57.0	6.11
0.772	0.904	1.000	0.984	0.906	433	47.8	9.63
1.038	1.049	1.116	1.189	1.182	348	37.4	10.42

1.041	1.032	1.045	1.229	1.220	1005	114.3	6.52
1.009	1.440	1.582	0.451	1.073	341	36.8	10.04
1.567	1.105	0.888	1.591	1.055	101	11.7	9.70
1.377	0.950	1.000	1.267	1.358	424	47.0	6.61
1.423	1.092	0.903	0.905	1.074	83	9.5	4.84
0.888	0.967	0.971	1.147	0.776	182	20.4	5.52
1.105	1.035	0.886	0.647	0.717	72	8.6	4.82
1.195	1.000	0.983	1.651	1.074	437	49.1	5.82
1.106	1.074	0.997	1.096	1.276	181	20.3	5.30
1.108	1.018	0.945	1.096	0.986	304	32.9	5.10
1.572	0.930	1.014	1.132	1.482	826	91.8	8.47
1.279	0.917	0.996	1.153	1.127	224	25.2	10.20
1.031	0.958	0.989	1.115	1.109	340	38.3	6.96
0.700	0.721	1.038	1.303	0.792	169	18.6	5.97
1.340	0.972	0.998	1.118	1.044	129	13.7	11.44
0.898	1.120	1.044	0.514	0.826	320	35.4	7.56
1.030	0.993	0.999	1.169	0.819	330	36.4	6.61
2.030	1.003	0.983	1.284	1.847	503	56.1	9.54
0.779	0.949	0.938	0.944	0.839	228	25.4	5.17
1.190	0.965	1.021	1.029	1.090	302	34.8	5.72
1.096	0.901	1.109	1.123	1.558	459	50.9	6.07
0.962	1.009	1.023	1.331	0.857	192	22.1	6.54
1.282	0.976	0.967	1.207	0.945	84	9.7	9.79
1.178	1.011	0.976	1.288	0.997	280	32.0	5.26
0.977	1.056	0.983	1.109	1.014	158	17.3	4.60
0.886	1.007	0.955	0.442	0.968	320	35.4	7.14
1.048	1.001	0.836	1.023	1.012	158	18.1	4.83
1.047	0.937	0.999	0.744	1.142	356	40.2	7.11
1.177	0.912	0.837	2.117	1.062	158	18.1	4.89
0.853	1.175	1.053	0.380	0.939	420	47.1	5.63
0.987	1.359	0.973	0.658	0.977	161	18.2	4.49
1.223	0.990	1.019	1.054	1.109	874	96.2	6.32
1.175	1.007	1.055	1.154	1.538	342	38.0	5.44
1.370	1.001	0.998	1.103	1.007	361	40.3	6.01
1.361	0.972	0.998	0.927	1.007	122	13.2	9.92
1.078	1.045	0.958	1.215	1.067	413	46.7	4.92
0.685	0.783	0.774		0.805	335	38.3	4.94
0.783	1.099	0.896	0.504	0.927	357	40.3	7.11
0.828	1.089	0.972	1.156	0.967	285	31.4	5.64
1.495	1.083	1.062	1.761	0.996	101	11.7	9.94
1.275	1.143	1.214	0.559	1.353	209	22.8	6.38
1.225	1.026	1.003	1.192	1.033	144	17.2	4.68
1.348	0.979	1.059	1.799	1.555	540	60.9	5.03
1.024	1.073	1.034	1.181	0.954	184	21.1	3.98
1.068	0.942	0.899	0.670	0.900	595	63.1	5.44
1.004	0.934	0.949	0.720	0.899	405	45.8	6.81
1.018	1.024	1.001	1.041	1.008	558	60.6	6.46
1.411	0.980	0.967	1.142	1.220	401	43.6	6.13
1.085	0.805	0.944	2.145	1.908	315	35.7	7.31
0.939	1.013	0.961	1.147	0.834	331	37.1	5.12
1.033	0.932	0.988	1.216	1.026	476	54.7	5.86
0.947	1.386	1.208	0.560	1.113	704	80.5	9.33
1.092	1.077	0.983	1.072	1.043	242	26.6	4.89
1.096	0.903	0.873	1.546	1.272	315	35.7	7.71
1.105	0.913	1.082	1.034	1.595	148	17.1	5.25

1.211	0.874	0.990	1.073	1.435	329	35.1	5.72
1.134	1.037	1.013	0.955	1.344	450	50.6	8.87
1.410	1.047	0.986	0.809	0.980	117	12.8	9.91
1.419	1.047	0.976	1.198	1.078	318	36.4	6.62
0.826	1.027	0.989	1.184	0.824	200	21.7	7.53
1.014	0.944	0.977	0.975	1.125	366	40.3	4.96
0.894	0.957	0.990	0.863	0.931	352	38.7	5.49
1.209	0.973	0.990	1.210	1.065	523	59.2	5.67
1.100	1.025	1.040	1.215	0.946	93	10.4	9.88
1.017	0.987	0.996	1.279	1.112	279	31.8	8.73
1.057	1.049	0.954	0.955	0.958	304	35.0	5.27
1.122	0.998	1.023	1.093	1.191	748	84.5	6.11
0.986	0.984	1.091		0.790	307	33.4	5.06
1.012	0.980	0.941	0.813	0.855	179	19.8	5.21
0.515	0.936	1.012	0.709	0.980	407	45.1	9.67
0.948	1.024	0.985	1.228	0.784	437	50.2	6.29
1.456	0.965	0.990	0.703	1.280	141	15.4	8.40
0.960	0.940	0.837	2.440	1.017	403	44.4	6.74
1.126	1.053	0.972	1.213	1.071	275	30.8	4.97
1.969	0.915	1.090	1.043	1.491	140	15.2	8.40
1.180	0.999	1.026	1.130	1.057	768	89.5	7.47
0.926	0.985	0.930	0.984	0.842	141	15.4	4.50
1.070	1.027	0.975	0.924	0.891	362	41.5	5.11
1.231	0.970	1.054	1.046	1.009	476	53.3	9.36
0.958	1.030	0.992	1.115	1.037	380	43.2	5.92
1.148	1.050	0.986	1.053	1.264	333	36.2	5.50
1.053	0.935	0.949	0.927	0.978	309	34.2	9.70
1.091	0.963	1.003	0.689	1.224	212	23.7	5.30
1.298	0.934	0.983	1.708	1.201	466	51.2	5.43
0.960	1.029	0.964	1.170	0.889	255	28.3	5.69
1.261	1.033	1.057	0.942	0.936	103	11.4	9.19
0.824	0.997	0.978	1.469	1.040	541	61.1	9.39
0.982	0.876	0.990	2.941	0.832	405	45.8	6.87
1.011	1.025	1.051	1.012	1.071	447	46.9	5.20
0.931	1.011	0.993	1.412	0.875	214	24.3	5.02
0.623	0.941	0.993	0.826	0.818	309	32.6	6.33
0.949	1.012	1.024	0.960	0.925	329	35.5	5.38
0.881	0.893	0.943	0.924	0.889	336	36.3	6.81
0.990	0.993	0.978	0.928	0.789	419	46.8	5.15
1.358	0.998	0.893	0.941	1.135	102	11.5	9.63
0.939	0.831	1.082	0.581	0.742	457	48.7	9.60
0.955	1.031	1.017	1.117	0.857	322	35.9	6.62
0.896	0.877	1.033	0.996	1.178	319	34.9	8.00
0.843	0.917	0.969	0.901	0.870	721	79.1	5.76
0.949	0.987	1.074	0.950	1.019	308	32.8	4.92
1.124	1.523	0.865	1.017	0.976	614	65.2	5.52
0.720	0.872	1.176	1.033	0.852	163	18.4	5.39
1.174	0.914	1.143	1.441	1.297	212	23.7	5.31
1.129	1.123	1.163	0.823	1.420	450	50.6	8.75
0.844	0.934	0.991	1.082	0.983	238	27.4	6.07
1.121	1.086	1.099		0.876	304	32.9	5.11
1.284	1.002	0.936	0.952	1.018	146	16.2	10.62
1.030	1.025	0.999	1.076	0.906	156	17.3	5.01
1.245	1.074	1.010	0.793	0.987	76	8.8	10.73
1.433	1.038	1.052	1.438	1.421	779	88.6	6.47

1.456	0.971	0.898	0.956	0.974	127	14.4	10.42
1.033	1.028	1.014	0.956	1.009	483	55.7	6.34
0.903	1.045	0.950	0.929	0.845	194	21.5	5.08
0.996	1.014	0.966	0.942	0.816	80	9.3	4.23
0.948	1.054	1.021	0.848	0.876	271	30.3	5.68
1.144	1.000	0.997	1.407	1.044	218	24.4	6.79
0.896	1.081	1.071	1.394	1.344	149	16.9	4.73
1.140	1.076	1.079	1.113	0.954	119	13.0	9.86
1.306	1.025	1.018	1.155	1.219	661	73.8	6.86
0.972	1.072	1.022	1.134	1.144	231	25.1	4.78
1.211	0.946	1.029	1.003	1.268	865	96.3	5.88
1.207	0.887	1.058	1.306	1.511	785	87.9	8.95
0.968	1.045	1.007	1.109	0.889	252	28.9	9.23
0.998	1.047	1.028	0.840	1.076	190	21.0	5.68
1.115	0.994	1.107	4.052	1.047	388	43.3	5.12
1.167	0.993	1.014	1.344	1.146	465	53.9	8.40
0.777	0.965	0.987	1.711	0.970	299	33.6	6.38
0.841	1.055	0.973	0.670	0.820	214	24.4	5.02
1.229	1.006	1.037	1.006	0.931	131	14.4	10.39
1.233	0.966	0.953	0.944	1.348	540	60.7	5.03
1.076	0.999	1.048	1.001	1.111	431	46.1	5.41
0.869	1.153	0.957	1.133	1.036	415	47.2	4.59
0.804	1.059	0.918	0.723	0.761	80	9.1	4.12
0.908	2.074	0.994	1.854	0.562	367	41.2	9.72
1.120	1.015	1.020	1.119	1.257	431	49.0	5.29
1.126	1.009	1.048	1.001	1.126	376	41.6	6.07
0.944	1.005	0.981	1.102	0.777	470	53.4	5.66
1.245	1.003	0.988	1.128	1.167	185	21.0	5.00
1.199	0.923	0.981	0.824	1.033	135	15.0	10.80
1.012	0.939	0.959	0.766	0.964	279	30.3	6.54
0.564	1.043	0.964	1.243	0.698	93	10.8	6.37
0.914	0.936	0.911	0.833	0.905	181	21.7	5.43
1.268	1.015	1.045	0.905	1.467	243	25.9	7.49
0.796	1.028	0.979	1.246	0.838	384	42.5	5.35
0.933	0.993	1.047	1.186	0.766	230	25.1	5.87
1.072	0.844	0.956	1.318	1.018	279	30.3	6.43
1.084	0.929	0.971	0.949	0.985	350	39.2	5.99
0.770	0.977	0.974	0.916	0.803	167	18.7	5.08
1.188	0.923	0.993	1.070	1.082	698	78.8	6.68
1.083	0.961	1.026	0.554	1.275	421	47.2	8.43
1.003	0.881	1.023	1.096	1.075	113	12.9	9.28
1.128	1.352	0.930		1.175	415	47.2	4.60
0.947	1.128	1.024	1.193	0.934	73	8.7	5.45
0.902	0.972	0.976	1.272	0.837	276	30.3	5.24
1.063	0.864	0.966	0.957	1.126	654	72.9	7.74
0.941	0.979	1.004	0.865	1.086	212	23.8	5.19
1.234	0.942	0.995	1.380	1.108	585	65.5	8.63
0.827	1.066	0.990	0.943	0.741	311	34.4	5.15
2.085	1.091	0.957	1.856	1.430	330	36.7	8.95
1.125	1.011	1.023	1.054	0.902	349	39.7	5.85
1.355	1.008	1.028	1.127	1.433	453	52.0	9.80
1.514	1.135	1.012	1.268	0.881	65	7.6	9.82
1.001	0.902	0.947	2.842	1.114	388	43.3	5.16
0.948	1.252	1.203	0.426	1.065	138	15.3	6.57
1.466	0.973	1.112	0.804	1.451	305	33.4	5.99

0.887	0.872	0.844	1.084	1.047	421	47.1	7.15
0.869	1.038	1.132	1.441	0.736	242	26.6	4.98
1.267	0.892	0.820	0.656	1.343	309	34.3	9.67
0.916	0.931	0.997	1.590	1.039	464	52.1	9.55
1.065	0.997	1.051	0.968	0.876	88	10.6	10.29
1.134	1.028	0.977	0.986	1.089	299	34.6	5.35
0.907	0.887	0.991	1.217	1.134	333	37.4	6.28
0.604	0.902	0.989	0.660	0.901	286	32.0	10.01
1.048	1.014	1.050	1.454	1.403	206	22.4	6.24
1.149	0.951	1.053	0.864	1.094	284	31.9	7.59
1.030	0.993	1.004	1.117	1.056	887	100.7	6.01
0.971	0.968	0.973	1.383	0.930	189	21.7	5.03
1.312	1.016	1.005	1.375	1.372	267	30.5	5.35
0.765	0.961	1.007	1.033	0.800	189	21.3	6.00
1.127	0.963	1.019	1.016	1.383	325	37.0	6.06
0.788	0.930	1.030	1.066	0.990	137	15.7	6.55
1.405	0.912	1.025	0.959	1.913	327	35.3	5.06
1.577	0.986	1.007	1.197	1.912	492	55.8	5.48
1.095	0.980	0.990	1.096	0.999	624	68.4	9.35
1.026	1.003	1.082	1.005	1.294	298	32.5	8.41
0.761	1.070	1.055	0.992	0.691	184	21.3	6.84
0.779	1.081	0.988	1.158	1.013	140	16.0	5.87
1.029	1.053	0.969	1.003	0.834	701	80.1	6.54
1.092	0.970	1.051	1.017	0.957	151	16.9	9.67
0.998	0.957	0.983	0.868	1.002	204	23.0	6.24
1.038	1.010	0.924	0.717	0.933	479	53.3	6.49
1.505	1.024	0.989	1.053	1.120	96	10.2	10.33
1.081	1.023	0.997	1.077	1.097	73	8.3	8.48
1.097	1.051	1.017	2.039	1.170	255	29.1	4.46
0.859	0.708	0.958	1.108	1.138	242	27.4	9.76
1.108	1.082	1.019	0.924	0.880	38	4.5	5.95
1.043	1.062	0.975	1.187	1.025	151	17.0	4.07
1.070	0.921	1.026	1.128	1.213	612	68.8	5.16
0.971	1.010	1.036	1.127	1.358	145	16.6	6.68
0.943	1.013	1.039	1.065	1.020	275	31.3	6.89
0.978	1.019	1.017	0.995	0.932	374	42.5	8.57
1.030	1.028	1.062	1.021	1.118	551	62.5	5.06
1.104	0.939	1.055	1.068	1.266	1438	162.1	5.88
1.107	1.029	1.052	1.217	0.920	74	8.0	6.28
1.161	0.991	1.006	1.091	0.965	159	17.9	9.83
0.715	1.054	1.154	1.091	1.013	325	35.3	9.95
0.863	0.965	0.991	1.149	0.961	186	21.2	7.12
1.116	1.031	1.025	1.347	1.038	460	52.3	9.35
0.980	0.949	0.949	0.978	0.920	120	12.7	8.90
1.149	1.051	1.048	0.920	0.926	116	13.2	10.35
1.267	0.979	1.057	1.302	1.480	365	39.5	8.44
1.062	0.999	0.991	0.993	1.123	564	65.7	9.20
1.686	1.067	0.886	0.920	1.393	61	7.0	10.78
0.939	1.025	0.956	1.012	0.855	216	24.9	5.45
0.627	0.853	0.925	0.833	0.980	365	40.6	9.63
0.955	0.981	0.996	1.184	0.901	318	37.9	5.33
0.900	0.858	1.008	1.200	1.100	264	29.3	8.50
1.203	0.986	1.010	1.269	0.983	184	21.3	4.86
0.758	1.043	0.994	1.379	0.749	384	42.5	5.41
1.115	0.912	1.106	1.058	1.189	296	32.1	6.18

1.065	0.999	1.038	1.303	1.410	224	24.7	6.81
0.964	1.043	0.954	0.856	0.865	351	39.9	5.41
0.929	1.009	1.083	0.975	0.842	59	6.7	7.05
0.637	1.014	1.053	0.894	0.741	454	50.1	9.94
1.183	0.968	1.065	1.245	1.366	349	37.8	5.76
1.244	1.054	1.075	1.077	1.490	409	43.0	5.31
1.205	0.991	0.984	1.024	1.201	461	50.4	5.33
0.774	0.879	0.896	1.653	0.902	464	52.1	9.58
0.941	0.958	1.055	1.206	1.011	197	22.8	8.07
0.897	0.928	0.983	1.207	1.080	291	32.4	8.15
1.119	0.869	1.009	0.975	1.133	369	41.2	7.01
1.088	1.040	1.002	1.020	0.887	189	21.0	5.63
1.057	1.030	1.023	1.095	1.046	186	21.8	5.66
0.833	0.899	0.911	0.601	0.875	141	15.4	5.20
0.961	1.309	1.367	0.841	1.130	189	20.9	5.60
0.964	0.975	0.979	0.874	0.985	174	18.7	5.24
0.947	0.990	0.994	0.879	0.863	387	42.7	5.17
0.915	1.096	1.060	0.792	0.776	79	9.2	5.01
1.149	1.013	1.038	1.173	1.000	130	14.7	5.17
1.083	1.008	1.002	1.229	0.904	337	38.9	5.88
1.079	0.983	0.974	1.158	1.003	244	27.2	5.67
0.832	1.000	1.071	1.478	0.809	172	18.7	5.16
1.311	0.939	1.007	1.615	1.549	386	42.6	6.02
0.485	0.745	1.216	1.288	0.318	504	57.2	5.64
0.746	1.036	0.940	1.142	0.887	238	27.4	5.38
0.834	0.942	1.042	1.288	1.106	276	29.5	10.20
1.057	0.972	1.023	1.195	1.023	332	37.9	5.34
0.864	1.000	0.992	0.743	0.895	186	21.2	7.47
1.056	0.990	1.044	1.349	1.067	89	10.0	4.88
1.031	0.982	1.051	0.853	1.000	147	16.7	9.29
1.127	0.913	1.028	1.010	1.160	246	27.7	8.63
1.082	1.004	1.051	1.219	1.208	219	24.6	6.74
1.323	1.109	0.935	1.058	1.342	256	28.5	5.66
0.892	0.917	0.856	1.940	0.988	272	29.7	9.67
1.320	1.020	0.974	1.201	1.428	401	42.1	5.24
1.216	0.905	1.024	1.139	1.358	335	37.8	8.81
1.126	0.889	0.978	0.913	1.117	301	34.0	8.15
0.966	0.846	0.975	0.497	0.978	272	29.8	9.76
1.275	1.031	1.009	0.990	1.102	111	12.2	4.82
1.164	1.131	0.983	1.028	0.944	61	6.6	10.15
1.140	0.955	1.059	0.963	1.226	437	48.0	7.97
1.148	0.932	1.042	0.999	1.107	199	22.5	7.83
0.705	0.885	0.978	1.196	1.041	394	43.1	5.57
0.955	0.956	1.004	0.978	0.837	1063	119.4	6.16
0.980	0.984	0.995	1.386	0.865	265	29.8	5.22
0.936	1.029	1.043	0.235	0.934	374	42.7	9.04
1.067	0.919	1.039	1.055	1.259	445	49.8	9.20
1.067	1.000	1.048	1.138	0.888	217	24.9	6.28
0.933	0.896	0.964	1.127	0.963	333	35.9	5.49
0.702	0.991	1.005	0.936	0.864	553	64.4	5.80
1.636	1.014	1.108	1.763	1.402	238	27.1	9.45
1.234	0.986	1.028	1.037	1.128	244	28.6	7.21
1.322	1.009	1.048	1.215	1.080	89	10.4	9.48
1.032	0.948	1.023	1.266	1.142	447	51.1	8.81
1.345	0.978	0.969	0.983	1.083	162	17.2	7.56

0.920	1.004	1.072	1.172	0.935	418	47.6	6.58
1.001	1.043	0.961	0.767	0.966	207	23.8	4.74
0.949	0.960	1.030	1.056	1.071	175	20.7	8.06
1.093	0.960	1.020	1.028	1.189	185	21.0	8.27
1.156	0.977	0.934	0.920	1.236	595	66.7	6.70
1.074	0.937	1.098	2.031	0.924	475	52.8	7.39
1.589	1.001	1.075	1.276	1.632	421	47.5	6.77
1.055	1.024	0.946	0.991	0.796	628	73.5	5.39
1.147	0.951	1.092	1.014	1.262	386	41.4	5.52
1.175	1.046	1.042	1.993	1.412	149	17.0	5.16
1.055	1.016	1.019	1.052	1.114	438	48.2	6.35
1.150	0.906	1.075	0.948	1.456	749	85.8	6.99
1.118	0.987	1.031	0.933	1.170	1189	134.3	6.58
0.975	0.970	0.942	1.123	0.995	225	25.6	5.20
1.024	1.083	0.964	1.222	0.875	298	34.6	5.27
1.050	0.963	1.052	1.205	1.168	1035	116.5	8.85
1.090	1.069	0.993	0.811	0.895	102	11.6	4.53
1.294	0.988	0.995	1.166	1.534	255	29.2	5.24
0.770	0.942	0.951	0.758	1.170	115	13.5	9.63
1.019	1.054	0.941	0.716	0.974	118	13.3	8.32
1.175	1.781	1.984	0.218	1.476	136	15.3	10.51
0.835	0.932	0.993	0.990	1.097	234	26.2	5.11
0.922	0.935	0.977	0.870	1.083	430	48.4	4.39
1.490	0.917	1.050	1.049	1.229	196	22.4	9.31
0.999	0.981	1.007	0.891	0.951	927	106.0	6.52
0.930	1.019	0.924	1.135	0.776	315	35.4	4.96
0.898	1.085	1.004	1.408	1.024	110	12.6	5.11
0.681	1.001	0.966	1.077	0.777	93	11.0	6.14
0.930	1.130	1.014	1.268	0.892	113	12.7	8.31
1.127	0.957	1.038	1.029	1.329	385	42.2	5.94
0.854	1.035	0.997	1.389	0.759	185	20.7	5.81
0.907	0.967	0.982	0.740	0.934	1122	119.6	9.23
0.834	0.990	0.968	0.904	0.822	252	29.1	6.02
1.176	0.928	1.088	0.958	1.193	348	37.4	10.37
1.278	1.142	1.163	0.898	0.949	66	7.8	12.66
0.773	0.907	1.045	0.575	0.805	283	30.0	9.76
1.925	0.987	1.014	1.429	1.843	278	31.3	5.43
1.456	1.050	1.029	1.376	1.424	172	20.1	6.80
0.850	1.023	1.051	0.895	0.954	406	43.7	9.51
0.995	1.027	0.971	1.193	0.892	259	28.9	5.54
0.996	1.022	0.989	0.995	1.000	220	24.7	4.89
0.855	0.980	1.028	0.898	1.204	185	20.3	5.41
1.039	0.878	1.066	1.227	1.360	296	32.0	6.18
1.373	0.999	1.063	1.241	1.294	818	92.6	8.81
0.940	1.127	1.003	0.975	0.968	265	30.3	4.48
0.856	0.890	0.960	0.604	0.890	873	94.6	6.52
1.324	1.026	1.026	1.344	1.272	356	40.8	5.77
0.906	0.994	0.968	0.794	0.988	304	33.1	9.61
0.657	0.974	0.987	0.825	0.934	246	28.4	5.86
0.861	0.992	1.002	0.929	0.986	220	24.7	6.24
1.119	1.037	0.978	1.414	0.968	244	28.4	6.18
1.073	0.961	1.039	0.882	1.237	174	20.2	4.64
1.001	0.833	0.992	1.115	1.152	368	40.6	9.48
1.009	0.930	0.871	1.019	0.932	334	36.3	5.12
1.087	1.045	0.963	1.251	1.028	149	17.1	4.65

0.872	0.961	1.009	1.194	1.082	236	26.6	9.10
0.937	1.019	1.063	0.904	1.154	135	15.8	8.84
0.925	0.950	1.009	0.484	0.974	133	15.4	9.95
1.399	0.962	1.042	1.336	1.503	648	72.6	8.19
1.020	1.028	1.002	1.180	0.876	306	33.3	5.44
0.601	0.985	1.030	1.177	0.865	151	16.6	4.61
0.849	0.845	1.035	0.755	0.868	283	30.0	9.83
1.018	1.087	0.960	1.168	0.904	214	24.6	8.09
0.712	0.861	1.016	1.148	1.040	215	24.0	9.16
1.091	1.089	0.945	0.859	0.826	97	10.5	5.07
0.934	0.988	1.006	1.103	1.027	492	56.7	5.31
0.878	0.938	0.999	0.872	1.027	204	23.8	6.68
1.179	1.046	0.952	0.907	0.972	115	13.1	4.79
1.438	0.915	1.113	1.386	1.504	375	42.1	6.10
0.975	1.046	0.957	0.887	1.057	297	33.5	8.66
1.435	1.083	0.981	1.042	1.166	250	27.6	8.95
0.742	0.919	1.082	0.673	0.812	314	33.8	9.01
1.027	1.141	0.896	0.539	0.886	261	28.9	5.17
1.066	1.067	0.997	0.967	1.140	228	25.7	5.38
1.061	1.072	0.940	1.033	0.963	199	22.6	5.62
1.036	1.007	1.044	1.135	1.133	155	17.9	8.69
0.915	0.946	0.954	1.164	0.845	289	31.9	5.10
0.987	0.949	0.965	0.926	1.286	347	38.1	5.38
1.033	0.897	1.002	0.929	1.033	209	23.6	7.31
0.946	0.964	0.932	0.859	1.117	413	46.1	9.26
1.020	0.998	0.995	0.949	1.112	321	36.6	6.93
0.922	1.003	1.017	0.586	0.986	288	32.0	9.99
1.083	1.014	1.051	1.246	1.377	799	90.8	9.07
1.524	1.080	1.029	0.922	1.237	246	28.6	4.98
1.160	0.975	1.035	1.285	1.339	384	42.0	7.02
1.031	0.968	0.983	1.175	0.951	383	43.5	8.19
1.362	0.877	0.784	0.909	1.681	386	42.5	5.90
0.833	0.988	1.082	0.765	0.869	457	51.2	5.76
0.883	0.901	0.947	1.145	0.926	333	37.7	5.39
0.864	0.993	0.954	1.133	0.847	148	16.5	4.03
1.017	1.028	1.022	1.223	1.167	266	29.5	6.42
0.860	1.051	1.006	1.304	0.866	79	8.9	3.99
0.956	0.986	0.992	0.815	0.988	129	14.7	4.53
0.729	0.918	0.957	0.994	0.854	996	114.4	9.69
0.654	1.165	1.066	2.320	0.935	74	8.4	6.02
1.010	0.975	1.033	1.211	1.284	275	31.1	5.55
1.030	0.892	1.036	1.181	1.212	637	72.6	5.77
1.591	0.973	1.094	1.007	1.738	218	25.3	5.68
1.297	1.012	0.993	1.336	1.160	196	21.6	5.19
0.957	0.903	0.956	0.716	1.085	665	70.8	6.65
0.665	0.907	1.036	1.236	0.805	265	29.8	5.73
1.010	0.955	0.940	1.370	1.205	233	26.8	9.06
1.002	0.970	1.049	0.940	1.018	240	27.0	8.92
1.159	1.008	0.946	1.153	0.835	186	21.2	8.66
0.818	1.059	1.029	0.909	0.942	239	26.5	6.15
1.364	0.976	1.022	1.441	1.218	273	30.2	9.11
1.093	0.956	0.991	1.100	0.961	302	33.0	6.73
0.960	1.064	1.005	1.155	0.953	80	8.9	4.60
0.810	0.959	1.018	1.255	0.993	133	15.3	5.08
0.981	1.015	0.982	0.807	1.266	165	19.1	5.66

1.025	0.999	1.138	0.900	1.279	438	48.3	6.02
1.118	1.057	0.994	0.944	1.067	273	31.3	4.65
0.900	0.975	0.994	1.046	1.109	331	36.8	8.95
1.049	0.958	0.965	0.986	1.055	365	41.0	5.08
1.059	1.038	0.979	0.901	0.893	318	35.9	6.05
0.887	0.883	0.981	0.979	0.949	272	31.1	6.14
0.888	0.924	1.022	0.856	1.194	293	32.2	5.16
0.946	1.006	0.975	0.570	0.944	353	40.3	9.70
0.852	0.959	1.025	0.675	0.912	428	49.7	9.83
0.993	0.978	0.999	1.164	1.039	128	14.3	5.20
1.082	0.939	1.085	0.916	1.296	259	28.4	5.94
0.941	0.953	1.003	0.819	0.916	433	47.9	9.69
0.989	0.942	1.078	0.859	1.091	251	27.5	7.49
0.985	1.030	0.998	0.793	0.872	214	22.7	4.93
0.926	1.055	1.014	1.468	0.942	367	42.3	4.65
0.924	1.013	0.957	0.947	0.811	162	18.1	4.86
0.873	1.001	1.061	1.009	1.074	165	18.5	10.48
1.146	0.928	0.983	1.043	0.916	283	31.5	9.31
1.026	1.006	0.944	1.186	1.005	161	17.7	5.07
0.822	0.986	1.117	0.690	1.240	303	32.7	5.72
0.892	0.946	1.015	0.841	0.969	431	46.8	9.77
1.168	1.003	1.054	1.224	1.008	91	10.4	9.11
0.849	0.944	0.994	0.800	0.982	569	65.7	5.43
1.214	0.948	1.118	0.919	1.351	460	51.8	6.60
0.809	0.869	0.954	0.591	0.845	306	33.8	9.73
1.022	0.981	1.005	1.050	0.889	263	30.5	5.31
1.020	0.931	0.957	1.158	1.259	194	21.8	6.11
1.661	0.971	1.116	1.000	1.131	38	4.4	10.62
1.032	0.856	0.986	0.973	1.201	252	28.0	9.52
2.061	1.066	1.010	1.602	1.718	177	19.9	9.26
0.959	1.019	1.069	0.892	0.916	366	42.6	6.55
1.140	0.912	1.136	1.279	1.476	270	30.1	6.29
1.103	1.102	0.912	1.286	0.871	183	21.0	6.95
1.040	0.970	1.000	1.075	1.176	428	48.4	5.59
0.806	0.970	0.998	0.831	1.068	141	15.4	9.57
0.816	0.962	0.984	0.716	0.888	272	29.8	9.86
0.920	1.027	0.906	1.061	1.041	375	41.1	9.19
0.943	0.911	1.054	0.658	1.024	892	98.9	7.15
0.754	0.921	1.010	0.754	0.901	589	66.0	9.50
0.898	0.968	1.113	1.404	0.950	181	21.3	9.64
1.029	1.076	0.979	1.285	0.774	375	41.1	9.19
1.025	0.916	1.010	0.950	0.889	294	33.0	8.44
0.866	0.970	1.047	0.721	0.823	301	33.8	9.44
1.104	0.896	1.162	0.974	1.064	251	27.6	5.16
0.912	1.065	0.980	1.052	0.919	208	23.1	6.81
0.690	0.930	0.934	0.830	0.883	458	49.0	9.74
1.029	0.974	0.936	0.986	0.972	238	27.3	5.16
0.863	0.902	0.969	1.171	1.007	215	24.2	6.81
0.946	0.915	0.980	0.984	1.066	560	63.1	4.89
1.081	0.962	0.991	0.969	1.072	450	50.3	8.69
0.958	1.030	1.022	0.825	0.895	450	50.3	8.69
0.764	1.017	1.029	0.989	0.899	85	9.9	6.29
0.995	0.926	0.985	0.794	1.057	543	60.8	6.42
1.195	0.987	1.098	1.563	1.445	442	49.8	8.94
0.777	1.062	0.991	1.370	0.780	132	14.5	5.15

1.123	1.048	1.086	0.887	0.976	320	37.3	7.25
1.020	0.944	0.923	1.404	0.958	225	25.9	6.38
0.884	1.027	1.025	0.717	0.860	384	40.5	5.99
1.113	1.003	0.959	1.805	1.149	106	12.5	5.00
0.977	1.081	0.962	1.068	0.888	228	26.1	5.06
0.907	1.023	1.091	1.232	0.945	314	35.5	10.46
0.916	0.905	1.062	0.914	1.095	183	21.8	9.06
1.048	0.967	1.027	0.848	0.989	210	24.6	6.58
1.481	1.108	0.964	1.020	1.351	313	36.1	9.32
1.104	0.881	1.077	1.450	1.309	361	40.5	6.73
1.255	0.980	0.992	1.238	0.997	121	13.9	9.13
0.814	0.775	0.926	1.343	0.673	116	13.1	5.82
0.827	0.989	1.053	1.056	0.959	273	29.7	9.83
1.066	0.837	1.021	0.913	1.418	291	33.4	8.87
0.954	1.052	1.012	1.004	0.862	199	23.1	8.28
1.130	1.030	1.021	1.219	1.008	122	14.3	9.52
1.029	1.042	0.990	0.886	0.860	126	14.4	4.41
1.209	1.014	0.969	1.055	1.062	188	20.9	9.55
0.765	0.971	1.055	0.647	0.890	401	44.9	9.80
0.914	0.988	0.998	0.758	1.080	199	23.7	5.60
0.990	0.961	0.946	1.316	0.946	216	23.8	5.17
1.049	1.038	0.890	0.968	1.102	130	15.2	4.51
1.348	1.020	1.038	1.411	1.444	317	36.4	5.59
1.005	1.008	0.937	0.675	0.925	996	114.4	9.69
0.999	0.964	1.067	1.043	1.110	305	35.4	8.50
0.931	0.950	1.080	1.224	1.185	235	27.8	5.96
0.839	0.972	0.968	1.206	1.025	504	57.2	9.42
0.928	1.001	1.030	0.883	0.896	202	23.7	9.32
0.784	0.942	0.998	0.706	0.862	266	29.5	5.57
0.996	0.910	0.988	1.000	1.188	347	40.0	6.87
0.872	1.074	0.975	1.924	0.938	80	8.9	4.61
0.979	0.991	1.043	1.188	0.937	394	43.3	7.87
1.111	0.994	1.012	0.999	0.951	152	17.7	10.14
0.923	0.980	1.014	0.832	1.213	165	18.9	5.38
1.193	1.080	0.990	1.340	1.026	75	8.5	8.43
1.287	0.996	1.066	1.257	1.478	455	52.3	6.83
1.248	1.077	0.995	0.734	1.195	649	73.5	5.49
0.727	0.817	0.953	0.560	0.812	237	26.7	6.55
1.264	1.028	0.894	1.734	1.001	158	18.3	5.22
1.115	0.928	0.999	1.260	1.019	412	46.6	6.98
0.822	0.962	1.011	0.813	0.929	314	35.5	9.48
0.944	0.981	1.004	0.986	1.040	1204	139.3	5.59
0.792	0.933	1.002	0.765	0.915	372	40.5	9.44
1.456	1.001	1.046	1.081	0.975	85	9.4	10.62
0.958	1.288	0.824		0.823	115	13.2	4.79
1.238	0.984	1.100	1.394	1.473	513	58.0	6.33
0.948	1.006	0.971	0.891	0.936	406	46.2	5.06
0.942	1.203	1.033	1.005	0.944	250	27.8	8.79
0.921	1.081	1.009	1.276	1.024	92	10.8	4.94
0.856	0.971	0.918	0.898	0.901	247	27.5	6.81
0.917	0.984	0.988	1.046	0.877	338	37.6	5.71
1.251	1.033	0.989	0.776	1.023	309	34.7	5.06
1.375	1.069	1.039	1.228	1.328	297	34.1	4.96
0.862	1.007	1.008	0.636	1.015	241	27.5	5.97
0.960	1.010	1.006	1.264	1.011	454	52.7	8.51

0.830	0.997	1.005	1.069	0.896	186	20.9	4.70
0.967	0.981	0.995	1.143	1.024	146	16.7	9.69
1.208	0.985	1.021	1.235	1.429	308	33.6	8.51
0.676	0.833	0.951	0.885	0.949	319	35.6	9.28
1.164	1.138	0.943	0.565	0.959	249	28.5	5.73
0.877	1.214	1.073	1.183	0.946	279	29.5	6.40
0.829	0.880	1.180	0.643	0.987	279	29.6	6.40
0.979	1.064	0.996	0.946	1.125	174	20.1	5.74
0.920	0.822	0.953	0.965	0.974	260	28.5	9.03
0.945	1.090	1.028	0.641	0.850	159	18.2	6.70
1.046	0.981	0.983	0.693	0.990	514	56.4	8.82
1.133	1.104	0.951	1.990	1.413	155	18.0	5.10
0.795	1.012	0.982	0.827	0.847	155	17.6	7.43
1.173	0.875	1.067	0.956	1.345	302	34.4	6.96
1.264	0.968	1.043	0.924	1.444	266	31.2	8.98
0.885	0.964	1.019	0.724	0.815	135	15.3	7.49
0.900	0.939	1.038	0.976	0.960	137	15.3	9.33
0.906	0.978	1.023	0.701	0.926	721	79.7	9.32
0.686	0.968	1.050	0.875	0.885	373	41.2	9.52
1.226	1.000	0.994	1.160	1.295	264	29.5	8.91
0.871	0.991	1.004	0.818	1.040	234	26.6	6.79
0.739	0.852	1.052	0.639	0.715	72	7.9	9.70
1.238	0.897	0.901	2.954	2.326	136	15.6	5.39
1.039	1.072	1.048	1.085	0.903	267	30.6	6.23
0.953	0.963	1.084	0.915	1.028	351	38.6	5.21
0.954	0.964	0.987	0.863	1.128	309	35.1	5.22
0.837	0.979	1.103	0.754	1.061	259	28.3	5.63
0.996	1.050	0.949	0.867	1.020	171	19.8	4.72
0.836	0.922	0.897	0.967	0.847	75	8.4	7.12
0.988	1.048	0.956	0.753	0.954	425	47.5	5.60
3.268	1.070	0.912	2.513	2.363	66	7.2	5.95
1.229	1.302	1.390	0.631	1.256	410	46.5	9.74
1.021	1.045	0.994	1.005	0.930	411	45.7	9.63
1.405	1.032	1.273	1.198	1.599	232	26.2	8.51
0.969	1.081	1.033	0.651	1.011	204	22.5	5.80
1.077	0.970	1.051	0.901	1.077	783	86.7	5.57
1.280	1.054	0.962	0.893	1.277	349	38.9	5.20
1.166	1.040	0.992	0.867	1.002	279	31.6	9.32
0.898	0.972	1.003	0.815	0.982	190	21.4	8.25
1.031	1.031	0.900	0.555	1.183	210	24.1	5.14
0.979	1.042	1.000	0.667	1.168	681	74.0	9.44
0.888	1.048	1.048	0.849	0.974	246	28.0	7.03
0.854	1.111	1.027	1.019	0.810	189	21.5	5.01
0.794	0.986	0.973	0.598	0.907	319	35.4	9.51
1.055	0.994	0.913	0.821	1.085	155	18.0	9.44
1.165	1.061	1.017	0.985	1.080	285	32.3	6.16
1.087	0.991	1.019	1.281	1.150	946	105.1	8.31
0.892	0.974	1.034	1.058	1.034	397	43.3	9.01
0.997	0.987	0.980	0.654	0.967	235	26.8	5.35
1.080	0.960	1.043	1.718	1.428	404	46.5	9.29
0.972	0.975	1.039	1.155	1.347	226	25.8	5.67
1.201	1.069	0.994	1.142	1.063	209	23.2	7.49
1.330	1.037	0.948	1.078	1.232	320	35.8	7.42
0.881	0.997	0.849	0.738	0.841	213	23.9	6.20
0.927	0.973	1.024	0.753	0.830	351	38.6	4.82

0.701	1.062	1.034	0.848	1.164	119	13.4	9.99
1.069	0.977	1.004	0.434	0.942	409	43.9	9.44
1.014	0.943	1.026	1.096	1.114	227	25.9	5.76
0.705	0.906	0.973	0.806	0.970	644	68.5	6.40
1.486	0.906	1.176	1.582	1.580	458	50.3	6.64
1.467	1.129	1.270	1.616	1.558	164	18.7	8.10
1.118	0.949	0.940	0.715	0.911	190	21.4	8.34
1.328	1.061	0.975	1.190	1.294	455	51.5	9.42
0.907	0.914	0.983	0.735	0.982	171	19.5	7.65
0.924	0.865	0.980	1.373	0.987	452	48.6	6.24
1.090	0.859	1.112	1.737	1.268	99	10.9	9.76
1.001	0.916	1.101	0.746	1.078	384	42.5	5.57
1.099	1.151	0.942	0.658	0.739	75	8.8	4.74
1.138	1.052	1.080	1.090	1.064	82	9.8	9.50
0.981	1.051	1.049	1.188	0.938	314	35.5	10.42
1.130	1.220	0.951	0.690	1.146	287	32.1	5.68
0.861	0.978	0.990	0.831	1.050	320	36.0	9.41
1.019	0.998	1.033	1.053	1.103	1007	116.8	6.39
0.710	1.070	0.966	1.368	0.865	384	45.4	8.90
1.118	1.069	1.066	0.817	1.131	222	25.2	5.29
1.100	0.992	1.049	0.965	1.021	277	31.9	5.95
1.007	1.172	0.977	0.473	1.058	288	31.8	9.14
0.896	0.890	0.998	0.999	1.042	321	36.7	8.50
1.078	1.004	1.059	1.547	1.143	309	34.7	5.85
0.880	1.001	1.104	1.142	0.975	188	20.5	9.69
0.842	1.098	0.987	0.660	0.830	331	35.9	9.48
1.228	1.004	0.970	1.164	1.061	301	34.9	9.26
1.406	1.051	1.021	0.851	1.202	80	9.2	9.88
0.830	0.965	1.113	0.649	1.022	382	43.4	9.23
0.975	0.949	1.034	0.907	1.038	463	52.7	6.81
1.147	1.036	0.973	1.132	1.494	298	33.9	9.55
0.910	0.990	1.011	0.861	1.035	251	27.9	5.95
1.056	0.984	1.002	0.993	1.120	283	31.2	6.39
1.033	1.000	0.945	0.798	1.004	284	32.0	10.13
0.984	1.137	0.860	1.078	0.978	366	40.9	8.29
0.487	0.938	1.200	0.935	0.943	229	24.3	9.32
0.876	0.983	0.839	1.146	0.989	265	27.3	4.82
1.163	0.975	1.018	0.904	1.309	294	33.3	9.32
1.223	1.018	1.095	0.883	1.356	682	78.1	6.60
1.008	0.944	0.994	1.341	1.026	264	29.5	5.57
0.826	0.909	0.924	1.262	1.028	336	37.8	9.35
0.972	0.976	0.956	1.239	1.055	402	45.9	5.12
1.075	0.987	1.017	1.157	0.986	403	43.9	9.17
0.908	0.871	1.204	1.204	0.756	515	56.7	7.83
0.866	0.977	1.010	0.719	0.898	202	23.3	10.46
0.930	0.995	0.985	0.727	0.900	435	45.9	9.28
0.800	1.012	0.937	0.660	0.856	431	47.6	9.67
1.145	0.998	1.001	0.835	1.085	182	20.3	9.03
0.747	0.979	0.948	0.841	0.717	352	38.0	9.69
1.157	1.035	1.037	1.323	1.045	256	29.4	9.20
0.740	0.947	1.002	0.626	0.864	286	31.8	9.91
1.009	1.067	1.175	0.783	1.133	366	40.9	8.72
1.497	1.047	1.000	0.999	1.545	215	24.7	5.24
1.116	0.955	1.107	1.218	1.293	190	21.0	4.98
1.221	1.001	1.018	0.945	1.203	1165	132.0	5.66

1.005	1.036	1.204	0.726	1.043	471	55.1	7.08
1.069	0.954	1.019	0.768	1.232	245	28.1	4.87
1.614	0.905	1.065	1.192	1.523	189	21.5	5.19
0.855	1.021	1.111	0.609	0.981	224	24.8	9.94
1.044	1.064	1.007	0.717	1.042	464	51.2	4.81
0.838	0.942	1.000	0.704	0.892	204	23.4	9.96
1.619	0.944	0.906	0.831	1.057	77	8.3	10.15
1.007	1.320	0.736	0.789	1.228	298	33.3	6.38
1.390	1.116	1.087	1.434	1.399	142	16.0	9.42
0.944	0.961	1.015	0.758	1.052	224	25.4	9.13
0.828	0.902	0.972	0.917	1.002	123	13.3	4.35
0.914	1.093	1.171	0.748	1.031	360	40.7	10.23
0.814	0.952	1.015	1.344	0.888	172	19.6	8.31
0.919	1.016	0.963	1.302	0.899	519	58.6	6.87
0.836	0.880	0.964	0.708	0.979	292	32.3	7.88
1.114	0.889	1.041	1.217	0.911	123	14.0	8.88
0.816	1.083	1.076	0.898	0.942	605	66.7	9.82
1.150	1.049	1.082	1.110	1.050	150	16.9	5.77
1.182	1.013	1.076	1.255	1.434	303	34.3	8.63
0.969	1.168	0.795	1.183	1.117	32	3.5	9.54
1.475	1.026	0.877	0.648	1.503	58	7.1	10.73
2.357	0.951	1.027	1.496	2.107	237	27.5	8.88
0.706	0.958	1.087	1.075	1.135	183	19.5	9.76
0.654	1.834	1.224	0.249	1.092	106	11.7	9.13
0.780	0.932	0.977	0.756	0.860	320	35.6	9.89
1.064	0.954	1.003	0.903	0.981	594	67.3	8.54
0.793	1.007	0.994	0.856	0.931	217	25.7	8.41
0.840	0.961	0.962	0.678	1.052	242	26.7	5.10
1.091	0.939	0.961	0.863	1.091	122	14.5	10.11
0.825	0.955	0.991	0.949	0.951	90	10.7	9.70
0.691	1.077	0.970	0.661	0.834	228	24.8	6.32
0.837	0.988	1.027	1.500	1.201	78	9.3	5.01
0.895	1.167	1.028	0.771	0.874	294	33.9	5.52
0.899	0.968	0.960	1.076	1.138	1160	134.0	5.19
0.949	0.821	1.090	0.637	1.013	251	28.1	5.53
2.328	1.001	0.994	1.226	2.073	739	84.5	7.05
0.892	0.874	1.047	0.811	1.106	181	20.9	9.26
1.081	0.977	1.037	0.843	0.915	140	16.0	5.92
1.204	1.019	1.057	1.555	1.038	290	33.1	4.84
1.058	1.055	1.119	1.121	1.199	908	104.2	8.16
0.949	0.952	1.042	0.811	1.054	276	31.3	9.17
0.847	1.056	0.971	1.361	1.042	537	59.6	9.82
1.050	0.957	1.001	1.033	1.035	343	37.7	7.11
0.893	0.925	1.018	0.967	0.746	145	16.1	5.64
0.878	1.142	0.975	0.925	0.952	279	30.9	4.61
1.389	1.018	1.068	0.924	1.368	360	39.8	8.10
0.883	1.068	1.061	0.900	0.877	188	19.9	5.26
0.871	0.995	0.988	1.339	0.826	363	41.2	8.78
1.141	0.915	1.017	1.371	1.086	270	30.7	7.40
0.930	1.044	1.167	2.495	0.770	144	16.0	5.80
0.561	0.796	1.001	1.958	1.126	90	10.3	5.10
0.667	1.032	0.977	0.943	0.877	138	16.1	5.27
0.800	0.986	0.968	1.031	1.024	111	12.6	6.32
1.007	0.994	0.969	0.795	1.068	476	52.7	5.35
0.879	0.957	1.022	0.682	0.880	265	30.1	9.76

0.745	0.899	0.988	0.598	0.836	309	33.7	9.48
0.908	0.997	1.002	0.834	0.998	240	27.9	8.56
0.868	1.027	1.074	0.744	0.806	139	15.4	4.97
1.037	0.851	0.940	0.757	1.097	174	20.0	9.77
1.133	0.929	0.976	0.872	1.012	467	52.4	6.27
0.876	1.077	0.972	1.365	0.916	174	20.6	6.96
1.199	1.055	1.020	1.083	1.294	312	34.7	9.26
0.722	0.973	0.936	0.833	0.929	494	54.6	9.69
0.993	0.953	0.902	1.067	0.961	385	43.2	9.54
1.092	1.152	1.123	0.695	1.006	118	14.3	8.69
0.971	1.015	0.983	0.805	0.884	201	22.8	5.11
1.139	1.030	0.965	0.924	1.169	405	46.4	5.97
1.151	1.020	1.072	0.965	1.081	176	19.6	10.05
1.076	0.887	1.005	0.334	1.615	61	7.2	10.77
0.793	0.947	0.983	1.137	0.952	453	51.1	5.16
0.886	0.914	1.024	0.899	1.030	251	28.1	5.72
1.157	1.113	1.032	1.036	1.403	306	35.5	8.22
0.959	1.113	1.282	0.854	1.055	227	24.9	10.24
1.004	0.967	1.055	0.861	1.002	278	32.6	9.13
0.826	1.016	0.987	1.049	0.875	282	30.4	6.89
1.074	0.931	1.134	0.876	1.068	315	36.0	9.36
0.977	0.937	1.008	1.428	1.028	222	25.9	7.97
1.007	0.890	0.984	0.757	0.967	224	25.0	8.82
0.988	1.113	0.921	0.951	0.828	75	9.0	8.37
0.633	1.000	0.955	0.558	1.081	234	25.8	8.16
1.086	0.994	1.073	1.111	1.000	274	32.2	9.29
0.876	1.012	1.062	1.397	1.049	319	36.7	8.98
1.074	0.977	1.081	0.982	1.290	311	37.1	8.18
1.081	0.983	0.713		1.014	536	60.7	9.36
1.040	1.091	1.080	1.110	1.038	256	29.2	5.22
1.070	0.965	1.108	0.713	0.916	272	29.8	8.91
0.824	0.842	0.956	0.639	0.955	443	47.1	8.65
0.938	1.046	0.943	1.173	0.892	102	11.7	5.01
1.578	0.995	1.126	1.917	1.523	341	38.4	8.98
0.694	0.788	1.019	1.475	0.872	199	22.0	9.88
1.123	0.984	1.018	0.946	0.925	169	18.9	9.20
0.763	1.008	0.950	0.606	1.014	223	25.2	9.80
0.836	0.842	1.049	0.876	0.938	438	47.7	7.52
0.933	1.061	0.936	1.127	0.834	263	30.3	6.19
1.241	1.087	1.175	1.318	1.235	67	7.8	8.69
0.744	0.882	0.949	0.535	0.978	271	29.9	9.09
1.177	1.058	0.960	1.085	1.174	311	35.5	9.42
0.963	1.024	1.012	0.968	1.112	212	24.2	8.81
0.875	1.044	1.000	0.776	0.899	392	46.8	9.80
1.034	0.999	0.981	1.206	1.354	210	24.0	8.75
1.033	1.208	0.968	1.166	1.112	172	20.1	9.01
0.881	0.950	1.018	0.980	1.133	834	91.7	7.74
0.966	0.868	1.103	1.113	1.344	228	25.8	5.60
1.172	1.073	0.989	0.921	1.333	178	19.8	7.20
0.833	1.010	1.120	0.818	0.895	249	27.0	10.51
1.087	0.950	0.988	0.850	0.856	399	45.8	5.30
0.997	0.980	0.994	0.900	1.110	501	57.3	9.35
0.895	1.020	1.024	0.929	0.996	219	24.8	9.45
0.873	0.916	0.943	0.740	0.962	254	28.4	9.83
0.702	0.965	1.046	1.397	0.936	117	13.4	8.15

0.950	0.962	0.944	0.842	0.985	833	94.2	5.43
1.001	0.882	1.022	0.780	1.196	304	35.2	7.68
1.276	0.928	1.031	0.991	1.247	636	71.7	7.06
0.729	0.918	0.944	0.596	0.839	483	54.8	9.38
1.576	1.070	1.079	0.994	1.559	338	37.3	5.14
0.835	1.041	1.035	0.760	0.963	245	28.2	9.89
1.108	1.044	0.948	0.765	0.924	207	25.0	9.77
1.004	1.060	1.030	0.939	1.002	209	23.0	5.69
1.159	1.118	0.922	0.484	1.042	334	37.3	5.69
1.285	0.961	1.042	1.490	1.704	163	19.3	9.25
1.093	1.125	1.201	0.692	1.006	413	48.3	9.63
0.919	1.230	1.473	1.156	0.818	270	30.8	9.83
1.074	1.104	0.898	0.913	1.035	250	26.5	7.44
1.002	1.175	0.906	1.139	0.913	186	20.9	5.96
1.018	0.993	1.011	0.783	1.308	943	108.5	8.51
0.941	0.988	0.773	1.611	1.051	344	38.4	9.76
0.918	1.204	1.014	0.794	0.854	395	43.8	6.52
0.665	1.018	1.003	1.150	0.988	120	14.5	8.75
0.816	0.991	0.971	0.927	0.879	561	63.0	9.76
0.933	1.014	1.114	0.588	0.902	757	83.1	6.71
0.926	0.999	1.149	1.677	1.158	234	26.8	10.02
1.167	1.087	0.951	0.983	1.172	166	18.8	5.05
0.814	0.958	1.006	1.141	0.904	353	39.4	6.38
0.937	0.887	1.037	0.841	1.150	343	39.3	6.16
1.118	0.918	1.105	0.894	1.122	159	18.1	9.26
1.165	0.917	0.991	1.138	1.147	157	18.0	9.50
0.832	1.091	1.069	0.910	1.144	159	18.5	9.64
0.981	1.114	1.071	0.676	0.857	418	46.0	8.54
0.926	1.095	1.055	1.358	0.849	237	26.8	9.22
0.813	1.009	0.944	0.440	0.956	338	36.9	9.60
1.296	1.156	1.060	2.740	1.340	146	16.7	4.64
1.121	1.018	1.166	0.670	1.270	328	37.6	5.64
1.102	1.043	1.129	0.945	1.261	145	16.7	6.80
0.880	0.894	1.009	0.578	0.978	186	20.8	9.67
1.159	1.100	1.137	0.877	1.399	177	20.1	5.71
0.837	1.134	1.074	1.161	0.978	388	45.6	6.67
1.045	1.000	1.020	0.819	1.359	257	29.8	5.99
0.762	0.951	1.011	0.399	0.934	277	32.5	5.15
0.887	0.944	1.027	0.902	0.845	107	12.5	4.50
0.895	1.112	1.035	0.991	1.011	673	74.6	9.36
1.025	1.079	1.005	0.754	0.977	152	18.0	9.66
2.131	0.982	0.999	0.658	1.058	46	5.2	10.29
0.952	0.960	0.989	1.093	0.955	285	32.0	8.06
0.927	1.046	0.929	1.677	1.060	685	78.3	9.51
0.921	1.063	1.056	0.559	0.949	173	19.5	8.91
1.085	1.072	0.919		1.139	91	10.1	8.29
1.063	0.991	1.069	0.553	0.994	590	68.2	9.73
1.179	1.061	0.954	0.794	1.132	261	29.9	5.39
1.056	1.075	0.964	1.056	1.227	170	19.1	5.03
1.031	0.988	1.053	0.710	1.263	218	25.1	7.59
0.977	1.042	1.030	0.945	0.925	188	21.3	9.70
0.865	1.043	0.931	0.487	0.996	248	27.8	6.39
0.886	1.008	1.014	0.759	0.885	685	75.9	9.54
0.725	1.094	1.010	1.011	0.967	104	12.0	6.14
1.040	0.933	0.949	0.922	0.868	312	35.1	6.20

0.718	0.939	0.931	0.727	0.980	189	22.1	9.58
1.184	1.254	0.949	0.742	1.080	448	52.2	9.63
0.772	1.242	1.066	0.821	0.886	310	33.4	10.14
1.170	1.234	1.214	1.306	1.135	727	77.1	5.74
0.926	1.270	1.182	0.754	0.872	90	10.2	6.79
0.915	1.007	0.923	0.693	0.780	164	18.3	6.21
0.982	0.935	0.916	0.703	1.099	665	74.1	7.39
1.159	0.947	1.027	0.469	1.436	273	30.0	5.20
1.000	0.964	0.734	1.026	0.725	889	97.0	5.47
1.240	0.985	0.952	1.840	1.228	122	14.3	9.25
0.735	1.020	0.933	0.578	0.904	457	50.5	9.41
1.179	1.019	1.017	0.995	1.333	60	6.9	5.06
0.856	1.146	0.957	0.634	0.921	217	24.3	8.38
1.074	1.058	0.916	1.183	0.879	288	32.7	9.86
0.939	0.890	0.891	0.707	0.827	155	16.9	10.07
1.057	1.089	0.930	0.752	0.928	767	87.8	8.06
1.291	1.053	1.062	0.584	1.020	46	5.5	12.60
0.824	0.874	0.994	1.599	0.918	576	64.3	8.56
1.227	1.039	0.983	2.221	1.074	128	15.2	7.42
0.978	1.030	1.162	0.786	0.970	99	11.2	8.09
0.909	0.932	0.991	0.734	0.948	291	33.2	10.18
1.034	0.978	1.121	0.731	1.003	179	21.1	10.29
0.960	1.147	1.022	1.219	0.794	133	15.2	4.70
0.957	1.043	1.095	0.745	1.095	206	23.2	8.78
0.822	1.039	0.979	0.795	0.849	805	88.7	8.75
0.997	0.941	0.997	0.604	0.922	362	38.9	9.74
1.750	1.078	0.919	0.672	1.112	61	6.9	11.77
0.953	0.969	0.992	0.589	0.989	525	58.0	9.09
1.293	0.993	1.059	2.154	1.169	207	23.3	5.47
0.994	0.999	0.995	1.096	0.897	132	15.6	9.10
1.028	1.152	1.172	0.551	0.956	63	7.2	4.55
0.911	1.119	1.028	0.743	0.842	161	18.8	9.16
1.311	1.218	1.011	0.973	1.225	157	18.8	8.40
0.388	0.696	0.915	0.726	1.117	58	6.7	11.22
0.983	0.926	0.834	0.600	0.942	272	30.1	9.52
1.000	1.029	0.922	0.693	1.035	285	32.5	8.10
1.150	1.064	1.283	1.178	1.181	418	49.2	8.97
0.701	0.888	1.003	0.846	0.716	274	31.2	9.06
1.366	0.820	1.429	2.466	1.454	323	38.3	9.61
0.689	1.125	1.108	0.689	0.839	316	34.9	10.11
0.806	0.973	0.942	0.740	0.994	618	69.1	6.21
0.697	0.621	1.070	0.661	0.772	355	38.6	7.93
0.771	1.029	1.018	0.699	0.940	469	50.0	9.58
1.104	0.950	1.067	0.871	0.859	442	47.2	8.84
0.934	1.038	1.134	0.981	0.884	109	12.7	4.53
1.147	0.984	0.992		1.044	87	10.0	7.50
1.262	1.046	1.026	0.849	1.450	415	48.4	7.18
1.073	0.976	1.077	0.728	1.023	640	68.4	5.86
1.124	1.048	0.994	0.794	0.984	145	16.2	5.10
1.205	0.892	0.939	0.891	0.835	246	27.9	8.63
0.648	1.070	0.942	0.666	0.771	66	7.8	4.83
0.815	0.880	1.012	0.490	1.067	55	6.4	9.70
0.903	0.932	1.070	0.645	0.982	295	33.1	9.45
1.193	0.994	1.132	1.011	1.230	611	69.9	7.46
0.923	1.270	0.983	1.046	0.863	200	22.7	6.81

0.845	0.930	1.069	0.513	1.014	141	16.1	9.67
0.808	0.956	0.969	0.949	0.901	344	39.3	9.69
0.937	0.957	0.964	1.171	1.136	79	9.2	9.31
1.011	0.806	1.151	0.783	1.195	289	33.3	8.56
0.795	0.962	1.279		0.838	671	74.7	9.47
0.951	1.106	1.039	1.578	0.855	253	28.3	8.98
0.765	1.001	1.102	0.602	0.874	341	37.1	10.05
0.929	0.833	1.096	1.170	0.992	240	28.4	9.25
0.743	1.013	0.889	0.713	0.867	364	40.0	9.01
1.366	1.297	1.012	0.783	1.396	228	25.8	9.31
1.114	1.009	1.016	1.317	1.357	120	13.8	9.64
1.147	0.976	1.076	0.902	1.021	171	19.6	10.15
0.991	0.924	0.956	0.622	1.052	428	47.1	5.30
0.777	0.841	0.983	0.638	0.783	797	91.1	6.58
0.875	1.014	1.138	0.706	0.952	377	42.1	8.73
0.970	0.978	1.021	0.777	0.931	236	27.7	5.72
0.978	1.155	1.157	0.615	0.957	337	38.2	9.41
0.796	0.936	1.077	0.698	1.062	125	14.8	10.29
0.792	1.067	1.077	0.514	1.059	423	48.5	9.48
1.412	1.104	1.031	0.733	1.292	192	21.3	6.39
1.214	1.038	1.153	1.252	1.170	49	5.5	10.18
1.351	2.458	0.995	0.698	1.293	541	56.8	4.84
0.872	1.057	1.081	0.561	0.751	342	36.4	4.93
1.279	0.914	1.138	2.261	2.126	358	40.3	5.17
1.253	1.119	1.094	0.615	1.157	244	27.5	9.28
0.864	1.056	0.896	0.368	0.849	212	24.5	9.55
1.281	1.126	1.035	0.814	1.298	343	40.4	8.25
0.871	0.977	1.097	0.687	1.104	897	98.5	9.03
0.645	0.746	0.606		0.699	310	33.0	4.84
0.927	1.039	1.103		0.841	803	90.8	6.14
1.187	1.019	0.972	0.399	1.050	449	51.9	9.14
1.339	1.088	0.859	0.993	1.382	368	43.3	9.67
0.997	1.156	0.805	0.383	1.330	285	33.5	5.41
1.087	1.258	0.957	0.694	1.004	172	19.6	8.82
0.518	0.993	1.304		0.697	185	21.5	10.30
0.947	0.961	1.132		0.818	216	24.2	9.14
0.546	1.043	0.932	1.084	0.723	1070	123.2	6.60
0.929	1.099	1.053	0.508	1.141	228	25.8	7.14
0.648	1.068	0.961	0.570	0.663	353	38.6	4.45
0.995	0.849	0.981	0.990	1.198	249	27.3	9.16
2.312	1.202	0.959	2.593	1.415	69	7.8	9.99
0.632	0.753	0.964	1.414	1.174	494	55.4	5.48
0.912	0.911	1.085	0.687	0.962	589	65.8	6.48
1.174	1.049	0.957	1.750	1.281	244	27.9	8.00
0.770	1.107	1.224	1.346	0.516	390	43.5	5.99
0.992	1.234	0.804	0.428	1.252	217	24.0	9.14
1.104	0.935	0.962	0.564	0.978	405	45.4	9.52
0.953	1.009	1.025	0.721	0.905	227	25.0	9.73
1.045	1.306	1.195	0.675	1.060	1002	114.4	6.74
0.590	0.349	2.751	0.335	1.136	381	41.2	5.96
0.964	1.066	1.082	0.742	1.143	344	39.4	9.36
1.357	1.259	0.884	0.768	0.804	392	45.9	8.10
0.976	0.956	0.822		0.876	368	40.9	9.51
1.030	1.059	1.208	0.597	1.087	212	24.6	9.45
1.068	0.893	0.696		1.265	750	85.8	9.92

0.851	0.878	0.979	0.678	0.655	828	96.5	5.48
1.070	0.863	1.030	1.729	0.934	436	47.5	8.16
1.066	1.010	0.998	0.681	0.982	407	46.7	6.65
0.675	1.021	1.038	0.523	0.821	270	30.9	10.18
1.163	0.919	1.172	1.280	1.209	369	41.9	8.92
1.437	1.214	0.933		1.473	320	36.2	8.78
0.750	0.953	0.947	0.707	0.826	114	13.3	4.86
0.934	1.260	1.217	0.603	1.078	317	34.9	9.11
0.933	1.084	0.961	1.080	0.774	306	35.6	9.58
0.721	0.900	0.852	1.020	0.745	89	10.1	9.57
1.066	1.235	1.055	0.746	0.987	170	20.3	5.87
0.990	1.135	1.105	0.713	0.972	417	48.6	5.38
0.861	0.934	1.093	0.580	0.898	367	42.6	9.67
1.423	1.730	1.051		1.032	174	20.2	9.17
1.278	1.205	0.854	0.475	1.065	574	67.1	6.38
1.346	0.959	0.895	0.784	1.118	676	76.0	7.72
0.949	0.990	0.970	0.625	1.057	673	75.3	5.66
1.016	1.344	1.027	0.810	0.936	296	33.6	9.52
0.678	0.919	0.926	1.142	0.636	350	41.0	8.78
0.884	0.988	1.051	0.736	0.995	213	24.6	10.07
1.086	1.543	0.862		0.754	755	88.1	6.47
0.862	0.929	1.013	0.640	0.975	551	61.7	9.26
1.209	1.227	1.216	0.538	1.342	106	12.3	4.77
1.071	0.857	1.109	0.725	1.074	168	19.8	9.80
1.170	1.079	1.417	1.185	1.132	375	43.0	8.97
1.000	1.263	1.044	0.513	1.045	141	15.7	5.68
0.977	0.973	1.019	1.309	0.749	196	21.9	5.67
0.755	1.179	1.023	1.270	0.773	202	22.6	9.23
1.077	1.043	0.985	0.741	1.174	144	17.0	9.48
0.613	0.827	1.054		0.429	137	16.2	9.44
1.008	0.963	1.069	0.777	1.015	805	88.9	9.01
0.881	1.033	0.988	0.830	0.784	141	15.4	9.41
1.053	0.978	1.039	0.612	1.238	119	13.1	9.17
0.802	0.617	0.754		1.693	553	63.7	5.81
0.746	0.927	1.094	1.027	0.743	511	56.9	5.90
1.131	0.672	0.747		1.060	270	29.6	9.26
0.992	1.006	0.912	1.253	1.035	266	30.9	7.62
1.078	1.091	1.113	1.612	0.968	449	48.8	9.36
1.025	0.962	1.005	0.835	0.939	300	35.7	9.54
1.094	1.156	1.044	0.442	1.162	310	36.7	8.94
1.170	0.968	0.993	1.028	1.037	534	59.5	8.60
0.881	1.005	1.051	0.579	0.959	501	58.1	9.55
1.051	1.066	1.146	1.094	0.962	220	24.8	9.07
0.993	1.039	1.115	0.799	1.119	58	7.0	8.41
1.456	1.072	1.181	1.407	1.589	232	25.8	4.72
0.907	1.068	0.955	1.203	1.009	289	32.2	5.47
0.805	0.950	0.950		0.890	338	38.2	8.35
1.297	0.885	1.168		1.518	344	39.6	9.55
1.050	0.943	1.248	0.379	1.156	337	38.4	9.28
1.717	1.248	0.853	0.277	1.935	207	24.0	9.48
2.290	0.602	4.358	1.998	1.084	298	32.7	5.80
1.039	1.001	0.994	0.904	1.264	281	31.9	9.54
1.038	0.984	1.169	0.337	1.216	125	15.0	9.88
1.184	0.941	1.056	0.870	1.157	757	85.1	5.22
0.805	0.874	0.980	2.757	0.724	395	46.4	9.32

1.662	1.286	1.001	1.195	0.961	104	11.7	9.58
1.004	1.353	1.092	0.513	1.039	443	50.3	8.10
1.034	0.986	0.906	1.554	0.811	402	47.0	9.67
1.074	1.065	1.114	1.022	0.893	95	11.0	9.73
0.988	0.982	0.999	0.576	1.187	172	19.6	9.33
1.088	0.949	1.003	1.012	0.987	269	30.9	6.96
0.911	1.110	1.182	1.452	0.958	519	56.6	9.60
0.954	1.032	1.020	1.678	1.600	711	80.4	9.41
0.817	0.979	1.056		1.113	299	33.6	6.47
1.106	1.141	0.876	1.300	0.844	296	33.5	9.29
0.707	0.940	1.029	0.761	0.887	207	23.8	9.88
0.957	1.073	0.990	0.911	0.928	213	23.8	9.77
0.935	1.247	0.963	0.956	0.833	345	37.3	6.24
0.978	0.843	1.028	0.794	0.736	74	7.6	8.66
0.801	0.917	0.919	1.425	0.940	77	8.6	8.62
0.820	0.976	1.047	0.980	1.060	146	16.7	7.80
1.255	1.106	0.968	0.510	1.199	295	31.4	9.23
0.905	1.082	1.032	0.756	0.850	483	54.7	9.96
1.173	1.226	1.100	0.568	0.867	172	19.4	10.55
1.219	1.165	0.995	0.755	1.094	371	41.1	5.12
1.330	1.224	1.155	1.371	1.171	76	9.3	4.67
0.912	1.206	1.092	0.820	0.956	56	6.4	4.94
1.066	1.234	0.824	0.181	1.010	312	35.6	9.11
1.240	0.874	1.086	0.845	1.506	89	10.3	10.04
0.833	0.978	0.976	0.903	1.006	97	11.5	4.64
0.931	1.034	1.020	0.823	0.877	271	30.5	8.41
1.097	1.104	0.990	0.887	1.295	270	31.0	6.14
0.943	1.031	0.978	1.001	0.854	153	17.9	8.37
0.988	1.022	1.024	0.405	1.260	599	67.4	9.95
1.127	0.950	1.039	0.799	1.414	519	57.5	7.08
1.242	0.922	0.924	0.629	2.098	39	4.4	8.62
0.648	0.992	1.035	1.136	0.854	229	26.3	8.90
1.197	1.161	1.148		0.883	158	17.6	6.11
1.049	1.086	1.034	0.935	0.916	310	34.9	7.53
0.778	1.148	0.923	1.307	1.023	192	22.7	9.32
0.909	0.964	1.050	0.795	1.037	176	21.2	9.74
1.073	1.380	1.117	0.571	1.288	488	55.0	9.70
0.953	1.053	1.105	0.709	1.075	147	16.9	5.24
1.581	0.959	0.957	1.027	1.370	134	15.9	9.14
1.031	0.826	0.891	1.245	0.985	489	52.8	9.64
1.329	1.176	1.009	0.407	1.472	101	11.8	7.49
2.260	0.820	0.905	1.417	1.432	55	5.6	11.43
0.752	0.680	1.243	0.887	0.693	69	7.9	4.77
0.850	1.654	1.491	0.996	1.001	226	26.0	8.60
0.907	0.940	0.876		0.898	148	16.9	9.23
0.882	1.058	0.951	1.106	0.843	148	16.8	8.69
0.860	0.949	0.977	1.255	1.031	260	29.2	9.54
0.849	1.204	0.875	0.687	0.979	149	16.1	10.15
1.062	1.043	1.043	1.163	1.202	315	37.3	9.19
1.070	0.914	1.112	1.268	0.974	214	25.0	5.77
0.925	1.012	0.861		0.850	113	12.7	8.82
0.848	0.995	0.997	1.033	0.894	392	46.1	9.64
0.786	1.038	0.960	0.425	0.850	387	42.0	9.92
0.902	1.064	0.928	1.226	0.890	256	28.2	5.29
0.803	1.029	1.039	1.036	0.921	301	33.5	10.07

0.954	1.267	0.983	1.080	0.885	119	14.1	4.54
0.874	1.153	0.997	0.825	1.064	113	13.1	5.00
1.281	0.992	1.058	0.888	1.535	246	28.4	5.96
1.108	1.209	0.816		1.021	300	34.4	9.22
0.359	0.655	1.342	0.895	0.164	167	19.6	7.33
0.923	1.030	0.946	0.977	0.865	619	68.8	5.90
0.791	0.909	1.048	0.837	0.918	180	20.8	9.55
1.375	1.004	1.079	1.358	1.402	329	37.1	8.91
0.976	1.026	1.092	0.579	0.843	116	13.5	5.08
1.103	1.199	1.179	0.931	0.926	369	43.5	4.89
0.752	1.073	0.854	0.470	0.701	140	15.0	9.70
1.082	0.993	1.128	0.887	1.208	38	4.1	4.91
0.642	0.421	1.838	1.435	0.884	114	13.0	5.77
1.064	1.250	1.229	0.412	1.167	461	49.8	9.04
0.901	1.000	0.720	1.097	1.046	73	8.6	10.01
0.794	0.971	1.268	0.467	0.945	228	26.1	9.44
0.972	0.992	0.953	0.475	0.787	436	46.5	8.53
0.914	0.964	0.861	1.025	0.834	231	27.1	9.33
0.703	0.922	1.050	0.782	0.709	486	53.0	9.63
1.163	1.036	1.062	1.217	0.772	142	16.7	8.41
0.924	1.000	1.059	0.763	1.010	276	31.8	9.42
1.236	1.167	0.956	1.963	1.170	162	18.8	5.43
0.913	0.964	1.022	0.836	0.806	125	13.6	8.46
1.329	1.075	1.123	0.799	1.052	291	31.8	5.80
0.917	0.982	0.814	0.642	1.173	238	26.8	9.01
0.894	0.947	0.946		0.834	282	32.5	9.11
0.923	1.099	1.129	0.796	0.506	93	10.9	6.29
1.240	1.019	0.971	0.977	1.175	137	15.4	7.24
1.023	1.186	1.003	0.873	1.128	214	25.5	6.20
1.505	1.051	0.902	2.695	0.925	58	6.9	6.00
1.123	0.528	0.804		1.266	35	4.1	4.61
0.603	0.991	1.023	0.827	0.602	199	22.5	9.47
1.339	1.106	1.094	1.257	1.298	242	27.9	9.92
0.798	1.174	1.045	0.553	0.833	322	36.6	8.60
0.670	0.936	0.960	0.582	0.941	39	4.5	8.50
1.003	1.041	0.951	0.912	0.899	249	28.2	5.38
0.778	0.888	1.189	0.701	0.910	230	25.9	9.51
0.819	0.961	1.015	1.793	0.559	163	18.3	9.94
1.012	1.201	0.934	1.131	0.931	234	25.3	9.20
0.746	1.295	1.048	2.277	0.724	120	14.2	10.11
0.868	0.873	1.144	0.720	0.885	368	39.4	9.47
1.180	0.998	0.981	0.857	0.923	105	11.5	8.12
0.999	1.133	1.021	1.219	1.174	340	38.3	9.92
0.796	1.288	1.051	1.021	0.800	62	7.4	6.10
					45	5.0	8.68
0.739	0.922	0.930	0.650	0.849	65	7.7	7.18
0.872	1.474	1.436	0.570	1.232	50	5.8	9.66
1.214	1.123	1.114		0.916	45	5.4	10.55
